# Biodiversity of protists and nematodes in the wild nonhuman primate gut

**DOI:** 10.1038/s41396-019-0551-4

**Published:** 2019-11-12

**Authors:** Allison E. Mann, Florent Mazel, Matthew A. Lemay, Evan Morien, Vincent Billy, Martin Kowalewski, Anthony Di Fiore, Andrés Link, Tony L. Goldberg, Stacey Tecot, Andrea L. Baden, Andres Gomez, Michelle L. Sauther, Frank P. Cuozzo, Gillian A. O. Rice, Nathaniel J. Dominy, Rebecca Stumpf, Rebecca J. Lewis, Larissa Swedell, Katherine Amato, Laura Wegener Parfrey

**Affiliations:** 1 Department of Botany and Biodiversity Research Centre, University of British Columbia, Vancouver, BC Canada; 2 Department of Microbiology, Immunology, and Genetics, University of North Texas, 3500 Camp Bowie Blvd, Fort Worth, TX 76107 USA; 3 Estacion Biologica Corrientes (MACN-BR)—CONICET, Corrientes, Argentina; 4 Department of Anthropology, University of Texas Austin, Austin, TX USA; 5 Department of Biological Sciences, Universidad de los Andes, Bogota, Colombia; 6 Department of Pathobiological Science, University of Wisconsin-Madison, Madison, WI USA; 7 School of Anthropology, University of Arizona, Tucson, AZ USA; 8 Department of Anthropology, Hunter College of City University of New York, New York, NY USA; 9 Department of Animal Science, University of Minnesota, St. Paul, MN USA; 10 Department of Anthropology, University of Colorado Boulder, Boulder, CO USA; 11 Lajuma Research Centre, Louis Trichardt (Makhado), Lajuma, South Africa; 12 Department of Anthropology, Dartmouth College, Hanover, NH USA; 13 Department of Anthropology, University of Illinois at Urbana-Champaign, Urbana, IL USA; 14 Department of Anthropology, Queens College, City University of New York, Flushing, New York, NY USA; 15 Department of Archaeology, University of Cape Town, Cape Town, South Africa; 16 Department of Anthropology, Northwestern University, Evanston, IL USA; 17 Department of Zoology, University of British Columbia, Vancouver, BC Canada

**Keywords:** Biodiversity, Microbial ecology

## Abstract

Documenting the natural diversity of eukaryotic organisms in the nonhuman primate (NHP) gut is important for understanding the evolution of the mammalian gut microbiome, its role in digestion, health and disease, and the consequences of anthropogenic change on primate biology and conservation. Despite the ecological significance of gut-associated eukaryotes, little is known about the factors that influence their assembly and diversity in mammals. In this study, we used an 18S rRNA gene fragment metabarcoding approach to assess the eukaryotic assemblage of 62 individuals representing 16 NHP species. We find that cercopithecoids, and especially the cercopithecines, have substantially higher alpha diversity than other NHP groups. Gut-associated protists and nematodes are widespread among NHPs, consistent with their ancient association with NHP hosts. However, we do not find a consistent signal of phylosymbiosis or host-species specificity. Rather, gut eukaryotes are only weakly structured by primate phylogeny with minimal signal from diet, in contrast to previous reports of NHP gut bacteria. The results of this study indicate that gut-associated eukaryotes offer different information than gut-associated bacteria and add to our understanding of the structure of the gut microbiome.

## Introduction

Comparative studies of gut microbiome structure across phylogenetically similar but ecologically distinct host species help clarify factors that regulate microbial community assembly, structure, and stability over time. In mammals, these patterns are shaped by a variety of factors including host gut physiology, diet, phylogeny [1–6], host age, geography [7, 8], and social behavior [9, 10]. Our current understanding of microbial community dynamics is shaped by a disproportionate focus on bacteria. This bias is due in part to the fact that eukaryotes are generally studied as parasites rather than community members [11], but they can also be beneficial or neutral depending on species and context [12]. The diversity and structure of the eukaryotic microbiome across hosts remains an open question [13, 14], even though eukaryotes are important members of the gastrointestinal microbial community [15, 16].

Microeukaryotes (protists and fungi) and macroeukaryotes (helminths) influence the gut ecosystem in myriad ways. Gut eukaryotes modulate other microbes through predation, resource and niche competition, and interaction with the host immune system [12, 15, 17, 18]. For example, *Entamoeba* and *Blastocystis* are associated with major shifts in the gut microbiome [16, 19–21]. Larger gut eukaryotes can be colonized with their own suite of microbes [22], which may influence the host microbial community. Likewise, bacteria in the gut regulate eukaryotic taxa that co-colonize the same ecological niche. For instance, strains of *Escherichia coli* suppress the growth of the opportunistic pathogen *Candida albicans* [23]. Because *C. albicans* reportedly colonizes the gut of healthy humans [24–26], antagonistic relationships between eukaryotes and other microbes may promote gut homeostasis.

Nonhuman primates (NHPs) are a valuable study system for understanding underlying processes governing the ecology and evolution of the gut microbiome. Extant primate groups are descendants of several major radiations since the last common ancestor approximately 70 MYA [27, 28] and fall into multiple major clades (host “phylogroups” hereafter): (1) cercopithecoids (African and Asian monkeys), (2) hominoids (apes), (3) platyrrhines (Central and South American monkeys), and (4) strepsirhines (lemurs, galagos, and lorises). Within and across these phylogroups, wild NHPs occupy highly variable ecological and dietary niches and live in diverse social systems that range from solitary and pair-bonded family groups to large, multilevel societies [29, 30]. Given that NHPs are our closest living relatives, documentation of the wild NHP gut microbiome also provides an important evolutionary context for understanding the human gut microbiome. As human populations have shifted into more urban and industrialized lifestyles, loss of gut microbial diversity has been documented, which is linked to several chronic diseases [31–33]. This loss of diversity includes gut eukaryotes, which have been targeted by aggressive antiparasitic initiatives throughout the 20th century [34]. The presence of specific eukaryotic groups is correlated with an enrichment of bacteria that are extirpated in industrialized populations [19, 35–37], so understanding the relationships between bacteria and eukaryotes in NHPs may provide clues to how this loss of diversity occurred.

In this study, we analyzed 62 individual fecal samples from wild NHPs representing 16 species using an 18S rRNA gene fragment metabarcoding approach. NHPs were chosen to represent a wide geographic distribution including five platyrrhine species from Central and South America, six cercopithecoids and two ape species from Uganda, the Central African Republic, and Ethiopia, and three lemur species from Madagascar. We examine the effects of host phylogroup on the total eukaryotic assemblage structure, the relationship between bacterial and eukaryotic diversity, and the distribution of particular eukaryotic taxa across NHP lineages. Our results shed new light on the role of eukaryotes in microbial communities, as well as the utility of cross species comparative studies for understanding the evolution of the mammalian gut microbiome.

## Materials and methods

### Samples and DNA extraction

Raw fecal samples from 62 individual NHPs were collected and extracted as described in Amato et al. [2] and in Supplementary Methods. Metadata corresponding to samples can be found in Supplementary Table 1.

### Illumina amplicon library preparation

Extracted DNA was sent to the Integrated Microbiome Resource (IMR) laboratories at Dalhousie University, Halifax, Canada for library construction, quantitation, pooling, and sequencing following protocols in Comeau et al. [38]. Sequences were amplified with the 18S rRNA V4 targeted primers E572F (5′-CYGCGGTAATTCCAGCTC-3′) and E1009R (5′-CRAAGAYGATYAGATACCRT-3′). PCR reactions included a PNA mammal blocking primer (5′-TCTTAATCATGGCCTCAGTT-3′) to minimize host DNA amplification. Conditions for PCR are based on those described in Comeau et al. [39] but include an additional step at 65 °C to anneal the blocking primer. The final pool was sequenced on an Illumina MiSeq using paired-end 300-cycle chemistry.

### Computational analyses

**Fig. 1:**
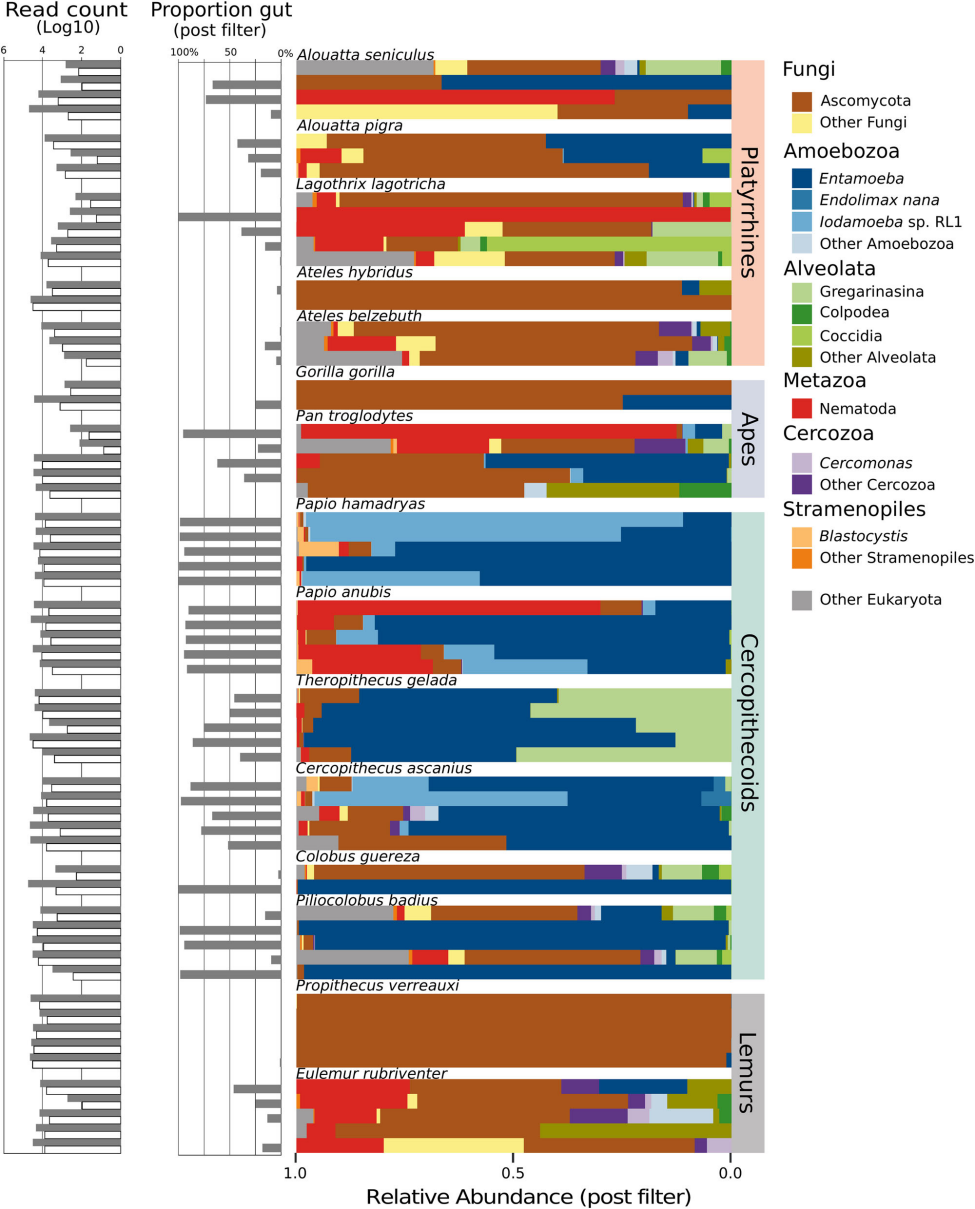
Relative abundance of taxonomic groups document Fungi-dominated and Amoebozoa-dominated eukaryotic assemblages. Platyrrhines, most apes, and lemurs tend to be dominated primarily by Ascomycota, while cercopithecoids are dominated by Amoebozoa. Corresponding histograms illustrate the log transformed number of raw reads (gray bars) and log transformed reads after insects, vertebrates, and plants were removed (white bars), as well as the proportion of reads post filter that belong to known gut residents for each sample

An average of 30,244 reads were generated per sample (±25,015) (Supplementary Table 2). Primers were removed from raw sequences using Cutadapt v.1.18 [40]. We then merged and quality filtered trimmed paired-end reads with a phred score threshold of 30 using PEAR v.0.9.10 [41]. Merged and unmerged forward reads that passed quality thresholds were checked for chimeras using VSEARCH v.2.8.1 [42]. Operational taxonomic units (OTUs) were generated with Swarm v.2.2.2 [43] as implemented in QIIME v.1.9 [44] resulting in 203,222 unique OTUs. We then assigned taxonomy to each OTU using VSEARCH v.2.8.1 and the SILVA 18S rRNA gene database v.128 [45]. Any OTU that failed to match the SILVA database was checked against the full NCBI NT database with BLAST [46] and assigned taxonomy using MEGAN v.6 [47]. OTUs observed fewer than ten times across all samples, only once within a sample, or originated from clear dietary sources or the host (i.e., plants, vertebrates, and insects) were discarded. Detected taxa and predicted source (e.g., gut, environment, and diet) are found in Supplementary Table 3. The proportion of reads retained after filtering is highly variable between individuals (x̄ 40.31 ± 25.74%) (Fig. 1). The sole *Lemur catta* individual had no reads post filtering and was removed from downstream analysis. Samples were rarefied to 500 reads per sample prior to alpha and beta diversity analyses, but taxa summaries, distributions, and presence/absence were assessed from the unrarefied data.

Paired 16S rRNA gene amplicon data were accessed from EBI (ERP104379) [2] and processed into OTUs in an identical manner as described above using the EZBioCloud database [48] to assign taxonomy before rarifying to 5,000 reads per sample. We asked whether common gut eukaryotes (*Blastocystis*, *Entamoeba*, and gut-specific nematodes) are associated with different bacterial communities using LEfSe [49]. We tested for associations between the frequency and relative abundance of bacterial genera and the presence or absence of these eukaryotes, followed by FDR correction for multiple comparisons.

Data analysis was performed in the R v.3.5.0 environment [50]. We performed phylogenetic and diversity analyses using Vegan v.2.5-3 [51], Ape v.5.2 [52], and Phyloseq [53] and generated figures using ggplot v.3.1 [54]. We performed a PERMANOVA analysis using the adonis function in Vegan v.2.5-3 [51]. Weighted UniFrac distances and alpha diversity (observed OTUs) were calculated using QIIME v1.9 [44]. As correlation between bacterial and eukaryotic alpha diversity cannot be calculated using classic linear methods because host species are not independent from each other due to shared evolutionary history [55, 56], we used a Bayesian generalized linear mixed-model (MCMCglmm) [57] to explicitly control for host phylogeny. We used host species as a random effect and simultaneously controlled for host phylogenetic effects. We ran a Bayesian model using 10^6^ iterations, discarding the first 10,000 iterations as burn-in and sampled the chain every 5,000 iterations. We tested for phylosymbiosis following the recommendation of Mazel et al. [58], using a Mantel test to measure the correlation between pairwise beta diversity (weighted UniFrac) and host species phylogenetic distance (in millions of years) using the Vegan R package with a Pearson correction. The Mantel statistic significance was measured using 999 permutations. As the data contains multiple individuals per species, we randomly sampled a single individual per species and recorded the correlation statistic and its significance. We repeated this procedure 100 times. Corresponding scripts for both PERMANOVA and Mantel tests are available at https://github.com/FloMazel/Primates_eukaryome_diversity_Analysis.

We built reference phylogenetic trees based on published phylogenies for *Entamoeba* [59] and *Blastocystis* [60] which were then expanded each with the EukRef curation pipeline [61]. A nematode reference tree was generated from the SILVA v.128 99% clustered reference database. Sequences were aligned using Mafft v.7.407 [62] and maximum likelihood trees constructed with RAxML v.8.1.20 [63]. Phylogenetic placement of OTUs from this study was performed using RAxML with the reference tree as constraint. Some OTUs are collapsed into a single branch in the tree when clustered sequences had the same distribution across individual samples and one OTU had a higher read count (or when low abundance OTUs were found in a subset of samples), as these low abundance OTUs are often generated by sequencing artifacts. Finally, a statistical parsimony network [64] for *Iodamoeba* phylotypes was built using PopART [65] instead of a tree because *Iodamoeba* diversity is quite high and very few reference sequences are available for comparison [66]. The full OTU frequency table and metadata, as well as scripts used to process data and generate figures are at https://github.com/aemann01/primateEuk. Raw data and metadata are uploaded to the European Nucleotide Archive under accession number PRJEB32407.

## Results

### Overview of the total eukaryotic assemblage

Eukaryotic taxa detected across NHP species include organisms that reside in the gut along with many non-gut residents. Read counts were generally high after quality filtering (mean 20,377 reads, range 60–65,515), but in some samples large portions were dropped when removing clear dietary (plants, insects) or host reads (mean 9,822 reads, range 0–50,814; Fig. 1). After filtering, we find a eukaryotic gut assemblage composed of diverse gut residents as well as organisms that are likely dietary, transient, or postdepositional eukaryotes (Fig. 1 and Supplementary Fig. 1). While we aim to study only true NHP gut residents, identifying them is dependent on the taxonomic resolution of the data as well as *a priori* knowledge of which organisms colonize the NHP gut. Thus, we analyze diversity patterns for the whole eukaryotic gut assemblage to facilitate comparison with subsequent studies before focusing on clear gut residents.

**Fig. 2:**
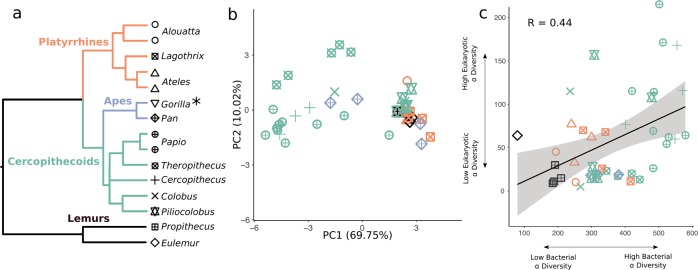
Diversity patterns of eukaryotes in the NHP gut microbiome. **a** Phylogeny of NHP species in this study. Shapes throughout figures correspond to NHP genus, color indicates NHP phylogroup. *No gorilla samples were included in diversity analyses because of low read counts. **b** PCoA of weighted UniFrac distances of the full eukaryotic assemblage. The majority of variance (69.75%) separates the cercopithecines and two apes from all other NHPs. The single *Papio* individual clustering away from the *Papio* group has the lowest overall proportion of Amoebozoa as compared with other members of its genus. **c** Higher alpha diversity in the eukaryotic assemblage is positively correlated with higher bacterial alpha diversity using a simple linear model (Pearson product moment correlation *R* = 0.44), a pattern that is also supported when host phylogeny is accounted for (*p* = 0.01)

The eukaryotic gut assemblage in our wild NHPs follows two general patterns—(1) high variability across individuals within most species and (2) a tendency toward Ascomycota or Amoebozoa dominated communities (Fig. 1). A PCoA of weighted UniFrac distances calculated from the eukaryotic gut assemblage illustrates the separation between NHPs with Ascomycota and Amoebozoa dominated profiles along PC1, which explains 69.75% of the variation (Fig. 2b). Overall, we find that host species explains most of the variation in beta diversity, measured by weighted UniFrac (PERMANOVA: R2 = 0.46, *p* = 0.001), largely driven by the cercopithecine group. The effect of host phylogroup is weak (PERMANOVA: R2 = 0.13, *p* = 0.001) and the effect of diet is weaker, though significant (PERMANOVA: R2 = 0.05, *p* = 0.001). We found no evidence for phylosymbiosis, as the correlation between beta diversity and host phylogenetic distance was slightly negative and nonsignificant (Supplementary Fig. 2).

Few OTUs are shared across species and variability is high within species, with many low abundance OTUs of likely dietary or postdepositional origin. Thus, we favor weighted UniFrac because it accounts for phylogenetic signal in the microbiota and emphasizes common taxa [67]. We show alternative distance measurements for comparison and note that the patterns observed are not robust across ordination methods (Supplementary Fig. 3), supporting our conclusion that it is best to focus efforts on true gut residents rather than the total eukaryotic gut assemblage.

The cercopithecines, and in particular members of *Papio* (baboons) and *Cercopithecus* (red tailed monkeys), harbor the highest diversity (Supplemental Fig. 4) and highest number of reads assigned to gut residents (Fig. 1). In fact, all clades of gut residents found in this dataset are represented in the cercopithecines. This is in contrast to species such as *Propithecus verreauxi* (Verreaux’s sifaka lemur) and *Ateles* (spider monkeys), which have few reads assigned to gut residents. This result is intriguing and will motivate wider surveys of wild NHP species aimed at examining this pattern. Perhaps the cercopithecines uniquely harbor the breadth of potential diversity of eukaryotic gut residents because of their evolutionary history (immune system tuned towards tolerating rather than rejecting “parasites”) or their life style (large social groups). Alternatively, we may have inadvertently sampled low diversity species in other NHP groups. For example, in the current study, the platyrrhine monkeys are represented by only one of the three major NHP clades (Atelines) found in the Americas.

The richness of gut eukaryotic and bacterial communities is correlated, but this relationship is highly dependent on host species. Overall, bacterial and eukaryotic diversity are positively correlated using a linear model (Pearson: 0.44; 95% CI: 0.15–0.65) (Fig. 2c), a relationship that is significant after controlling for host phylogeny (*p* = 0.01).

Individual gut-associated eukaryotes are associated with specific bacterial genera. For example, the bacterial genus *Oscillibacter* is enriched in the presence of nematodes (effect size: 4.38, pFDR = 0.03) and *Blastocystis* (effect size: 4.24, pFDR = 0.00005), while *Lachnopiraceae* is enriched in *Entamoeba* positive individuals (effect size: 4.59, pFDR = 0.03). Interestingly, we find the bacterial genus *Treponema* is significantly enriched in individuals that carry one or more of the major eukaryotic groups tested (Nematoda pFDR = 0.03*, Entamoeba* pFDR = 0.05, and *Blastocystis* pFDR = 0.0003), an association also found in human studies of the gut microbiome [19].

In contrast to reports that the human fungal microbiome is dominated by yeasts belonging to the genera *Saccharomyces, Candida*, and *Malassezia* [26], we find no dominant fungal taxa shared across NHPs. Of all OTUs assigned to fungi, 52.91% are unique to the individual they are found in, and only 4.53% are found in ten or more individuals. The most ubiquitous fungal taxa found across NHPs include *Pestalotiopsis maculans* (52.38%) and *Cladosporium herbarum* (46.03%), both of which are common environmental species [68, 69].

**Fig. 3:**
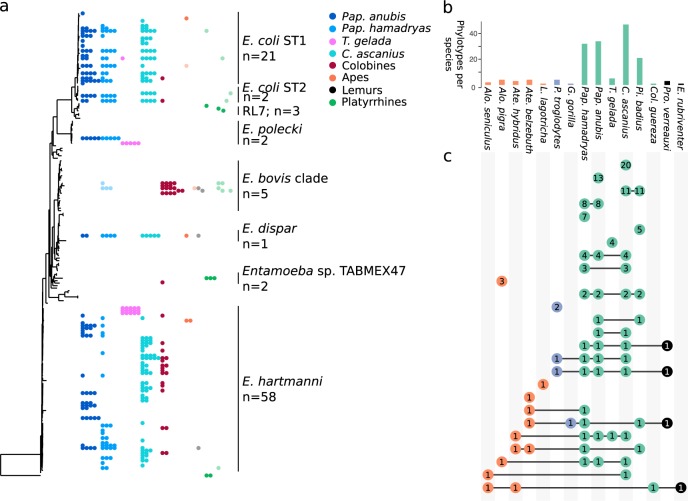
Phylotypes of *Entamoeba* display both host specificity and sharing across NHP groups. **a** Maximum likelihood tree of *Entamoeba* phylotypes with *Entamoeba* species and number of phylotypes indicated. Each dot represents a single NHP individual and color indicates NHP species. Each row of dots indicates a single phylotype corresponding to the phylogenetic tree. Lighter colored dots reflect samples in which an OTU was detected but at much lower relative abundance (below 0.001). Detection may therefore be an artifact of sequencing and should be interpreted with caution. **b** Bar chart summarizing the number of phylotypes found in each NHP species colored by NHP phylogroup. **c** Upset plot illustrating host-specificity of *Entamoeba* phylotypes. Unconnected dots indicate phylotypes found only in one NHP species. Connected dots indicate phylotypes that are shared between two or more NHP species. Numbers within dots indicate the number of shared phylotypes. Colors reflect NHP phylogroup

We detect various protists known to reside in the primate gut (e.g., *Entamoeba* sp*., Blastocystis* sp*., Iodamoeba* sp., and *Endolimax nana*), as well as those that are likely postdepositional (e.g., colpodid ciliates that are common in soils and *Copromyxa*, which colonizes feces [70]) or arrive in the primate gut as a result of consumption of insects (e.g., gregarine apicomplexans, which are common symbionts in insects and other animals, but are not residents of the vertebrate gut), plants (*Pythium* and other plant saprotrophs), or spores. *Entamoeba* sp. are the most widespread of the gut-associated protists found in 83.87% of all NHP individuals followed by *Iodamoeba* sp. (48.39%) and *Blastocystis* (30.64%) subtypes ST1, ST2, ST3, ST8, and ST11 (Supplemental Fig. 5). The relative abundance and number of *Blastocystis* OTUs detected (*n* = 15) was unexpectedly low, reflecting a poor match between *Blastocystis* and the primers used here. While most gut-associated protist clades span multiple NHP species, others show a limited distribution. For instance, the ciliate *Troglodytella abrassarti* which promotes hindgut fermentation in chimpanzees [71], was detected in two chimpanzees here. Within *Entamoeba* we also see a mix of widespread phylotypes (e.g., *Ent. dispar, Ent. coli*) and species-specific phylotypes (*Ent. polecki, Ent. hartmanni*; Fig. 3)

We find nematodes that typically colonize the vertebrate gut including pinworms (Oxyurida; *Trypanoxyuris)*, whipworms (Enoplea; *Trichuris* sp.), and members of orders Spirurida and Rhabditida. Like other major taxa, we detect many nematode species that are not gut residents and were likely consumed or are environmental taxa acquired post deposition. For example, the nematode order Tylenchida includes species that parasitize plants [72], while members of the genus *Geocentrophora* include widespread free living nematodes from a variety of environmental sources [73, 74]. Interestingly, the nematode genus *Schistonchus*, which colonizes the fruiting bodies of fig trees [75], was detected in two black howler monkeys (*A. pigra*), which are known to dedicate a significant portion of their feeding activity to fig leaves and fruit in some ecological contexts [76].

As the eukaryotic gut signal cannot be reliably separated from dietary or environmental contamination at the community level, downstream analyses focused on known gut protist and helminth species. This study highlights the care necessary in interpreting eukaryotic gut microbiome data.

### Nematodes

**Fig. 4:**
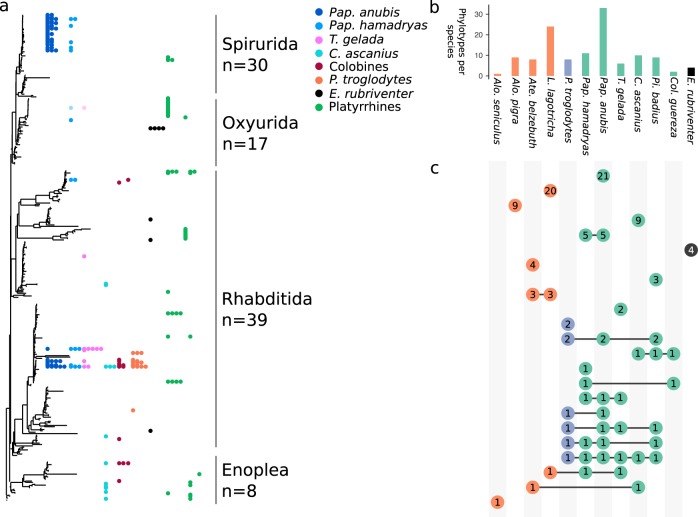
Phylotype host-specificity among gut-resident nematode groups. **a** Maximum likelihood tree of nematode phylotypes that are probable gut residents with nematode group and number of phylotypes indicated. Each dot represents a single NHP individual and color indicates NHP species group. Each row of dots indicates a single phylotype corresponding to a tip in the phylogenetic tree. Lighter colored dots indicate very low abundance in a sample, as in Fig. 3. **b** Bar chart summarizing the number of nematode phylotypes found in each NHP species colored by NHP phylogroup. **c** Upset plot illustrating host-specificity of nematode phylotypes. Unconnected dots are phylotypes found in only one NHP species. Connected dots indicate phylotypes shared by two or more NHP species. Numbers within dots indicate the number of phylotypes

We find gut-associated nematode phylotypes that differ in distribution from host-specific to cosmopolitan (Fig. 4). For example, *Physaloptera* sp. (order Spirurida) is exclusively found in the brown woolly monkeys, *L. lagotricha*, while other Spirurida are found in both species of baboon (*Pap. anubis* and *Pap. hamadryas*) (Fig. 4a). Other nematode phylotypes have a more cosmopolitan distribution across many host species. For example, a group of similar phylotypes within the nematode order Rhabditida is present among multiple individuals in the species *P. troglodytes, C. ascanius, Pap. anubis, Pap. hamadryas, T. gelada*, and *Pi. badius* (Fig. 4a), which represent a diverse suite of diets, environments, and geographic distribution.

### Protists

*Entamoeba* is the dominant eukaryotic gut resident detected in the current study. While *Entamoeba* is best known as a pathogen (*Ent. histolytica* is the causative agent of amoebic dysentery), many species of *Entamoeba* are commensal. We find multiple nonpathogenic lineages (Fig. 3a). *Entamoeba* are found in all NHP phylogroups with the highest frequency among cercopithecoids and especially *Pap. anubis, Pap. hamadryas, Pi. badius*, and *C. ascanius* (Fig. 3b). The colobine monkeys harbor phylotypes that fall within the *Ent. bovis* clade, but are distinct from RL3 previously detected in langurs [59]. This observation further supports an association between the *Ent. bovis* clade and folivorous primates, but expands known diversity. We also detected two novel *Entamoeba* phylotypes in *Alouatta pigra*, including one previously found in *A. pigra* [77]. *Entamoeba* phylotype diversity in the platyrrhine monkeys and apes is lower than in cercopithecines. We don’t find any phylotypes unique to lemurs and all phylotypes detected in lemurs are at low abundance (indicated by light colored dots in Fig. 3a). This may reflect an artifact of sequencing, such as barcode switching, rather than a true signal of shared phylotypes. Overall, the highest degree of phylotype sharing is between *Papio* and *Cercopithecus* (Fig. 3c). While phylotypes of *Ent. coli* and *Ent. dispar* are largely shared across multiple species, phylotypes of *Ent. hartmanni* and *Ent. polecki* have a higher degree of host specificity. This is especially apparent among phylotypes assigned to *Ent. hartmanni* and *Ent. polecki*: phylotypes detected in *T. gelada* (pink) are not found in any other NHP (Fig. 3a).

**Fig. 5:**
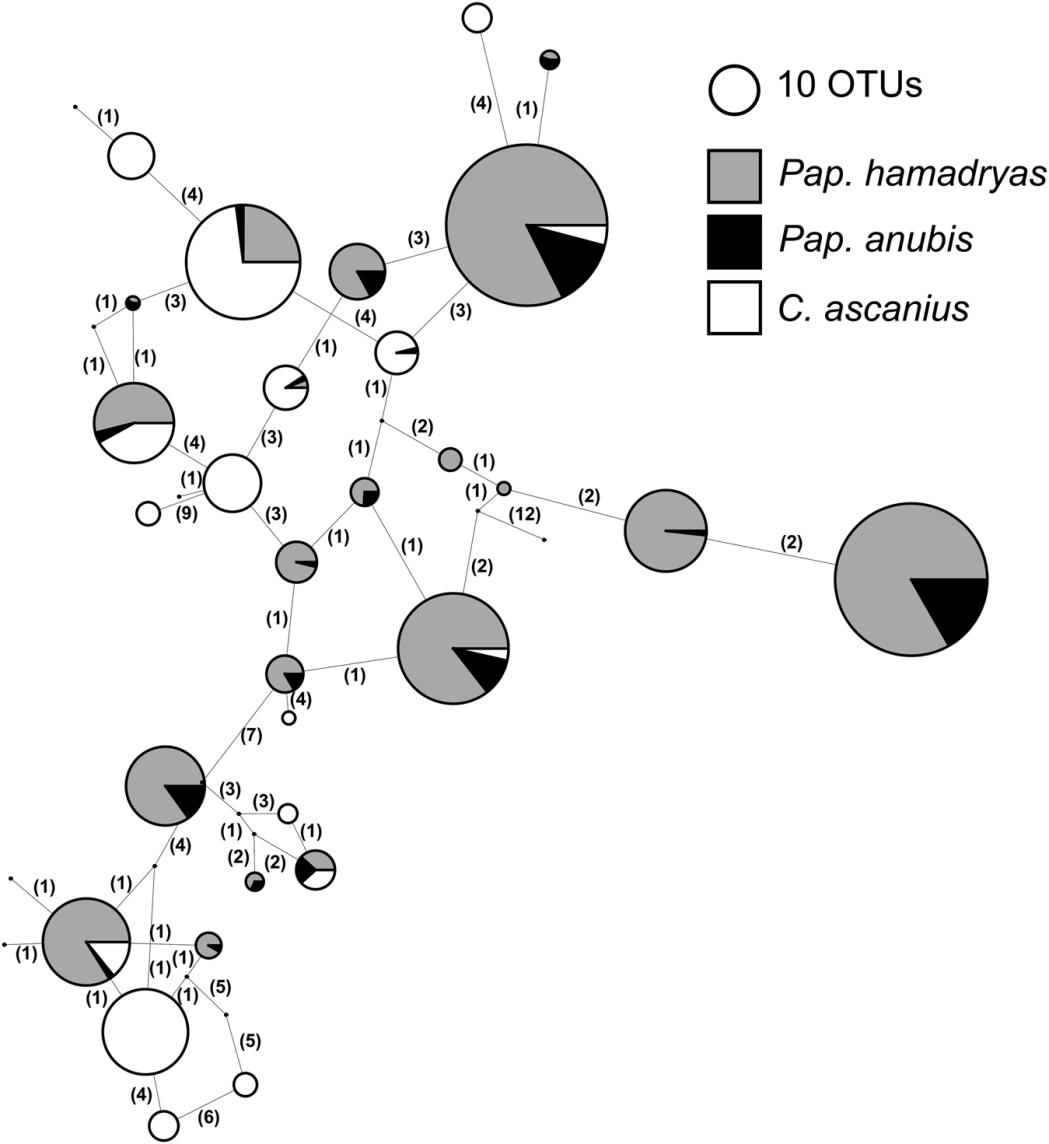
High diversity of *Iodamoeba* sp. RL1 found in *Pap. hamadryas, Pap. anubis*, and *C. ascanius*. Statistical parsimony network of *Iodamoeba* phylotypes found in the current dataset. Size of node corresponds to the number of OTUs that are collapsed into the node after trimming positions in the alignment where there is a gap in at least 10% of all sequences. Number listed on branches indicates the number of stepwise mutations between nodes

We detect a high diversity of *Iodamoeba* ribosomal lineage one (RL1) [66] phylotypes in *Pap. anubis, Pap. hamadryas*, and *C. ascanius*, many of which are shared (Fig. 5). We also found several very short reads assigned weakly to *Iodamoeba* from chimpanzees, but these were excluded from the network analysis because of low confidence. *Iodamoeba* is rarely studied and these results highlight unexpected diversity of *Iodamoeba*, and the need for further sampling across host species. We also find multiple phylotypes of *Endolimax nana*, another gut resident related to *Entamoeba*, in two *C. ascanius* individuals.

## Discussion

### Community level analysis of eukaryotic data is confounded by mixed nature of eukaryotic assemblage

Targeted eukaryotic amplicon surveys yield a diverse suite of taxa that originate from dietary and environmental sources and are passing through the gut, as well as those that are true residents of the mammalian gut. Thus, eukaryotes detected in these surveys cannot be simply assumed to be gut residents as is commonly done for the mammalian gut bacterial community. The proportion of reads assigned to known gut-associated eukaryotes among NHPs in this study is highly variable within and across species groups with the highest proportion in the cercopithecines (45–99%) and the lowest among the lemurs (0–46%) (Fig. 1). Given the mixed nature of these data, isolating the true signal of the gut eukaryome is challenging. As such, community level analyses (e.g., beta diversity, comparisons of relative abundances across samples) are not fair representations of the data. Instead, we argue here that a focus on known gut taxa (e.g., *Entamoeba, Iodamoeba, Blastocystis*, and nematodes) that are common across NHPs can provide insights into the evolution of the gut eukaryome.

### Distribution of gut-associated eukaryotes within and across NHPs is likely driven by host behavior and ecology

While not explicitly tested in the current study, the diversity of gut eukaryotes across NHP hosts appears to be driven by individual dynamics and host behavior and not phylosymbiosis or codiversification. For example, the Verreaux’s sifakas (*P. verreauxi*) in this study have very few reads assigned to gut-associated eukaryotes despite having high initial read counts (Fig. 1). Members of this species spend nearly all of their time in trees and their water is obtained from their food, limiting their contact with eukaryotic organisms that are transmitted through fecal contaminated soil or water [78, 79]. This observation is in extreme contrast to the baboons where obtained reads are dominated by eukaryotes known to reside in the gut. Baboons are large terrestrial monkeys and as such are in direct contact with potentially contaminated soil and water sources. Moreover, baboons have diverse and highly flexible omnivorous diets and are known to provision themselves on human crops and refuse [80], giving them ample opportunity to acquire diverse microorganisms. A previous study of the gut microbiome in baboons demonstrated that the local environment plays a substantial role in shaping the bacterial community [81], something that likely has an important role in the eukaryotic assemblage as well. In addition to those eukaryotic organisms that are natural residents of the NHP gut, organisms that likely originate from the environment provide insights into behavior or environmental context. For instance, gelada monkeys rely heavily on grass in their diets [82] but they may consume high numbers of insects under certain circumstances [83]. We find many reads assigned to Gregarinasina, a clade of apicomplexan insect symbionts which are not members of the vertebrate gut community, across all five gelada monkeys. This may indicate that these geladas supplement their diet with insects or that the grass they eat is contaminated with gregarine spores.

In NHPs, patterns of gut-eukaryotic load are modulated by host behavior including social structure, grooming, and parasite avoidance behaviors [5, 9, 84, 85], proximity to humans and other sources of transmission [86–88], and ecological factors (e.g., wet versus dry season) [89]. Assessing the relative contribution of these factors to variation in gut-eukaryotic load across NHPs in this study is beyond the scope of the current dataset but these are important considerations for future comparative research. As samples for each primate species in were collected at the same time, results from the current study provides a partial glimpse into the diversity of gut eukaryotes in wild NHPs.

### Fungi are not natural residents of the NHP gut

Fungal OTUs detected in the current study are predominantly specific to individual NHPs with very few OTUs shared across hosts. This is in contrast to expectations of a resident suite of gut-associated fungi based on reports from human cohorts [90], though the status of human gut-associated fungi as true residents has been challenged [91, 92]. Instead, the majority of fungi detected here likely derive from food or environmental sources. For example, while *Candida* sp. are reported to be common members of the human gut [26], the highest number of reads assigned to this genus in the current dataset is to *Candida fructus*, a yeast commonly found on fruit [93]. Though the high diversity and interindividual inconsistency of fungi represented here likely reflect individual dynamics and feeding behavior, it does not preclude the possibility that some fungi may colonize the gut.

### Gut-associated eukaryotes in NHP provide insights into bacterial diversity and the consequences of microbial loss

NHPs with high eukaryotic alpha diversity also tend to have higher bacterial alpha diversity, a pattern that suggests that mechanisms that promote high diversity in one also increase diversity in the other. For example, a diverse diet or larger social group may increase transmission or exposure to more diverse bacterial and eukaryotic organisms. Bacteria detected from fecal samples, like eukaryotes, are an assemblage of microbes originating from the environment, food and other ingested material, and true gut residents. Unlike eukaryotes, however, determining the origin of bacteria in the gut is difficult without strong prior expectations. NHPs with high bacterial diversity also tend to be those dominated by Amoebozoa. Thus, an alternative hypothesis is that the presence of gut-associated protists promotes higher bacterial diversity in the gut or the increased bacterial diversity is driven by intra- or extracellular symbionts carried by protist species. In the current study, these comparisons are confounded by host phylogeny as the cercopithecines have both the highest eukaryotic and bacterial diversity, and all individuals in these species are colonized by similar eukaryotes. Testing these hypotheses will require more extensive sampling within a host species to identify populations that differ independently in their eukaryotic and bacterial community composition.

NHPs colonized by *Blastocystis* sp., Nematoda, and *Entamoeba* sp. are enriched in bacterial taxa associated with gut permeability and inflammation (i.e., *Oscillibacter*) [94], but also those that may be protective against infection (e.g., *Lachnospiraceae*) [95], among others (Supplementary Table 4). Interestingly, the spirochete *Treponema* is enriched in NHPs with gut-associated nematodes or protists. Enrichment of this bacterial genus has previously been reported in *Entamoeba* sp*.-*positive humans [19]. *Treponema* is notably absent in industrialized human populations but common in extant traditional [36, 37, 96, 97] and extinct preindustrial human groups [35]. Our documentation of co-occurrence between gut eukaryotes and *Treponema* and other bacteria across primates suggests heretofore unappreciated interactions among classes of gut microbes that may provide mechanistic explanations for the loss of functionally linked taxa in industrialized populations.

### Nematodes phylotypes are generally host-specific with a minority detected across multiple host species

Nematode phylotypes are typically host-specific or constrained to species within the same geographic region (Fig. 4c). For example, a single phylotype assigned to the Rhabditida nematode order is only found in red-bellied lemurs (*E. rubriventer*), while the whipworm genus *Trichuris* is detected in three arboreal cercopithecoids collected in Uganda (*Pi. badius, Cer. ascanius*, and *Col. guereza*). Similarly, a single phylotype within Oxyurida is found in a single brown woolly monkey (*L. lagotricha*) and is 99.24% identical to a *Trypanoxyuris atelis* (KU285460) specimen isolated from a black-handed spider monkey (*Ateles geoffroyi*). This phylotype was also detected at very low abundance in two cercopithecoids (<10 reads) as compared with the brown woolly monkey (>40,000) and is commonly isolated in wild NHPs in Central and South America, making it likely that cercopithecoid sequences are artifacts (e.g., barcode switching) and the brown woolly monkey is the true host of this parasitic nematode. The wide distribution of other nematode phylotypes, however, is more robust. For example, a phylotype assigned to Rhabditida is found in 19 individuals in the cercopithecoid group with only five individuals with fewer than 50 reads assigned. This phylotype is 100% identical to the 18S rRNA gene of *Oesophagostomum aculeatum* (AB677956) isolated from a Japanese macaque (*Macaca fuscata yakui*), demonstrating its wide range in African and Asian cercopithecoids.

### Gut-associated protists are predominantly nonpathogenic and are common across all NHP groups

Gut-associated protists including *Blastocystis* and *Entamoeba* are broadly distributed with the highest diversity in the cercopithecoids. This contrasts with greater host-specificity of nematodes. However, we see phylotype specificity within *Blastocystis* and *Entamoeba*, highlighting the great diversity encompassed within single genera for these ancient clades associated with the vertebrate gut. Interestingly, *Entamoeba* phylotype diversity in the platyrrhines, hominoids, and lemurs is largely a subset of the diversity found in the cercopithecoids, which may indicate that the association between NHPs and this protist group predates the divergence of major NHP phylogroups. Substantial overlap in *Entamoeba* phylotypes is especially prevalent in the cercopithecines, particularly *Pap. anubis, Pap. hamadryas*, *C. ascanius*, and *Pi. badius*. A high degree of phylotype sharing between these NHP species is likely driven by shared habitat (both baboon species were sampled from groups living in Ethiopia, while the red-tailed monkeys and western red colobus in this study live in the Kibale National Park in Uganda) but other phylotypes are shared across NHPs independent of geographic distance or habitat type. Despite substantial phylotype sharing among cercopithecines across *Entamoeba* at the genus level, *Entamoeba* species exhibit different levels of host-specificity. For example, while phylotypes of *Ent. coli* are shared across multiple host species, phylotypes of *Ent. hartmanni* may be more host-specific. This is especially clear among *Ent. hartmanni* phylotypes detected in the gelada monkeys (*T. gelada*), which are distinct from those found in other NHPs. Differential host specificity of *Entamoeba* phylotypes in the current study mirror results of previous studies of wild hominoids and humans in neighboring environments [87]. Unlike *Entamoeba*, which was found across all NHP phylogroups, *Iodamoeba* is primarily found in only three species of the cercopithecine group with a high degree of phylotype sharing that cannot be explained solely by a shared habitat. Instead, there appears to be a gradient of phylotype sharing across the baboons and red-tailed monkeys. The results of this study illustrate that the diversity of *Iodamoeba* is yet to be fully appreciated in wild NHPs. Finally, we detect *Blastocystis* across NHP hosts. We find ST1, ST2, and ST3 in baboons, with geladas and baboons sharing some phylotypes of ST1 and ST3. *Cercopithecus ascanius* harbor distinct ST3 phylotypes. *Alouatta pigra* harbors ST8 and we find ST11 in one chimpanzee (Supplementary Fig. 5, Supplementary Table 5).

This study represents the largest to-date 18S rRNA amplicon survey of the gut microbiome in wild NHPs. Unlike the bacterial community [2], we find that the impact of host phylogeny on gut-associated eukaryotes is weak. We do not find evidence of phylosymbiosis and instead it is likely that individual dynamics, local ecology, and behavioral aspects shape the total eukaryotic assemblage, though the current dataset cannot verify the mechanisms that shape these patterns. Future studies should examine the impact of behavior and local ecology on the gut eukaryotic assemblage. Moreover, specific gut-associated eukaryotes document varying patterns of host specificity and geographic distribution. We suggest that further studies examining the major classes of microbes that constitute the complex community of taxa in the primate gut, including their interactions, will shed additional light on the structure, function, and evolution of the gastrointestinal microbiome.

## Supplementary information

Supplemental methods and figures

Supplementary tables
