# Supplementary material for: Biodiversity of protists and nematodes in the wild nonhuman primate gut
Source: ISME J. 2019 Nov 12;14(2):609–22. doi: 10.1038/s41396-019-0551-4 (PMC6976604; doi:10.1038/s41396-019-0551-4)
Supplement: Supplementary file 1 — Supplemental methods and figures [file 41396_2019_551_MOESM1_ESM.docx]

SUPPLEMENTARY INFORMATION

Biogeography of protists and nematodes in the wild non-human primate gut microbiome

Allison E. Mann, Florent Mazel, Matt Lemay, Evan Morien, Vincent Billy, Martin Kowalewski, Anthony Di Fiore, Andres Link, Tony L. Goldberg, Stacey Tecot, Andrea Baden, Andres Gomez, Michelle L. Sauther, Frank Cuozzo, Gillian A.O. Britton, Nathaniel J. Dominy, Rebecca Stumpf, Rebecca J. Lewis, Larissa Swedell, Katherine Amato, Laura Wegener Parfrey

**Supplementary Methods**

Fecal samples from wild NHPs were collected as soon as possible after defecation using a sterile tool and stored in either a 95% ETOH solution (*L. catta, G. gorilla, A. pigra)* or RNAlater (all other samples) in a sterile collection tube. Samples were collected in the field and transported to the United States by collaborators. NHP species associated collaborator and preservation method can be found in the table below. Fecal samples were prepared following the Earth Microbiome Project protocol (Thompson, Sanders et al. 2017). DNA was extracted using a MO BIO PowerSoil extraction kit. All samples were collected with formal approval and appropriate permits from host countries and author’s institutions.

**Supplementary Figures**

Figure 1: Gut eukaryote assemblages are composed of a mixture of host-associated and environmental organisms. Environmental and dietary organisms are found in all major eukaryotic groups detected in this study after removing plants, vertebrates, and insects. Cercopithecoids, and particularly those belonging to the cercopithecine group, have the strongest and most consistent gut signal of all primate groups. This is in stark contrast to members of *P. verreauxi* and, to a lesser extent, *A. belzebuth* of which very little gut signal is detected.

Figure 2: No evidence for phylsymbosis in the primate gut eukaryome. This figure presents (A) the raw plot between pairwise beta-diversity (y-axis, weighted Unifrac) and host phylogenetic distance (x-axis, millions of years) and (B) the distribution of resulting mantel statistics. The panel (A) encompasses all possible pairs of samples in the dataset (including intra-specific comparisons, that have null value on the X-axis) while results presented in panel (B) are restricted to sub samples of the dataset with only one randomly chosen individual per species. The variability across the 100 random sets is presented as a box plot. All 100 mantel tests yielded non-significant results.


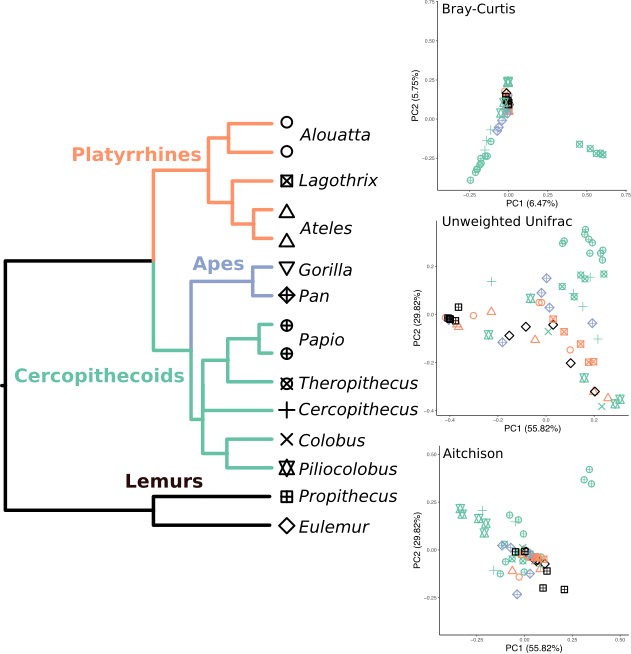


Figure 3: Alternative distance matrix ordination plots. While a PCoA plot of a Bray Curtis dissimilarity matrix is similar to that generated by weighted UniFrac, both unweighted UniFrac and Aitchison distance ordinations do not support major differences by host phylogroup.

Figure 4: Highest eukaryotic alpha diversity found in the *Papio* and *Cercopithecus* individuals. Eukaryotic diversity (number of observed OTUs) is variable across host species.

Figure 5: *Blastocystis* placement tree. *Blastocystis* subtype sequences isolated from previously published NHP individuals collected from GenBank as well as reference sequences of *Blastocystis* in the SILVA and PR2 databases were collected to create a reference database (see Supplemental Table 5 for a list of accessions, hosts, and original study). Sequences from the current study were aligned to the reference tree as a backbone constraint. Bootstrap values over 90 are shown. Sequences from the current study are annotated with *

**Supplementary References**

Thompson, L. R., J. G. Sanders, D. McDonald, A. Amir, J. Ladau, K. J. Locey, R. J. Prill, A. Tripathi, S. M. Gibbons, G. Ackermann, J. A. Navas-Molina, S. Janssen, E. Kopylova, Y. Vázquez-Baeza, A. González, J. T. Morton, S. Mirarab, Z. Zech Xu, L. Jiang, M. F. Haroon, J. Kanbar, Q. Zhu, S. Jin Song, T. Kosciolek, N. A. Bokulich, J. Lefler, C. J. Brislawn, G. Humphrey, S. M. Owens, J. Hampton-Marcell, D. Berg-Lyons, V. McKenzie, N. Fierer, J. A. Fuhrman, A. Clauset, R. L. Stevens, A. Shade, K. S. Pollard, K. D. Goodwin, J. K. Jansson, J. A. Gilbert, R. Knight, C. The Earth Microbiome Project, J. L. A. Rivera, L. Al-Moosawi, J. Alverdy, K. R. Amato, J. Andras, L. T. Angenent, D. A. Antonopoulos, A. Apprill, D. Armitage, K. Ballantine, J. Bárta, J. K. Baum, A. Berry, A. Bhatnagar, M. Bhatnagar, J. F. Biddle, L. Bittner, B. Boldgiv, E. Bottos, D. M. Boyer, J. Braun, W. Brazelton, F. Q. Brearley, A. H. Campbell, J. G. Caporaso, C. Cardona, J. Carroll, S. C. Cary, B. B. Casper, T. C. Charles, H. Chu, D. C. Claar, R. G. Clark, J. B. Clayton, J. C. Clemente, A. Cochran, M. L. Coleman, G. Collins, R. R. Colwell, M. Contreras, B. B. Crary, S. Creer, D. A. Cristol, B. C. Crump, D. Cui, S. E. Daly, L. Davalos, R. D. Dawson, J. Defazio, F. Delsuc, H. M. Dionisi, M. G. Dominguez-Bello, R. Dowell, E. A. Dubinsky, P. O. Dunn, D. Ercolini, R. E. Espinoza, V. Ezenwa, N. Fenner, H. S. Findlay, I. D. Fleming, V. Fogliano, A. Forsman, C. Freeman, E. S. Friedman, G. Galindo, L. Garcia, M. A. Garcia-Amado, D. Garshelis, R. B. Gasser, G. Gerdts, M. K. Gibson, I. Gifford, R. T. Gill, T. Giray, A. Gittel, P. Golyshin, D. Gong, H.-P. Grossart, K. Guyton, S.-J. Haig, V. Hale, R. S. Hall, S. J. Hallam, K. M. Handley, N. A. Hasan, S. R. Haydon, J. E. Hickman, G. Hidalgo, K. S. Hofmockel, J. Hooker, S. Hulth, J. Hultman, E. Hyde, J. D. Ibáñez-Álamo, J. D. Jastrow, A. R. Jex, L. S. Johnson, E. R. Johnston, S. Joseph, S. D. Jurburg, D. Jurelevicius, A. Karlsson, R. Karlsson, S. Kauppinen, C. T. E. Kellogg, S. J. Kennedy, L. J. Kerkhof, G. M. King, G. W. Kling, A. V. Koehler, M. Krezalek, J. Kueneman, R. Lamendella, E. M. Landon, K. Lane-deGraaf, J. LaRoche, P. Larsen, B. Laverock, S. Lax, M. Lentino, I. I. Levin, P. Liancourt, W. Liang, A. M. Linz, D. A. Lipson, Y. Liu, M. E. Lladser, M. Lozada, C. M. Spirito, W. P. MacCormack, A. MacRae-Crerar, M. Magris, A. M. Martín-Platero, M. Martín-Vivaldi, L. M. Martínez, M. Martínez-Bueno, E. M. Marzinelli, O. U. Mason, G. D. Mayer, J. M. McDevitt-Irwin, J. E. McDonald, K. L. McGuire, K. D. McMahon, R. McMinds, M. Medina, J. R. Mendelson, J. L. Metcalf, F. Meyer, F. Michelangeli, K. Miller, D. A. Mills, J. Minich, S. Mocali, L. Moitinho-Silva, A. Moore, R. M. Morgan-Kiss, P. Munroe, D. Myrold, J. D. Neufeld, Y. Ni, G. W. Nicol, S. Nielsen, J. I. Nissimov, K. Niu, M. J. Nolan, K. Noyce, S. L. O’Brien, N. Okamoto, L. Orlando, Y. O. Castellano, O. Osuolale, W. Oswald, J. Parnell, J. M. Peralta-Sánchez, P. Petraitis, C. Pfister, E. Pilon-Smits, P. Piombino, S. B. Pointing, F. J. Pollock, C. Potter, B. Prithiviraj, C. Quince, A. Rani, R. Ranjan, S. Rao, A. P. Rees, M. Richardson, U. Riebesell, C. Robinson, K. J. Rockne, S. M. Rodriguezl, F. Rohwer, W. Roundstone, R. J. Safran, N. Sangwan, V. Sanz, M. Schrenk, M. D. Schrenzel, N. M. Scott, R. L. Seger, A. Seguin-Orlando, L. Seldin, L. M. Seyler, B. Shakhsheer, G. M. Sheets, C. Shen, Y. Shi, H. Shin, B. D. Shogan, D. Shutler, J. Siegel, S. Simmons, S. Sjöling, D. P. Smith, J. J. Soler, M. Sperling, P. D. Steinberg, B. Stephens, M. A. Stevens, S. Taghavi, V. Tai, K. Tait, C. L. Tan, N. Tas¸, D. L. Taylor, T. Thomas, I. Timling, B. L. Turner, T. Urich, L. K. Ursell, D. van der Lelie, W. Van Treuren, L. van Zwieten, D. Vargas-Robles, R. V. Thurber, P. Vitaglione, D. A. Walker, W. A. Walters, S. Wang, T. Wang, T. Weaver, N. S. Webster, B. Wehrle, P. Weisenhorn, S. Weiss, J. J. Werner, K. West, A. Whitehead, S. R. Whitehead, L. A. Whittingham, E. Willerslev, A. E. Williams, S. A. Wood, D. C. Woodhams, Y. Yang, J. Zaneveld, I. Zarraonaindia, Q. Zhang and H. Zhao (2017). "A communal catalogue reveals Earth’s multiscale microbial diversity." Nature **551**: 457.
