# Supplementary material for: Biodiversity of protists and nematodes in the wild nonhuman primate gut
Source: ISME J. 2019 Nov 12;14(2):609–22. doi: 10.1038/s41396-019-0551-4 (PMC6976604; doi:10.1038/s41396-019-0551-4)
Supplement: Supplementary file 2 — Supplementary tables [file 41396_2019_551_MOESM2_ESM.pdf]

Table 1: Supplementary Table 1: Sample metadata

| **ID** | **Genus** | **Species** | **Location** | **Diet** | **Country** | **Habitat** | **Sample preservation** | **Collaborators** |
|---|---|---|---|---|---|---|---|---|
| APF37 | *Alouatta* | *pigra* | Palenque National Park | Folivore | Mexico | Rainforest | 95% Ethanol | Martin Kowalewski |
| APF38 | *Alouatta* | *pigra* | Palenque National Park | Folivore | Mexico | Rainforest | 95% Ethanol | Martin Kowalewski |
| APF39 | *Alouatta* | *pigra* | Palenque National Park | Folivore | Mexico | Rainforest | 95% Ethanol | Martin Kowalewski |
| AsenLink1 | *Alouatta* | *seniculus* | Yasuni Biosphere Reserve | Folivore | Ecuador | Rainforest | RNAlater | Anthony Di Fiore, Andres Link |
| AsenLink2 | *Alouatta* | *seniculus* | Yasuni Biosphere Reserve | Folivore | Ecuador | Rainforest | RNAlater | Anthony Di Fiore, Andres Link |
| AsenLink3 | *Alouatta* | *seniculus* | Yasuni Biosphere Reserve | Folivore | Ecuador | Rainforest | RNAlater | Anthony Di Fiore, Andres Link |
| AtbelzLink1 | *Ateles* | *belzebuth* | Yasuni Biosphere Reserve | Not | Ecuador | Rainforest | RNAlater | Anthony Di Fiore |

Table 1: Supplementary Table 1: Sample metadata

| ID | Genus | Species | Location | Diet | Country | Habitat | Sample preservation | Collaborators |
|---|---|---|---|---|---|---|---|---|
| AtbelzLink2 | *Ateles* | *belzebuth* | Yasuni Biosphere Reserve | Not | Ecuador | Rainforest | RNAlater | Anthony Di Fiore |
| AtbelzLink4 | *Ateles* | *belzebuth* | Yasuni Biosphere Reserve | Not | Ecuador | Rainforest | RNAlater | Anthony Di Fiore |
| AtbelzLink5 | *Ateles* | *belzebuth* | Yasuni Biosphere Reserve | Not | Ecuador | Rainforest | RNAlater | Anthony Di Fiore |
| Ahyb1000 | *Ateles* | *hybridus* | San Juan de Carare | Not | Colombia | Rainforest | RNAlater | Andres Link |
| Ahyb135 | *Ateles* | *hybridus* | San Juan de Carare | Not | Colombia | Rainforest | RNAlater | Andres Link |
| RT2013 | *Cercopithecus* | *ascanius* | Kibale National Park | Not | Uganda | Rainforest | RNAlater | Tony Goldberg |
| RT2017 | *Cercopithecus* | *ascanius* | Kibale National Park | Not | Uganda | Rainforest | RNAlater | Tony Goldberg |
| RT2019 | *Cercopithecus* | *ascanius* | Kibale National Park | Not | Uganda | Rainforest | RNAlater | Tony Goldberg |
| RT2021 | *Cercopithecus* | *ascanius* | Kibale National Park | Not | Uganda | Rainforest | RNAlater | Tony Goldberg |
| RT2048 | *Cercopithecus* | *ascanius* | Kibale National Park | Not | Uganda | Rainforest | RNAlater | Tony Goldberg |
| BWC2063 | *Colobus* | *guereza* | Kibale National Park | Folivore | Uganda | Rainforest | RNAlater | Tony Goldberg |
| BWC2115 | *Colobus* | *guereza* | Kibale National Park | Folivore | Uganda | Rainforest | RNAlater | Tony Goldberg |
| ERUB06M | *Eulemur* | *rubriventer* | Ranomafana National Park | Not | Madagascar | Rainforest | RNAlater | Stacey Tecot, Andrea Baden |

Table 1: Supplementary Table 1: Sample metadata

| **ID** | **Genus** | **Species** | **Location** | **Diet** | **Country** | **Habitat** | **Sample preservation** | **Collaborators** |
|---|---|---|---|---|---|---|---|---|
| ERUB101M | *Eulemur* | *rubriventer* | Ranomafana National Park | Not | Madagascar | Rainforest | RNAlater | Stacey Tecot, Andrea Baden |
| ERUB102M | *Eulemur* | *rubriventer* | Ranomafana National Park | Not | Madagascar | Rainforest | RNAlater | Stacey Tecot, Andrea Baden |
| ERUB104M | *Eulemur* | *rubriventer* | Ranomafana National Park | Not | Madagascar | Rainforest | RNAlater | Stacey Tecot, Andrea Baden |
| ERUB105M | *Eulemur* | *rubriventer* | Ranomafana National Park | Not | Madagascar | Rainforest | RNAlater | Stacey Tecot, Andrea Baden |
| Gor11 | *Gorilla* | *gorilla* | Dzanga Sangha | Folivore | CAR | Rainforest | 95% Ethanol | Andres Gomez |
| Gor12 | *Gorilla* | *gorilla* | Dzanga Sangha | Folivore | CAR | Rainforest | 95% Ethanol | Andres Gomez |
| LabEllis3 | *Lagothrix* | *lagotricha* | Yasuni Biosphere Reserve | Not | Ecuador | Rainforest | RNAlater | Anthony Di Fiore |
| LagAb2 | *Lagothrix* | *lagotricha* | Yasuni Biosphere Reserve | Not | Ecuador | Rainforest | RNAlater | Anthony Di Fiore |
| LagEllis1 | *Lagothrix* | *lagotricha* | Yasuni Biosphere Reserve | Not | Ecuador | Rainforest | RNAlater | Anthony Di Fiore |

Table 1: Supplementary Table 1: Sample metadata

| ID | Genus | Species | Location | Diet | Country | Habitat | Sample preservation | Collaborators |
|---|---|---|---|---|---|---|---|---|
| LagEllis4 | *Lagothrix* | *lagotricha* | Yasuni Biosphere Reserve | Not | Ecuador | Rainforest | RNAlater | Anthony Di Fiore |
| LagEllis5 | *Lagothrix* | *lagotricha* | Yasuni Biosphere Reserve | Not | Ecuador | Rainforest | RNAlater | Anthony Di Fiore |
| Lc310 | *Lemur* | *catta* | Beza Mahafaly Reserve | Not | Madagascar | Dry Forest | 95% Ethanol | Michelle Sauther, Frank Cuozzo |
| Ptr1 | *Pan* | *troglodytes* | Kibale National Park | Not | Uganda | Rainforest | RNAlater | Rebecca Stumpf |
| Ptr10 | *Pan* | *troglodytes* | Kibale National Park | Not | Uganda | Rainforest | RNAlater | Rebecca Stumpf |
| Ptr11 | *Pan* | *troglodytes* | Kibale National Park | Not | Uganda | Rainforest | RNAlater | Rebecca Stumpf |
| Ptr13 | *Pan* | *troglodytes* | Kibale National Park | Not | Uganda | Rainforest | RNAlater | Rebecca Stumpf |
| PTr14 | *Pan* | *troglodytes* | Kibale National Park | Not | Uganda | Rainforest | RNAlater | Rebecca Stumpf |
| JB10 | *Papio* | *anubis* | Awash National Park | Not | Ethiopia | Savannah | RNAlater | Larissa Swedell, Jill Rice, Nathaniel Dominy |

Table 1: Supplementary Table 1: Sample metadata

| ID | Genus | Species | Location | Diet | Country | Habitat | Sample preservation | Collaborators |
|---|---|---|---|---|---|---|---|---|
| JB12 | *Papio* | *anubis* | Awash National Park | Not | Ethiopia | Savannah | RNAlater | Larissa Swedell, Jill Rice, Nathaniel Dominy |
| JB24 | *Papio* | *anubis* | Awash National Park | Not | Ethiopia | Savannah | RNAlater | Larissa Swedell, Jill Rice, Nathaniel Dominy |
| JB25 | *Papio* | *anubis* | Awash National Park | Not | Ethiopia | Savannah | RNAlater | Larissa Swedell, Jill Rice, Nathaniel Dominy |
| JB28 | *Papio* | *anubis* | Awash National Park | Not | Ethiopia | Savannah | RNAlater | Larissa Swedell, Jill Rice, Nathaniel Dominy |

Table 1: Supplementary Table 1: Sample metadata

| **ID** | **Genus** | **Species** | **Location** | **Diet** | **Country** | **Habitat** | **Sample preservation** | **Collaborators** |
|---|---|---|---|---|---|---|---|---|
| JB1 | *Papio* | *hamadryas* | Awash National Park | Not | Ethiopia | Savannah | RNAlater | Larissa Swedell, Jill Rice, Nathaniel Dominy |
| JB13 | *Papio* | *hamadryas* | Awash National Park | Not | Ethiopia | Savannah | RNAlater | Larissa Swedell, Jill Rice, Nathaniel Dominy |
| JB15 | *Papio* | *hamadryas* | Awash National Park | Not | Ethiopia | Savannah | RNAlater | Larissa Swedell, Jill Rice, Nathaniel Dominy |
| JB17 | *Papio* | *hamadryas* | Awash National Park | Not | Ethiopia | Savannah | RNAlater | Larissa Swedell, Jill Rice, Nathaniel Dominy |

Table 1: Supplementary Table 1: Sample metadata

| ID | Genus | Species | Location | Diet | Country | Habitat | Sample preservation | Collaborators |
|---|---|---|---|---|---|---|---|---|
| JB18 | *Papio* | *hamadryas* | Awash National Park | Not | Ethiopia | Savannah | RNAlater | Larissa Swedell, Jill Rice, Nathaniel Dominy |
| RC2036 | *Piliocolobus* | *badius* | Kibale National Park | Folivore | Uganda | Rainforest | RNAlater | Tony Goldberg |
| RC2068 | *Piliocolobus* | *badius* | Kibale National Park | Folivore | Uganda | Rainforest | RNAlater | Tony Goldberg |
| RC2071 | *Piliocolobus* | *badius* | Kibale National Park | Folivore | Uganda | Rainforest | RNAlater | Tony Goldberg |
| RC2096 | *Piliocolobus* | *badius* | Kibale National Park | Folivore | Uganda | Rainforest | RNAlater | Tony Goldberg |
| RC2098 | *Piliocolobus* | *badius* | Kibale National Park | Folivore | Uganda | Rainforest | RNAlater | Tony Goldberg |
| PVAbb | *Propithecus* | *verreauxi* | Kirindy Mitea | Folivore | Madagascar | tropical dry forest | RNAlater | Rebecca Lewis |
| PVLou | *Propithecus* | *verreauxi* | Kirindy Mitea | Folivore | Madagascar | tropical dry forest | RNAlater | Rebecca Lewis |
| PVPam | *Propithecus* | *verreauxi* | Kirindy Mitea | Folivore | Madagascar | tropical dry forest | RNAlater | Rebecca Lewis |
| PVPet | *Propithecus* | *verreauxi* | Kirindy Mitea | Folivore | Madagascar | tropical dry forest | RNAlater | Rebecca Lewis |
| PVQui | *Propithecus* | *verreauxi* | Kirindy Mitea | Folivore | Madagascar | tropical dry forest | RNAlater | Rebecca Lewis |
| JB27 | *Theropithecus* | *gelada* | Guassa | Folivore | Ethiopia | Savannah | RNAlater | Peter Fashing, Nga Nguyen, Jill Britton, Nathaniel Dominy |

Table 1: Supplementary Table 1: Sample metadata

| ID | Genus | Species | Location | Diet | Country | Habitat | Sample preservation | Collaborators |
|---|---|---|---|---|---|---|---|---|
| JB29 | *Theropithecus* | *gelada* | Guassa | Folivore | Ethiopia | Savannah | RNAlater | Peter Fashing, Nga Nguyen, Jill Britton, Nathaniel Dominy |
| JB3 | *Theropithecus* | *gelada* | Guassa | Folivore | Ethiopia | Savannah | RNAlater | Peter Fashing, Nga Nguyen, Jill Britton, Nathaniel Dominy |
| JB32 | *Theropithecus* | *gelada* | Guassa | Folivore | Ethiopia | Savannah | RNAlater | Peter Fashing, Nga Nguyen, Jill Britton, Nathaniel Dominy |

Table 1: Supplementary Table 1: Sample metadata

| ID | Genus | Species | Location | Diet | Country | Habitat | Sample preservation | Collaborators |
|---|---|---|---|---|---|---|---|---|
| JB34 | *Theropithecus* | *gelada* | Guassa | Folivore | Ethiopia | Savannah | RNAlater | Peter Fashing, Nga Nguyen, Jill Britton, Nathaniel Dominy |

Supplementary Table 2: Read Statistics

| ID | Raw | QualityFiltered | ReadsInSwarms | PostDietaryFilter | GutResidentReads | EnvironmentalReads |
|---|---|---|---|---|---|---|
| RC2098 | 72422 | 72112 | 56839 | 50814 | 49941 | 873 |
| BWC2115 | 67054 | 66817 | 53833 | 49025 | 48877 | 148 |
| LagEllis1 | 73401 | 73010 | 55732 | 48211 | 48076 | 135 |
| RC2096 | 60587 | 60254 | 48198 | 43066 | 42536 | 530 |
| RC2071 | 49062 | 48688 | 38508 | 31542 | 29791 | 1751 |
| JB17 | 46714 | 46292 | 29900 | 20686 | 20600 | 86 |
| JB32 | 39657 | 39253 | 27866 | 22946 | 19753 | 3193 |
| JB1 | 43972 | 30701 | 23566 | 15139 | 14911 | 228 |
| JB10 | 46574 | 45542 | 28282 | 15077 | 13590 | 1487 |
| JB29 | 49898 | 49653 | 31689 | 16395 | 12351 | 4044 |
| JB18 | 26891 | 20624 | 15284 | 12303 | 12272 | 31 |
| JB24 | 39033 | 37413 | 26381 | 12737 | 11813 | 924 |
| RT2021 | 34628 | 25786 | 19339 | 11288 | 11023 | 265 |
| JB13 | 38624 | 29483 | 20234 | 10418 | 10273 | 145 |
| RT2048 | 50940 | 47455 | 32623 | 11452 | 10120 | 1332 |
| JB25 | 44450 | 43181 | 31618 | 6832 | 6459 | 373 |
| RT2013 | 20188 | 19916 | 15101 | 9142 | 6143 | 2999 |
| JB34 | 40838 | 40345 | 26043 | 14506 | 5788 | 8718 |
| JB12 | 35303 | 34759 | 22898 | 6034 | 5626 | 408 |
| JB15 | 35849 | 35178 | 23342 | 5720 | 5399 | 321 |
| RT2019 | 21930 | 21595 | 12387 | 6423 | 4994 | 1429 |
| JB27 | 64291 | 63952 | 43160 | 9923 | 4940 | 4983 |
| JB28 | 19711 | 17828 | 10440 | 5341 | 4902 | 439 |
| RC2036 | 67139 | 66709 | 48645 | 21506 | 3355 | 18151 |
| Ptr11 | 68944 | 67882 | 39186 | 9069 | 3272 | 5797 |
| JB3 | 21556 | 21370 | 15204 | 4492 | 2054 | 2438 |
| PTr14 | 90176 | 89517 | 65515 | 2842 | 1768 | 1074 |

| | | | | | | |
|---|---|---|---|---|---|---|
| Ptr1 | 47530 | 47058 | 20385 | 7420 | 1668 | 5752 |
| Ptr10 | 72463 | 71773 | 46289 | 1722 | 1643 | 79 |
| AtbelzLink4 | 19327 | 19095 | 14897 | 7422 | 1167 | 6255 |
| RC2068 | 22093 | 21886 | 15373 | 8430 | 823 | 7607 |
| APF39 | 11095 | 10976 | 8202 | 3916 | 777 | 3139 |
| LagEllis4 | 15455 | 15308 | 11548 | 4829 | 758 | 4071 |
| APF37 | 23609 | 23239 | 16755 | 2177 | 700 | 1477 |
| ERUB105M | 17876 | 17733 | 13099 | 3398 | 453 | 2945 |
| ERUB104M | 43099 | 42842 | 30348 | 1728 | 427 | 1301 |
| LagAb2 | 6803 | 6605 | 3580 | 711 | 275 | 436 |
| APF38 | 73686 | 73108 | 52868 | 633 | 270 | 363 |
| ERUB06M | 6709 | 6623 | 4904 | 1278 | 234 | 1044 |
| BWC2063 | 9539 | 9438 | 7949 | 4484 | 123 | 4361 |
| LagEllis5 | 18137 | 17958 | 13288 | 8971 | 80 | 8891 |
| RT2017 | 725 | 674 | 430 | 114 | 59 | 55 |
| AtbelzLink2 | 5391 | 5298 | 4505 | 2675 | 35 | 2640 |
| LabEllis3 | 15578 | 15399 | 11684 | 3444 | 34 | 3410 |
| ERUB101M | 1099 | 1069 | 797 | 69 | 32 | 37 |
| AtbelzLink1 | 2361 | 2302 | 1877 | 667 | 31 | 636 |
| AsenLink3 | 252 | 210 | 182 | 41 | 30 | 11 |
| AsenLink1 | 459 | 359 | 242 | 18 | 12 | 6 |
| Ahyb1000 | 831 | 779 | 532 | 176 | 7 | 169 |
| AsenLink2 | 2582 | 2545 | 2105 | 914 | 5 | 909 |
| PVQui | 4482 | 4349 | 2821 | 348 | 4 | 344 |
| PVPet | 52591 | 51867 | 36814 | 25235 | 3 | 25232 |
| AtbelzLink5 | 534 | 473 | 337 | 20 | 2 | 18 |
| Gor12 | 166 | 115 | 79 | 8 | 2 | 6 |
| PVPam | 53548 | 53044 | 29907 | 13196 | 2 | 13194 |
| Ahyb135 | 1537 | 1454 | 1083 | 117 | 0 | 117 |

| | | | | | | |
|---|---|---|---|---|---|---|
| ERUB102M | 1038 | 1011 | 901 | 468 | 0 | 468 |
| Gor11 | 515 | 461 | 353 | 49 | 0 | 49 |
| Lc310 | 101 | 63 | 60 | 0 | 0 | 0 |
| Ptr13 | 3137 | 3060 | 2214 | 233 | 0 | 233 |
| PVAbb | 20082 | 19799 | 9247 | 2468 | 0 | 2468 |
| PVLou | 50837 | 50117 | 35933 | 28654 | 0 | 28654 |

Supplementary Table 3: Taxonomy and environment of OTUs

| OTUID | Taxonomy | Environment |
|---|---|---|
| denovo3176 | Eukaryota; Amoebozoa; Archamoebae; Entamoebidae; Entamoeba | gut_resident |
| denovo3125 | Eukaryota; Amoebozoa; Archamoebae; Entamoebidae; Entamoeba | gut_resident |
| denovo2765 | Eukaryota; Amoebozoa; Archamoebae; Entamoebidae; Entamoeba | gut_resident |
| denovo1571 | Eukaryota; Amoebozoa; Archamoebae; Entamoebidae; Entamoeba | gut_resident |
| denovo1775 | Eukaryota; Amoebozoa; Archamoebae; Entamoebidae; Entamoeba | gut_resident |
| denovo659 | Eukaryota; Amoebozoa; Archamoebae; Entamoebidae; Entamoeba | gut_resident |
| denovo1280 | Eukaryota; Amoebozoa; Archamoebae; Entamoebidae; Entamoeba | gut_resident |
| denovo828 | Eukaryota; Amoebozoa; Archamoebae; Entamoebidae; Entamoeba | gut_resident |
| denovo18 | Eukaryota; Amoebozoa; Archamoebae; Entamoebidae; Entamoeba | gut_resident |
| denovo2424 | Eukaryota; Amoebozoa; Archamoebae; Entamoebidae; Entamoeba | gut_resident |
| denovo908 | Eukaryota; Amoebozoa; Archamoebae; Entamoebidae; Entamoeba | gut_resident |
| denovo752 | Eukaryota; Amoebozoa; Archamoebae; Entamoebidae; Entamoeba | gut_resident |
| denovo719 | Eukaryota; Amoebozoa; Archamoebae; Entamoebidae; Entamoeba | gut_resident |
| denovo701 | Eukaryota; Amoebozoa; Archamoebae; Entamoebidae; Entamoeba | gut_resident |
| denovo609 | Eukaryota; Amoebozoa; Archamoebae; Entamoebidae; Entamoeba | gut_resident |
| denovo3013 | Eukaryota; Amoebozoa; Archamoebae; Entamoebidae; Entamoeba | gut_resident |
| denovo1107 | Eukaryota; Amoebozoa; Archamoebae; Entamoebidae; Entamoeba | gut_resident |
| denovo1201 | Eukaryota; Amoebozoa; Archamoebae; Entamoebidae; Entamoeba | gut_resident |
| denovo1478 | Eukaryota; Amoebozoa; Archamoebae; Entamoebidae; Entamoeba | gut_resident |
| denovo2263 | Eukaryota; Amoebozoa; Archamoebae; Entamoebidae; Entamoeba | gut_resident |
| denovo243 | Eukaryota; Amoebozoa; Archamoebae; Entamoebidae; Entamoeba | gut_resident |
| denovo210 | Eukaryota; Amoebozoa; Archamoebae; Entamoebidae; Entamoeba | gut_resident |
| denovo2 | Eukaryota; Amoebozoa; Archamoebae; Entamoebidae; Entamoeba | gut_resident |
| denovo2813 | Eukaryota; Amoebozoa; Archamoebae; Entamoebidae; Entamoeba | gut_resident |
| denovo2652 | Eukaryota; Amoebozoa; Archamoebae; Entamoebidae; Entamoeba | gut_resident |
| denovo1449 | Eukaryota; Amoebozoa; Archamoebae; Entamoebidae; Entamoeba | gut_resident |
| denovo1716 | Eukaryota; Amoebozoa; Archamoebae; Entamoebidae; Entamoeba | gut_resident |
| denovo1943 | Eukaryota; Amoebozoa; Archamoebae; Entamoebidae; Entamoeba | gut_resident |
| denovo1999 | Eukaryota; Amoebozoa; Archamoebae; Entamoebidae; Entamoeba | gut_resident |
| denovo963 | Eukaryota; Amoebozoa; Archamoebae; Entamoebidae; Entamoeba | gut_resident |
| denovo758 | Eukaryota; Amoebozoa; Archamoebae; Entamoebidae; Entamoeba | gut_resident |
| denovo336 | Eukaryota; Amoebozoa; Archamoebae; Entamoebidae; Entamoeba | gut_resident |
| denovo1008 | Eukaryota; Amoebozoa; Archamoebae; Entamoebidae; Entamoeba | gut_resident |
| denovo1172 | Eukaryota; Amoebozoa; Archamoebae; Entamoebidae; Entamoeba | gut_resident |
| denovo558 | Eukaryota; Amoebozoa; Archamoebae; Entamoebidae; Entamoeba | gut_resident |
| denovo3 | Eukaryota; Amoebozoa; Archamoebae; Entamoebidae; Entamoeba | gut_resident |
| denovo227 | Eukaryota; Amoebozoa; Archamoebae; Entamoebidae; Entamoeba | gut_resident |
| denovo868 | Eukaryota; Amoebozoa; Archamoebae; Entamoeba; Entamoeba; Entamoeba; Entamoeba | gut_resident |

| | | |
|---|---|---|
| denovo3048 | Eukaryota; Amoebozoa; Archamoebae; Entamoeba; Entamoeba; Entamoeba; Entamoeba | gut_resident |
| denovo865 | Eukaryota; Amoebozoa; Archamoebae; Entamoeba; Entamoeba; Entamoeba; Entamoeba; Entamoeba_bovis | gut_resident |
| denovo294 | Eukaryota; Amoebozoa; Archamoebae; Entamoeba; Entamoeba; Entamoeba; Entamoeba; Entamoeba_bovis | gut_resident |
| denovo2594 | Eukaryota; Amoebozoa; Archamoebae; Entamoeba; Entamoeba; Entamoeba; Entamoeba; Entamoeba_bovis | gut_resident |
| denovo2593 | Eukaryota; Amoebozoa; Archamoebae; Entamoeba; Entamoeba; Entamoeba; Entamoeba; Entamoeba_bovis | gut_resident |
| denovo1190 | Eukaryota; Amoebozoa; Archamoebae; Entamoeba; Entamoeba; Entamoeba; Entamoeba; Entamoeba_bovis | gut_resident |
| denovo2224 | Eukaryota; Amoebozoa; Archamoebae; Entamoeba; Entamoeba; Entamoeba; Entamoeba; Entamoeba_bovis | gut_resident |
| denovo5 | Eukaryota; Amoebozoa; Archamoebae; Entamoebidae; Entamoeba | gut_resident |
| denovo922 | Eukaryota; Amoebozoa; Archamoebae; Entamoebidae; Entamoeba | gut_resident |
| denovo269 | Eukaryota; Amoebozoa; Archamoebae; Entamoebidae; Entamoeba | gut_resident |
| denovo2453 | Eukaryota; Amoebozoa; Archamoebae; Entamoebidae; Entamoeba | gut_resident |
| denovo1082 | Eukaryota; Amoebozoa; Archamoebae; Entamoebidae; Entamoeba | gut_resident |
| denovo1618 | Eukaryota; Amoebozoa; Archamoebae; Entamoebidae; Entamoeba | gut_resident |
| denovo2647 | Eukaryota; Amoebozoa; Archamoebae; Entamoeba; Entamoeba; Entamoeba; Entamoeba; Entamoeba_coli | gut_resident |
| denovo2467 | Eukaryota; Amoebozoa; Archamoebae; Entamoeba; Entamoeba; Entamoeba; Entamoeba; Entamoeba_coli | gut_resident |
| denovo1769 | Eukaryota; Amoebozoa; Archamoebae; Entamoeba; Entamoeba; Entamoeba; Entamoeba; Entamoeba_coli | gut_resident |
| denovo2064 | Eukaryota; Amoebozoa; Archamoebae; Entamoeba; Entamoeba; Entamoeba; Entamoeba; Entamoeba_coli | gut_resident |
| denovo1442 | Eukaryota; Amoebozoa; Archamoebae; Entamoeba; Entamoeba; Entamoeba; Entamoeba; Entamoeba_coli | gut_resident |
| denovo601 | Eukaryota; Amoebozoa; Archamoebae; Entamoeba; Entamoeba; Entamoeba; Entamoeba; Entamoeba_coli | gut_resident |
| denovo17 | Eukaryota; Amoebozoa; Archamoebae; Entamoeba; Entamoeba; Entamoeba; Entamoeba; Entamoeba_coli | gut_resident |
| denovo2365 | Eukaryota; Amoebozoa; Archamoebae; Entamoeba; Entamoeba; Entamoeba; Entamoeba; Entamoeba_coli | gut_resident |
| denovo2460 | Eukaryota; Amoebozoa; Archamoebae; Entamoeba; Entamoeba; Entamoeba; Entamoeba; Entamoeba_coli | gut_resident |
| denovo1183 | Eukaryota; Amoebozoa; Archamoebae; Entamoeba; Entamoeba; Entamoeba; Entamoeba; Entamoeba_coli | gut_resident |
| denovo1064 | Eukaryota; Amoebozoa; Archamoebae; Entamoeba; Entamoeba; Entamoeba; Entamoeba; Entamoeba_coli | gut_resident |
| denovo31 | Eukaryota; Amoebozoa; Archamoebae; Entamoeba; Entamoeba; Entamoeba; Entamoeba; Entamoeba_coli | gut_resident |
| denovo1186 | Eukaryota; Amoebozoa; Archamoebae; Entamoeba; Entamoeba; Entamoeba; Entamoeba; Entamoeba_coli | gut_resident |
| denovo1974 | Eukaryota; Amoebozoa; Archamoebae; Entamoeba; Entamoeba; Entamoeba; Entamoeba; Entamoeba_coli | gut_resident |
| denovo356 | Eukaryota; Amoebozoa; Archamoebae; Entamoeba; Entamoeba; Entamoeba; Entamoeba; Entamoeba_coli | gut_resident |
| denovo1890 | Eukaryota; Amoebozoa; Archamoebae; Entamoeba; Entamoeba; Entamoeba; Entamoeba; Entamoeba_coli | gut_resident |
| denovo1390 | Eukaryota; Amoebozoa; Archamoebae; Entamoeba; Entamoeba; Entamoeba; Entamoeba; Entamoeba_coli | gut_resident |
| denovo36 | Eukaryota; Amoebozoa; Archamoebae; Entamoeba; Entamoeba; Entamoeba; Entamoeba; Entamoeba_coli | gut_resident |
| denovo2915 | Eukaryota; Amoebozoa; Archamoebae; Entamoeba; Entamoeba; Entamoeba; Entamoeba; Entamoeba_coli | gut_resident |
| denovo1254 | Eukaryota; Amoebozoa; Archamoebae; Entamoeba; Entamoeba; Entamoeba; Entamoeba; Entamoeba_coli | gut_resident |
| denovo2618 | Eukaryota; Amoebozoa; Archamoebae; Entamoeba; Entamoeba; Entamoeba; Entamoeba; Entamoeba_coli | gut_resident |
| denovo42 | Eukaryota; Amoebozoa; Archamoebae; Entamoeba; Entamoeba; Entamoeba; Entamoeba; Entamoeba_coli | gut_resident |
| denovo2058 | Eukaryota; Amoebozoa; Archamoebae; Entamoeba; Entamoeba; Entamoeba; Entamoeba; Entamoeba_coli | gut_resident |
| denovo2756 | Eukaryota; Amoebozoa; Archamoebae; Entamoeba; Entamoeba; Entamoeba; Entamoeba; Entamoeba_coli | gut_resident |
| denovo50 | Eukaryota; Amoebozoa; Archamoebae; Entamoeba; Entamoeba; Entamoeba; Entamoeba; Entamoeba_coli | gut_resident |
| denovo3105 | Eukaryota; Amoebozoa; Archamoebae; Entamoeba; Entamoeba; Entamoeba; Entamoeba; Entamoeba_coli | gut_resident |
| denovo1957 | Eukaryota; Amoebozoa; Archamoebae; Entamoeba; Entamoeba; Entamoeba; Entamoeba; Entamoeba_coli | gut_resident |

| | | |
|---|---|---|
| denovo56 | Eukaryota; Amoebozoa; Archamoebae; Entamoeba; Entamoeba; Entamoeba; Entamoeba; Entamoeba_coli | gut_resident |
| denovo928 | Eukaryota; Amoebozoa; Archamoebae; Entamoeba; Entamoeba; Entamoeba; Entamoeba; Entamoeba_coli | gut_resident |
| denovo583 | Eukaryota; Amoebozoa; Archamoebae; Entamoeba; Entamoeba; Entamoeba; Entamoeba; Entamoeba_coli | gut_resident |
| denovo3292 | Eukaryota; Amoebozoa; Archamoebae; Entamoeba; Entamoeba; Entamoeba; Entamoeba; Entamoeba_coli | gut_resident |
| denovo1409 | Eukaryota; Amoebozoa; Archamoebae; Entamoeba; Entamoeba; Entamoeba; Entamoeba; Entamoeba_coli | gut_resident |
| denovo1995 | Eukaryota; Amoebozoa; Archamoebae; Entamoeba; Entamoeba; Entamoeba; Entamoeba; Entamoeba_coli | gut_resident |
| denovo2390 | Eukaryota; Amoebozoa; Archamoebae; Entamoeba; Entamoeba; Entamoeba; Entamoeba; Entamoeba_coli | gut_resident |
| denovo603 | Eukaryota; Amoebozoa; Archamoebae; Entamoeba; Entamoeba; Entamoeba; Entamoeba; Entamoeba_coli | gut_resident |
| denovo1024 | Eukaryota; Amoebozoa; Archamoebae; Entamoeba; Entamoeba; Entamoeba; Entamoeba; Entamoeba_coli | gut_resident |
| denovo1827 | Eukaryota; Amoebozoa; Archamoebae; Entamoeba; Entamoeba; Entamoeba; Entamoeba; Entamoeba_coli | gut_resident |
| denovo2912 | Eukaryota; Amoebozoa; Archamoebae; Entamoeba; Entamoeba; Entamoeba; Entamoeba; Entamoeba_coli | gut_resident |
| denovo2345 | Eukaryota; Amoebozoa; Archamoebae; Entamoeba; Entamoeba; Entamoeba; Entamoeba; Entamoeba_coli | gut_resident |
| denovo64 | Eukaryota; Amoebozoa; Archamoebae; Entamoeba; Entamoeba; Entamoeba; Entamoeba; Entamoeba_coli | gut_resident |
| denovo1524 | Eukaryota; Amoebozoa; Archamoebae; Entamoeba; Entamoeba; Entamoeba; Entamoeba; Entamoeba_coli | gut_resident |
| denovo1892 | Eukaryota; Amoebozoa; Archamoebae; Entamoeba; Entamoeba; Entamoeba; Entamoeba; Entamoeba_coli | gut_resident |
| denovo82 | Eukaryota; Amoebozoa; Archamoebae; Entamoeba; Entamoeba; Entamoeba; Entamoeba; Entamoeba_coli | gut_resident |
| denovo580 | Eukaryota; Amoebozoa; Archamoebae; Entamoeba; Entamoeba; Entamoeba; Entamoeba; Entamoeba_sp. | gut_resident |
| denovo167 | Eukaryota; Amoebozoa; Archamoebae; Entamoeba; Entamoeba; Entamoeba; Entamoeba; Entamoeba_sp. | gut_resident |
| denovo2787 | Eukaryota; Amoebozoa; Archamoebae; Entamoeba; Entamoeba; Entamoeba; Entamoeba; Entamoeba_histolytica | gut_resident |
| denovo2542 | Eukaryota; Amoebozoa; Archamoebae; Entamoeba; Entamoeba; Entamoeba; Entamoeba; Entamoeba_histolytica | gut_resident |
| denovo2387 | Eukaryota; Amoebozoa; Archamoebae; Entamoeba; Entamoeba; Entamoeba; Entamoeba; Entamoeba_histolytica | gut_resident |
| denovo1414 | Eukaryota; Amoebozoa; Archamoebae; Entamoeba; Entamoeba; Entamoeba; Entamoeba; Entamoeba_histolytica | gut_resident |
| denovo1922 | Eukaryota; Amoebozoa; Archamoebae; Entamoeba; Entamoeba; Entamoeba; Entamoeba; Entamoeba_histolytica | gut_resident |
| denovo1630 | Eukaryota; Amoebozoa; Archamoebae; Entamoeba; Entamoeba; Entamoeba; Entamoeba; Entamoeba_histolytica | gut_resident |
| denovo716 | Eukaryota; Amoebozoa; Archamoebae; Entamoeba; Entamoeba; Entamoeba; Entamoeba; Entamoeba_histolytica | gut_resident |
| denovo621 | Eukaryota; Amoebozoa; Archamoebae; Entamoeba; Entamoeba; Entamoeba; Entamoeba; Entamoeba_histolytica | gut_resident |
| denovo1551 | Eukaryota; Amoebozoa; Archamoebae; Entamoeba; Entamoeba; Entamoeba; Entamoeba; Entamoeba_histolytica | gut_resident |
| denovo19 | Eukaryota; Amoebozoa; Archamoebae; Entamoeba; Entamoeba; Entamoeba; Entamoeba; Entamoeba_histolytica | gut_resident |
| denovo120 | Eukaryota; Amoebozoa; Archamoebae; Entamoeba; Entamoeba; Entamoeba; Entamoeba; Entamoeba_sp. | gut_resident |
| denovo1902 | Eukaryota; Amoebozoa; Archamoebae; Entamoeba; Entamoeba; Entamoeba; Entamoeba; Entamoeba_sp. | gut_resident |
| denovo1035 | Eukaryota; Amoebozoa; Archamoebae; Entamoeba; Entamoeba; Entamoeba; Entamoeba; Entamoeba_sp. | gut_resident |
| denovo1395 | Eukaryota; Amoebozoa; Archamoebae; Entamoeba; Entamoeba; Entamoeba; Entamoeba; Entamoeba_sp. | gut_resident |
| denovo13 | Eukaryota; Amoebozoa; Archamoebae; Entamoeba; Entamoeba; Entamoeba; Entamoeba; Entamoeba_sp. | gut_resident |
| denovo1544 | Eukaryota; Amoebozoa; Archamoebae; Entamoeba; Entamoeba; Entamoeba; Entamoeba; Entamoeba_sp. | gut_resident |
| denovo131 | Eukaryota; Amoebozoa; Archamoebae; Entamoeba; Entamoeba; Entamoeba; Entamoeba; Entamoeba_sp. | gut_resident |
| denovo143 | Eukaryota; Amoebozoa; Archamoebae; Entamoeba; Entamoeba; Entamoeba; Entamoeba; Entamoeba_sp. | gut_resident |
| denovo185 | Eukaryota; Amoebozoa; Archamoebae; Entamoeba; Entamoeba; Entamoeba; Entamoeba; Entamoeba_sp. | gut_resident |
| denovo3279 | Eukaryota; Amoebozoa; Archamoebae; Entamoeba; Entamoeba; Entamoeba; Entamoeba; Entamoeba_sp. | gut_resident |
| denovo797 | Eukaryota; Amoebozoa; Archamoebae; Entamoeba; Entamoeba; Entamoeba; Entamoeba; Entamoeba_sp. | gut_resident |
| denovo2492 | Eukaryota; Amoebozoa; Archamoebae; Entamoeba; Entamoeba; Entamoeba; Entamoeba; Entamoeba_sp. | gut_resident |

| | | |
|---|---|---|
| denovo1441 | Eukaryota; Amoebozoa; Archamoebae; Entamoeba; Entamoeba; Entamoeba; Entamoeba; Entamoeba_sp. | gut_resident |
| denovo891 | Eukaryota; Amoebozoa; Archamoebae; Entamoeba; Entamoeba; Entamoeba; Entamoeba; Entamoeba_sp. | gut_resident |
| denovo20 | Eukaryota; Amoebozoa; Archamoebae; Entamoeba; Entamoeba; Entamoeba; Entamoeba; Entamoeba_sp. | gut_resident |
| denovo2167 | Eukaryota; Amoebozoa; Archamoebae; Entamoeba; Entamoeba; Entamoeba; Entamoeba; Entamoeba_sp. | gut_resident |
| denovo973 | Eukaryota; Amoebozoa; Archamoebae; Entamoeba; Entamoeba; Entamoeba; Entamoeba; Entamoeba_sp. | gut_resident |
| denovo517 | Eukaryota; Amoebozoa; Archamoebae; Entamoeba; Entamoeba; Entamoeba; Entamoeba; Entamoeba_sp. | gut_resident |
| denovo232 | Eukaryota; Amoebozoa; Archamoebae; Entamoeba; Entamoeba; Entamoeba; Entamoeba; Entamoeba_sp. | gut_resident |
| denovo2614 | Eukaryota; Amoebozoa; Archamoebae; Entamoeba; Entamoeba; Entamoeba; Entamoeba; Entamoeba_sp. | gut_resident |
| denovo25 | Eukaryota; Amoebozoa; Archamoebae; Entamoeba; Entamoeba; Entamoeba; Entamoeba; Entamoeba_sp. | gut_resident |
| denovo1011 | Eukaryota; Amoebozoa; Archamoebae; Entamoeba; Entamoeba; Entamoeba; Entamoeba; Entamoeba_sp. | gut_resident |
| denovo919 | Eukaryota; Amoebozoa; Archamoebae; Entamoeba; Entamoeba; Entamoeba; Entamoeba; Entamoeba_sp. | gut_resident |
| denovo1941 | Eukaryota; Amoebozoa; Archamoebae; Entamoeba; Entamoeba; Entamoeba; Entamoeba; Entamoeba_sp. | gut_resident |
| denovo255 | Eukaryota; Amoebozoa; Archamoebae; Entamoeba; Entamoeba; Entamoeba; Entamoeba; Entamoeba_sp. | gut_resident |
| denovo261 | Eukaryota; Amoebozoa; Archamoebae; Entamoeba; Entamoeba; Entamoeba; Entamoeba; Entamoeba_sp. | gut_resident |
| denovo303 | Eukaryota; Amoebozoa; Archamoebae; Entamoeba; Entamoeba; Entamoeba; Entamoeba; Entamoeba_sp. | gut_resident |
| denovo2546 | Eukaryota; Amoebozoa; Archamoebae; Entamoeba; Entamoeba; Entamoeba; Entamoeba; Entamoeba_sp. | gut_resident |
| denovo714 | Eukaryota; Amoebozoa; Archamoebae; Entamoeba; Entamoeba; Entamoeba; Entamoeba; Entamoeba_sp. | gut_resident |
| denovo32 | Eukaryota; Amoebozoa; Archamoebae; Entamoeba; Entamoeba; Entamoeba; Entamoeba; Entamoeba_sp. | gut_resident |
| denovo665 | Eukaryota; Amoebozoa; Archamoebae; Entamoeba; Entamoeba; Entamoeba; Entamoeba; Entamoeba_sp. | gut_resident |
| denovo334 | Eukaryota; Amoebozoa; Archamoebae; Entamoeba; Entamoeba; Entamoeba; Entamoeba; Entamoeba_sp. | gut_resident |
| denovo1393 | Eukaryota; Amoebozoa; Archamoebae; Entamoeba; Entamoeba; Entamoeba; Entamoeba; Entamoeba_sp. | gut_resident |
| denovo1828 | Eukaryota; Amoebozoa; Archamoebae; Entamoeba; Entamoeba; Entamoeba; Entamoeba; Entamoeba_sp. | gut_resident |
| denovo39 | Eukaryota; Amoebozoa; Archamoebae; Entamoeba; Entamoeba; Entamoeba; Entamoeba; Entamoeba_sp. | gut_resident |
| denovo1933 | Eukaryota; Amoebozoa; Archamoebae; Entamoeba; Entamoeba; Entamoeba; Entamoeba; Entamoeba_sp. | gut_resident |
| denovo2105 | Eukaryota; Amoebozoa; Archamoebae; Entamoeba; Entamoeba; Entamoeba; Entamoeba; Entamoeba_sp. | gut_resident |
| denovo418 | Eukaryota; Amoebozoa; Archamoebae; Entamoeba; Entamoeba; Entamoeba; Entamoeba; Entamoeba_sp. | gut_resident |
| denovo668 | Eukaryota; Amoebozoa; Archamoebae; Entamoeba; Entamoeba; Entamoeba; Entamoeba; Entamoeba_sp. | gut_resident |
| denovo1440 | Eukaryota; Amoebozoa; Archamoebae; Entamoeba; Entamoeba; Entamoeba; Entamoeba; Entamoeba_sp. | gut_resident |
| denovo52 | Eukaryota; Amoebozoa; Archamoebae; Entamoeba; Entamoeba; Entamoeba; Entamoeba; Entamoeba_sp. | gut_resident |
| denovo117 | Eukaryota; Amoebozoa; Archamoebae; Entamoeba; Entamoeba; Entamoeba; Entamoeba; Entamoeba_sp. | gut_resident |
| denovo130 | Eukaryota; Amoebozoa; Archamoebae; Entamoeba; Entamoeba; Entamoeba; Entamoeba; Entamoeba_sp. | gut_resident |
| denovo74 | Eukaryota; Amoebozoa; Archamoebae; Entamoeba; Entamoeba; Entamoeba; Entamoeba; Entamoeba_sp. | gut_resident |
| denovo2674 | Eukaryota; Amoebozoa; Archamoebae; Entamoeba; Entamoeba; Entamoeba; Entamoeba; Entamoeba_sp. | gut_resident |
| denovo108 | Eukaryota; Amoebozoa; Archamoebae; Entamoeba; Entamoeba; Entamoeba; Entamoeba; Entamoeba_sp. | gut_resident |
| denovo162 | Eukaryota; Amoebozoa; Archamoebae; Entamoeba; Entamoeba; Entamoeba; Entamoeba; Entamoeba_sp. | gut_resident |
| denovo95 | Eukaryota; Amoebozoa; Archamoebae; Entamoeba; Entamoeba; Entamoeba; Entamoeba; Entamoeba_sp. | gut_resident |
| denovo219 | Eukaryota; Amoebozoa; Archamoebae; Entamoeba; Entamoeba; Entamoeba; Entamoeba; Entamoeba_sp. | gut_resident |
| denovo218 | Eukaryota; Amoebozoa; Archamoebae; Entamoeba; Entamoeba; Entamoeba; Entamoeba; Entamoeba_sp. | gut_resident |
| denovo593 | Eukaryota; Amoebozoa; Archamoebae; Entamoeba; Entamoeba; Entamoeba; Entamoeba; Entamoeba_sp. | gut_resident |
| denovo2627 | Eukaryota; Amoebozoa; Archamoebae; Entamoeba; Entamoeba; Entamoeba; Entamoeba; Entamoeba_sp. | gut_resident |

| | | |
|---|---|---|
| denovo245 | Eukaryota; Amoebozoa; Archamoebae; Entamoeba; Entamoeba; Entamoeba; Entamoeba; Entamoeba_sp. | gut_resident |
| denovo77 | Eukaryota; Amoebozoa; Archamoebae; Entamoeba; Entamoeba; Entamoeba; Entamoeba; Entamoeba_sp. | gut_resident |
| denovo378 | Eukaryota; Amoebozoa; Archamoebae; Entamoeba; Entamoeba; Entamoeba; Entamoeba; Entamoeba_sp. | gut_resident |
| denovo85 | Eukaryota; Amoebozoa; Archamoebae; Entamoeba; Entamoeba; Entamoeba; Entamoeba; Entamoeba_sp. | gut_resident |
| denovo94 | Eukaryota; Amoebozoa; Archamoebae; Entamoeba; Entamoeba; Entamoeba; Entamoeba; Entamoeba_sp. | gut_resident |
| denovo1992 | Eukaryota; Amoebozoa; Archamoebae; Entamoeba; Entamoeba; Entamoeba; Entamoeba | gut_resident |
| denovo1770 | Eukaryota; Amoebozoa; Archamoebae; Entamoeba; Entamoeba; Entamoeba; Entamoeba | gut_resident |
| denovo543 | Eukaryota; Amoebozoa; Archamoebae; Entamoeba; Entamoeba; Entamoeba; Entamoeba | gut_resident |
| denovo470 | Eukaryota; Amoebozoa; Archamoebae; Entamoeba; Entamoeba; Entamoeba; Entamoeba | gut_resident |
| denovo14 | Eukaryota; Amoebozoa; Archamoebae; Entamoeba; Entamoeba; Entamoeba; Entamoeba | gut_resident |
| denovo978 | Eukaryota; Alveolata | unknown |
| denovo2023 | Eukaryota; Alveolata | unknown |
| denovo75 | Eukaryota; Alveolata; Apicomplexa; Conoidasida; Coccidia; Adeleorina; Adelina | invert_parasite |
| denovo66 | Eukaryota; Alveolata; Apicomplexa; Conoidasida; Coccidia; Adeleorina; Adelina | invert_parasite |
| denovo2860 | Eukaryota; Alveolata; Apicomplexa; Conoidasida; Coccidia; Adeleorina; Adelina | invert_parasite |
| denovo2753 | Eukaryota; Alveolata; Apicomplexa; Conoidasida; Coccidia; Adeleorina; Adelina | invert_parasite |
| denovo2456 | Eukaryota; Alveolata; Apicomplexa; Conoidasida; Coccidia; Adeleorina; Adelina | invert_parasite |
| denovo2403 | Eukaryota; Alveolata; Apicomplexa; Conoidasida; Coccidia; Adeleorina; Adelina | invert_parasite |
| denovo2327 | Eukaryota; Alveolata; Apicomplexa; Conoidasida; Coccidia; Adeleorina; Adelina | invert_parasite |
| denovo2313 | Eukaryota; Alveolata; Apicomplexa; Conoidasida; Coccidia; Adeleorina; Adelina | invert_parasite |
| denovo1233 | Eukaryota; Alveolata; Apicomplexa; Conoidasida; Coccidia; Adeleorina; Adelina | invert_parasite |
| denovo1650 | Eukaryota; Alveolata; Apicomplexa; Conoidasida; Coccidia; Adeleorina; Adelina | invert_parasite |
| denovo1817 | Eukaryota; Alveolata; Apicomplexa; Conoidasida; Coccidia; Adeleorina; Adelina | invert_parasite |
| denovo1882 | Eukaryota; Alveolata; Apicomplexa; Conoidasida; Coccidia; Adeleorina; Adelina | invert_parasite |
| denovo1881 | Eukaryota; Alveolata; Apicomplexa; Conoidasida; Coccidia; Adeleorina; Adelina | invert_parasite |
| denovo1942 | Eukaryota; Alveolata; Apicomplexa; Conoidasida; Coccidia; Adeleorina; Adelina | invert_parasite |
| denovo2113 | Eukaryota; Alveolata; Apicomplexa; Conoidasida; Coccidia; Adeleorina; Adelina | invert_parasite |
| denovo2296 | Eukaryota; Alveolata; Apicomplexa; Conoidasida; Coccidia; Adeleorina; Adelina | invert_parasite |
| denovo2385 | Eukaryota; Alveolata; Apicomplexa; Conoidasida; Coccidia; Adeleorina; Adelina; Adelina_dimidiata | invert_parasite |
| denovo2277 | Eukaryota; Alveolata; Apicomplexa; Conoidasida; Coccidia; Adeleorina; Adelina; Adelina_dimidiata | invert_parasite |
| denovo834 | Eukaryota; Alveolata; Apicomplexa; Conoidasida; Coccidia; Eimeriorina; Cyclospora | gut_resident |
| denovo837 | Eukaryota; Alveolata; Apicomplexa; Conoidasida; Coccidia; Eimeriorina; Cyclospora; Cyclospora_cayetanensis | gut_resident |
| denovo577 | Eukaryota; Alveolata; Apicomplexa; Conoidasida; Coccidia; Eucoccidiorida; Eimeriorina | probable_invert_parasite |
| denovo360 | Eukaryota; Alveolata; Apicomplexa; Conoidasida; Coccidia; Eucoccidiorida; Eimeriorina; Eimeriidae | probable_invert_parasite |
| denovo308 | Eukaryota; Alveolata; Apicomplexa; Conoidasida; Coccidia; Eucoccidiorida; Eimeriorina; Eimeriidae | probable_invert_parasite |
| denovo826 | Eukaryota; Alveolata; Apicomplexa; Conoidasida; Cryptosporida; Cryptosporidium; Cryptosporidium; Colpodella_angusta | environmental |
| denovo2902 | Eukaryota; Alveolata; Apicomplexa; Conoidasida; Cryptosporida; Cryptosporidium; Cryptosporidium; Colpodella_angusta | environmental |
| denovo2749 | Eukaryota; Alveolata; Apicomplexa; Conoidasida; Cryptosporida; Cryptosporidium; Cryptosporidium; Colpodella_angusta | environmental |
| denovo519 | Eukaryota; Alveolata; Apicomplexa; Conoidasida; Cryptosporida; Cryptosporidium; Cryptosporidium; Colpodella_sp._HLJ | environmental |
| denovo416 | Eukaryota; Alveolata; Apicomplexa; Conoidasida; Cryptosporida; Cryptosporidium; Cryptosporidium; Colpodella_sp._HLJ | environmental |

| | | |
|---|---|---|
| denovo852 | Eukaryota; Alveolata; Apicomplexa; Conoidasida; Gregarinasina; Eugregarinorida | probable_invert_parasite |
| denovo461 | Eukaryota; Alveolata; Apicomplexa; Conoidasida; Gregarinasina; Eugregarinorida | probable_invert_parasite |
| denovo3216 | Eukaryota; Alveolata; Apicomplexa; Conoidasida; Gregarinasina; Eugregarinorida | probable_invert_parasite |
| denovo3213 | Eukaryota; Alveolata; Apicomplexa; Conoidasida; Gregarinasina; Eugregarinorida | probable_invert_parasite |
| denovo3066 | Eukaryota; Alveolata; Apicomplexa; Conoidasida; Gregarinasina; Eugregarinorida | probable_invert_parasite |
| denovo2936 | Eukaryota; Alveolata; Apicomplexa; Conoidasida; Gregarinasina; Eugregarinorida | probable_invert_parasite |
| denovo2602 | Eukaryota; Alveolata; Apicomplexa; Conoidasida; Gregarinasina; Eugregarinorida | probable_invert_parasite |
| denovo1115 | Eukaryota; Alveolata; Apicomplexa; Conoidasida; Gregarinasina; Eugregarinorida | probable_invert_parasite |
| denovo1229 | Eukaryota; Alveolata; Apicomplexa; Conoidasida; Gregarinasina; Eugregarinorida | probable_invert_parasite |
| denovo1324 | Eukaryota; Alveolata; Apicomplexa; Conoidasida; Gregarinasina; Eugregarinorida | probable_invert_parasite |
| denovo2122 | Eukaryota; Alveolata; Apicomplexa; Conoidasida; Gregarinasina; Eugregarinorida | probable_invert_parasite |
| denovo2145 | Eukaryota; Alveolata; Apicomplexa; Conoidasida; Gregarinasina; Eugregarinorida | probable_invert_parasite |
| denovo264 | Eukaryota; Alveolata; Apicomplexa; Conoidasida; Gregarinasina; Eugregarinorida; Ascogregarina | probable_invert_parasite |
| denovo745 | Eukaryota; Alveolata; Apicomplexa; Conoidasida; Gregarinasina; Eugregarinorida; Diabrotica; Diabrotica_virgifera_virgifera_(western_corn_rootworm) | probable_invert_parasite |
| denovo1063 | Eukaryota; Alveolata; Apicomplexa; Conoidasida; Gregarinasina; Eugregarinorida; Diabrotica; Diabrotica_virgifera_virgifera_(western_corn_rootworm) | probable_invert_parasite |
| denovo1154 | Eukaryota; Alveolata; Apicomplexa; Conoidasida; Gregarinasina; Eugregarinorida; Diabrotica; Diabrotica_virgifera_virgifera_(western_corn_rootworm) | probable_invert_parasite |
| denovo977 | Eukaryota; Alveolata; Apicomplexa; Conoidasida; Gregarinasina; Eugregarinorida; Elev-18S-1089 | probable_invert_parasite |
| denovo932 | Eukaryota; Alveolata; Apicomplexa; Conoidasida; Gregarinasina; Eugregarinorida; Elev-18S-1089 | probable_invert_parasite |
| denovo733 | Eukaryota; Alveolata; Apicomplexa; Conoidasida; Gregarinasina; Eugregarinorida; Elev-18S-1089 | probable_invert_parasite |
| denovo706 | Eukaryota; Alveolata; Apicomplexa; Conoidasida; Gregarinasina; Eugregarinorida; Elev-18S-1089 | probable_invert_parasite |
| denovo432 | Eukaryota; Alveolata; Apicomplexa; Conoidasida; Gregarinasina; Eugregarinorida; Elev-18S-1089 | probable_invert_parasite |
| denovo358 | Eukaryota; Alveolata; Apicomplexa; Conoidasida; Gregarinasina; Eugregarinorida; Elev-18S-1089 | probable_invert_parasite |
| denovo3310 | Eukaryota; Alveolata; Apicomplexa; Conoidasida; Gregarinasina; Eugregarinorida; Elev-18S-1089 | probable_invert_parasite |
| denovo3070 | Eukaryota; Alveolata; Apicomplexa; Conoidasida; Gregarinasina; Eugregarinorida; Elev-18S-1089 | probable_invert_parasite |
| denovo2949 | Eukaryota; Alveolata; Apicomplexa; Conoidasida; Gregarinasina; Eugregarinorida; Elev-18S-1089 | probable_invert_parasite |
| denovo2803 | Eukaryota; Alveolata; Apicomplexa; Conoidasida; Gregarinasina; Eugregarinorida; Elev-18S-1089 | probable_invert_parasite |
| denovo2754 | Eukaryota; Alveolata; Apicomplexa; Conoidasida; Gregarinasina; Eugregarinorida; Elev-18S-1089 | probable_invert_parasite |
| denovo2716 | Eukaryota; Alveolata; Apicomplexa; Conoidasida; Gregarinasina; Eugregarinorida; Elev-18S-1089 | probable_invert_parasite |
| denovo268 | Eukaryota; Alveolata; Apicomplexa; Conoidasida; Gregarinasina; Eugregarinorida; Elev-18S-1089 | probable_invert_parasite |
| denovo2562 | Eukaryota; Alveolata; Apicomplexa; Conoidasida; Gregarinasina; Eugregarinorida; Elev-18S-1089 | probable_invert_parasite |
| denovo2560 | Eukaryota; Alveolata; Apicomplexa; Conoidasida; Gregarinasina; Eugregarinorida; Elev-18S-1089 | probable_invert_parasite |
| denovo2341 | Eukaryota; Alveolata; Apicomplexa; Conoidasida; Gregarinasina; Eugregarinorida; Elev-18S-1089 | probable_invert_parasite |
| denovo215 | Eukaryota; Alveolata; Apicomplexa; Conoidasida; Gregarinasina; Eugregarinorida; Elev-18S-1089 | probable_invert_parasite |
| denovo1165 | Eukaryota; Alveolata; Apicomplexa; Conoidasida; Gregarinasina; Eugregarinorida; Elev-18S-1089 | probable_invert_parasite |
| denovo1194 | Eukaryota; Alveolata; Apicomplexa; Conoidasida; Gregarinasina; Eugregarinorida; Elev-18S-1089 | probable_invert_parasite |
| denovo1420 | Eukaryota; Alveolata; Apicomplexa; Conoidasida; Gregarinasina; Eugregarinorida; Elev-18S-1089 | probable_invert_parasite |
| denovo1647 | Eukaryota; Alveolata; Apicomplexa; Conoidasida; Gregarinasina; Eugregarinorida; Elev-18S-1089 | probable_invert_parasite |
| denovo1690 | Eukaryota; Alveolata; Apicomplexa; Conoidasida; Gregarinasina; Eugregarinorida; Elev-18S-1089 | probable_invert_parasite |
| denovo1751 | Eukaryota; Alveolata; Apicomplexa; Conoidasida; Gregarinasina; Eugregarinorida; Elev-18S-1089 | probable_invert_parasite |
| denovo1711 | Eukaryota; Alveolata; Apicomplexa; Conoidasida; Gregarinasina; Eugregarinorida; Elev-18S-1089 | probable_invert_parasite |

| | | |
|---|---|---|
| denovo2026 | Eukaryota; Alveolata; Apicomplexa; Conoidasida; Gregarinasina; Eugregarinorida; Elev-18S-1089 | probable_invert_parasite |
| denovo688 | Eukaryota; Alveolata; Apicomplexa; Conoidasida; Gregarinasina; Eugregarinorida; Elev-18S-1089; uncultured_Eimeriidae | probable_invert_parasite |
| denovo473 | Eukaryota; Alveolata; Apicomplexa; Conoidasida; Gregarinasina; Eugregarinorida; Elev-18S-1089; uncultured_Eimeriidae | probable_invert_parasite |
| denovo449 | Eukaryota; Alveolata; Apicomplexa; Conoidasida; Gregarinasina; Eugregarinorida; Elev-18S-1089; uncultured_Eimeriidae | probable_invert_parasite |
| denovo341 | Eukaryota; Alveolata; Apicomplexa; Conoidasida; Gregarinasina; Eugregarinorida; Elev-18S-1089; uncultured_Eimeriidae | probable_invert_parasite |
| denovo249 | Eukaryota; Alveolata; Apicomplexa; Conoidasida; Gregarinasina; Eugregarinorida; Elev-18S-1089; uncultured_Eimeriidae | probable_invert_parasite |
| denovo247 | Eukaryota; Alveolata; Apicomplexa; Conoidasida; Gregarinasina; Eugregarinorida; Elev-18S-1089; uncultured_Eimeriidae | probable_invert_parasite |
| denovo1129 | Eukaryota; Alveolata; Apicomplexa; Conoidasida; Gregarinasina; Eugregarinorida; Elev-18S-1089; uncultured_Eimeriidae | probable_invert_parasite |
| denovo633 | Eukaryota; Alveolata; Apicomplexa; Conoidasida; Gregarinasina; Eugregarinorida; Gregarina | probable_invert_parasite |
| denovo1221 | Eukaryota; Alveolata; Apicomplexa; Conoidasida; Gregarinasina; Eugregarinorida; Gregarina | probable_invert_parasite |
| denovo1327 | Eukaryota; Alveolata; Apicomplexa; Conoidasida; Gregarinasina; Eugregarinorida; Gregarina | probable_invert_parasite |
| denovo88 | Eukaryota; Alveolata; Apicomplexa; Conoidasida; Gregarinasina; Eugregarinorida; Gregarina; Heterocapsaceae_environmental_sample | probable_invert_parasite |
| denovo429 | Eukaryota; Alveolata; Apicomplexa; Conoidasida; Gregarinasina; Eugregarinorida; Gregarina; Heterocapsaceae_environmental_sample | probable_invert_parasite |
| denovo387 | Eukaryota; Alveolata; Apicomplexa; Conoidasida; Gregarinasina; Eugregarinorida; Gregarina; Heterocapsaceae_environmental_sample | probable_invert_parasite |
| denovo3221 | Eukaryota; Alveolata; Apicomplexa; Conoidasida; Gregarinasina; Eugregarinorida; Gregarina; Heterocapsaceae_environmental_sample | probable_invert_parasite |
| denovo3031 | Eukaryota; Alveolata; Apicomplexa; Conoidasida; Gregarinasina; Eugregarinorida; Gregarina; Heterocapsaceae_environmental_sample | probable_invert_parasite |
| denovo2983 | Eukaryota; Alveolata; Apicomplexa; Conoidasida; Gregarinasina; Eugregarinorida; Gregarina; Heterocapsaceae_environmental_sample | probable_invert_parasite |
| denovo2721 | Eukaryota; Alveolata; Apicomplexa; Conoidasida; Gregarinasina; Eugregarinorida; Gregarina; Heterocapsaceae_environmental_sample | probable_invert_parasite |
| denovo2639 | Eukaryota; Alveolata; Apicomplexa; Conoidasida; Gregarinasina; Eugregarinorida; Gregarina; Heterocapsaceae_environmental_sample | probable_invert_parasite |
| denovo12 | Eukaryota; Alveolata; Apicomplexa; Conoidasida; Gregarinasina; Eugregarinorida; Gregarina; Heterocapsaceae_environmental_sample | probable_invert_parasite |
| denovo1389 | Eukaryota; Alveolata; Apicomplexa; Conoidasida; Gregarinasina; Eugregarinorida; Gregarina; Heterocapsaceae_environmental_sample | probable_invert_parasite |
| denovo1883 | Eukaryota; Alveolata; Apicomplexa; Conoidasida; Gregarinasina; Eugregarinorida; Gregarina; Heterocapsaceae_environmental_sample | probable_invert_parasite |
| denovo1857 | Eukaryota; Alveolata; Apicomplexa; Conoidasida; Gregarinasina; Eugregarinorida; Gregarina; Heterocapsaceae_environmental_sample | probable_invert_parasite |
| denovo1838 | Eukaryota; Alveolata; Apicomplexa; Conoidasida; Gregarinasina; Eugregarinorida; Gregarina; Heterocapsaceae_environmental_sample | probable_invert_parasite |
| denovo1810 | Eukaryota; Alveolata; Apicomplexa; Conoidasida; Gregarinasina; Eugregarinorida; Gregarina; Heterocapsaceae_environmental_sample | probable_invert_parasite |
| denovo2123 | Eukaryota; Alveolata; Apicomplexa; Conoidasida; Gregarinasina; Eugregarinorida; Gregarina; Heterocapsaceae_environmental_sample | probable_invert_parasite |
| denovo453 | Eukaryota; Alveolata; Apicomplexa; Conoidasida; Gregarinasina; Eugregarinorida; Gregarinidae; Amoebogregarina; Amoebogregarina_nigra | invert_parasite |
| denovo3217 | Eukaryota; Alveolata; Apicomplexa; Conoidasida; Gregarinasina; Eugregarinorida; Gregarinidae; Amoebogregarina; Amoebogregarina_nigra | invert_parasite |
| denovo2405 | Eukaryota; Alveolata; Apicomplexa; Conoidasida; Gregarinasina; Eugregarinorida; Gregarinidae; Amoebogregarina; Amoebogregarina_nigra | invert_parasite |
| denovo1189 | Eukaryota; Alveolata; Apicomplexa; Conoidasida; Gregarinasina; Eugregarinorida; Gregarinidae; Amoebogregarina; Amoebogregarina_nigra | invert_parasite |
| denovo540 | Eukaryota; Alveolata; Apicomplexa; Conoidasida; Gregarinasina; Eugregarinorida; Gregarinidae; Gregarina | invert_parasite |
| denovo2520 | Eukaryota; Alveolata; Apicomplexa; Conoidasida; Gregarinasina; Eugregarinorida; Gregarinidae; Gregarina | invert_parasite |
| denovo2340 | Eukaryota; Alveolata; Apicomplexa; Conoidasida; Gregarinasina; Eugregarinorida; Gregarinidae; Gregarina | invert_parasite |
| denovo1726 | Eukaryota; Alveolata; Apicomplexa; Conoidasida; Gregarinasina; Eugregarinorida; Gregarinidae; Gregarina | invert_parasite |
| denovo690 | Eukaryota; Alveolata; Apicomplexa; Conoidasida; Gregarinasina; Eugregarinorida; Gregarinidae; Gregarina; Gregarina_kingi | invert_parasite |
| denovo640 | Eukaryota; Alveolata; Apicomplexa; Conoidasida; Gregarinasina; Eugregarinorida; Gregarinidae; Gregarina; Gregarina_kingi | invert_parasite |
| denovo2715 | Eukaryota; Alveolata; Apicomplexa; Conoidasida; Gregarinasina; Eugregarinorida; Gregarinidae; Gregarina; Gregarina_kingi | invert_parasite |
| denovo1666 | Eukaryota; Alveolata; Apicomplexa; Conoidasida; Gregarinasina; Eugregarinorida; Gregarinidae; Gregarina; Gregarina_kingi | invert_parasite |
| denovo794 | Eukaryota; Alveolata; Apicomplexa; Conoidasida; Gregarinasina; Eugregarinorida; Paraschneideria | invert_parasite |
| denovo599 | Eukaryota; Alveolata; Apicomplexa; Conoidasida; Gregarinasina; Eugregarinorida; Paraschneideria | invert_parasite |

| | | |
|---|---|---|
| denovo2695 | Eukaryota; Alveolata; Apicomplexa; Conoidasida; Gregarinasina; Eugregarinorida; Paraschneideria | invert_parasite |
| denovo257 | Eukaryota; Alveolata; Apicomplexa; Conoidasida; Gregarinasina; Eugregarinorida; Paraschneideria | invert_parasite |
| denovo301 | Eukaryota; Alveolata; Apicomplexa; Conoidasida; Gregarinasina; Eugregarinorida; Stenophora; Stenophora_robusta | invert_parasite |
| denovo267 | Eukaryota; Alveolata; Apicomplexa; Conoidasida; Gregarinasina; Eugregarinorida; Stenophora; Stenophora_robusta | invert_parasite |
| denovo3207 | Eukaryota; Alveolata; Apicomplexa; Conoidasida; Gregarinasina; Eugregarinorida; uncultured | invert_parasite |
| denovo504 | Eukaryota; Alveolata; Apicomplexa; Conoidasida; Gregarinasina; Neogregarinorida; Mattesia | invert_parasite |
| denovo2290 | Eukaryota; Alveolata; Apicomplexa; Conoidasida; Gregarinasina; Neogregarinorida; Ophryocystis; Apicomplexan_Acarus_siro_new_pathogen | invert_parasite |
| denovo1926 | Eukaryota; Alveolata; Ciliophora; Conthreep; Colpodea; Bursariomorphida; Bryometopus | environmental |
| denovo613 | Eukaryota; Alveolata; Ciliophora; Conthreep; Colpodea; Colpodida | environmental |
| denovo2898 | Eukaryota; Alveolata; Ciliophora; Conthreep; Colpodea; Colpodida | environmental |
| denovo2662 | Eukaryota; Alveolata; Ciliophora; Conthreep; Colpodea; Colpodida | environmental |
| denovo2512 | Eukaryota; Alveolata; Ciliophora; Conthreep; Colpodea; Colpodida | environmental |
| denovo2445 | Eukaryota; Alveolata; Ciliophora; Conthreep; Colpodea; Colpodida | environmental |
| denovo1510 | Eukaryota; Alveolata; Ciliophora; Conthreep; Colpodea; Colpodida | environmental |
| denovo2702 | Eukaryota; Alveolata; Ciliophora; Conthreep; Colpodea; Colpodida; Bresslauides | environmental |
| denovo1722 | Eukaryota; Alveolata; Ciliophora; Conthreep; Colpodea; Colpodida; Bresslauides | environmental |
| denovo379 | Eukaryota; Alveolata; Ciliophora; Conthreep; Colpodea; Colpodida; Bromeliothrix | environmental |
| denovo1366 | Eukaryota; Alveolata; Ciliophora; Conthreep; Colpodea; Colpodida; Bromeliothrix | environmental |
| denovo2815 | Eukaryota; Alveolata; Ciliophora; Conthreep; Colpodea; Colpodida; Colpoda | environmental |
| denovo2678 | Eukaryota; Alveolata; Ciliophora; Conthreep; Colpodea; Colpodida; Colpoda | environmental |
| denovo2417 | Eukaryota; Alveolata; Ciliophora; Conthreep; Colpodea; Colpodida; Colpoda | environmental |
| denovo1026 | Eukaryota; Alveolata; Ciliophora; Conthreep; Colpodea; Colpodida; Colpoda | environmental |
| denovo1056 | Eukaryota; Alveolata; Ciliophora; Conthreep; Colpodea; Colpodida; Colpoda | environmental |
| denovo1300 | Eukaryota; Alveolata; Ciliophora; Conthreep; Colpodea; Colpodida; Colpoda | environmental |
| denovo1580 | Eukaryota; Alveolata; Ciliophora; Conthreep; Colpodea; Colpodida; Colpoda | environmental |
| denovo2202 | Eukaryota; Alveolata; Ciliophora; Conthreep; Colpodea; Colpodida; Colpoda | environmental |
| denovo886 | Eukaryota; Alveolata; Ciliophora; Conthreep; Colpodea; Colpodida; Colpoda; Colpoda_aspera | environmental |
| denovo800 | Eukaryota; Alveolata; Ciliophora; Conthreep; Colpodea; Colpodida; Colpoda; Colpoda_aspera | environmental |
| denovo1246 | Eukaryota; Alveolata; Ciliophora; Conthreep; Colpodea; Colpodida; Colpoda; Colpoda_aspera | environmental |
| denovo708 | Eukaryota; Alveolata; Ciliophora; Conthreep; Colpodea; Colpodida; Maryna | environmental |
| denovo3256 | Eukaryota; Alveolata; Ciliophora; Conthreep; Colpodea; Colpodida; Maryna | environmental |
| denovo2194 | Eukaryota; Alveolata; Ciliophora; Conthreep; Colpodea; Colpodida; Maryna | environmental |
| denovo896 | Eukaryota; Alveolata; Ciliophora; Conthreep; Colpodea; Colpodida; Pseudoplatyophyra | environmental |
| denovo667 | Eukaryota; Alveolata; Ciliophora; Conthreep; Colpodea; Colpodida; Pseudoplatyophyra | environmental |
| denovo2306 | Eukaryota; Alveolata; Ciliophora; Conthreep; Colpodea; Colpodida; Pseudoplatyophyra | environmental |
| denovo1073 | Eukaryota; Alveolata; Ciliophora; Conthreep; Colpodea; Colpodida; Pseudoplatyophyra | environmental |
| denovo1936 | Eukaryota; Alveolata; Ciliophora; Conthreep; Colpodea; Colpodida; Pseudoplatyophyra | environmental |
| denovo2869 | Eukaryota; Alveolata; Ciliophora; Conthreep; Colpodea; Cyrtolophosidida | environmental |
| denovo1481 | Eukaryota; Alveolata; Ciliophora; Conthreep; Colpodea; Cyrtolophosidida; Cyrtolophosis | environmental |
| denovo1645 | Eukaryota; Alveolata; Ciliophora; Conthreep; Colpodea; Cyrtolophosidida; Cyrtolophosis | environmental |

| | | |
|---|---|---|
| denovo1688 | Eukaryota; Alveolata; Ciliophora; Conthreep; Colpodea; Cyrtolophosidida; Cyrtolophosis | environmental |
| denovo675 | Eukaryota; Alveolata; Ciliophora; Conthreep; Colpodea; Platyophryida; Platyophrya | environmental |
| denovo535 | Eukaryota; Alveolata; Ciliophora; Conthreep; Colpodea; Platyophryida; Platyophrya | environmental |
| denovo359 | Eukaryota; Alveolata; Ciliophora; Conthreep; Colpodea; Platyophryida; Platyophrya | environmental |
| denovo2333 | Eukaryota; Alveolata; Ciliophora; Conthreep; Colpodea; Platyophryida; Platyophrya | environmental |
| denovo1021 | Eukaryota; Alveolata; Ciliophora; Conthreep; Colpodea; Platyophryida; Platyophrya | environmental |
| denovo1045 | Eukaryota; Alveolata; Ciliophora; Conthreep; Colpodea; Platyophryida; Platyophrya | environmental |
| denovo1586 | Eukaryota; Alveolata; Ciliophora; Conthreep; Colpodea; Platyophryida; Platyophrya | environmental |
| denovo2065 | Eukaryota; Alveolata; Ciliophora; Conthreep; Colpodea; Platyophryida; Platyophrya | environmental |
| denovo2261 | Eukaryota; Alveolata; Ciliophora; Conthreep; Colpodea; Platyophryida; Platyophrya | environmental |
| denovo1819 | Eukaryota; Alveolata; Ciliophora; Conthreep; Nassophorea | environmental |
| denovo1326 | Eukaryota; Alveolata; Ciliophora; Conthreep; Nassophorea; Leptopharynx; Leptopharynx | environmental |
| denovo789 | Eukaryota; Alveolata; Ciliophora; Conthreep; Oligohymenophorea; Peniculia; uncultured; uncultured_alveolate | environmental |
| denovo400 | Eukaryota; Alveolata; Ciliophora; Conthreep; Oligohymenophorea; Peritrichia; Pseudovorticella; uncultured_eukaryote | environmental |
| denovo959 | Eukaryota; Alveolata; Ciliophora; Conthreep; Oligohymenophorea; Peritrichia; uncultured | environmental |
| denovo1435 | Eukaryota; Alveolata; Ciliophora; Conthreep; Oligohymenophorea; Peritrichia; Vorticella | environmental |
| denovo1887 | Eukaryota; Alveolata; Ciliophora; Conthreep; Oligohymenophorea; Scuticociliatia | environmental |
| denovo2025 | Eukaryota; Alveolata; Ciliophora; Conthreep; Oligohymenophorea; Scuticociliatia | environmental |
| denovo3239 | Eukaryota; Alveolata; Ciliophora; Conthreep; Oligohymenophorea; Scuticociliatia; Cyclidium; Cyclidium_glaucoma | environmental |
| denovo2181 | Eukaryota; Alveolata; Ciliophora; Conthreep; Oligohymenophorea; Scuticociliatia; Homalogastra | environmental |
| denovo790 | Eukaryota; Alveolata; Ciliophora; Litostomatea; Haptoria | environmental |
| denovo431 | Eukaryota; Alveolata; Ciliophora; Litostomatea; Haptoria | environmental |
| denovo402 | Eukaryota; Alveolata; Ciliophora; Litostomatea; Haptoria | environmental |
| denovo2861 | Eukaryota; Alveolata; Ciliophora; Litostomatea; Haptoria | environmental |
| denovo2748 | Eukaryota; Alveolata; Ciliophora; Litostomatea; Haptoria | environmental |
| denovo1061 | Eukaryota; Alveolata; Ciliophora; Litostomatea; Haptoria; Arcuosnew; Arcuosnew | environmental |
| denovo2897 | Eukaryota; Alveolata; Ciliophora; Litostomatea; Haptoria; Snew; Snew | environmental |
| denovo2853 | Eukaryota; Alveolata; Ciliophora; Litostomatea; Haptoria; Snew; Snew | environmental |
| denovo1597 | Eukaryota; Alveolata; Ciliophora; Litostomatea; Trichostomatia; Troglodytella; Troglodytella; Troglodytella_abrassarti | gut_resident |
| denovo250 | Eukaryota; Alveolata; Ciliophora; Litostomatea; uncultured | environmental |
| denovo721 | Eukaryota; Alveolata; Ciliophora; Spirotrichea; Hypotrichia | environmental |
| denovo498 | Eukaryota; Alveolata; Ciliophora; Spirotrichea; Hypotrichia | environmental |
| denovo3051 | Eukaryota; Alveolata; Ciliophora; Spirotrichea; Hypotrichia | environmental |
| denovo2693 | Eukaryota; Alveolata; Ciliophora; Spirotrichea; Hypotrichia | environmental |
| denovo2557 | Eukaryota; Alveolata; Ciliophora; Spirotrichea; Hypotrichia | environmental |
| denovo2525 | Eukaryota; Alveolata; Ciliophora; Spirotrichea; Hypotrichia; Bergeriella; Bergeriella | environmental |
| denovo3037 | Eukaryota; Alveolata; Ciliophora; Spirotrichea; Hypotrichia; Gonostomum; Gonostomum; uncultured_Oxytrichidae | environmental |
| denovo2670 | Eukaryota; Alveolata; Ciliophora; Spirotrichea; Hypotrichia; Halteria; Halteria | environmental |
| denovo1134 | Eukaryota; Alveolata; Ciliophora; Spirotrichea; Hypotrichia; Holosticha; Holosticha | environmental |

| | | |
|---|---|---|
| denovo1508 | Eukaryota; Alveolata; Ciliophora; Spirotrichea; Hypotrichia; Holosticha; Holosticha | environmental |
| denovo430 | Eukaryota; Alveolata; Ciliophora; Spirotrichea; Hypotrichia; Pseudourostyla; Pseudourostyla | environmental |
| denovo3269 | Eukaryota; Alveolata; Ciliophora; Spirotrichea; Hypotrichia; Pseudourostyla; Pseudourostyla; Pseudourostyla_cristata | environmental |
| denovo2438 | Eukaryota; Alveolata; Ciliophora; Spirotrichea; Hypotrichia; Pseudourostyla; Pseudourostyla; Pseudourostyla_cristata | environmental |
| denovo3273 | Eukaryota; Alveolata; Ciliophora; Spirotrichea; Hypotrichia; uncultured; uncultured; uncultured_Oxytrichidae | environmental |
| denovo2598 | Eukaryota; Alveolata; Ciliophora; Spirotrichea; Hypotrichia; Uroleptus; Uroleptus | environmental |
| denovo1948 | Eukaryota; Alveolata; Ciliophora; Spirotrichea; Hypotrichia; Urostyla; Urostyla | environmental |
| denovo2712 | Eukaryota; Alveolata; Ciliophora; Spirotrichea; uncultured; uncultured; uncultured | environmental |
| denovo2214 | Eukaryota; Alveolata; Ciliophora; Spirotrichea; uncultured; uncultured; uncultured | environmental |
| denovo1320 | Eukaryota; Alveolata; Colpodellidae; Colpodella | environmental |
| denovo1382 | Eukaryota; Alveolata; Dinoflagellata; Dinophyceae; Gymnodiniphycidae; Gymnodinium_clade; Spiniferodinium | environmental |
| denovo1252 | Eukaryota; Alveolata; Dinoflagellata; Dinophyceae; Gymnodiniphycidae; Gyrodinium; Gyrodinium; uncultured_eukaryote | environmental |
| denovo3314 | Eukaryota; Alveolata; Dinophyceae; Peridiniales; Heterocapsaceae; environmental_samples_<Heterocapsaceae>; Heterocapsaceae_environmental_sample | environmental |
| denovo2163 | Eukaryota; Alveolata; Dinophyceae; Peridiniales; Heterocapsaceae; environmental_samples_<Heterocapsaceae>; Heterocapsaceae_environmental_sample | environmental |
| denovo1687 | Eukaryota; Alveolata; Protalveolata; Colpodellida; Colpodella; Colpodella; Colpodella; uncultured_eukaryote | environmental |
| denovo589 | Eukaryota; Amoebozoa | unknown |
| denovo3203 | Eukaryota; Amoebozoa | unknown |
| denovo3084 | Eukaryota; Amoebozoa | unknown |
| denovo2784 | Eukaryota; Amoebozoa | unknown |
| denovo2530 | Eukaryota; Amoebozoa | unknown |
| denovo1709 | Eukaryota; Amoebozoa | unknown |
| denovo903 | Eukaryota; Amoebozoa; Archamoebae; Entamoeba; Entamoeba; Entamoeba; Entamoeba; Entamoeba_coli | gut_resident |
| denovo726 | Eukaryota; Amoebozoa; Archamoebae; Entamoeba; Entamoeba; Entamoeba; Entamoeba; Entamoeba_coli | gut_resident |
| denovo1973 | Eukaryota; Amoebozoa; Archamoebae; Entamoeba; Entamoeba; Entamoeba; Entamoeba; Entamoeba_coli | gut_resident |
| denovo2473 | Eukaryota; Amoebozoa; Archamoebae; Entamoeba; Entamoeba; Entamoeba; Entamoeba; Entamoeba_coli | gut_resident |
| denovo1706 | Eukaryota; Amoebozoa; Archamoebae; Entamoeba; Entamoeba; Entamoeba; Entamoeba; Entamoeba_coli | gut_resident |
| denovo1843 | Eukaryota; Amoebozoa; Archamoebae; Entamoeba; Entamoeba; Entamoeba; Entamoeba; Entamoeba_coli | gut_resident |
| denovo282 | Eukaryota; Amoebozoa; Archamoebae; Entamoeba; Entamoeba; Entamoeba; Entamoeba; Entamoeba_sp. | gut_resident |
| denovo99 | Eukaryota; Amoebozoa; Archamoebae; Entamoeba; Entamoeba; Entamoeba; Entamoeba; Entamoeba_sp. | gut_resident |
| denovo444 | Eukaryota; Amoebozoa; Archamoebae; Entamoeba; Entamoeba; Entamoeba; Entamoeba; Entamoeba_sp. | gut_resident |
| denovo124 | Eukaryota; Amoebozoa; Archamoebae; Entamoeba; Entamoeba; Entamoeba; Entamoeba; Entamoeba_sp. | gut_resident |
| denovo142 | Eukaryota; Amoebozoa; Archamoebae; Entamoeba; Entamoeba; Entamoeba; Entamoeba; Entamoeba_sp. | gut_resident |
| denovo149 | Eukaryota; Amoebozoa; Archamoebae; Entamoeba; Entamoeba; Entamoeba; Entamoeba; Entamoeba_sp. | gut_resident |
| denovo186 | Eukaryota; Amoebozoa; Archamoebae; Entamoeba; Entamoeba; Entamoeba; Entamoeba; Entamoeba_sp. | gut_resident |
| denovo150 | Eukaryota; Amoebozoa; Archamoebae; Entamoeba; Entamoeba; Entamoeba; Entamoeba; Entamoeba_coli | gut_resident |
| denovo369 | Eukaryota; Amoebozoa; Archamoebae; Entamoeba; Entamoeba; Entamoeba; Entamoeba; Entamoeba_sp. | gut_resident |
| denovo132 | Eukaryota; Amoebozoa; Archamoebae; Entamoeba; Entamoeba; Entamoeba; Entamoeba; Entamoeba_sp. | gut_resident |
| denovo157 | Eukaryota; Amoebozoa; Archamoebae; Entamoeba; Entamoeba; Entamoeba; Entamoeba; Entamoeba_sp. | gut_resident |
| denovo159 | Eukaryota; Amoebozoa; Archamoebae; Entamoeba; Entamoeba; Entamoeba; Entamoeba; Entamoeba_sp. | gut_resident |
| denovo65 | Eukaryota; Amoebozoa; Archamoebae; Entamoeba; Entamoeba; Entamoeba; Entamoeba | gut_resident |

| | | |
|---|---|---|
| denovo651 | Eukaryota; Amoebozoa; Archamoebae; Entamoeba; Entamoeba; Entamoeba; Entamoeba; Entamoeba_coli | gut_resident |
| denovo2070 | Eukaryota; Amoebozoa; Archamoebae; Entamoeba; Entamoeba; Entamoeba; Entamoeba; Entamoeba_coli | gut_resident |
| denovo594 | Eukaryota; Amoebozoa; Archamoebae; Entamoeba; Entamoeba; Entamoeba; Entamoeba; Entamoeba_sp. | gut_resident |
| denovo374 | Eukaryota; Amoebozoa; Archamoebae; Entamoeba; Entamoeba; Entamoeba; Entamoeba; Entamoeba_sp. | gut_resident |
| denovo1031 | Eukaryota; Amoebozoa; Archamoebae; Entamoeba; Entamoeba; Entamoeba; Entamoeba; Entamoeba_sp. | gut_resident |
| denovo1037 | Eukaryota; Amoebozoa; Archamoebae; Entamoeba; Entamoeba; Entamoeba; Entamoeba; Entamoeba_sp. | gut_resident |
| denovo658 | Eukaryota; Amoebozoa; Archamoebae; Entamoebidae; Endolimax; Endolimax_nana | gut_resident |
| denovo3097 | Eukaryota; Amoebozoa; Archamoebae; Entamoebidae; Endolimax; Endolimax_nana | gut_resident |
| denovo2764 | Eukaryota; Amoebozoa; Archamoebae; Entamoebidae; Endolimax; Endolimax_nana | gut_resident |
| denovo2523 | Eukaryota; Amoebozoa; Archamoebae; Entamoebidae; Endolimax; Endolimax_nana | gut_resident |
| denovo2507 | Eukaryota; Amoebozoa; Archamoebae; Entamoebidae; Endolimax; Endolimax_nana | gut_resident |
| denovo216 | Eukaryota; Amoebozoa; Archamoebae; Entamoebidae; Endolimax; Endolimax_nana | gut_resident |
| denovo1088 | Eukaryota; Amoebozoa; Archamoebae; Entamoebidae; Endolimax; Endolimax_nana | gut_resident |
| denovo1200 | Eukaryota; Amoebozoa; Archamoebae; Entamoebidae; Endolimax; Endolimax_nana | gut_resident |
| denovo1940 | Eukaryota; Amoebozoa; Archamoebae; Entamoebidae; Endolimax; Endolimax_nana | gut_resident |
| denovo1917 | Eukaryota; Amoebozoa; Archamoebae; Entamoebidae; Endolimax; Endolimax_nana | gut_resident |
| denovo1891 | Eukaryota; Amoebozoa; Archamoebae; Entamoebidae; Endolimax; Endolimax_nana | gut_resident |
| denovo1959 | Eukaryota; Amoebozoa; Archamoebae; Entamoebidae; Endolimax; Endolimax_nana | gut_resident |
| denovo1958 | Eukaryota; Amoebozoa; Archamoebae; Entamoebidae; Endolimax; Endolimax_nana | gut_resident |
| denovo2241 | Eukaryota; Amoebozoa; Archamoebae; Entamoebidae; Endolimax; Endolimax_nana | gut_resident |
| denovo2235 | Eukaryota; Amoebozoa; Archamoebae; Entamoebidae; Endolimax; Endolimax_nana | gut_resident |
| denovo1365 | Eukaryota; Amoebozoa; Archamoebae; Entamoeba; Entamoeba; Entamoeba; Entamoeba; Entamoeba_coli | gut_resident |
| denovo616 | Eukaryota; Amoebozoa; Archamoebae; Entamoeba; Entamoeba; Entamoeba; Entamoeba; Entamoeba_sp. | gut_resident |
| denovo2789 | Eukaryota; Amoebozoa; Archamoebae; Entamoebidae; Endolimax; Endolimax_nana | gut_resident |
| denovo100 | Eukaryota; Amoebozoa; Archamoebae; Entamoeba; Entamoeba; Entamoeba; Entamoeba; Entamoeba_sp. | gut_resident |
| denovo1622 | Eukaryota; Amoebozoa; Archamoebae; Entamoeba; Entamoeba; Entamoeba; Entamoeba; Entamoeba_coli | gut_resident |
| denovo488 | Eukaryota; Amoebozoa; Archamoebae; Entamoeba; Entamoeba; Entamoeba; Entamoeba; Entamoeba_sp. | gut_resident |
| denovo352 | Eukaryota; Amoebozoa; Archamoebae; Entamoeba; Entamoeba; Entamoeba; Entamoeba; Entamoeba_sp. | gut_resident |
| denovo994 | Eukaryota; Amoebozoa; Archamoebae; Entamoebidae; Endolimax; Endolimax_nana | gut_resident |
| denovo3146 | Eukaryota; Amoebozoa; Archamoebae; Entamoebidae; Endolimax; Endolimax_nana | gut_resident |
| denovo3109 | Eukaryota; Amoebozoa; Archamoebae; Entamoebidae; Endolimax; Endolimax_nana | gut_resident |
| denovo2772 | Eukaryota; Amoebozoa; Archamoebae; Entamoebidae; Endolimax; Endolimax_nana | gut_resident |
| denovo2750 | Eukaryota; Amoebozoa; Archamoebae; Entamoebidae; Endolimax; Endolimax_nana | gut_resident |
| denovo2608 | Eukaryota; Amoebozoa; Archamoebae; Entamoebidae; Endolimax; Endolimax_nana | gut_resident |
| denovo2476 | Eukaryota; Amoebozoa; Archamoebae; Entamoebidae; Endolimax; Endolimax_nana | gut_resident |
| denovo2463 | Eukaryota; Amoebozoa; Archamoebae; Entamoebidae; Endolimax; Endolimax_nana | gut_resident |
| denovo2454 | Eukaryota; Amoebozoa; Archamoebae; Entamoebidae; Endolimax; Endolimax_nana | gut_resident |
| denovo2342 | Eukaryota; Amoebozoa; Archamoebae; Entamoebidae; Endolimax; Endolimax_nana | gut_resident |
| denovo1237 | Eukaryota; Amoebozoa; Archamoebae; Entamoebidae; Endolimax; Endolimax_nana | gut_resident |
| denovo1256 | Eukaryota; Amoebozoa; Archamoebae; Entamoebidae; Endolimax; Endolimax_nana | gut_resident |

| | | |
|---|---|---|
| denovo1773 | Eukaryota; Amoebozoa; Archamoebae; Entamoebidae; Endolimax; Endolimax_nana | gut_resident |
| denovo1907 | Eukaryota; Amoebozoa; Archamoebae; Entamoebidae; Endolimax; Endolimax_nana | gut_resident |
| denovo2051 | Eukaryota; Amoebozoa; Archamoebae; Entamoebidae; Endolimax; Endolimax_nana | gut_resident |
| denovo1783 | Eukaryota; Amoebozoa; Archamoebae; Entamoeba; Entamoeba; Entamoeba; Entamoeba; Entamoeba_coli | gut_resident |
| denovo483 | Eukaryota; Amoebozoa; Archamoebae; Entamoebidae; Endolimax; Endolimax_nana | gut_resident |
| denovo1279 | Eukaryota; Amoebozoa; Archamoebae; Entamoebidae; Endolimax; Endolimax_nana | gut_resident |
| denovo148 | Eukaryota; Amoebozoa; Archamoebae; Entamoeba; Entamoeba; Entamoeba; Entamoeba; Entamoeba_coli | gut_resident |
| denovo399 | Eukaryota; Amoebozoa; Archamoebae; Entamoeba; Entamoeba; Entamoeba; Entamoeba; Entamoeba_sp. | gut_resident |
| denovo258 | Eukaryota; Amoebozoa; Archamoebae; Entamoeba; Entamoeba; Entamoeba; Entamoeba; Entamoeba_sp. | gut_resident |
| denovo2089 | Eukaryota; Amoebozoa; Archamoebae; Entamoeba; Entamoeba; Entamoeba; Entamoeba; Entamoeba_sp. | gut_resident |
| denovo48 | Eukaryota; Amoebozoa; Archamoebae; Entamoeba; Entamoeba; Entamoeba; Entamoeba; Entamoeba_coli | gut_resident |
| denovo2131 | Eukaryota; Amoebozoa; Archamoebae; Entamoeba; Entamoeba; Entamoeba; Entamoeba; Entamoeba_sp. | gut_resident |
| denovo2423 | Eukaryota; Amoebozoa; Archamoebae; Entamoebidae; Entamoeba; Entamoeba_sp. | gut_resident |
| denovo183 | Eukaryota; Amoebozoa; Archamoebae; Entamoeba; Entamoeba; Entamoeba; Entamoeba; Entamoeba_sp. | gut_resident |
| denovo307 | Eukaryota; Amoebozoa; Archamoebae; Entamoeba; Entamoeba; Entamoeba; Entamoeba; Entamoeba_sp. | gut_resident |
| denovo1180 | Eukaryota; Amoebozoa; Archamoebae; Entamoeba; Entamoeba; Entamoeba; Entamoeba; Entamoeba_sp. | gut_resident |
| denovo364 | Eukaryota; Amoebozoa; Archamoebae; Entamoeba; Entamoeba; Entamoeba; Entamoeba; Entamoeba_sp. | gut_resident |
| denovo351 | Eukaryota; Amoebozoa; Archamoebae; Entamoeba; Entamoeba; Entamoeba; Entamoeba; Entamoeba_sp. | gut_resident |
| denovo768 | Eukaryota; Amoebozoa; Archamoebae; Entamoebidae; Entamoeba | gut_resident |
| denovo831 | Eukaryota; Amoebozoa; Archamoebae; Entamoeba; Entamoeba; Entamoeba; Entamoeba; Entamoeba_coli | gut_resident |
| denovo2308 | Eukaryota; Amoebozoa; Archamoebae; Entamoebidae; Entamoeba | gut_resident |
| denovo1715 | Eukaryota; Amoebozoa; Archamoebae; Entamoebidae; Entamoeba | gut_resident |
| denovo2053 | Eukaryota; Amoebozoa; Archamoebae; Entamoebidae; Entamoeba | gut_resident |
| denovo102 | Eukaryota; Amoebozoa; Archamoebae; Entamoeba; Entamoeba; Entamoeba; Entamoeba; Entamoeba_sp. | gut_resident |
| denovo96 | Eukaryota; Amoebozoa; Archamoebae; Entamoeba; Entamoeba; Entamoeba; Entamoeba; Entamoeba_coli | gut_resident |
| denovo235 | Eukaryota; Amoebozoa; Archamoebae; Entamoebidae; environmental_samples_<Entamoebidae>; uncultured_Entamoeba | gut_resident |
| denovo83 | Eukaryota; Amoebozoa; Archamoebae; Entamoebidae; Entamoeba; Entamoeba_sp. | gut_resident |
| denovo999 | Eukaryota; Amoebozoa; Archamoebae; Entamoebidae; Iodamoeba; Iodamoeba_sp._RL1 | gut_resident |
| denovo997 | Eukaryota; Amoebozoa; Archamoebae; Entamoebidae; Iodamoeba; Iodamoeba_sp._RL1 | gut_resident |
| denovo995 | Eukaryota; Amoebozoa; Archamoebae; Entamoebidae; Iodamoeba; Iodamoeba_sp._RL1 | gut_resident |
| denovo993 | Eukaryota; Amoebozoa; Archamoebae; Entamoebidae; Iodamoeba; Iodamoeba_sp._RL1 | gut_resident |
| denovo983 | Eukaryota; Amoebozoa; Archamoebae; Entamoebidae; Iodamoeba; Iodamoeba_sp._RL1 | gut_resident |
| denovo980 | Eukaryota; Amoebozoa; Archamoebae; Entamoebidae; Iodamoeba; Iodamoeba_sp._RL1 | gut_resident |
| denovo970 | Eukaryota; Amoebozoa; Archamoebae; Entamoebidae; Iodamoeba; Iodamoeba_sp._RL1 | gut_resident |
| denovo966 | Eukaryota; Amoebozoa; Archamoebae; Entamoebidae; Iodamoeba; Iodamoeba_sp._RL1 | gut_resident |
| denovo964 | Eukaryota; Amoebozoa; Archamoebae; Entamoebidae; Iodamoeba; Iodamoeba_sp._RL1 | gut_resident |
| denovo948 | Eukaryota; Amoebozoa; Archamoebae; Entamoebidae; Iodamoeba; Iodamoeba_sp._RL1 | gut_resident |
| denovo947 | Eukaryota; Amoebozoa; Archamoebae; Entamoebidae; Iodamoeba; Iodamoeba_sp._RL1 | gut_resident |
| denovo946 | Eukaryota; Amoebozoa; Archamoebae; Entamoebidae; Iodamoeba; Iodamoeba_sp._RL1 | gut_resident |
| denovo945 | Eukaryota; Amoebozoa; Archamoebae; Entamoebidae; Iodamoeba; Iodamoeba_sp._RL1 | gut_resident |

| | | |
|---|---|---|
| denovo944 | Eukaryota; Amoebozoa; Archamoebae; Entamoebidae; Iodamoeba; Iodamoeba_sp._RL1 | gut_resident |
| denovo937 | Eukaryota; Amoebozoa; Archamoebae; Entamoebidae; Iodamoeba; Iodamoeba_sp._RL1 | gut_resident |
| denovo929 | Eukaryota; Amoebozoa; Archamoebae; Entamoebidae; Iodamoeba; Iodamoeba_sp._RL1 | gut_resident |
| denovo926 | Eukaryota; Amoebozoa; Archamoebae; Entamoebidae; Iodamoeba; Iodamoeba_sp._RL1 | gut_resident |
| denovo915 | Eukaryota; Amoebozoa; Archamoebae; Entamoebidae; Iodamoeba; Iodamoeba_sp._RL1 | gut_resident |
| denovo914 | Eukaryota; Amoebozoa; Archamoebae; Entamoebidae; Iodamoeba; Iodamoeba_sp._RL1 | gut_resident |
| denovo913 | Eukaryota; Amoebozoa; Archamoebae; Entamoebidae; Iodamoeba; Iodamoeba_sp._RL1 | gut_resident |
| denovo909 | Eukaryota; Amoebozoa; Archamoebae; Entamoebidae; Iodamoeba; Iodamoeba_sp._RL1 | gut_resident |
| denovo906 | Eukaryota; Amoebozoa; Archamoebae; Entamoebidae; Iodamoeba; Iodamoeba_sp._RL1 | gut_resident |
| denovo905 | Eukaryota; Amoebozoa; Archamoebae; Entamoebidae; Iodamoeba; Iodamoeba_sp._RL1 | gut_resident |
| denovo904 | Eukaryota; Amoebozoa; Archamoebae; Entamoebidae; Iodamoeba; Iodamoeba_sp._RL1 | gut_resident |
| denovo900 | Eukaryota; Amoebozoa; Archamoebae; Entamoebidae; Iodamoeba; Iodamoeba_sp._RL1 | gut_resident |
| denovo899 | Eukaryota; Amoebozoa; Archamoebae; Entamoebidae; Iodamoeba; Iodamoeba_sp._RL1 | gut_resident |
| denovo890 | Eukaryota; Amoebozoa; Archamoebae; Entamoebidae; Iodamoeba; Iodamoeba_sp._RL1 | gut_resident |
| denovo888 | Eukaryota; Amoebozoa; Archamoebae; Entamoebidae; Iodamoeba; Iodamoeba_sp._RL1 | gut_resident |
| denovo883 | Eukaryota; Amoebozoa; Archamoebae; Entamoebidae; Iodamoeba; Iodamoeba_sp._RL1 | gut_resident |
| denovo880 | Eukaryota; Amoebozoa; Archamoebae; Entamoebidae; Iodamoeba; Iodamoeba_sp._RL1 | gut_resident |
| denovo879 | Eukaryota; Amoebozoa; Archamoebae; Entamoebidae; Iodamoeba; Iodamoeba_sp._RL1 | gut_resident |
| denovo877 | Eukaryota; Amoebozoa; Archamoebae; Entamoebidae; Iodamoeba; Iodamoeba_sp._RL1 | gut_resident |
| denovo875 | Eukaryota; Amoebozoa; Archamoebae; Entamoebidae; Iodamoeba; Iodamoeba_sp._RL1 | gut_resident |
| denovo870 | Eukaryota; Amoebozoa; Archamoebae; Entamoebidae; Iodamoeba; Iodamoeba_sp._RL1 | gut_resident |
| denovo867 | Eukaryota; Amoebozoa; Archamoebae; Entamoebidae; Iodamoeba; Iodamoeba_sp._RL1 | gut_resident |
| denovo866 | Eukaryota; Amoebozoa; Archamoebae; Entamoebidae; Iodamoeba; Iodamoeba_sp._RL1 | gut_resident |
| denovo863 | Eukaryota; Amoebozoa; Archamoebae; Entamoebidae; Iodamoeba; Iodamoeba_sp._RL1 | gut_resident |
| denovo860 | Eukaryota; Amoebozoa; Archamoebae; Entamoebidae; Iodamoeba; Iodamoeba_sp._RL1 | gut_resident |
| denovo856 | Eukaryota; Amoebozoa; Archamoebae; Entamoebidae; Iodamoeba; Iodamoeba_sp._RL1 | gut_resident |
| denovo855 | Eukaryota; Amoebozoa; Archamoebae; Entamoebidae; Iodamoeba; Iodamoeba_sp._RL1 | gut_resident |
| denovo829 | Eukaryota; Amoebozoa; Archamoebae; Entamoebidae; Iodamoeba; Iodamoeba_sp._RL1 | gut_resident |
| denovo824 | Eukaryota; Amoebozoa; Archamoebae; Entamoebidae; Iodamoeba; Iodamoeba_sp._RL1 | gut_resident |
| denovo822 | Eukaryota; Amoebozoa; Archamoebae; Entamoebidae; Iodamoeba; Iodamoeba_sp._RL1 | gut_resident |
| denovo820 | Eukaryota; Amoebozoa; Archamoebae; Entamoebidae; Iodamoeba; Iodamoeba_sp._RL1 | gut_resident |
| denovo819 | Eukaryota; Amoebozoa; Archamoebae; Entamoebidae; Iodamoeba; Iodamoeba_sp._RL1 | gut_resident |
| denovo812 | Eukaryota; Amoebozoa; Archamoebae; Entamoebidae; Iodamoeba; Iodamoeba_sp._RL1 | gut_resident |
| denovo805 | Eukaryota; Amoebozoa; Archamoebae; Entamoebidae; Iodamoeba; Iodamoeba_sp._RL1 | gut_resident |
| denovo804 | Eukaryota; Amoebozoa; Archamoebae; Entamoebidae; Iodamoeba; Iodamoeba_sp._RL1 | gut_resident |
| denovo799 | Eukaryota; Amoebozoa; Archamoebae; Entamoebidae; Iodamoeba; Iodamoeba_sp._RL1 | gut_resident |
| denovo798 | Eukaryota; Amoebozoa; Archamoebae; Entamoebidae; Iodamoeba; Iodamoeba_sp._RL1 | gut_resident |
| denovo796 | Eukaryota; Amoebozoa; Archamoebae; Entamoebidae; Iodamoeba; Iodamoeba_sp._RL1 | gut_resident |
| denovo795 | Eukaryota; Amoebozoa; Archamoebae; Entamoebidae; Iodamoeba; Iodamoeba_sp._RL1 | gut_resident |
| denovo792 | Eukaryota; Amoebozoa; Archamoebae; Entamoebidae; Iodamoeba; Iodamoeba_sp._RL1 | gut_resident |

| | | |
|---|---|---|
| denovo791 | Eukaryota; Amoebozoa; Archamoebae; Entamoebidae; Iodamoeba; Iodamoeba_sp._RL1 | gut_resident |
| denovo788 | Eukaryota; Amoebozoa; Archamoebae; Entamoebidae; Iodamoeba; Iodamoeba_sp._RL1 | gut_resident |
| denovo787 | Eukaryota; Amoebozoa; Archamoebae; Entamoebidae; Iodamoeba; Iodamoeba_sp._RL1 | gut_resident |
| denovo780 | Eukaryota; Amoebozoa; Archamoebae; Entamoebidae; Iodamoeba; Iodamoeba_sp._RL1 | gut_resident |
| denovo779 | Eukaryota; Amoebozoa; Archamoebae; Entamoebidae; Iodamoeba; Iodamoeba_sp._RL1 | gut_resident |
| denovo777 | Eukaryota; Amoebozoa; Archamoebae; Entamoebidae; Iodamoeba; Iodamoeba_sp._RL1 | gut_resident |
| denovo771 | Eukaryota; Amoebozoa; Archamoebae; Entamoebidae; Iodamoeba; Iodamoeba_sp._RL1 | gut_resident |
| denovo770 | Eukaryota; Amoebozoa; Archamoebae; Entamoebidae; Iodamoeba; Iodamoeba_sp._RL1 | gut_resident |
| denovo769 | Eukaryota; Amoebozoa; Archamoebae; Entamoebidae; Iodamoeba; Iodamoeba_sp._RL1 | gut_resident |
| denovo760 | Eukaryota; Amoebozoa; Archamoebae; Entamoebidae; Iodamoeba; Iodamoeba_sp._RL1 | gut_resident |
| denovo759 | Eukaryota; Amoebozoa; Archamoebae; Entamoebidae; Iodamoeba; Iodamoeba_sp._RL1 | gut_resident |
| denovo756 | Eukaryota; Amoebozoa; Archamoebae; Entamoebidae; Iodamoeba; Iodamoeba_sp._RL1 | gut_resident |
| denovo750 | Eukaryota; Amoebozoa; Archamoebae; Entamoebidae; Iodamoeba; Iodamoeba_sp._RL1 | gut_resident |
| denovo749 | Eukaryota; Amoebozoa; Archamoebae; Entamoebidae; Iodamoeba; Iodamoeba_sp._RL1 | gut_resident |
| denovo747 | Eukaryota; Amoebozoa; Archamoebae; Entamoebidae; Iodamoeba; Iodamoeba_sp._RL1 | gut_resident |
| denovo746 | Eukaryota; Amoebozoa; Archamoebae; Entamoebidae; Iodamoeba; Iodamoeba_sp._RL1 | gut_resident |
| denovo744 | Eukaryota; Amoebozoa; Archamoebae; Entamoebidae; Iodamoeba; Iodamoeba_sp._RL1 | gut_resident |
| denovo743 | Eukaryota; Amoebozoa; Archamoebae; Entamoebidae; Iodamoeba; Iodamoeba_sp._RL1 | gut_resident |
| denovo739 | Eukaryota; Amoebozoa; Archamoebae; Entamoebidae; Iodamoeba; Iodamoeba_sp._RL1 | gut_resident |
| denovo734 | Eukaryota; Amoebozoa; Archamoebae; Entamoebidae; Iodamoeba; Iodamoeba_sp._RL1 | gut_resident |
| denovo732 | Eukaryota; Amoebozoa; Archamoebae; Entamoebidae; Iodamoeba; Iodamoeba_sp._RL1 | gut_resident |
| denovo728 | Eukaryota; Amoebozoa; Archamoebae; Entamoebidae; Iodamoeba; Iodamoeba_sp._RL1 | gut_resident |
| denovo723 | Eukaryota; Amoebozoa; Archamoebae; Entamoebidae; Iodamoeba; Iodamoeba_sp._RL1 | gut_resident |
| denovo717 | Eukaryota; Amoebozoa; Archamoebae; Entamoebidae; Iodamoeba; Iodamoeba_sp._RL1 | gut_resident |
| denovo709 | Eukaryota; Amoebozoa; Archamoebae; Entamoebidae; Iodamoeba; Iodamoeba_sp._RL1 | gut_resident |
| denovo700 | Eukaryota; Amoebozoa; Archamoebae; Entamoebidae; Iodamoeba; Iodamoeba_sp._RL1 | gut_resident |
| denovo698 | Eukaryota; Amoebozoa; Archamoebae; Entamoebidae; Iodamoeba; Iodamoeba_sp._RL1 | gut_resident |
| denovo689 | Eukaryota; Amoebozoa; Archamoebae; Entamoebidae; Iodamoeba; Iodamoeba_sp._RL1 | gut_resident |
| denovo681 | Eukaryota; Amoebozoa; Archamoebae; Entamoebidae; Iodamoeba; Iodamoeba_sp._RL1 | gut_resident |
| denovo680 | Eukaryota; Amoebozoa; Archamoebae; Entamoebidae; Iodamoeba; Iodamoeba_sp._RL1 | gut_resident |
| denovo677 | Eukaryota; Amoebozoa; Archamoebae; Entamoebidae; Iodamoeba; Iodamoeba_sp._RL1 | gut_resident |
| denovo674 | Eukaryota; Amoebozoa; Archamoebae; Entamoebidae; Iodamoeba; Iodamoeba_sp._RL1 | gut_resident |
| denovo671 | Eukaryota; Amoebozoa; Archamoebae; Entamoebidae; Iodamoeba; Iodamoeba_sp._RL1 | gut_resident |
| denovo670 | Eukaryota; Amoebozoa; Archamoebae; Entamoebidae; Iodamoeba; Iodamoeba_sp._RL1 | gut_resident |
| denovo669 | Eukaryota; Amoebozoa; Archamoebae; Entamoebidae; Iodamoeba; Iodamoeba_sp._RL1 | gut_resident |
| denovo666 | Eukaryota; Amoebozoa; Archamoebae; Entamoebidae; Iodamoeba; Iodamoeba_sp._RL1 | gut_resident |
| denovo663 | Eukaryota; Amoebozoa; Archamoebae; Entamoebidae; Iodamoeba; Iodamoeba_sp._RL1 | gut_resident |
| denovo662 | Eukaryota; Amoebozoa; Archamoebae; Entamoebidae; Iodamoeba; Iodamoeba_sp._RL1 | gut_resident |
| denovo656 | Eukaryota; Amoebozoa; Archamoebae; Entamoebidae; Iodamoeba; Iodamoeba_sp._RL1 | gut_resident |
| denovo654 | Eukaryota; Amoebozoa; Archamoebae; Entamoebidae; Iodamoeba; Iodamoeba_sp._RL1 | gut_resident |

| | | |
|---|---|---|
| denovo653 | Eukaryota; Amoebozoa; Archamoebae; Entamoebidae; Iodamoeba; Iodamoeba_sp._RL1 | gut_resident |
| denovo650 | Eukaryota; Amoebozoa; Archamoebae; Entamoebidae; Iodamoeba; Iodamoeba_sp._RL1 | gut_resident |
| denovo646 | Eukaryota; Amoebozoa; Archamoebae; Entamoebidae; Iodamoeba; Iodamoeba_sp._RL1 | gut_resident |
| denovo641 | Eukaryota; Amoebozoa; Archamoebae; Entamoebidae; Iodamoeba; Iodamoeba_sp._RL1 | gut_resident |
| denovo634 | Eukaryota; Amoebozoa; Archamoebae; Entamoebidae; Iodamoeba; Iodamoeba_sp._RL1 | gut_resident |
| denovo631 | Eukaryota; Amoebozoa; Archamoebae; Entamoebidae; Iodamoeba; Iodamoeba_sp._RL1 | gut_resident |
| denovo630 | Eukaryota; Amoebozoa; Archamoebae; Entamoebidae; Iodamoeba; Iodamoeba_sp._RL1 | gut_resident |
| denovo629 | Eukaryota; Amoebozoa; Archamoebae; Entamoebidae; Iodamoeba; Iodamoeba_sp._RL1 | gut_resident |
| denovo627 | Eukaryota; Amoebozoa; Archamoebae; Entamoebidae; Iodamoeba; Iodamoeba_sp._RL1 | gut_resident |
| denovo625 | Eukaryota; Amoebozoa; Archamoebae; Entamoebidae; Iodamoeba; Iodamoeba_sp._RL1 | gut_resident |
| denovo619 | Eukaryota; Amoebozoa; Archamoebae; Entamoebidae; Iodamoeba; Iodamoeba_sp._RL1 | gut_resident |
| denovo612 | Eukaryota; Amoebozoa; Archamoebae; Entamoebidae; Iodamoeba; Iodamoeba_sp._RL1 | gut_resident |
| denovo610 | Eukaryota; Amoebozoa; Archamoebae; Entamoebidae; Iodamoeba; Iodamoeba_sp._RL1 | gut_resident |
| denovo602 | Eukaryota; Amoebozoa; Archamoebae; Entamoebidae; Iodamoeba; Iodamoeba_sp._RL1 | gut_resident |
| denovo595 | Eukaryota; Amoebozoa; Archamoebae; Entamoebidae; Iodamoeba; Iodamoeba_sp._RL1 | gut_resident |
| denovo591 | Eukaryota; Amoebozoa; Archamoebae; Entamoebidae; Iodamoeba; Iodamoeba_sp._RL1 | gut_resident |
| denovo587 | Eukaryota; Amoebozoa; Archamoebae; Entamoebidae; Iodamoeba; Iodamoeba_sp._RL1 | gut_resident |
| denovo579 | Eukaryota; Amoebozoa; Archamoebae; Entamoebidae; Iodamoeba; Iodamoeba_sp._RL1 | gut_resident |
| denovo578 | Eukaryota; Amoebozoa; Archamoebae; Entamoebidae; Iodamoeba; Iodamoeba_sp._RL1 | gut_resident |
| denovo575 | Eukaryota; Amoebozoa; Archamoebae; Entamoebidae; Iodamoeba; Iodamoeba_sp._RL1 | gut_resident |
| denovo569 | Eukaryota; Amoebozoa; Archamoebae; Entamoebidae; Iodamoeba; Iodamoeba_sp._RL1 | gut_resident |
| denovo565 | Eukaryota; Amoebozoa; Archamoebae; Entamoebidae; Iodamoeba; Iodamoeba_sp._RL1 | gut_resident |
| denovo560 | Eukaryota; Amoebozoa; Archamoebae; Entamoebidae; Iodamoeba; Iodamoeba_sp._RL1 | gut_resident |
| denovo549 | Eukaryota; Amoebozoa; Archamoebae; Entamoebidae; Iodamoeba; Iodamoeba_sp._RL1 | gut_resident |
| denovo547 | Eukaryota; Amoebozoa; Archamoebae; Entamoebidae; Iodamoeba; Iodamoeba_sp._RL1 | gut_resident |
| denovo544 | Eukaryota; Amoebozoa; Archamoebae; Entamoebidae; Iodamoeba; Iodamoeba_sp._RL1 | gut_resident |
| denovo537 | Eukaryota; Amoebozoa; Archamoebae; Entamoebidae; Iodamoeba; Iodamoeba_sp._RL1 | gut_resident |
| denovo533 | Eukaryota; Amoebozoa; Archamoebae; Entamoebidae; Iodamoeba; Iodamoeba_sp._RL1 | gut_resident |
| denovo532 | Eukaryota; Amoebozoa; Archamoebae; Entamoebidae; Iodamoeba; Iodamoeba_sp._RL1 | gut_resident |
| denovo528 | Eukaryota; Amoebozoa; Archamoebae; Entamoebidae; Iodamoeba; Iodamoeba_sp._RL1 | gut_resident |
| denovo524 | Eukaryota; Amoebozoa; Archamoebae; Entamoebidae; Iodamoeba; Iodamoeba_sp._RL1 | gut_resident |
| denovo522 | Eukaryota; Amoebozoa; Archamoebae; Entamoebidae; Iodamoeba; Iodamoeba_sp._RL1 | gut_resident |
| denovo518 | Eukaryota; Amoebozoa; Archamoebae; Entamoebidae; Iodamoeba; Iodamoeba_sp._RL1 | gut_resident |
| denovo510 | Eukaryota; Amoebozoa; Archamoebae; Entamoebidae; Iodamoeba; Iodamoeba_sp._RL1 | gut_resident |
| denovo509 | Eukaryota; Amoebozoa; Archamoebae; Entamoebidae; Iodamoeba; Iodamoeba_sp._RL1 | gut_resident |
| denovo506 | Eukaryota; Amoebozoa; Archamoebae; Entamoebidae; Iodamoeba; Iodamoeba_sp._RL1 | gut_resident |
| denovo502 | Eukaryota; Amoebozoa; Archamoebae; Entamoebidae; Iodamoeba; Iodamoeba_sp._RL1 | gut_resident |
| denovo501 | Eukaryota; Amoebozoa; Archamoebae; Entamoebidae; Iodamoeba; Iodamoeba_sp._RL1 | gut_resident |
| denovo499 | Eukaryota; Amoebozoa; Archamoebae; Entamoebidae; Iodamoeba; Iodamoeba_sp._RL1 | gut_resident |
| denovo492 | Eukaryota; Amoebozoa; Archamoebae; Entamoebidae; Iodamoeba; Iodamoeba_sp._RL1 | gut_resident |

| | | |
|---|---|---|
| denovo466 | Eukaryota; Amoebozoa; Archamoebae; Entamoebidae; Iodamoeba; Iodamoeba_sp._RL1 | gut_resident |
| denovo464 | Eukaryota; Amoebozoa; Archamoebae; Entamoebidae; Iodamoeba; Iodamoeba_sp._RL1 | gut_resident |
| denovo457 | Eukaryota; Amoebozoa; Archamoebae; Entamoebidae; Iodamoeba; Iodamoeba_sp._RL1 | gut_resident |
| denovo450 | Eukaryota; Amoebozoa; Archamoebae; Entamoebidae; Iodamoeba; Iodamoeba_sp._RL1 | gut_resident |
| denovo443 | Eukaryota; Amoebozoa; Archamoebae; Entamoebidae; Iodamoeba; Iodamoeba_sp._RL1 | gut_resident |
| denovo436 | Eukaryota; Amoebozoa; Archamoebae; Entamoebidae; Iodamoeba; Iodamoeba_sp._RL1 | gut_resident |
| denovo414 | Eukaryota; Amoebozoa; Archamoebae; Entamoebidae; Iodamoeba; Iodamoeba_sp._RL1 | gut_resident |
| denovo409 | Eukaryota; Amoebozoa; Archamoebae; Entamoebidae; Iodamoeba; Iodamoeba_sp._RL1 | gut_resident |
| denovo401 | Eukaryota; Amoebozoa; Archamoebae; Entamoebidae; Iodamoeba; Iodamoeba_sp._RL1 | gut_resident |
| denovo392 | Eukaryota; Amoebozoa; Archamoebae; Entamoebidae; Iodamoeba; Iodamoeba_sp._RL1 | gut_resident |
| denovo391 | Eukaryota; Amoebozoa; Archamoebae; Entamoebidae; Iodamoeba; Iodamoeba_sp._RL1 | gut_resident |
| denovo388 | Eukaryota; Amoebozoa; Archamoebae; Entamoebidae; Iodamoeba; Iodamoeba_sp._RL1 | gut_resident |
| denovo382 | Eukaryota; Amoebozoa; Archamoebae; Entamoebidae; Iodamoeba; Iodamoeba_sp._RL1 | gut_resident |
| denovo376 | Eukaryota; Amoebozoa; Archamoebae; Entamoebidae; Iodamoeba; Iodamoeba_sp._RL1 | gut_resident |
| denovo372 | Eukaryota; Amoebozoa; Archamoebae; Entamoebidae; Iodamoeba; Iodamoeba_sp._RL1 | gut_resident |
| denovo371 | Eukaryota; Amoebozoa; Archamoebae; Entamoebidae; Iodamoeba; Iodamoeba_sp._RL1 | gut_resident |
| denovo37 | Eukaryota; Amoebozoa; Archamoebae; Entamoebidae; Iodamoeba; Iodamoeba_sp._RL1 | gut_resident |
| denovo368 | Eukaryota; Amoebozoa; Archamoebae; Entamoebidae; Iodamoeba; Iodamoeba_sp._RL1 | gut_resident |
| denovo362 | Eukaryota; Amoebozoa; Archamoebae; Entamoebidae; Iodamoeba; Iodamoeba_sp._RL1 | gut_resident |
| denovo347 | Eukaryota; Amoebozoa; Archamoebae; Entamoebidae; Iodamoeba; Iodamoeba_sp._RL1 | gut_resident |
| denovo343 | Eukaryota; Amoebozoa; Archamoebae; Entamoebidae; Iodamoeba; Iodamoeba_sp._RL1 | gut_resident |
| denovo340 | Eukaryota; Amoebozoa; Archamoebae; Entamoebidae; Iodamoeba; Iodamoeba_sp._RL1 | gut_resident |
| denovo339 | Eukaryota; Amoebozoa; Archamoebae; Entamoebidae; Iodamoeba; Iodamoeba_sp._RL1 | gut_resident |
| denovo3318 | Eukaryota; Amoebozoa; Archamoebae; Entamoebidae; Iodamoeba; Iodamoeba_sp._RL1 | gut_resident |
| denovo3317 | Eukaryota; Amoebozoa; Archamoebae; Entamoebidae; Iodamoeba; Iodamoeba_sp._RL1 | gut_resident |
| denovo3306 | Eukaryota; Amoebozoa; Archamoebae; Entamoebidae; Iodamoeba; Iodamoeba_sp._RL1 | gut_resident |
| denovo330 | Eukaryota; Amoebozoa; Archamoebae; Entamoebidae; Iodamoeba; Iodamoeba_sp._RL1 | gut_resident |
| denovo33 | Eukaryota; Amoebozoa; Archamoebae; Entamoebidae; Iodamoeba; Iodamoeba_sp._RL1 | gut_resident |
| denovo3296 | Eukaryota; Amoebozoa; Archamoebae; Entamoebidae; Iodamoeba; Iodamoeba_sp._RL1 | gut_resident |
| denovo3295 | Eukaryota; Amoebozoa; Archamoebae; Entamoebidae; Iodamoeba; Iodamoeba_sp._RL1 | gut_resident |
| denovo3290 | Eukaryota; Amoebozoa; Archamoebae; Entamoebidae; Iodamoeba; Iodamoeba_sp._RL1 | gut_resident |
| denovo3289 | Eukaryota; Amoebozoa; Archamoebae; Entamoebidae; Iodamoeba; Iodamoeba_sp._RL1 | gut_resident |
| denovo3288 | Eukaryota; Amoebozoa; Archamoebae; Entamoebidae; Iodamoeba; Iodamoeba_sp._RL1 | gut_resident |
| denovo3286 | Eukaryota; Amoebozoa; Archamoebae; Entamoebidae; Iodamoeba; Iodamoeba_sp._RL1 | gut_resident |
| denovo3248 | Eukaryota; Amoebozoa; Archamoebae; Entamoebidae; Iodamoeba; Iodamoeba_sp._RL1 | gut_resident |
| denovo3247 | Eukaryota; Amoebozoa; Archamoebae; Entamoebidae; Iodamoeba; Iodamoeba_sp._RL1 | gut_resident |
| denovo3231 | Eukaryota; Amoebozoa; Archamoebae; Entamoebidae; Iodamoeba; Iodamoeba_sp._RL1 | gut_resident |
| denovo3230 | Eukaryota; Amoebozoa; Archamoebae; Entamoebidae; Iodamoeba; Iodamoeba_sp._RL1 | gut_resident |
| denovo3229 | Eukaryota; Amoebozoa; Archamoebae; Entamoebidae; Iodamoeba; Iodamoeba_sp._RL1 | gut_resident |
| denovo3228 | Eukaryota; Amoebozoa; Archamoebae; Entamoebidae; Iodamoeba; Iodamoeba_sp._RL1 | gut_resident |

| | | |
|---|---|---|
| denovo3227 | Eukaryota; Amoebozoa; Archamoebae; Entamoebidae; Iodamoeba; Iodamoeba_sp._RL1 | gut_resident |
| denovo3225 | Eukaryota; Amoebozoa; Archamoebae; Entamoebidae; Iodamoeba; Iodamoeba_sp._RL1 | gut_resident |
| denovo3224 | Eukaryota; Amoebozoa; Archamoebae; Entamoebidae; Iodamoeba; Iodamoeba_sp._RL1 | gut_resident |
| denovo3223 | Eukaryota; Amoebozoa; Archamoebae; Entamoebidae; Iodamoeba; Iodamoeba_sp._RL1 | gut_resident |
| denovo3201 | Eukaryota; Amoebozoa; Archamoebae; Entamoebidae; Iodamoeba; Iodamoeba_sp._RL1 | gut_resident |
| denovo3193 | Eukaryota; Amoebozoa; Archamoebae; Entamoebidae; Iodamoeba; Iodamoeba_sp._RL1 | gut_resident |
| denovo3192 | Eukaryota; Amoebozoa; Archamoebae; Entamoebidae; Iodamoeba; Iodamoeba_sp._RL1 | gut_resident |
| denovo3189 | Eukaryota; Amoebozoa; Archamoebae; Entamoebidae; Iodamoeba; Iodamoeba_sp._RL1 | gut_resident |
| denovo3174 | Eukaryota; Amoebozoa; Archamoebae; Entamoebidae; Iodamoeba; Iodamoeba_sp._RL1 | gut_resident |
| denovo3173 | Eukaryota; Amoebozoa; Archamoebae; Entamoebidae; Iodamoeba; Iodamoeba_sp._RL1 | gut_resident |
| denovo3170 | Eukaryota; Amoebozoa; Archamoebae; Entamoebidae; Iodamoeba; Iodamoeba_sp._RL1 | gut_resident |
| denovo3169 | Eukaryota; Amoebozoa; Archamoebae; Entamoebidae; Iodamoeba; Iodamoeba_sp._RL1 | gut_resident |
| denovo3168 | Eukaryota; Amoebozoa; Archamoebae; Entamoebidae; Iodamoeba; Iodamoeba_sp._RL1 | gut_resident |
| denovo3167 | Eukaryota; Amoebozoa; Archamoebae; Entamoebidae; Iodamoeba; Iodamoeba_sp._RL1 | gut_resident |
| denovo3165 | Eukaryota; Amoebozoa; Archamoebae; Entamoebidae; Iodamoeba; Iodamoeba_sp._RL1 | gut_resident |
| denovo3164 | Eukaryota; Amoebozoa; Archamoebae; Entamoebidae; Iodamoeba; Iodamoeba_sp._RL1 | gut_resident |
| denovo316 | Eukaryota; Amoebozoa; Archamoebae; Entamoebidae; Iodamoeba; Iodamoeba_sp._RL1 | gut_resident |
| denovo3147 | Eukaryota; Amoebozoa; Archamoebae; Entamoebidae; Iodamoeba; Iodamoeba_sp._RL1 | gut_resident |
| denovo3145 | Eukaryota; Amoebozoa; Archamoebae; Entamoebidae; Iodamoeba; Iodamoeba_sp._RL1 | gut_resident |
| denovo3144 | Eukaryota; Amoebozoa; Archamoebae; Entamoebidae; Iodamoeba; Iodamoeba_sp._RL1 | gut_resident |
| denovo3143 | Eukaryota; Amoebozoa; Archamoebae; Entamoebidae; Iodamoeba; Iodamoeba_sp._RL1 | gut_resident |
| denovo3142 | Eukaryota; Amoebozoa; Archamoebae; Entamoebidae; Iodamoeba; Iodamoeba_sp._RL1 | gut_resident |
| denovo3141 | Eukaryota; Amoebozoa; Archamoebae; Entamoebidae; Iodamoeba; Iodamoeba_sp._RL1 | gut_resident |
| denovo3140 | Eukaryota; Amoebozoa; Archamoebae; Entamoebidae; Iodamoeba; Iodamoeba_sp._RL1 | gut_resident |
| denovo3138 | Eukaryota; Amoebozoa; Archamoebae; Entamoebidae; Iodamoeba; Iodamoeba_sp._RL1 | gut_resident |
| denovo3137 | Eukaryota; Amoebozoa; Archamoebae; Entamoebidae; Iodamoeba; Iodamoeba_sp._RL1 | gut_resident |
| denovo3136 | Eukaryota; Amoebozoa; Archamoebae; Entamoebidae; Iodamoeba; Iodamoeba_sp._RL1 | gut_resident |
| denovo3135 | Eukaryota; Amoebozoa; Archamoebae; Entamoebidae; Iodamoeba; Iodamoeba_sp._RL1 | gut_resident |
| denovo3134 | Eukaryota; Amoebozoa; Archamoebae; Entamoebidae; Iodamoeba; Iodamoeba_sp._RL1 | gut_resident |
| denovo3132 | Eukaryota; Amoebozoa; Archamoebae; Entamoebidae; Iodamoeba; Iodamoeba_sp._RL1 | gut_resident |
| denovo3123 | Eukaryota; Amoebozoa; Archamoebae; Entamoebidae; Iodamoeba; Iodamoeba_sp._RL1 | gut_resident |
| denovo3122 | Eukaryota; Amoebozoa; Archamoebae; Entamoebidae; Iodamoeba; Iodamoeba_sp._RL1 | gut_resident |
| denovo3121 | Eukaryota; Amoebozoa; Archamoebae; Entamoebidae; Iodamoeba; Iodamoeba_sp._RL1 | gut_resident |
| denovo3120 | Eukaryota; Amoebozoa; Archamoebae; Entamoebidae; Iodamoeba; Iodamoeba_sp._RL1 | gut_resident |
| denovo3118 | Eukaryota; Amoebozoa; Archamoebae; Entamoebidae; Iodamoeba; Iodamoeba_sp._RL1 | gut_resident |
| denovo3117 | Eukaryota; Amoebozoa; Archamoebae; Entamoebidae; Iodamoeba; Iodamoeba_sp._RL1 | gut_resident |
| denovo3116 | Eukaryota; Amoebozoa; Archamoebae; Entamoebidae; Iodamoeba; Iodamoeba_sp._RL1 | gut_resident |
| denovo3111 | Eukaryota; Amoebozoa; Archamoebae; Entamoebidae; Iodamoeba; Iodamoeba_sp._RL1 | gut_resident |
| denovo3110 | Eukaryota; Amoebozoa; Archamoebae; Entamoebidae; Iodamoeba; Iodamoeba_sp._RL1 | gut_resident |
| denovo3108 | Eukaryota; Amoebozoa; Archamoebae; Entamoebidae; Iodamoeba; Iodamoeba_sp._RL1 | gut_resident |

| | | |
|---|---|---|
| denovo3107 | Eukaryota; Amoebozoa; Archamoebae; Entamoebidae; Iodamoeba; Iodamoeba_sp._RL1 | gut_resident |
| denovo3106 | Eukaryota; Amoebozoa; Archamoebae; Entamoebidae; Iodamoeba; Iodamoeba_sp._RL1 | gut_resident |
| denovo3104 | Eukaryota; Amoebozoa; Archamoebae; Entamoebidae; Iodamoeba; Iodamoeba_sp._RL1 | gut_resident |
| denovo3098 | Eukaryota; Amoebozoa; Archamoebae; Entamoebidae; Iodamoeba; Iodamoeba_sp._RL1 | gut_resident |
| denovo3094 | Eukaryota; Amoebozoa; Archamoebae; Entamoebidae; Iodamoeba; Iodamoeba_sp._RL1 | gut_resident |
| denovo309 | Eukaryota; Amoebozoa; Archamoebae; Entamoebidae; Iodamoeba; Iodamoeba_sp._RL1 | gut_resident |
| denovo3082 | Eukaryota; Amoebozoa; Archamoebae; Entamoebidae; Iodamoeba; Iodamoeba_sp._RL1 | gut_resident |
| denovo3077 | Eukaryota; Amoebozoa; Archamoebae; Entamoebidae; Iodamoeba; Iodamoeba_sp._RL1 | gut_resident |
| denovo3076 | Eukaryota; Amoebozoa; Archamoebae; Entamoebidae; Iodamoeba; Iodamoeba_sp._RL1 | gut_resident |
| denovo3075 | Eukaryota; Amoebozoa; Archamoebae; Entamoebidae; Iodamoeba; Iodamoeba_sp._RL1 | gut_resident |
| denovo3046 | Eukaryota; Amoebozoa; Archamoebae; Entamoebidae; Iodamoeba; Iodamoeba_sp._RL1 | gut_resident |
| denovo3043 | Eukaryota; Amoebozoa; Archamoebae; Entamoebidae; Iodamoeba; Iodamoeba_sp._RL1 | gut_resident |
| denovo3042 | Eukaryota; Amoebozoa; Archamoebae; Entamoebidae; Iodamoeba; Iodamoeba_sp._RL1 | gut_resident |
| denovo3041 | Eukaryota; Amoebozoa; Archamoebae; Entamoebidae; Iodamoeba; Iodamoeba_sp._RL1 | gut_resident |
| denovo3040 | Eukaryota; Amoebozoa; Archamoebae; Entamoebidae; Iodamoeba; Iodamoeba_sp._RL1 | gut_resident |
| denovo3039 | Eukaryota; Amoebozoa; Archamoebae; Entamoebidae; Iodamoeba; Iodamoeba_sp._RL1 | gut_resident |
| denovo3038 | Eukaryota; Amoebozoa; Archamoebae; Entamoebidae; Iodamoeba; Iodamoeba_sp._RL1 | gut_resident |
| denovo3009 | Eukaryota; Amoebozoa; Archamoebae; Entamoebidae; Iodamoeba; Iodamoeba_sp._RL1 | gut_resident |
| denovo3008 | Eukaryota; Amoebozoa; Archamoebae; Entamoebidae; Iodamoeba; Iodamoeba_sp._RL1 | gut_resident |
| denovo3007 | Eukaryota; Amoebozoa; Archamoebae; Entamoebidae; Iodamoeba; Iodamoeba_sp._RL1 | gut_resident |
| denovo2995 | Eukaryota; Amoebozoa; Archamoebae; Entamoebidae; Iodamoeba; Iodamoeba_sp._RL1 | gut_resident |
| denovo2992 | Eukaryota; Amoebozoa; Archamoebae; Entamoebidae; Iodamoeba; Iodamoeba_sp._RL1 | gut_resident |
| denovo2991 | Eukaryota; Amoebozoa; Archamoebae; Entamoebidae; Iodamoeba; Iodamoeba_sp._RL1 | gut_resident |
| denovo2963 | Eukaryota; Amoebozoa; Archamoebae; Entamoebidae; Iodamoeba; Iodamoeba_sp._RL1 | gut_resident |
| denovo2962 | Eukaryota; Amoebozoa; Archamoebae; Entamoebidae; Iodamoeba; Iodamoeba_sp._RL1 | gut_resident |
| denovo2961 | Eukaryota; Amoebozoa; Archamoebae; Entamoebidae; Iodamoeba; Iodamoeba_sp._RL1 | gut_resident |
| denovo2960 | Eukaryota; Amoebozoa; Archamoebae; Entamoebidae; Iodamoeba; Iodamoeba_sp._RL1 | gut_resident |
| denovo2958 | Eukaryota; Amoebozoa; Archamoebae; Entamoebidae; Iodamoeba; Iodamoeba_sp._RL1 | gut_resident |
| denovo2957 | Eukaryota; Amoebozoa; Archamoebae; Entamoebidae; Iodamoeba; Iodamoeba_sp._RL1 | gut_resident |
| denovo2956 | Eukaryota; Amoebozoa; Archamoebae; Entamoebidae; Iodamoeba; Iodamoeba_sp._RL1 | gut_resident |
| denovo2944 | Eukaryota; Amoebozoa; Archamoebae; Entamoebidae; Iodamoeba; Iodamoeba_sp._RL1 | gut_resident |
| denovo2941 | Eukaryota; Amoebozoa; Archamoebae; Entamoebidae; Iodamoeba; Iodamoeba_sp._RL1 | gut_resident |
| denovo2940 | Eukaryota; Amoebozoa; Archamoebae; Entamoebidae; Iodamoeba; Iodamoeba_sp._RL1 | gut_resident |
| denovo2939 | Eukaryota; Amoebozoa; Archamoebae; Entamoebidae; Iodamoeba; Iodamoeba_sp._RL1 | gut_resident |
| denovo2938 | Eukaryota; Amoebozoa; Archamoebae; Entamoebidae; Iodamoeba; Iodamoeba_sp._RL1 | gut_resident |
| denovo2934 | Eukaryota; Amoebozoa; Archamoebae; Entamoebidae; Iodamoeba; Iodamoeba_sp._RL1 | gut_resident |
| denovo2932 | Eukaryota; Amoebozoa; Archamoebae; Entamoebidae; Iodamoeba; Iodamoeba_sp._RL1 | gut_resident |
| denovo2931 | Eukaryota; Amoebozoa; Archamoebae; Entamoebidae; Iodamoeba; Iodamoeba_sp._RL1 | gut_resident |
| denovo2930 | Eukaryota; Amoebozoa; Archamoebae; Entamoebidae; Iodamoeba; Iodamoeba_sp._RL1 | gut_resident |
| denovo2929 | Eukaryota; Amoebozoa; Archamoebae; Entamoebidae; Iodamoeba; Iodamoeba_sp._RL1 | gut_resident |

| | | |
|---|---|---|
| denovo2928 | Eukaryota; Amoebozoa; Archamoebae; Entamoebidae; Iodamoeba; Iodamoeba_sp._RL1 | gut_resident |
| denovo2925 | Eukaryota; Amoebozoa; Archamoebae; Entamoebidae; Iodamoeba; Iodamoeba_sp._RL1 | gut_resident |
| denovo2924 | Eukaryota; Amoebozoa; Archamoebae; Entamoebidae; Iodamoeba; Iodamoeba_sp._RL1 | gut_resident |
| denovo2923 | Eukaryota; Amoebozoa; Archamoebae; Entamoebidae; Iodamoeba; Iodamoeba_sp._RL1 | gut_resident |
| denovo2922 | Eukaryota; Amoebozoa; Archamoebae; Entamoebidae; Iodamoeba; Iodamoeba_sp._RL1 | gut_resident |
| denovo2921 | Eukaryota; Amoebozoa; Archamoebae; Entamoebidae; Iodamoeba; Iodamoeba_sp._RL1 | gut_resident |
| denovo2916 | Eukaryota; Amoebozoa; Archamoebae; Entamoebidae; Iodamoeba; Iodamoeba_sp._RL1 | gut_resident |
| denovo2914 | Eukaryota; Amoebozoa; Archamoebae; Entamoebidae; Iodamoeba; Iodamoeba_sp._RL1 | gut_resident |
| denovo2913 | Eukaryota; Amoebozoa; Archamoebae; Entamoebidae; Iodamoeba; Iodamoeba_sp._RL1 | gut_resident |
| denovo2911 | Eukaryota; Amoebozoa; Archamoebae; Entamoebidae; Iodamoeba; Iodamoeba_sp._RL1 | gut_resident |
| denovo2910 | Eukaryota; Amoebozoa; Archamoebae; Entamoebidae; Iodamoeba; Iodamoeba_sp._RL1 | gut_resident |
| denovo2893 | Eukaryota; Amoebozoa; Archamoebae; Entamoebidae; Iodamoeba; Iodamoeba_sp._RL1 | gut_resident |
| denovo2892 | Eukaryota; Amoebozoa; Archamoebae; Entamoebidae; Iodamoeba; Iodamoeba_sp._RL1 | gut_resident |
| denovo2890 | Eukaryota; Amoebozoa; Archamoebae; Entamoebidae; Iodamoeba; Iodamoeba_sp._RL1 | gut_resident |
| denovo2889 | Eukaryota; Amoebozoa; Archamoebae; Entamoebidae; Iodamoeba; Iodamoeba_sp._RL1 | gut_resident |
| denovo2873 | Eukaryota; Amoebozoa; Archamoebae; Entamoebidae; Iodamoeba; Iodamoeba_sp._RL1 | gut_resident |
| denovo2872 | Eukaryota; Amoebozoa; Archamoebae; Entamoebidae; Iodamoeba; Iodamoeba_sp._RL1 | gut_resident |
| denovo2865 | Eukaryota; Amoebozoa; Archamoebae; Entamoebidae; Iodamoeba; Iodamoeba_sp._RL1 | gut_resident |
| denovo2848 | Eukaryota; Amoebozoa; Archamoebae; Entamoebidae; Iodamoeba; Iodamoeba_sp._RL1 | gut_resident |
| denovo2847 | Eukaryota; Amoebozoa; Archamoebae; Entamoebidae; Iodamoeba; Iodamoeba_sp._RL1 | gut_resident |
| denovo2846 | Eukaryota; Amoebozoa; Archamoebae; Entamoebidae; Iodamoeba; Iodamoeba_sp._RL1 | gut_resident |
| denovo2837 | Eukaryota; Amoebozoa; Archamoebae; Entamoebidae; Iodamoeba; Iodamoeba_sp._RL1 | gut_resident |
| denovo2836 | Eukaryota; Amoebozoa; Archamoebae; Entamoebidae; Iodamoeba; Iodamoeba_sp._RL1 | gut_resident |
| denovo2835 | Eukaryota; Amoebozoa; Archamoebae; Entamoebidae; Iodamoeba; Iodamoeba_sp._RL1 | gut_resident |
| denovo2832 | Eukaryota; Amoebozoa; Archamoebae; Entamoebidae; Iodamoeba; Iodamoeba_sp._RL1 | gut_resident |
| denovo2812 | Eukaryota; Amoebozoa; Archamoebae; Entamoebidae; Iodamoeba; Iodamoeba_sp._RL1 | gut_resident |
| denovo2808 | Eukaryota; Amoebozoa; Archamoebae; Entamoebidae; Iodamoeba; Iodamoeba_sp._RL1 | gut_resident |
| denovo2807 | Eukaryota; Amoebozoa; Archamoebae; Entamoebidae; Iodamoeba; Iodamoeba_sp._RL1 | gut_resident |
| denovo2806 | Eukaryota; Amoebozoa; Archamoebae; Entamoebidae; Iodamoeba; Iodamoeba_sp._RL1 | gut_resident |
| denovo2805 | Eukaryota; Amoebozoa; Archamoebae; Entamoebidae; Iodamoeba; Iodamoeba_sp._RL1 | gut_resident |
| denovo2792 | Eukaryota; Amoebozoa; Archamoebae; Entamoebidae; Iodamoeba; Iodamoeba_sp._RL1 | gut_resident |
| denovo2791 | Eukaryota; Amoebozoa; Archamoebae; Entamoebidae; Iodamoeba; Iodamoeba_sp._RL1 | gut_resident |
| denovo2790 | Eukaryota; Amoebozoa; Archamoebae; Entamoebidae; Iodamoeba; Iodamoeba_sp._RL1 | gut_resident |
| denovo2788 | Eukaryota; Amoebozoa; Archamoebae; Entamoebidae; Iodamoeba; Iodamoeba_sp._RL1 | gut_resident |
| denovo2779 | Eukaryota; Amoebozoa; Archamoebae; Entamoebidae; Iodamoeba; Iodamoeba_sp._RL1 | gut_resident |
| denovo2776 | Eukaryota; Amoebozoa; Archamoebae; Entamoebidae; Iodamoeba; Iodamoeba_sp._RL1 | gut_resident |
| denovo2775 | Eukaryota; Amoebozoa; Archamoebae; Entamoebidae; Iodamoeba; Iodamoeba_sp._RL1 | gut_resident |
| denovo2774 | Eukaryota; Amoebozoa; Archamoebae; Entamoebidae; Iodamoeba; Iodamoeba_sp._RL1 | gut_resident |
| denovo2773 | Eukaryota; Amoebozoa; Archamoebae; Entamoebidae; Iodamoeba; Iodamoeba_sp._RL1 | gut_resident |
| denovo2771 | Eukaryota; Amoebozoa; Archamoebae; Entamoebidae; Iodamoeba; Iodamoeba_sp._RL1 | gut_resident |

| | | |
|---|---|---|
| denovo2763 | Eukaryota; Amoebozoa; Archamoebae; Entamoebidae; Iodamoeba; Iodamoeba_sp._RL1 | gut_resident |
| denovo2761 | Eukaryota; Amoebozoa; Archamoebae; Entamoebidae; Iodamoeba; Iodamoeba_sp._RL1 | gut_resident |
| denovo2757 | Eukaryota; Amoebozoa; Archamoebae; Entamoebidae; Iodamoeba; Iodamoeba_sp._RL1 | gut_resident |
| denovo2755 | Eukaryota; Amoebozoa; Archamoebae; Entamoebidae; Iodamoeba; Iodamoeba_sp._RL1 | gut_resident |
| denovo2745 | Eukaryota; Amoebozoa; Archamoebae; Entamoebidae; Iodamoeba; Iodamoeba_sp._RL1 | gut_resident |
| denovo2743 | Eukaryota; Amoebozoa; Archamoebae; Entamoebidae; Iodamoeba; Iodamoeba_sp._RL1 | gut_resident |
| denovo2722 | Eukaryota; Amoebozoa; Archamoebae; Entamoebidae; Iodamoeba; Iodamoeba_sp._RL1 | gut_resident |
| denovo2700 | Eukaryota; Amoebozoa; Archamoebae; Entamoebidae; Iodamoeba; Iodamoeba_sp._RL1 | gut_resident |
| denovo27 | Eukaryota; Amoebozoa; Archamoebae; Entamoebidae; Iodamoeba; Iodamoeba_sp._RL1 | gut_resident |
| denovo2699 | Eukaryota; Amoebozoa; Archamoebae; Entamoebidae; Iodamoeba; Iodamoeba_sp._RL1 | gut_resident |
| denovo2698 | Eukaryota; Amoebozoa; Archamoebae; Entamoebidae; Iodamoeba; Iodamoeba_sp._RL1 | gut_resident |
| denovo2697 | Eukaryota; Amoebozoa; Archamoebae; Entamoebidae; Iodamoeba; Iodamoeba_sp._RL1 | gut_resident |
| denovo2673 | Eukaryota; Amoebozoa; Archamoebae; Entamoebidae; Iodamoeba; Iodamoeba_sp._RL1 | gut_resident |
| denovo2672 | Eukaryota; Amoebozoa; Archamoebae; Entamoebidae; Iodamoeba; Iodamoeba_sp._RL1 | gut_resident |
| denovo2666 | Eukaryota; Amoebozoa; Archamoebae; Entamoebidae; Iodamoeba; Iodamoeba_sp._RL1 | gut_resident |
| denovo2665 | Eukaryota; Amoebozoa; Archamoebae; Entamoebidae; Iodamoeba; Iodamoeba_sp._RL1 | gut_resident |
| denovo2664 | Eukaryota; Amoebozoa; Archamoebae; Entamoebidae; Iodamoeba; Iodamoeba_sp._RL1 | gut_resident |
| denovo2650 | Eukaryota; Amoebozoa; Archamoebae; Entamoebidae; Iodamoeba; Iodamoeba_sp._RL1 | gut_resident |
| denovo2649 | Eukaryota; Amoebozoa; Archamoebae; Entamoebidae; Iodamoeba; Iodamoeba_sp._RL1 | gut_resident |
| denovo2646 | Eukaryota; Amoebozoa; Archamoebae; Entamoebidae; Iodamoeba; Iodamoeba_sp._RL1 | gut_resident |
| denovo2645 | Eukaryota; Amoebozoa; Archamoebae; Entamoebidae; Iodamoeba; Iodamoeba_sp._RL1 | gut_resident |
| denovo2644 | Eukaryota; Amoebozoa; Archamoebae; Entamoebidae; Iodamoeba; Iodamoeba_sp._RL1 | gut_resident |
| denovo2642 | Eukaryota; Amoebozoa; Archamoebae; Entamoebidae; Iodamoeba; Iodamoeba_sp._RL1 | gut_resident |
| denovo2640 | Eukaryota; Amoebozoa; Archamoebae; Entamoebidae; Iodamoeba; Iodamoeba_sp._RL1 | gut_resident |
| denovo2634 | Eukaryota; Amoebozoa; Archamoebae; Entamoebidae; Iodamoeba; Iodamoeba_sp._RL1 | gut_resident |
| denovo2632 | Eukaryota; Amoebozoa; Archamoebae; Entamoebidae; Iodamoeba; Iodamoeba_sp._RL1 | gut_resident |
| denovo2631 | Eukaryota; Amoebozoa; Archamoebae; Entamoebidae; Iodamoeba; Iodamoeba_sp._RL1 | gut_resident |
| denovo2626 | Eukaryota; Amoebozoa; Archamoebae; Entamoebidae; Iodamoeba; Iodamoeba_sp._RL1 | gut_resident |
| denovo2625 | Eukaryota; Amoebozoa; Archamoebae; Entamoebidae; Iodamoeba; Iodamoeba_sp._RL1 | gut_resident |
| denovo2622 | Eukaryota; Amoebozoa; Archamoebae; Entamoebidae; Iodamoeba; Iodamoeba_sp._RL1 | gut_resident |
| denovo2613 | Eukaryota; Amoebozoa; Archamoebae; Entamoebidae; Iodamoeba; Iodamoeba_sp._RL1 | gut_resident |
| denovo2612 | Eukaryota; Amoebozoa; Archamoebae; Entamoebidae; Iodamoeba; Iodamoeba_sp._RL1 | gut_resident |
| denovo2611 | Eukaryota; Amoebozoa; Archamoebae; Entamoebidae; Iodamoeba; Iodamoeba_sp._RL1 | gut_resident |
| denovo2609 | Eukaryota; Amoebozoa; Archamoebae; Entamoebidae; Iodamoeba; Iodamoeba_sp._RL1 | gut_resident |
| denovo2606 | Eukaryota; Amoebozoa; Archamoebae; Entamoebidae; Iodamoeba; Iodamoeba_sp._RL1 | gut_resident |
| denovo2605 | Eukaryota; Amoebozoa; Archamoebae; Entamoebidae; Iodamoeba; Iodamoeba_sp._RL1 | gut_resident |
| denovo2592 | Eukaryota; Amoebozoa; Archamoebae; Entamoebidae; Iodamoeba; Iodamoeba_sp._RL1 | gut_resident |
| denovo2591 | Eukaryota; Amoebozoa; Archamoebae; Entamoebidae; Iodamoeba; Iodamoeba_sp._RL1 | gut_resident |
| denovo2588 | Eukaryota; Amoebozoa; Archamoebae; Entamoebidae; Iodamoeba; Iodamoeba_sp._RL1 | gut_resident |
| denovo2567 | Eukaryota; Amoebozoa; Archamoebae; Entamoebidae; Iodamoeba; Iodamoeba_sp._RL1 | gut_resident |

| | | |
|---|---|---|
| denovo2566 | Eukaryota; Amoebozoa; Archamoebae; Entamoebidae; Iodamoeba; Iodamoeba_sp._RL1 | gut_resident |
| denovo2556 | Eukaryota; Amoebozoa; Archamoebae; Entamoebidae; Iodamoeba; Iodamoeba_sp._RL1 | gut_resident |
| denovo2545 | Eukaryota; Amoebozoa; Archamoebae; Entamoebidae; Iodamoeba; Iodamoeba_sp._RL1 | gut_resident |
| denovo2544 | Eukaryota; Amoebozoa; Archamoebae; Entamoebidae; Iodamoeba; Iodamoeba_sp._RL1 | gut_resident |
| denovo2524 | Eukaryota; Amoebozoa; Archamoebae; Entamoebidae; Iodamoeba; Iodamoeba_sp._RL1 | gut_resident |
| denovo2517 | Eukaryota; Amoebozoa; Archamoebae; Entamoebidae; Iodamoeba; Iodamoeba_sp._RL1 | gut_resident |
| denovo2516 | Eukaryota; Amoebozoa; Archamoebae; Entamoebidae; Iodamoeba; Iodamoeba_sp._RL1 | gut_resident |
| denovo2515 | Eukaryota; Amoebozoa; Archamoebae; Entamoebidae; Iodamoeba; Iodamoeba_sp._RL1 | gut_resident |
| denovo2514 | Eukaryota; Amoebozoa; Archamoebae; Entamoebidae; Iodamoeba; Iodamoeba_sp._RL1 | gut_resident |
| denovo2513 | Eukaryota; Amoebozoa; Archamoebae; Entamoebidae; Iodamoeba; Iodamoeba_sp._RL1 | gut_resident |
| denovo2502 | Eukaryota; Amoebozoa; Archamoebae; Entamoebidae; Iodamoeba; Iodamoeba_sp._RL1 | gut_resident |
| denovo2501 | Eukaryota; Amoebozoa; Archamoebae; Entamoebidae; Iodamoeba; Iodamoeba_sp._RL1 | gut_resident |
| denovo2500 | Eukaryota; Amoebozoa; Archamoebae; Entamoebidae; Iodamoeba; Iodamoeba_sp._RL1 | gut_resident |
| denovo2488 | Eukaryota; Amoebozoa; Archamoebae; Entamoebidae; Iodamoeba; Iodamoeba_sp._RL1 | gut_resident |
| denovo2487 | Eukaryota; Amoebozoa; Archamoebae; Entamoebidae; Iodamoeba; Iodamoeba_sp._RL1 | gut_resident |
| denovo2486 | Eukaryota; Amoebozoa; Archamoebae; Entamoebidae; Iodamoeba; Iodamoeba_sp._RL1 | gut_resident |
| denovo2485 | Eukaryota; Amoebozoa; Archamoebae; Entamoebidae; Iodamoeba; Iodamoeba_sp._RL1 | gut_resident |
| denovo2484 | Eukaryota; Amoebozoa; Archamoebae; Entamoebidae; Iodamoeba; Iodamoeba_sp._RL1 | gut_resident |
| denovo2483 | Eukaryota; Amoebozoa; Archamoebae; Entamoebidae; Iodamoeba; Iodamoeba_sp._RL1 | gut_resident |
| denovo2477 | Eukaryota; Amoebozoa; Archamoebae; Entamoebidae; Iodamoeba; Iodamoeba_sp._RL1 | gut_resident |
| denovo2474 | Eukaryota; Amoebozoa; Archamoebae; Entamoebidae; Iodamoeba; Iodamoeba_sp._RL1 | gut_resident |
| denovo2469 | Eukaryota; Amoebozoa; Archamoebae; Entamoebidae; Iodamoeba; Iodamoeba_sp._RL1 | gut_resident |
| denovo2466 | Eukaryota; Amoebozoa; Archamoebae; Entamoebidae; Iodamoeba; Iodamoeba_sp._RL1 | gut_resident |
| denovo2462 | Eukaryota; Amoebozoa; Archamoebae; Entamoebidae; Iodamoeba; Iodamoeba_sp._RL1 | gut_resident |
| denovo2461 | Eukaryota; Amoebozoa; Archamoebae; Entamoebidae; Iodamoeba; Iodamoeba_sp._RL1 | gut_resident |
| denovo2457 | Eukaryota; Amoebozoa; Archamoebae; Entamoebidae; Iodamoeba; Iodamoeba_sp._RL1 | gut_resident |
| denovo2441 | Eukaryota; Amoebozoa; Archamoebae; Entamoebidae; Iodamoeba; Iodamoeba_sp._RL1 | gut_resident |
| denovo2431 | Eukaryota; Amoebozoa; Archamoebae; Entamoebidae; Iodamoeba; Iodamoeba_sp._RL1 | gut_resident |
| denovo2426 | Eukaryota; Amoebozoa; Archamoebae; Entamoebidae; Iodamoeba; Iodamoeba_sp._RL1 | gut_resident |
| denovo2421 | Eukaryota; Amoebozoa; Archamoebae; Entamoebidae; Iodamoeba; Iodamoeba_sp._RL1 | gut_resident |
| denovo2414 | Eukaryota; Amoebozoa; Archamoebae; Entamoebidae; Iodamoeba; Iodamoeba_sp._RL1 | gut_resident |
| denovo2413 | Eukaryota; Amoebozoa; Archamoebae; Entamoebidae; Iodamoeba; Iodamoeba_sp._RL1 | gut_resident |
| denovo2412 | Eukaryota; Amoebozoa; Archamoebae; Entamoebidae; Iodamoeba; Iodamoeba_sp._RL1 | gut_resident |
| denovo2391 | Eukaryota; Amoebozoa; Archamoebae; Entamoebidae; Iodamoeba; Iodamoeba_sp._RL1 | gut_resident |
| denovo2389 | Eukaryota; Amoebozoa; Archamoebae; Entamoebidae; Iodamoeba; Iodamoeba_sp._RL1 | gut_resident |
| denovo2388 | Eukaryota; Amoebozoa; Archamoebae; Entamoebidae; Iodamoeba; Iodamoeba_sp._RL1 | gut_resident |
| denovo2379 | Eukaryota; Amoebozoa; Archamoebae; Entamoebidae; Iodamoeba; Iodamoeba_sp._RL1 | gut_resident |
| denovo2378 | Eukaryota; Amoebozoa; Archamoebae; Entamoebidae; Iodamoeba; Iodamoeba_sp._RL1 | gut_resident |
| denovo2370 | Eukaryota; Amoebozoa; Archamoebae; Entamoebidae; Iodamoeba; Iodamoeba_sp._RL1 | gut_resident |
| denovo2369 | Eukaryota; Amoebozoa; Archamoebae; Entamoebidae; Iodamoeba; Iodamoeba_sp._RL1 | gut_resident |

| | | |
|---|---|---|
| denovo2368 | Eukaryota; Amoebozoa; Archamoebae; Entamoebidae; Iodamoeba; Iodamoeba_sp._RL1 | gut_resident |
| denovo2367 | Eukaryota; Amoebozoa; Archamoebae; Entamoebidae; Iodamoeba; Iodamoeba_sp._RL1 | gut_resident |
| denovo2364 | Eukaryota; Amoebozoa; Archamoebae; Entamoebidae; Iodamoeba; Iodamoeba_sp._RL1 | gut_resident |
| denovo2363 | Eukaryota; Amoebozoa; Archamoebae; Entamoebidae; Iodamoeba; Iodamoeba_sp._RL1 | gut_resident |
| denovo2362 | Eukaryota; Amoebozoa; Archamoebae; Entamoebidae; Iodamoeba; Iodamoeba_sp._RL1 | gut_resident |
| denovo2359 | Eukaryota; Amoebozoa; Archamoebae; Entamoebidae; Iodamoeba; Iodamoeba_sp._RL1 | gut_resident |
| denovo2357 | Eukaryota; Amoebozoa; Archamoebae; Entamoebidae; Iodamoeba; Iodamoeba_sp._RL1 | gut_resident |
| denovo2356 | Eukaryota; Amoebozoa; Archamoebae; Entamoebidae; Iodamoeba; Iodamoeba_sp._RL1 | gut_resident |
| denovo2352 | Eukaryota; Amoebozoa; Archamoebae; Entamoebidae; Iodamoeba; Iodamoeba_sp._RL1 | gut_resident |
| denovo2351 | Eukaryota; Amoebozoa; Archamoebae; Entamoebidae; Iodamoeba; Iodamoeba_sp._RL1 | gut_resident |
| denovo2349 | Eukaryota; Amoebozoa; Archamoebae; Entamoebidae; Iodamoeba; Iodamoeba_sp._RL1 | gut_resident |
| denovo2347 | Eukaryota; Amoebozoa; Archamoebae; Entamoebidae; Iodamoeba; Iodamoeba_sp._RL1 | gut_resident |
| denovo2346 | Eukaryota; Amoebozoa; Archamoebae; Entamoebidae; Iodamoeba; Iodamoeba_sp._RL1 | gut_resident |
| denovo2339 | Eukaryota; Amoebozoa; Archamoebae; Entamoebidae; Iodamoeba; Iodamoeba_sp._RL1 | gut_resident |
| denovo2321 | Eukaryota; Amoebozoa; Archamoebae; Entamoebidae; Iodamoeba; Iodamoeba_sp._RL1 | gut_resident |
| denovo2320 | Eukaryota; Amoebozoa; Archamoebae; Entamoebidae; Iodamoeba; Iodamoeba_sp._RL1 | gut_resident |
| denovo2319 | Eukaryota; Amoebozoa; Archamoebae; Entamoebidae; Iodamoeba; Iodamoeba_sp._RL1 | gut_resident |
| denovo2318 | Eukaryota; Amoebozoa; Archamoebae; Entamoebidae; Iodamoeba; Iodamoeba_sp._RL1 | gut_resident |
| denovo231 | Eukaryota; Amoebozoa; Archamoebae; Entamoebidae; Iodamoeba; Iodamoeba_sp._RL1 | gut_resident |
| denovo2309 | Eukaryota; Amoebozoa; Archamoebae; Entamoebidae; Iodamoeba; Iodamoeba_sp._RL1 | gut_resident |
| denovo2304 | Eukaryota; Amoebozoa; Archamoebae; Entamoebidae; Iodamoeba; Iodamoeba_sp._RL1 | gut_resident |
| denovo2303 | Eukaryota; Amoebozoa; Archamoebae; Entamoebidae; Iodamoeba; Iodamoeba_sp._RL1 | gut_resident |
| denovo2302 | Eukaryota; Amoebozoa; Archamoebae; Entamoebidae; Iodamoeba; Iodamoeba_sp._RL1 | gut_resident |
| denovo208 | Eukaryota; Amoebozoa; Archamoebae; Entamoebidae; Iodamoeba; Iodamoeba_sp._RL1 | gut_resident |
| denovo214 | Eukaryota; Amoebozoa; Archamoebae; Entamoebidae; Iodamoeba; Iodamoeba_sp._RL1 | gut_resident |
| denovo224 | Eukaryota; Amoebozoa; Archamoebae; Entamoebidae; Iodamoeba; Iodamoeba_sp._RL1 | gut_resident |
| denovo223 | Eukaryota; Amoebozoa; Archamoebae; Entamoebidae; Iodamoeba; Iodamoeba_sp._RL1 | gut_resident |
| denovo228 | Eukaryota; Amoebozoa; Archamoebae; Entamoebidae; Iodamoeba; Iodamoeba_sp._RL1 | gut_resident |
| denovo172 | Eukaryota; Amoebozoa; Archamoebae; Entamoebidae; Iodamoeba; Iodamoeba_sp._RL1 | gut_resident |
| denovo169 | Eukaryota; Amoebozoa; Archamoebae; Entamoebidae; Iodamoeba; Iodamoeba_sp._RL1 | gut_resident |
| denovo176 | Eukaryota; Amoebozoa; Archamoebae; Entamoebidae; Iodamoeba; Iodamoeba_sp._RL1 | gut_resident |
| denovo1012 | Eukaryota; Amoebozoa; Archamoebae; Entamoebidae; Iodamoeba; Iodamoeba_sp._RL1 | gut_resident |
| denovo1049 | Eukaryota; Amoebozoa; Archamoebae; Entamoebidae; Iodamoeba; Iodamoeba_sp._RL1 | gut_resident |
| denovo1043 | Eukaryota; Amoebozoa; Archamoebae; Entamoebidae; Iodamoeba; Iodamoeba_sp._RL1 | gut_resident |
| denovo1036 | Eukaryota; Amoebozoa; Archamoebae; Entamoebidae; Iodamoeba; Iodamoeba_sp._RL1 | gut_resident |
| denovo1075 | Eukaryota; Amoebozoa; Archamoebae; Entamoebidae; Iodamoeba; Iodamoeba_sp._RL1 | gut_resident |
| denovo1072 | Eukaryota; Amoebozoa; Archamoebae; Entamoebidae; Iodamoeba; Iodamoeba_sp._RL1 | gut_resident |
| denovo1069 | Eukaryota; Amoebozoa; Archamoebae; Entamoebidae; Iodamoeba; Iodamoeba_sp._RL1 | gut_resident |
| denovo1066 | Eukaryota; Amoebozoa; Archamoebae; Entamoebidae; Iodamoeba; Iodamoeba_sp._RL1 | gut_resident |
| denovo1052 | Eukaryota; Amoebozoa; Archamoebae; Entamoebidae; Iodamoeba; Iodamoeba_sp._RL1 | gut_resident |

| | | |
|---|---|---|
| denovo1110 | Eukaryota; Amoebozoa; Archamoebae; Entamoebidae; Iodamoeba; Iodamoeba_sp._RL1 | gut_resident |
| denovo1100 | Eukaryota; Amoebozoa; Archamoebae; Entamoebidae; Iodamoeba; Iodamoeba_sp._RL1 | gut_resident |
| denovo1091 | Eukaryota; Amoebozoa; Archamoebae; Entamoebidae; Iodamoeba; Iodamoeba_sp._RL1 | gut_resident |
| denovo1124 | Eukaryota; Amoebozoa; Archamoebae; Entamoebidae; Iodamoeba; Iodamoeba_sp._RL1 | gut_resident |
| denovo1092 | Eukaryota; Amoebozoa; Archamoebae; Entamoebidae; Iodamoeba; Iodamoeba_sp._RL1 | gut_resident |
| denovo1086 | Eukaryota; Amoebozoa; Archamoebae; Entamoebidae; Iodamoeba; Iodamoeba_sp._RL1 | gut_resident |
| denovo1084 | Eukaryota; Amoebozoa; Archamoebae; Entamoebidae; Iodamoeba; Iodamoeba_sp._RL1 | gut_resident |
| denovo1141 | Eukaryota; Amoebozoa; Archamoebae; Entamoebidae; Iodamoeba; Iodamoeba_sp._RL1 | gut_resident |
| denovo1140 | Eukaryota; Amoebozoa; Archamoebae; Entamoebidae; Iodamoeba; Iodamoeba_sp._RL1 | gut_resident |
| denovo1132 | Eukaryota; Amoebozoa; Archamoebae; Entamoebidae; Iodamoeba; Iodamoeba_sp._RL1 | gut_resident |
| denovo1131 | Eukaryota; Amoebozoa; Archamoebae; Entamoebidae; Iodamoeba; Iodamoeba_sp._RL1 | gut_resident |
| denovo1128 | Eukaryota; Amoebozoa; Archamoebae; Entamoebidae; Iodamoeba; Iodamoeba_sp._RL1 | gut_resident |
| denovo1087 | Eukaryota; Amoebozoa; Archamoebae; Entamoebidae; Iodamoeba; Iodamoeba_sp._RL1 | gut_resident |
| denovo1176 | Eukaryota; Amoebozoa; Archamoebae; Entamoebidae; Iodamoeba; Iodamoeba_sp._RL1 | gut_resident |
| denovo1169 | Eukaryota; Amoebozoa; Archamoebae; Entamoebidae; Iodamoeba; Iodamoeba_sp._RL1 | gut_resident |
| denovo1159 | Eukaryota; Amoebozoa; Archamoebae; Entamoebidae; Iodamoeba; Iodamoeba_sp._RL1 | gut_resident |
| denovo1145 | Eukaryota; Amoebozoa; Archamoebae; Entamoebidae; Iodamoeba; Iodamoeba_sp._RL1 | gut_resident |
| denovo1139 | Eukaryota; Amoebozoa; Archamoebae; Entamoebidae; Iodamoeba; Iodamoeba_sp._RL1 | gut_resident |
| denovo1125 | Eukaryota; Amoebozoa; Archamoebae; Entamoebidae; Iodamoeba; Iodamoeba_sp._RL1 | gut_resident |
| denovo1123 | Eukaryota; Amoebozoa; Archamoebae; Entamoebidae; Iodamoeba; Iodamoeba_sp._RL1 | gut_resident |
| denovo1197 | Eukaryota; Amoebozoa; Archamoebae; Entamoebidae; Iodamoeba; Iodamoeba_sp._RL1 | gut_resident |
| denovo1161 | Eukaryota; Amoebozoa; Archamoebae; Entamoebidae; Iodamoeba; Iodamoeba_sp._RL1 | gut_resident |
| denovo1158 | Eukaryota; Amoebozoa; Archamoebae; Entamoebidae; Iodamoeba; Iodamoeba_sp._RL1 | gut_resident |
| denovo1152 | Eukaryota; Amoebozoa; Archamoebae; Entamoebidae; Iodamoeba; Iodamoeba_sp._RL1 | gut_resident |
| denovo1143 | Eukaryota; Amoebozoa; Archamoebae; Entamoebidae; Iodamoeba; Iodamoeba_sp._RL1 | gut_resident |
| denovo1206 | Eukaryota; Amoebozoa; Archamoebae; Entamoebidae; Iodamoeba; Iodamoeba_sp._RL1 | gut_resident |
| denovo1204 | Eukaryota; Amoebozoa; Archamoebae; Entamoebidae; Iodamoeba; Iodamoeba_sp._RL1 | gut_resident |
| denovo1184 | Eukaryota; Amoebozoa; Archamoebae; Entamoebidae; Iodamoeba; Iodamoeba_sp._RL1 | gut_resident |
| denovo1179 | Eukaryota; Amoebozoa; Archamoebae; Entamoebidae; Iodamoeba; Iodamoeba_sp._RL1 | gut_resident |
| denovo1175 | Eukaryota; Amoebozoa; Archamoebae; Entamoebidae; Iodamoeba; Iodamoeba_sp._RL1 | gut_resident |
| denovo1147 | Eukaryota; Amoebozoa; Archamoebae; Entamoebidae; Iodamoeba; Iodamoeba_sp._RL1 | gut_resident |
| denovo1217 | Eukaryota; Amoebozoa; Archamoebae; Entamoebidae; Iodamoeba; Iodamoeba_sp._RL1 | gut_resident |
| denovo1216 | Eukaryota; Amoebozoa; Archamoebae; Entamoebidae; Iodamoeba; Iodamoeba_sp._RL1 | gut_resident |
| denovo1214 | Eukaryota; Amoebozoa; Archamoebae; Entamoebidae; Iodamoeba; Iodamoeba_sp._RL1 | gut_resident |
| denovo1207 | Eukaryota; Amoebozoa; Archamoebae; Entamoebidae; Iodamoeba; Iodamoeba_sp._RL1 | gut_resident |
| denovo1205 | Eukaryota; Amoebozoa; Archamoebae; Entamoebidae; Iodamoeba; Iodamoeba_sp._RL1 | gut_resident |
| denovo1160 | Eukaryota; Amoebozoa; Archamoebae; Entamoebidae; Iodamoeba; Iodamoeba_sp._RL1 | gut_resident |
| denovo1148 | Eukaryota; Amoebozoa; Archamoebae; Entamoebidae; Iodamoeba; Iodamoeba_sp._RL1 | gut_resident |
| denovo1249 | Eukaryota; Amoebozoa; Archamoebae; Entamoebidae; Iodamoeba; Iodamoeba_sp._RL1 | gut_resident |
| denovo1242 | Eukaryota; Amoebozoa; Archamoebae; Entamoebidae; Iodamoeba; Iodamoeba_sp._RL1 | gut_resident |

| | | |
|---|---|---|
| denovo1196 | Eukaryota; Amoebozoa; Archamoebae; Entamoebidae; Iodamoeba; Iodamoeba_sp._RL1 | gut_resident |
| denovo1273 | Eukaryota; Amoebozoa; Archamoebae; Entamoebidae; Iodamoeba; Iodamoeba_sp._RL1 | gut_resident |
| denovo1272 | Eukaryota; Amoebozoa; Archamoebae; Entamoebidae; Iodamoeba; Iodamoeba_sp._RL1 | gut_resident |
| denovo1239 | Eukaryota; Amoebozoa; Archamoebae; Entamoebidae; Iodamoeba; Iodamoeba_sp._RL1 | gut_resident |
| denovo1225 | Eukaryota; Amoebozoa; Archamoebae; Entamoebidae; Iodamoeba; Iodamoeba_sp._RL1 | gut_resident |
| denovo1212 | Eukaryota; Amoebozoa; Archamoebae; Entamoebidae; Iodamoeba; Iodamoeba_sp._RL1 | gut_resident |
| denovo1191 | Eukaryota; Amoebozoa; Archamoebae; Entamoebidae; Iodamoeba; Iodamoeba_sp._RL1 | gut_resident |
| denovo1286 | Eukaryota; Amoebozoa; Archamoebae; Entamoebidae; Iodamoeba; Iodamoeba_sp._RL1 | gut_resident |
| denovo1325 | Eukaryota; Amoebozoa; Archamoebae; Entamoebidae; Iodamoeba; Iodamoeba_sp._RL1 | gut_resident |
| denovo1307 | Eukaryota; Amoebozoa; Archamoebae; Entamoebidae; Iodamoeba; Iodamoeba_sp._RL1 | gut_resident |
| denovo1298 | Eukaryota; Amoebozoa; Archamoebae; Entamoebidae; Iodamoeba; Iodamoeba_sp._RL1 | gut_resident |
| denovo1288 | Eukaryota; Amoebozoa; Archamoebae; Entamoebidae; Iodamoeba; Iodamoeba_sp._RL1 | gut_resident |
| denovo1282 | Eukaryota; Amoebozoa; Archamoebae; Entamoebidae; Iodamoeba; Iodamoeba_sp._RL1 | gut_resident |
| denovo1260 | Eukaryota; Amoebozoa; Archamoebae; Entamoebidae; Iodamoeba; Iodamoeba_sp._RL1 | gut_resident |
| denovo1258 | Eukaryota; Amoebozoa; Archamoebae; Entamoebidae; Iodamoeba; Iodamoeba_sp._RL1 | gut_resident |
| denovo1241 | Eukaryota; Amoebozoa; Archamoebae; Entamoebidae; Iodamoeba; Iodamoeba_sp._RL1 | gut_resident |
| denovo1238 | Eukaryota; Amoebozoa; Archamoebae; Entamoebidae; Iodamoeba; Iodamoeba_sp._RL1 | gut_resident |
| denovo1346 | Eukaryota; Amoebozoa; Archamoebae; Entamoebidae; Iodamoeba; Iodamoeba_sp._RL1 | gut_resident |
| denovo1331 | Eukaryota; Amoebozoa; Archamoebae; Entamoebidae; Iodamoeba; Iodamoeba_sp._RL1 | gut_resident |
| denovo1354 | Eukaryota; Amoebozoa; Archamoebae; Entamoebidae; Iodamoeba; Iodamoeba_sp._RL1 | gut_resident |
| denovo1351 | Eukaryota; Amoebozoa; Archamoebae; Entamoebidae; Iodamoeba; Iodamoeba_sp._RL1 | gut_resident |
| denovo1347 | Eukaryota; Amoebozoa; Archamoebae; Entamoebidae; Iodamoeba; Iodamoeba_sp._RL1 | gut_resident |
| denovo1338 | Eukaryota; Amoebozoa; Archamoebae; Entamoebidae; Iodamoeba; Iodamoeba_sp._RL1 | gut_resident |
| denovo1322 | Eukaryota; Amoebozoa; Archamoebae; Entamoebidae; Iodamoeba; Iodamoeba_sp._RL1 | gut_resident |
| denovo1316 | Eukaryota; Amoebozoa; Archamoebae; Entamoebidae; Iodamoeba; Iodamoeba_sp._RL1 | gut_resident |
| denovo1426 | Eukaryota; Amoebozoa; Archamoebae; Entamoebidae; Iodamoeba; Iodamoeba_sp._RL1 | gut_resident |
| denovo1425 | Eukaryota; Amoebozoa; Archamoebae; Entamoebidae; Iodamoeba; Iodamoeba_sp._RL1 | gut_resident |
| denovo1413 | Eukaryota; Amoebozoa; Archamoebae; Entamoebidae; Iodamoeba; Iodamoeba_sp._RL1 | gut_resident |
| denovo1394 | Eukaryota; Amoebozoa; Archamoebae; Entamoebidae; Iodamoeba; Iodamoeba_sp._RL1 | gut_resident |
| denovo1376 | Eukaryota; Amoebozoa; Archamoebae; Entamoebidae; Iodamoeba; Iodamoeba_sp._RL1 | gut_resident |
| denovo1364 | Eukaryota; Amoebozoa; Archamoebae; Entamoebidae; Iodamoeba; Iodamoeba_sp._RL1 | gut_resident |
| denovo1339 | Eukaryota; Amoebozoa; Archamoebae; Entamoebidae; Iodamoeba; Iodamoeba_sp._RL1 | gut_resident |
| denovo1330 | Eukaryota; Amoebozoa; Archamoebae; Entamoebidae; Iodamoeba; Iodamoeba_sp._RL1 | gut_resident |
| denovo1328 | Eukaryota; Amoebozoa; Archamoebae; Entamoebidae; Iodamoeba; Iodamoeba_sp._RL1 | gut_resident |
| denovo1299 | Eukaryota; Amoebozoa; Archamoebae; Entamoebidae; Iodamoeba; Iodamoeba_sp._RL1 | gut_resident |
| denovo1411 | Eukaryota; Amoebozoa; Archamoebae; Entamoebidae; Iodamoeba; Iodamoeba_sp._RL1 | gut_resident |
| denovo1410 | Eukaryota; Amoebozoa; Archamoebae; Entamoebidae; Iodamoeba; Iodamoeba_sp._RL1 | gut_resident |
| denovo1406 | Eukaryota; Amoebozoa; Archamoebae; Entamoebidae; Iodamoeba; Iodamoeba_sp._RL1 | gut_resident |
| denovo1399 | Eukaryota; Amoebozoa; Archamoebae; Entamoebidae; Iodamoeba; Iodamoeba_sp._RL1 | gut_resident |
| denovo1398 | Eukaryota; Amoebozoa; Archamoebae; Entamoebidae; Iodamoeba; Iodamoeba_sp._RL1 | gut_resident |

| | | |
|---|---|---|
| denovo1391 | Eukaryota; Amoebozoa; Archamoebae; Entamoebidae; Iodamoeba; Iodamoeba_sp._RL1 | gut_resident |
| denovo1373 | Eukaryota; Amoebozoa; Archamoebae; Entamoebidae; Iodamoeba; Iodamoeba_sp._RL1 | gut_resident |
| denovo1372 | Eukaryota; Amoebozoa; Archamoebae; Entamoebidae; Iodamoeba; Iodamoeba_sp._RL1 | gut_resident |
| denovo1363 | Eukaryota; Amoebozoa; Archamoebae; Entamoebidae; Iodamoeba; Iodamoeba_sp._RL1 | gut_resident |
| denovo1360 | Eukaryota; Amoebozoa; Archamoebae; Entamoebidae; Iodamoeba; Iodamoeba_sp._RL1 | gut_resident |
| denovo1301 | Eukaryota; Amoebozoa; Archamoebae; Entamoebidae; Iodamoeba; Iodamoeba_sp._RL1 | gut_resident |
| denovo1495 | Eukaryota; Amoebozoa; Archamoebae; Entamoebidae; Iodamoeba; Iodamoeba_sp._RL1 | gut_resident |
| denovo1491 | Eukaryota; Amoebozoa; Archamoebae; Entamoebidae; Iodamoeba; Iodamoeba_sp._RL1 | gut_resident |
| denovo1490 | Eukaryota; Amoebozoa; Archamoebae; Entamoebidae; Iodamoeba; Iodamoeba_sp._RL1 | gut_resident |
| denovo1475 | Eukaryota; Amoebozoa; Archamoebae; Entamoebidae; Iodamoeba; Iodamoeba_sp._RL1 | gut_resident |
| denovo1462 | Eukaryota; Amoebozoa; Archamoebae; Entamoebidae; Iodamoeba; Iodamoeba_sp._RL1 | gut_resident |
| denovo1461 | Eukaryota; Amoebozoa; Archamoebae; Entamoebidae; Iodamoeba; Iodamoeba_sp._RL1 | gut_resident |
| denovo1453 | Eukaryota; Amoebozoa; Archamoebae; Entamoebidae; Iodamoeba; Iodamoeba_sp._RL1 | gut_resident |
| denovo1447 | Eukaryota; Amoebozoa; Archamoebae; Entamoebidae; Iodamoeba; Iodamoeba_sp._RL1 | gut_resident |
| denovo1443 | Eukaryota; Amoebozoa; Archamoebae; Entamoebidae; Iodamoeba; Iodamoeba_sp._RL1 | gut_resident |
| denovo1432 | Eukaryota; Amoebozoa; Archamoebae; Entamoebidae; Iodamoeba; Iodamoeba_sp._RL1 | gut_resident |
| denovo1431 | Eukaryota; Amoebozoa; Archamoebae; Entamoebidae; Iodamoeba; Iodamoeba_sp._RL1 | gut_resident |
| denovo1430 | Eukaryota; Amoebozoa; Archamoebae; Entamoebidae; Iodamoeba; Iodamoeba_sp._RL1 | gut_resident |
| denovo1403 | Eukaryota; Amoebozoa; Archamoebae; Entamoebidae; Iodamoeba; Iodamoeba_sp._RL1 | gut_resident |
| denovo1400 | Eukaryota; Amoebozoa; Archamoebae; Entamoebidae; Iodamoeba; Iodamoeba_sp._RL1 | gut_resident |
| denovo1397 | Eukaryota; Amoebozoa; Archamoebae; Entamoebidae; Iodamoeba; Iodamoeba_sp._RL1 | gut_resident |
| denovo1359 | Eukaryota; Amoebozoa; Archamoebae; Entamoebidae; Iodamoeba; Iodamoeba_sp._RL1 | gut_resident |
| denovo1349 | Eukaryota; Amoebozoa; Archamoebae; Entamoebidae; Iodamoeba; Iodamoeba_sp._RL1 | gut_resident |
| denovo1531 | Eukaryota; Amoebozoa; Archamoebae; Entamoebidae; Iodamoeba; Iodamoeba_sp._RL1 | gut_resident |
| denovo1522 | Eukaryota; Amoebozoa; Archamoebae; Entamoebidae; Iodamoeba; Iodamoeba_sp._RL1 | gut_resident |
| denovo1518 | Eukaryota; Amoebozoa; Archamoebae; Entamoebidae; Iodamoeba; Iodamoeba_sp._RL1 | gut_resident |
| denovo1483 | Eukaryota; Amoebozoa; Archamoebae; Entamoebidae; Iodamoeba; Iodamoeba_sp._RL1 | gut_resident |
| denovo1463 | Eukaryota; Amoebozoa; Archamoebae; Entamoebidae; Iodamoeba; Iodamoeba_sp._RL1 | gut_resident |
| denovo1408 | Eukaryota; Amoebozoa; Archamoebae; Entamoebidae; Iodamoeba; Iodamoeba_sp._RL1 | gut_resident |
| denovo1401 | Eukaryota; Amoebozoa; Archamoebae; Entamoebidae; Iodamoeba; Iodamoeba_sp._RL1 | gut_resident |
| denovo1591 | Eukaryota; Amoebozoa; Archamoebae; Entamoebidae; Iodamoeba; Iodamoeba_sp._RL1 | gut_resident |
| denovo1585 | Eukaryota; Amoebozoa; Archamoebae; Entamoebidae; Iodamoeba; Iodamoeba_sp._RL1 | gut_resident |
| denovo1578 | Eukaryota; Amoebozoa; Archamoebae; Entamoebidae; Iodamoeba; Iodamoeba_sp._RL1 | gut_resident |
| denovo1577 | Eukaryota; Amoebozoa; Archamoebae; Entamoebidae; Iodamoeba; Iodamoeba_sp._RL1 | gut_resident |
| denovo1566 | Eukaryota; Amoebozoa; Archamoebae; Entamoebidae; Iodamoeba; Iodamoeba_sp._RL1 | gut_resident |
| denovo1538 | Eukaryota; Amoebozoa; Archamoebae; Entamoebidae; Iodamoeba; Iodamoeba_sp._RL1 | gut_resident |
| denovo1528 | Eukaryota; Amoebozoa; Archamoebae; Entamoebidae; Iodamoeba; Iodamoeba_sp._RL1 | gut_resident |
| denovo1527 | Eukaryota; Amoebozoa; Archamoebae; Entamoebidae; Iodamoeba; Iodamoeba_sp._RL1 | gut_resident |
| denovo1526 | Eukaryota; Amoebozoa; Archamoebae; Entamoebidae; Iodamoeba; Iodamoeba_sp._RL1 | gut_resident |
| denovo1523 | Eukaryota; Amoebozoa; Archamoebae; Entamoebidae; Iodamoeba; Iodamoeba_sp._RL1 | gut_resident |

| | | |
|---|---|---|
| denovo1497 | Eukaryota; Amoebozoa; Archamoebae; Entamoebidae; Iodamoeba; Iodamoeba_sp._RL1 | gut_resident |
| denovo1488 | Eukaryota; Amoebozoa; Archamoebae; Entamoebidae; Iodamoeba; Iodamoeba_sp._RL1 | gut_resident |
| denovo1486 | Eukaryota; Amoebozoa; Archamoebae; Entamoebidae; Iodamoeba; Iodamoeba_sp._RL1 | gut_resident |
| denovo1632 | Eukaryota; Amoebozoa; Archamoebae; Entamoebidae; Iodamoeba; Iodamoeba_sp._RL1 | gut_resident |
| denovo1631 | Eukaryota; Amoebozoa; Archamoebae; Entamoebidae; Iodamoeba; Iodamoeba_sp._RL1 | gut_resident |
| denovo1621 | Eukaryota; Amoebozoa; Archamoebae; Entamoebidae; Iodamoeba; Iodamoeba_sp._RL1 | gut_resident |
| denovo1620 | Eukaryota; Amoebozoa; Archamoebae; Entamoebidae; Iodamoeba; Iodamoeba_sp._RL1 | gut_resident |
| denovo1613 | Eukaryota; Amoebozoa; Archamoebae; Entamoebidae; Iodamoeba; Iodamoeba_sp._RL1 | gut_resident |
| denovo1575 | Eukaryota; Amoebozoa; Archamoebae; Entamoebidae; Iodamoeba; Iodamoeba_sp._RL1 | gut_resident |
| denovo1568 | Eukaryota; Amoebozoa; Archamoebae; Entamoebidae; Iodamoeba; Iodamoeba_sp._RL1 | gut_resident |
| denovo1559 | Eukaryota; Amoebozoa; Archamoebae; Entamoebidae; Iodamoeba; Iodamoeba_sp._RL1 | gut_resident |
| denovo1546 | Eukaryota; Amoebozoa; Archamoebae; Entamoebidae; Iodamoeba; Iodamoeba_sp._RL1 | gut_resident |
| denovo1534 | Eukaryota; Amoebozoa; Archamoebae; Entamoebidae; Iodamoeba; Iodamoeba_sp._RL1 | gut_resident |
| denovo1680 | Eukaryota; Amoebozoa; Archamoebae; Entamoebidae; Iodamoeba; Iodamoeba_sp._RL1 | gut_resident |
| denovo1665 | Eukaryota; Amoebozoa; Archamoebae; Entamoebidae; Iodamoeba; Iodamoeba_sp._RL1 | gut_resident |
| denovo1655 | Eukaryota; Amoebozoa; Archamoebae; Entamoebidae; Iodamoeba; Iodamoeba_sp._RL1 | gut_resident |
| denovo1635 | Eukaryota; Amoebozoa; Archamoebae; Entamoebidae; Iodamoeba; Iodamoeba_sp._RL1 | gut_resident |
| denovo1625 | Eukaryota; Amoebozoa; Archamoebae; Entamoebidae; Iodamoeba; Iodamoeba_sp._RL1 | gut_resident |
| denovo1619 | Eukaryota; Amoebozoa; Archamoebae; Entamoebidae; Iodamoeba; Iodamoeba_sp._RL1 | gut_resident |
| denovo1614 | Eukaryota; Amoebozoa; Archamoebae; Entamoebidae; Iodamoeba; Iodamoeba_sp._RL1 | gut_resident |
| denovo1610 | Eukaryota; Amoebozoa; Archamoebae; Entamoebidae; Iodamoeba; Iodamoeba_sp._RL1 | gut_resident |
| denovo1606 | Eukaryota; Amoebozoa; Archamoebae; Entamoebidae; Iodamoeba; Iodamoeba_sp._RL1 | gut_resident |
| denovo1593 | Eukaryota; Amoebozoa; Archamoebae; Entamoebidae; Iodamoeba; Iodamoeba_sp._RL1 | gut_resident |
| denovo1581 | Eukaryota; Amoebozoa; Archamoebae; Entamoebidae; Iodamoeba; Iodamoeba_sp._RL1 | gut_resident |
| denovo1579 | Eukaryota; Amoebozoa; Archamoebae; Entamoebidae; Iodamoeba; Iodamoeba_sp._RL1 | gut_resident |
| denovo1569 | Eukaryota; Amoebozoa; Archamoebae; Entamoebidae; Iodamoeba; Iodamoeba_sp._RL1 | gut_resident |
| denovo1567 | Eukaryota; Amoebozoa; Archamoebae; Entamoebidae; Iodamoeba; Iodamoeba_sp._RL1 | gut_resident |
| denovo1558 | Eukaryota; Amoebozoa; Archamoebae; Entamoebidae; Iodamoeba; Iodamoeba_sp._RL1 | gut_resident |
| denovo1535 | Eukaryota; Amoebozoa; Archamoebae; Entamoebidae; Iodamoeba; Iodamoeba_sp._RL1 | gut_resident |
| denovo1529 | Eukaryota; Amoebozoa; Archamoebae; Entamoebidae; Iodamoeba; Iodamoeba_sp._RL1 | gut_resident |
| denovo1746 | Eukaryota; Amoebozoa; Archamoebae; Entamoebidae; Iodamoeba; Iodamoeba_sp._RL1 | gut_resident |
| denovo1714 | Eukaryota; Amoebozoa; Archamoebae; Entamoebidae; Iodamoeba; Iodamoeba_sp._RL1 | gut_resident |
| denovo1710 | Eukaryota; Amoebozoa; Archamoebae; Entamoebidae; Iodamoeba; Iodamoeba_sp._RL1 | gut_resident |
| denovo1705 | Eukaryota; Amoebozoa; Archamoebae; Entamoebidae; Iodamoeba; Iodamoeba_sp._RL1 | gut_resident |
| denovo1703 | Eukaryota; Amoebozoa; Archamoebae; Entamoebidae; Iodamoeba; Iodamoeba_sp._RL1 | gut_resident |
| denovo1686 | Eukaryota; Amoebozoa; Archamoebae; Entamoebidae; Iodamoeba; Iodamoeba_sp._RL1 | gut_resident |
| denovo1669 | Eukaryota; Amoebozoa; Archamoebae; Entamoebidae; Iodamoeba; Iodamoeba_sp._RL1 | gut_resident |
| denovo1667 | Eukaryota; Amoebozoa; Archamoebae; Entamoebidae; Iodamoeba; Iodamoeba_sp._RL1 | gut_resident |
| denovo1658 | Eukaryota; Amoebozoa; Archamoebae; Entamoebidae; Iodamoeba; Iodamoeba_sp._RL1 | gut_resident |
| denovo1656 | Eukaryota; Amoebozoa; Archamoebae; Entamoebidae; Iodamoeba; Iodamoeba_sp._RL1 | gut_resident |

| | | |
|---|---|---|
| denovo1652 | Eukaryota; Amoebozoa; Archamoebae; Entamoebidae; Iodamoeba; Iodamoeba_sp._RL1 | gut_resident |
| denovo1617 | Eukaryota; Amoebozoa; Archamoebae; Entamoebidae; Iodamoeba; Iodamoeba_sp._RL1 | gut_resident |
| denovo1611 | Eukaryota; Amoebozoa; Archamoebae; Entamoebidae; Iodamoeba; Iodamoeba_sp._RL1 | gut_resident |
| denovo1572 | Eukaryota; Amoebozoa; Archamoebae; Entamoebidae; Iodamoeba; Iodamoeba_sp._RL1 | gut_resident |
| denovo1818 | Eukaryota; Amoebozoa; Archamoebae; Entamoebidae; Iodamoeba; Iodamoeba_sp._RL1 | gut_resident |
| denovo1797 | Eukaryota; Amoebozoa; Archamoebae; Entamoebidae; Iodamoeba; Iodamoeba_sp._RL1 | gut_resident |
| denovo1793 | Eukaryota; Amoebozoa; Archamoebae; Entamoebidae; Iodamoeba; Iodamoeba_sp._RL1 | gut_resident |
| denovo1792 | Eukaryota; Amoebozoa; Archamoebae; Entamoebidae; Iodamoeba; Iodamoeba_sp._RL1 | gut_resident |
| denovo1752 | Eukaryota; Amoebozoa; Archamoebae; Entamoebidae; Iodamoeba; Iodamoeba_sp._RL1 | gut_resident |
| denovo1702 | Eukaryota; Amoebozoa; Archamoebae; Entamoebidae; Iodamoeba; Iodamoeba_sp._RL1 | gut_resident |
| denovo1663 | Eukaryota; Amoebozoa; Archamoebae; Entamoebidae; Iodamoeba; Iodamoeba_sp._RL1 | gut_resident |
| denovo1662 | Eukaryota; Amoebozoa; Archamoebae; Entamoebidae; Iodamoeba; Iodamoeba_sp._RL1 | gut_resident |
| denovo1661 | Eukaryota; Amoebozoa; Archamoebae; Entamoebidae; Iodamoeba; Iodamoeba_sp._RL1 | gut_resident |
| denovo1659 | Eukaryota; Amoebozoa; Archamoebae; Entamoebidae; Iodamoeba; Iodamoeba_sp._RL1 | gut_resident |
| denovo1654 | Eukaryota; Amoebozoa; Archamoebae; Entamoebidae; Iodamoeba; Iodamoeba_sp._RL1 | gut_resident |
| denovo1616 | Eukaryota; Amoebozoa; Archamoebae; Entamoebidae; Iodamoeba; Iodamoeba_sp._RL1 | gut_resident |
| denovo1609 | Eukaryota; Amoebozoa; Archamoebae; Entamoebidae; Iodamoeba; Iodamoeba_sp._RL1 | gut_resident |
| denovo1853 | Eukaryota; Amoebozoa; Archamoebae; Entamoebidae; Iodamoeba; Iodamoeba_sp._RL1 | gut_resident |
| denovo1851 | Eukaryota; Amoebozoa; Archamoebae; Entamoebidae; Iodamoeba; Iodamoeba_sp._RL1 | gut_resident |
| denovo1835 | Eukaryota; Amoebozoa; Archamoebae; Entamoebidae; Iodamoeba; Iodamoeba_sp._RL1 | gut_resident |
| denovo1834 | Eukaryota; Amoebozoa; Archamoebae; Entamoebidae; Iodamoeba; Iodamoeba_sp._RL1 | gut_resident |
| denovo1832 | Eukaryota; Amoebozoa; Archamoebae; Entamoebidae; Iodamoeba; Iodamoeba_sp._RL1 | gut_resident |
| denovo1814 | Eukaryota; Amoebozoa; Archamoebae; Entamoebidae; Iodamoeba; Iodamoeba_sp._RL1 | gut_resident |
| denovo1768 | Eukaryota; Amoebozoa; Archamoebae; Entamoebidae; Iodamoeba; Iodamoeba_sp._RL1 | gut_resident |
| denovo1765 | Eukaryota; Amoebozoa; Archamoebae; Entamoebidae; Iodamoeba; Iodamoeba_sp._RL1 | gut_resident |
| denovo1760 | Eukaryota; Amoebozoa; Archamoebae; Entamoebidae; Iodamoeba; Iodamoeba_sp._RL1 | gut_resident |
| denovo1759 | Eukaryota; Amoebozoa; Archamoebae; Entamoebidae; Iodamoeba; Iodamoeba_sp._RL1 | gut_resident |
| denovo1753 | Eukaryota; Amoebozoa; Archamoebae; Entamoebidae; Iodamoeba; Iodamoeba_sp._RL1 | gut_resident |
| denovo1657 | Eukaryota; Amoebozoa; Archamoebae; Entamoebidae; Iodamoeba; Iodamoeba_sp._RL1 | gut_resident |
| denovo1953 | Eukaryota; Amoebozoa; Archamoebae; Entamoebidae; Iodamoeba; Iodamoeba_sp._RL1 | gut_resident |
| denovo1939 | Eukaryota; Amoebozoa; Archamoebae; Entamoebidae; Iodamoeba; Iodamoeba_sp._RL1 | gut_resident |
| denovo1930 | Eukaryota; Amoebozoa; Archamoebae; Entamoebidae; Iodamoeba; Iodamoeba_sp._RL1 | gut_resident |
| denovo1903 | Eukaryota; Amoebozoa; Archamoebae; Entamoebidae; Iodamoeba; Iodamoeba_sp._RL1 | gut_resident |
| denovo1896 | Eukaryota; Amoebozoa; Archamoebae; Entamoebidae; Iodamoeba; Iodamoeba_sp._RL1 | gut_resident |
| denovo1885 | Eukaryota; Amoebozoa; Archamoebae; Entamoebidae; Iodamoeba; Iodamoeba_sp._RL1 | gut_resident |
| denovo1858 | Eukaryota; Amoebozoa; Archamoebae; Entamoebidae; Iodamoeba; Iodamoeba_sp._RL1 | gut_resident |
| denovo1841 | Eukaryota; Amoebozoa; Archamoebae; Entamoebidae; Iodamoeba; Iodamoeba_sp._RL1 | gut_resident |
| denovo1836 | Eukaryota; Amoebozoa; Archamoebae; Entamoebidae; Iodamoeba; Iodamoeba_sp._RL1 | gut_resident |
| denovo1823 | Eukaryota; Amoebozoa; Archamoebae; Entamoebidae; Iodamoeba; Iodamoeba_sp._RL1 | gut_resident |
| denovo1811 | Eukaryota; Amoebozoa; Archamoebae; Entamoebidae; Iodamoeba; Iodamoeba_sp._RL1 | gut_resident |

| | | |
|---|---|---|
| denovo1785 | Eukaryota; Amoebozoa; Archamoebae; Entamoebidae; Iodamoeba; Iodamoeba_sp._RL1 | gut_resident |
| denovo1766 | Eukaryota; Amoebozoa; Archamoebae; Entamoebidae; Iodamoeba; Iodamoeba_sp._RL1 | gut_resident |
| denovo1707 | Eukaryota; Amoebozoa; Archamoebae; Entamoebidae; Iodamoeba; Iodamoeba_sp._RL1 | gut_resident |
| denovo1653 | Eukaryota; Amoebozoa; Archamoebae; Entamoebidae; Iodamoeba; Iodamoeba_sp._RL1 | gut_resident |
| denovo2029 | Eukaryota; Amoebozoa; Archamoebae; Entamoebidae; Iodamoeba; Iodamoeba_sp._RL1 | gut_resident |
| denovo2009 | Eukaryota; Amoebozoa; Archamoebae; Entamoebidae; Iodamoeba; Iodamoeba_sp._RL1 | gut_resident |
| denovo2006 | Eukaryota; Amoebozoa; Archamoebae; Entamoebidae; Iodamoeba; Iodamoeba_sp._RL1 | gut_resident |
| denovo1972 | Eukaryota; Amoebozoa; Archamoebae; Entamoebidae; Iodamoeba; Iodamoeba_sp._RL1 | gut_resident |
| denovo1916 | Eukaryota; Amoebozoa; Archamoebae; Entamoebidae; Iodamoeba; Iodamoeba_sp._RL1 | gut_resident |
| denovo1910 | Eukaryota; Amoebozoa; Archamoebae; Entamoebidae; Iodamoeba; Iodamoeba_sp._RL1 | gut_resident |
| denovo1909 | Eukaryota; Amoebozoa; Archamoebae; Entamoebidae; Iodamoeba; Iodamoeba_sp._RL1 | gut_resident |
| denovo1842 | Eukaryota; Amoebozoa; Archamoebae; Entamoebidae; Iodamoeba; Iodamoeba_sp._RL1 | gut_resident |
| denovo1837 | Eukaryota; Amoebozoa; Archamoebae; Entamoebidae; Iodamoeba; Iodamoeba_sp._RL1 | gut_resident |
| denovo1833 | Eukaryota; Amoebozoa; Archamoebae; Entamoebidae; Iodamoeba; Iodamoeba_sp._RL1 | gut_resident |
| denovo1831 | Eukaryota; Amoebozoa; Archamoebae; Entamoebidae; Iodamoeba; Iodamoeba_sp._RL1 | gut_resident |
| denovo1826 | Eukaryota; Amoebozoa; Archamoebae; Entamoebidae; Iodamoeba; Iodamoeba_sp._RL1 | gut_resident |
| denovo1771 | Eukaryota; Amoebozoa; Archamoebae; Entamoebidae; Iodamoeba; Iodamoeba_sp._RL1 | gut_resident |
| denovo1767 | Eukaryota; Amoebozoa; Archamoebae; Entamoebidae; Iodamoeba; Iodamoeba_sp._RL1 | gut_resident |
| denovo1724 | Eukaryota; Amoebozoa; Archamoebae; Entamoebidae; Iodamoeba; Iodamoeba_sp._RL1 | gut_resident |
| denovo2116 | Eukaryota; Amoebozoa; Archamoebae; Entamoebidae; Iodamoeba; Iodamoeba_sp._RL1 | gut_resident |
| denovo2071 | Eukaryota; Amoebozoa; Archamoebae; Entamoebidae; Iodamoeba; Iodamoeba_sp._RL1 | gut_resident |
| denovo2063 | Eukaryota; Amoebozoa; Archamoebae; Entamoebidae; Iodamoeba; Iodamoeba_sp._RL1 | gut_resident |
| denovo2060 | Eukaryota; Amoebozoa; Archamoebae; Entamoebidae; Iodamoeba; Iodamoeba_sp._RL1 | gut_resident |
| denovo2059 | Eukaryota; Amoebozoa; Archamoebae; Entamoebidae; Iodamoeba; Iodamoeba_sp._RL1 | gut_resident |
| denovo2054 | Eukaryota; Amoebozoa; Archamoebae; Entamoebidae; Iodamoeba; Iodamoeba_sp._RL1 | gut_resident |
| denovo2052 | Eukaryota; Amoebozoa; Archamoebae; Entamoebidae; Iodamoeba; Iodamoeba_sp._RL1 | gut_resident |
| denovo2048 | Eukaryota; Amoebozoa; Archamoebae; Entamoebidae; Iodamoeba; Iodamoeba_sp._RL1 | gut_resident |
| denovo2044 | Eukaryota; Amoebozoa; Archamoebae; Entamoebidae; Iodamoeba; Iodamoeba_sp._RL1 | gut_resident |
| denovo2041 | Eukaryota; Amoebozoa; Archamoebae; Entamoebidae; Iodamoeba; Iodamoeba_sp._RL1 | gut_resident |
| denovo2019 | Eukaryota; Amoebozoa; Archamoebae; Entamoebidae; Iodamoeba; Iodamoeba_sp._RL1 | gut_resident |
| denovo2008 | Eukaryota; Amoebozoa; Archamoebae; Entamoebidae; Iodamoeba; Iodamoeba_sp._RL1 | gut_resident |
| denovo2004 | Eukaryota; Amoebozoa; Archamoebae; Entamoebidae; Iodamoeba; Iodamoeba_sp._RL1 | gut_resident |
| denovo1964 | Eukaryota; Amoebozoa; Archamoebae; Entamoebidae; Iodamoeba; Iodamoeba_sp._RL1 | gut_resident |
| denovo1960 | Eukaryota; Amoebozoa; Archamoebae; Entamoebidae; Iodamoeba; Iodamoeba_sp._RL1 | gut_resident |
| denovo1915 | Eukaryota; Amoebozoa; Archamoebae; Entamoebidae; Iodamoeba; Iodamoeba_sp._RL1 | gut_resident |
| denovo1911 | Eukaryota; Amoebozoa; Archamoebae; Entamoebidae; Iodamoeba; Iodamoeba_sp._RL1 | gut_resident |
| denovo1904 | Eukaryota; Amoebozoa; Archamoebae; Entamoebidae; Iodamoeba; Iodamoeba_sp._RL1 | gut_resident |
| denovo1900 | Eukaryota; Amoebozoa; Archamoebae; Entamoebidae; Iodamoeba; Iodamoeba_sp._RL1 | gut_resident |
| denovo1898 | Eukaryota; Amoebozoa; Archamoebae; Entamoebidae; Iodamoeba; Iodamoeba_sp._RL1 | gut_resident |
| denovo1830 | Eukaryota; Amoebozoa; Archamoebae; Entamoebidae; Iodamoeba; Iodamoeba_sp._RL1 | gut_resident |

| | | |
|---|---|---|
| denovo2184 | Eukaryota; Amoebozoa; Archamoebae; Entamoebidae; Iodamoeba; Iodamoeba_sp._RL1 | gut_resident |
| denovo2174 | Eukaryota; Amoebozoa; Archamoebae; Entamoebidae; Iodamoeba; Iodamoeba_sp._RL1 | gut_resident |
| denovo2164 | Eukaryota; Amoebozoa; Archamoebae; Entamoebidae; Iodamoeba; Iodamoeba_sp._RL1 | gut_resident |
| denovo2161 | Eukaryota; Amoebozoa; Archamoebae; Entamoebidae; Iodamoeba; Iodamoeba_sp._RL1 | gut_resident |
| denovo2151 | Eukaryota; Amoebozoa; Archamoebae; Entamoebidae; Iodamoeba; Iodamoeba_sp._RL1 | gut_resident |
| denovo2117 | Eukaryota; Amoebozoa; Archamoebae; Entamoebidae; Iodamoeba; Iodamoeba_sp._RL1 | gut_resident |
| denovo2115 | Eukaryota; Amoebozoa; Archamoebae; Entamoebidae; Iodamoeba; Iodamoeba_sp._RL1 | gut_resident |
| denovo2103 | Eukaryota; Amoebozoa; Archamoebae; Entamoebidae; Iodamoeba; Iodamoeba_sp._RL1 | gut_resident |
| denovo2102 | Eukaryota; Amoebozoa; Archamoebae; Entamoebidae; Iodamoeba; Iodamoeba_sp._RL1 | gut_resident |
| denovo2050 | Eukaryota; Amoebozoa; Archamoebae; Entamoebidae; Iodamoeba; Iodamoeba_sp._RL1 | gut_resident |
| denovo2047 | Eukaryota; Amoebozoa; Archamoebae; Entamoebidae; Iodamoeba; Iodamoeba_sp._RL1 | gut_resident |
| denovo2043 | Eukaryota; Amoebozoa; Archamoebae; Entamoebidae; Iodamoeba; Iodamoeba_sp._RL1 | gut_resident |
| denovo1983 | Eukaryota; Amoebozoa; Archamoebae; Entamoebidae; Iodamoeba; Iodamoeba_sp._RL1 | gut_resident |
| denovo1962 | Eukaryota; Amoebozoa; Archamoebae; Entamoebidae; Iodamoeba; Iodamoeba_sp._RL1 | gut_resident |
| denovo1923 | Eukaryota; Amoebozoa; Archamoebae; Entamoebidae; Iodamoeba; Iodamoeba_sp._RL1 | gut_resident |
| denovo1825 | Eukaryota; Amoebozoa; Archamoebae; Entamoebidae; Iodamoeba; Iodamoeba_sp._RL1 | gut_resident |
| denovo2282 | Eukaryota; Amoebozoa; Archamoebae; Entamoebidae; Iodamoeba; Iodamoeba_sp._RL1 | gut_resident |
| denovo2271 | Eukaryota; Amoebozoa; Archamoebae; Entamoebidae; Iodamoeba; Iodamoeba_sp._RL1 | gut_resident |
| denovo2270 | Eukaryota; Amoebozoa; Archamoebae; Entamoebidae; Iodamoeba; Iodamoeba_sp._RL1 | gut_resident |
| denovo2259 | Eukaryota; Amoebozoa; Archamoebae; Entamoebidae; Iodamoeba; Iodamoeba_sp._RL1 | gut_resident |
| denovo2245 | Eukaryota; Amoebozoa; Archamoebae; Entamoebidae; Iodamoeba; Iodamoeba_sp._RL1 | gut_resident |
| denovo2243 | Eukaryota; Amoebozoa; Archamoebae; Entamoebidae; Iodamoeba; Iodamoeba_sp._RL1 | gut_resident |
| denovo2240 | Eukaryota; Amoebozoa; Archamoebae; Entamoebidae; Iodamoeba; Iodamoeba_sp._RL1 | gut_resident |
| denovo2210 | Eukaryota; Amoebozoa; Archamoebae; Entamoebidae; Iodamoeba; Iodamoeba_sp._RL1 | gut_resident |
| denovo2209 | Eukaryota; Amoebozoa; Archamoebae; Entamoebidae; Iodamoeba; Iodamoeba_sp._RL1 | gut_resident |
| denovo2208 | Eukaryota; Amoebozoa; Archamoebae; Entamoebidae; Iodamoeba; Iodamoeba_sp._RL1 | gut_resident |
| denovo2197 | Eukaryota; Amoebozoa; Archamoebae; Entamoebidae; Iodamoeba; Iodamoeba_sp._RL1 | gut_resident |
| denovo2166 | Eukaryota; Amoebozoa; Archamoebae; Entamoebidae; Iodamoeba; Iodamoeba_sp._RL1 | gut_resident |
| denovo2165 | Eukaryota; Amoebozoa; Archamoebae; Entamoebidae; Iodamoeba; Iodamoeba_sp._RL1 | gut_resident |
| denovo2157 | Eukaryota; Amoebozoa; Archamoebae; Entamoebidae; Iodamoeba; Iodamoeba_sp._RL1 | gut_resident |
| denovo2150 | Eukaryota; Amoebozoa; Archamoebae; Entamoebidae; Iodamoeba; Iodamoeba_sp._RL1 | gut_resident |
| denovo2139 | Eukaryota; Amoebozoa; Archamoebae; Entamoebidae; Iodamoeba; Iodamoeba_sp._RL1 | gut_resident |
| denovo2119 | Eukaryota; Amoebozoa; Archamoebae; Entamoebidae; Iodamoeba; Iodamoeba_sp._RL1 | gut_resident |
| denovo2104 | Eukaryota; Amoebozoa; Archamoebae; Entamoebidae; Iodamoeba; Iodamoeba_sp._RL1 | gut_resident |
| denovo2086 | Eukaryota; Amoebozoa; Archamoebae; Entamoebidae; Iodamoeba; Iodamoeba_sp._RL1 | gut_resident |
| denovo2069 | Eukaryota; Amoebozoa; Archamoebae; Entamoebidae; Iodamoeba; Iodamoeba_sp._RL1 | gut_resident |
| denovo2062 | Eukaryota; Amoebozoa; Archamoebae; Entamoebidae; Iodamoeba; Iodamoeba_sp._RL1 | gut_resident |
| denovo2030 | Eukaryota; Amoebozoa; Archamoebae; Entamoebidae; Iodamoeba; Iodamoeba_sp._RL1 | gut_resident |
| denovo1982 | Eukaryota; Amoebozoa; Archamoebae; Entamoebidae; Iodamoeba; Iodamoeba_sp._RL1 | gut_resident |
| denovo1981 | Eukaryota; Amoebozoa; Archamoebae; Entamoebidae; Iodamoeba; Iodamoeba_sp._RL1 | gut_resident |

| | | |
|---|---|---|
| denovo2283 | Eukaryota; Amoebozoa; Archamoebae; Entamoebidae; Iodamoeba; Iodamoeba_sp._RL1 | gut_resident |
| denovo2249 | Eukaryota; Amoebozoa; Archamoebae; Entamoebidae; Iodamoeba; Iodamoeba_sp._RL1 | gut_resident |
| denovo2234 | Eukaryota; Amoebozoa; Archamoebae; Entamoebidae; Iodamoeba; Iodamoeba_sp._RL1 | gut_resident |
| denovo2207 | Eukaryota; Amoebozoa; Archamoebae; Entamoebidae; Iodamoeba; Iodamoeba_sp._RL1 | gut_resident |
| denovo2177 | Eukaryota; Amoebozoa; Archamoebae; Entamoebidae; Iodamoeba; Iodamoeba_sp._RL1 | gut_resident |
| denovo2168 | Eukaryota; Amoebozoa; Archamoebae; Entamoebidae; Iodamoeba; Iodamoeba_sp._RL1 | gut_resident |
| denovo2153 | Eukaryota; Amoebozoa; Archamoebae; Entamoebidae; Iodamoeba; Iodamoeba_sp._RL1 | gut_resident |
| denovo2152 | Eukaryota; Amoebozoa; Archamoebae; Entamoebidae; Iodamoeba; Iodamoeba_sp._RL1 | gut_resident |
| denovo2061 | Eukaryota; Amoebozoa; Archamoebae; Entamoebidae; Iodamoeba; Iodamoeba_sp._RL1 | gut_resident |
| denovo2045 | Eukaryota; Amoebozoa; Archamoebae; Entamoebidae; Iodamoeba; Iodamoeba_sp._RL1 | gut_resident |
| denovo2233 | Eukaryota; Amoebozoa; Archamoebae; Entamoebidae; Iodamoeba; Iodamoeba_sp._RL1 | gut_resident |
| denovo2284 | Eukaryota; Amoebozoa; Archamoebae; Entamoebidae; Iodamoeba; Iodamoeba_sp._RL1 | gut_resident |
| denovo2244 | Eukaryota; Amoebozoa; Archamoebae; Entamoebidae; Iodamoeba; Iodamoeba_sp._RL1 | gut_resident |
| denovo2149 | Eukaryota; Amoebozoa; Archamoebae; Entamoebidae; Iodamoeba; Iodamoeba_sp._RL1 | gut_resident |
| denovo2146 | Eukaryota; Amoebozoa; Archamoebae; Entamoebidae; Iodamoeba; Iodamoeba_sp._RL1 | gut_resident |
| denovo2142 | Eukaryota; Amoebozoa; Archamoebae; Entamoebidae; Iodamoeba; Iodamoeba_sp._RL1 | gut_resident |
| denovo2187 | Eukaryota; Amoebozoa; Cavosteliida; uncultured; uncultured; uncultured; uncultured; uncultured_eukaryote | environmental |
| denovo2819 | Eukaryota; Amoebozoa; Dictyostelia; Dictyostelium; Dictyostelium; Dictyostelium; Dictyostelium | environmental |
| denovo405 | Eukaryota; Amoebozoa; Discosea; Flabellinia; Dactylopodida | environmental |
| denovo2633 | Eukaryota; Amoebozoa; Discosea; Flabellinia; Dactylopodida | environmental |
| denovo240 | Eukaryota; Amoebozoa; Discosea; Flabellinia; Dactylopodida | environmental |
| denovo2226 | Eukaryota; Amoebozoa; Gracilipodida; Filamoeba; Filamoeba; Filamoeba; Filamoeba | environmental |
| denovo2350 | Eukaryota; Amoebozoa; Gracilipodida; Flamella; Flamella; Flamella; Flamella; uncultured_Eimeriidae | environmental |
| denovo1383 | Eukaryota; Amoebozoa; Incertae_Sedis; Incertae_Sedis; Incertae_Sedis; Incertae_Sedis; Arboramoeba; uncultured_eukaryote | environmental |
| denovo2707 | Eukaryota; Amoebozoa; Incertae_Sedis; Incertae_Sedis; Incertae_Sedis; Incertae_Sedis; Ischnamoeba; Ischnamoeba_sp._FN352 | environmental |
| denovo2570 | Eukaryota; Amoebozoa; Incertae_Sedis; Incertae_Sedis; Incertae_Sedis; Incertae_Sedis; Ischnamoeba; Ischnamoeba_sp._FN352 | environmental |
| denovo1013 | Eukaryota; Amoebozoa; Incertae_Sedis; Incertae_Sedis; Incertae_Sedis; Incertae_Sedis; Ischnamoeba; Ischnamoeba_sp._FN352 | environmental |
| denovo2189 | Eukaryota; Amoebozoa; Incertae_Sedis; Incertae_Sedis; Incertae_Sedis; Incertae_Sedis; Ischnamoeba; Ischnamoeba_sp._FN352 | environmental |
| denovo2766 | Eukaryota; Amoebozoa; LEMD255; uncultured; uncultured; uncultured; uncultured; uncultured_Eimeriidae | environmental |
| denovo1741 | Eukaryota; Amoebozoa; Mycetozoa; Myxogastria | environmental |
| denovo1899 | Eukaryota; Amoebozoa; Mycetozoa; Myxogastria | environmental |
| denovo2147 | Eukaryota; Amoebozoa; Mycetozoa; Myxogastria | environmental |
| denovo2967 | Eukaryota; Amoebozoa; Mycetozoa; Myxogastria; Myxogastromycetidae; Physariida | environmental |
| denovo154 | Eukaryota; Amoebozoa; Mycetozoa; Myxogastria; Myxogastromycetidae; Physariida | environmental |
| denovo699 | Eukaryota; Amoebozoa; Mycetozoa; Myxogastria; Myxogastromycetidae; Physariida; Didymiaceae; Diderma; Diderma_crustaceum | environmental |
| denovo2475 | Eukaryota; Amoebozoa; Mycetozoa; Myxogastria; Myxogastromycetidae; Physariida; Didymiaceae; Didymium | environmental |
| denovo1456 | Eukaryota; Amoebozoa; Mycetozoa; Myxogastria; Myxogastromycetidae; Physariida; Didymiaceae; Didymium | environmental |
| denovo1905 | Eukaryota; Amoebozoa; Mycetozoa; Myxogastria; Myxogastromycetidae; Physariida; Didymiaceae; Didymium | environmental |
| denovo442 | Eukaryota; Amoebozoa; Mycetozoa; Myxogastria; Stemonitida; Stemonitidae; Lamproderma; Lamproderma_scintillans | environmental |
| denovo2392 | Eukaryota; Amoebozoa; Mycetozoa; Protosteliales | environmental |

| | | |
|---|---|---|
| denovo1054 | Eukaryota; Amoebozoa; Mycetozoa; Protosteliales | environmental |
| denovo2107 | Eukaryota; Amoebozoa; Mycetozoa; Protosteliales | environmental |
| denovo3126 | Eukaryota; Amoebozoa; Myxogastria; Didymium; Didymium; Didymium; Didymium | environmental |
| denovo2374 | Eukaryota; Amoebozoa; Myxogastria; Didymium; Didymium; Didymium; Didymium | environmental |
| denovo1919 | Eukaryota; Amoebozoa; Myxogastria; Didymium; Didymium; Didymium; Didymium | environmental |
| denovo1989 | Eukaryota; Amoebozoa; Myxogastria; Didymium; Didymium; Didymium; Didymium | environmental |
| denovo2810 | Eukaryota; Amoebozoa; Myxogastria; Didymium; Didymium; Didymium; Didymium; Didymium_iridis | environmental |
| denovo1448 | Eukaryota; Amoebozoa; Myxogastria; Didymium; Didymium; Didymium; Didymium; Didymium_iridis | environmental |
| denovo2252 | Eukaryota; Amoebozoa; Myxogastria; Didymium; Didymium; Didymium; Didymium; Didymium_sp._E3P | environmental |
| denovo2393 | Eukaryota; Amoebozoa; Schizoplasmodiida | environmental |
| denovo3198 | Eukaryota; Amoebozoa; Tubulinea | environmental |
| denovo3152 | Eukaryota; Amoebozoa; Tubulinea | environmental |
| denovo2999 | Eukaryota; Amoebozoa; Tubulinea | environmental |
| denovo2373 | Eukaryota; Amoebozoa; Tubulinea | environmental |
| denovo2669 | Eukaryota; Amoebozoa; Tubulinea; Arcellinida; Phryganellina; Cryptodifflugia; Cryptodifflugia | environmental |
| denovo1788 | Eukaryota; Amoebozoa; Tubulinea; Arcellinida; Phryganellina; Cryptodifflugia; Cryptodifflugia | environmental |
| denovo1068 | Eukaryota; Amoebozoa; Tubulinea; Euamoebida; BOLA868; BOLA868; BOLA868; uncultured_eukaryote | environmental |
| denovo3017 | Eukaryota; Amoebozoa; Tubulinea; Euamoebida; BOLA868; BOLA868; BOLA868; uncultured_Lobosea | environmental |
| denovo2425 | Eukaryota; Amoebozoa; Tubulinea; Euamoebida; BOLA868; BOLA868; BOLA868; uncultured_Lobosea | environmental |
| denovo1297 | Eukaryota; Amoebozoa; Tubulinea; Euamoebida; Copromyxa; Copromyxa; Copromyxa; Copromyxa_protea | environmental |
| denovo1850 | Eukaryota; Amoebozoa; Tubulinea; Euamoebida; Saccamoeba; Saccamoeba; Saccamoeba; Saccamoeba_limax | environmental |
| denovo2529 | Eukaryota; Amoebozoa; Tubulinea; Euamoebida; Saccamoeba; Saccamoeba; Saccamoeba; Saccamoeba_sp._DP7 | environmental |
| denovo2945 | Eukaryota; Amoebozoa; Tubulinea; Leptomyxida | environmental |
| denovo2201 | Eukaryota; Amoebozoa; Tubulinea; Leptomyxida; uncultured; uncultured; uncultured; uncultured_Eimeriidae | environmental |
| denovo2178 | Eukaryota; Apusomonadidae; Apusomonadidae; Apusomonas; Apusomonas; Apusomonas; Apusomonas; Apusomonas_proboscidea | environmental |
| denovo1859 | Eukaryota; Centrohelida; H15-6; Choanocystis; Choanocystis; Choanocystis; Choanocystis | environmental |
| denovo2965 | Eukaryota; Centrohelida; Heterophryidae; uncultured; uncultured; uncultured; uncultured; uncultured_eukaryote | environmental |
| denovo1589 | Eukaryota; Centrohelida; Heterophryidae; uncultured; uncultured; uncultured; uncultured; uncultured_eukaryote | environmental |
| denovo97 | Eukaryota; environmental_samples_<Eukaryotae>; uncultured_eukaryote | unknown |
| denovo969 | Eukaryota; environmental_samples_<Eukaryotae>; uncultured_eukaryote | unknown |
| denovo949 | Eukaryota; environmental_samples_<Eukaryotae>; uncultured_eukaryote | unknown |
| denovo924 | Eukaryota; environmental_samples_<Eukaryotae>; uncultured_eukaryote | unknown |
| denovo907 | Eukaryota; environmental_samples_<Eukaryotae>; uncultured_eukaryote | unknown |
| denovo87 | Eukaryota; environmental_samples_<Eukaryotae>; uncultured_eukaryote | unknown |
| denovo793 | Eukaryota; environmental_samples_<Eukaryotae>; uncultured_eukaryote | unknown |
| denovo774 | Eukaryota; environmental_samples_<Eukaryotae>; uncultured_eukaryote | unknown |
| denovo645 | Eukaryota; environmental_samples_<Eukaryotae>; uncultured_eukaryote | unknown |
| denovo636 | Eukaryota; environmental_samples_<Eukaryotae>; uncultured_eukaryote | unknown |
| denovo63 | Eukaryota; environmental_samples_<Eukaryotae>; uncultured_eukaryote | unknown |
| denovo623 | Eukaryota; environmental_samples_<Eukaryotae>; uncultured_eukaryote | unknown |

| | | |
|---|---|---|
| denovo590 | Eukaryota; environmental_samples_<Eukaryotae>; uncultured_eukaryote | unknown |
| denovo570 | Eukaryota; environmental_samples_<Eukaryotae>; uncultured_eukaryote | unknown |
| denovo553 | Eukaryota; environmental_samples_<Eukaryotae>; uncultured_eukaryote | unknown |
| denovo536 | Eukaryota; environmental_samples_<Eukaryotae>; uncultured_eukaryote | unknown |
| denovo496 | Eukaryota; environmental_samples_<Eukaryotae>; uncultured_eukaryote | unknown |
| denovo490 | Eukaryota; environmental_samples_<Eukaryotae>; uncultured_eukaryote | unknown |
| denovo478 | Eukaryota; environmental_samples_<Eukaryotae>; uncultured_eukaryote | unknown |
| denovo440 | Eukaryota; environmental_samples_<Eukaryotae>; uncultured_eukaryote | unknown |
| denovo385 | Eukaryota; environmental_samples_<Eukaryotae>; uncultured_eukaryote | unknown |
| denovo381 | Eukaryota; environmental_samples_<Eukaryotae>; uncultured_eukaryote | unknown |
| denovo335 | Eukaryota; environmental_samples_<Eukaryotae>; uncultured_eukaryote | unknown |
| denovo3252 | Eukaryota; environmental_samples_<Eukaryotae>; uncultured_eukaryote | unknown |
| denovo3202 | Eukaryota; environmental_samples_<Eukaryotae>; uncultured_eukaryote | unknown |
| denovo318 | Eukaryota; environmental_samples_<Eukaryotae>; uncultured_eukaryote | unknown |
| denovo3177 | Eukaryota; environmental_samples_<Eukaryotae>; uncultured_eukaryote | unknown |
| denovo3175 | Eukaryota; environmental_samples_<Eukaryotae>; uncultured_eukaryote | unknown |
| denovo317 | Eukaryota; environmental_samples_<Eukaryotae>; uncultured_eukaryote | unknown |
| denovo300 | Eukaryota; environmental_samples_<Eukaryotae>; uncultured_eukaryote | unknown |
| denovo2919 | Eukaryota; environmental_samples_<Eukaryotae>; uncultured_eukaryote | unknown |
| denovo2900 | Eukaryota; environmental_samples_<Eukaryotae>; uncultured_eukaryote | unknown |
| denovo2814 | Eukaryota; environmental_samples_<Eukaryotae>; uncultured_eukaryote | unknown |
| denovo280 | Eukaryota; environmental_samples_<Eukaryotae>; uncultured_eukaryote | unknown |
| denovo2797 | Eukaryota; environmental_samples_<Eukaryotae>; uncultured_eukaryote | unknown |
| denovo2759 | Eukaryota; environmental_samples_<Eukaryotae>; uncultured_eukaryote | unknown |
| denovo2725 | Eukaryota; environmental_samples_<Eukaryotae>; uncultured_eukaryote | unknown |
| denovo2708 | Eukaryota; environmental_samples_<Eukaryotae>; uncultured_eukaryote | unknown |
| denovo2703 | Eukaryota; environmental_samples_<Eukaryotae>; uncultured_eukaryote | unknown |
| denovo2620 | Eukaryota; environmental_samples_<Eukaryotae>; uncultured_eukaryote | unknown |
| denovo259 | Eukaryota; environmental_samples_<Eukaryotae>; uncultured_eukaryote | unknown |
| denovo2577 | Eukaryota; environmental_samples_<Eukaryotae>; uncultured_eukaryote | unknown |
| denovo2574 | Eukaryota; environmental_samples_<Eukaryotae>; uncultured_eukaryote | unknown |
| denovo2552 | Eukaryota; environmental_samples_<Eukaryotae>; uncultured_eukaryote | unknown |
| denovo2446 | Eukaryota; environmental_samples_<Eukaryotae>; uncultured_eukaryote | unknown |
| denovo2440 | Eukaryota; environmental_samples_<Eukaryotae>; uncultured_eukaryote | unknown |
| denovo2434 | Eukaryota; environmental_samples_<Eukaryotae>; uncultured_eukaryote | unknown |
| denovo242 | Eukaryota; environmental_samples_<Eukaryotae>; uncultured_eukaryote | unknown |
| denovo2307 | Eukaryota; environmental_samples_<Eukaryotae>; uncultured_eukaryote | unknown |
| denovo121 | Eukaryota; environmental_samples_<Eukaryotae>; uncultured_eukaryote | unknown |
| denovo156 | Eukaryota; environmental_samples_<Eukaryotae>; uncultured_eukaryote | unknown |
| denovo163 | Eukaryota; environmental_samples_<Eukaryotae>; uncultured_eukaryote | unknown |

| | | |
|---|---|---|
| denovo1023 | Eukaryota; environmental_samples_<Eukaryotae>; uncultured_eukaryote | unknown |
| denovo1047 | Eukaryota; environmental_samples_<Eukaryotae>; uncultured_eukaryote | unknown |
| denovo1038 | Eukaryota; environmental_samples_<Eukaryotae>; uncultured_eukaryote | unknown |
| denovo1080 | Eukaryota; environmental_samples_<Eukaryotae>; uncultured_eukaryote | unknown |
| denovo1118 | Eukaryota; environmental_samples_<Eukaryotae>; uncultured_eukaryote | unknown |
| denovo1199 | Eukaryota; environmental_samples_<Eukaryotae>; uncultured_eukaryote | unknown |
| denovo1230 | Eukaryota; environmental_samples_<Eukaryotae>; uncultured_eukaryote | unknown |
| denovo1308 | Eukaryota; environmental_samples_<Eukaryotae>; uncultured_eukaryote | unknown |
| denovo1306 | Eukaryota; environmental_samples_<Eukaryotae>; uncultured_eukaryote | unknown |
| denovo1385 | Eukaryota; environmental_samples_<Eukaryotae>; uncultured_eukaryote | unknown |
| denovo1436 | Eukaryota; environmental_samples_<Eukaryotae>; uncultured_eukaryote | unknown |
| denovo1428 | Eukaryota; environmental_samples_<Eukaryotae>; uncultured_eukaryote | unknown |
| denovo1416 | Eukaryota; environmental_samples_<Eukaryotae>; uncultured_eukaryote | unknown |
| denovo1477 | Eukaryota; environmental_samples_<Eukaryotae>; uncultured_eukaryote | unknown |
| denovo1503 | Eukaryota; environmental_samples_<Eukaryotae>; uncultured_eukaryote | unknown |
| denovo1501 | Eukaryota; environmental_samples_<Eukaryotae>; uncultured_eukaryote | unknown |
| denovo1594 | Eukaryota; environmental_samples_<Eukaryotae>; uncultured_eukaryote | unknown |
| denovo1642 | Eukaryota; environmental_samples_<Eukaryotae>; uncultured_eukaryote | unknown |
| denovo1634 | Eukaryota; environmental_samples_<Eukaryotae>; uncultured_eukaryote | unknown |
| denovo1689 | Eukaryota; environmental_samples_<Eukaryotae>; uncultured_eukaryote | unknown |
| denovo1821 | Eukaryota; environmental_samples_<Eukaryotae>; uncultured_eukaryote | unknown |
| denovo1791 | Eukaryota; environmental_samples_<Eukaryotae>; uncultured_eukaryote | unknown |
| denovo1868 | Eukaryota; environmental_samples_<Eukaryotae>; uncultured_eukaryote | unknown |
| denovo1863 | Eukaryota; environmental_samples_<Eukaryotae>; uncultured_eukaryote | unknown |
| denovo1848 | Eukaryota; environmental_samples_<Eukaryotae>; uncultured_eukaryote | unknown |
| denovo1918 | Eukaryota; environmental_samples_<Eukaryotae>; uncultured_eukaryote | unknown |
| denovo2037 | Eukaryota; environmental_samples_<Eukaryotae>; uncultured_eukaryote | unknown |
| denovo2024 | Eukaryota; environmental_samples_<Eukaryotae>; uncultured_eukaryote | unknown |
| denovo2011 | Eukaryota; environmental_samples_<Eukaryotae>; uncultured_eukaryote | unknown |
| denovo1963 | Eukaryota; environmental_samples_<Eukaryotae>; uncultured_eukaryote | unknown |
| denovo2121 | Eukaryota; environmental_samples_<Eukaryotae>; uncultured_eukaryote | unknown |
| denovo2112 | Eukaryota; environmental_samples_<Eukaryotae>; uncultured_eukaryote | unknown |
| denovo2092 | Eukaryota; environmental_samples_<Eukaryotae>; uncultured_eukaryote | unknown |
| denovo2221 | Eukaryota; environmental_samples_<Eukaryotae>; uncultured_eukaryote | unknown |
| denovo2183 | Eukaryota; environmental_samples_<Eukaryotae>; uncultured_eukaryote | unknown |
| denovo2294 | Eukaryota; environmental_samples_<Eukaryotae>; uncultured_eukaryote | unknown |
| denovo2288 | Eukaryota; environmental_samples_<Eukaryotae>; uncultured_eukaryote | unknown |
| denovo2246 | Eukaryota; environmental_samples_<Eukaryotae>; uncultured_eukaryote | unknown |
| denovo2942 | Eukaryota; Excavata; Discicristata; Heterolobosea; Tetramitia; Naegleria; Naegleria | environmental |
| denovo2959 | Eukaryota; Fornicata; Diplomonadida; Enteromonadidae; Enteromonas; Enteromonas_hominis | gut_resident |

| | | |
|---|---|---|
| denovo3194 | Eukaryota; Heterolobosea | environmental |
| denovo1452 | Eukaryota; Heterolobosea; Schizopyrenida; Vahlkampfiidae | environmental |
| denovo1721 | Eukaryota; Opisthokonta; Discicristoidea; Incertae_Sedis; Incertae_Sedis; Incertae_Sedis; uncultured; uncultured_fungus | environmental |
| denovo2581 | Eukaryota; Opisthokonta; Fungi | dietary/environmental |
| denovo2579 | Eukaryota; Opisthokonta; Fungi | dietary/environmental |
| denovo2126 | Eukaryota; Opisthokonta; Fungi | dietary/environmental |
| denovo643 | Eukaryota; Opisthokonta; Fungi; Ascomycota | dietary/environmental |
| denovo3274 | Eukaryota; Opisthokonta; Fungi; Ascomycota | dietary/environmental |
| denovo3272 | Eukaryota; Opisthokonta; Fungi; Ascomycota | dietary/environmental |
| denovo3266 | Eukaryota; Opisthokonta; Fungi; Ascomycota | dietary/environmental |
| denovo2867 | Eukaryota; Opisthokonta; Fungi; Ascomycota | dietary/environmental |
| denovo2528 | Eukaryota; Opisthokonta; Fungi; Ascomycota | dietary/environmental |
| denovo2398 | Eukaryota; Opisthokonta; Fungi; Ascomycota | dietary/environmental |
| denovo1588 | Eukaryota; Opisthokonta; Fungi; Ascomycota | dietary/environmental |
| denovo1695 | Eukaryota; Opisthokonta; Fungi; Ascomycota | dietary/environmental |
| denovo1784 | Eukaryota; Opisthokonta; Fungi; Ascomycota | dietary/environmental |
| denovo1778 | Eukaryota; Opisthokonta; Fungi; Ascomycota | dietary/environmental |
| denovo1952 | Eukaryota; Opisthokonta; Fungi; Ascomycota | dietary/environmental |
| denovo2293 | Eukaryota; Opisthokonta; Fungi; Ascomycota | dietary/environmental |
| denovo2268 | Eukaryota; Opisthokonta; Fungi; Ascomycota | dietary/environmental |
| denovo1042 | Eukaryota; Opisthokonta; Fungi; Ascomycota; Archaeorhizomycetes; Archaeorhizomycetales; Archaeorhizomyces | dietary/environmental |
| denovo1276 | Eukaryota; Opisthokonta; Fungi; Ascomycota; Archaeorhizomycetes; Archaeorhizomycetales; Archaeorhizomyces | dietary/environmental |
| denovo1735 | Eukaryota; Opisthokonta; Fungi; Ascomycota; Archaeorhizomycetes; Archaeorhizomycetales; Archaeorhizomyces | dietary/environmental |
| denovo2404 | Eukaryota; Opisthokonta; Fungi; Ascomycota; Arthoniomycetes; uncultured; uncultured | dietary/environmental |
| denovo990 | Eukaryota; Opisthokonta; Fungi; Ascomycota; Dothideomycetes | dietary/environmental |
| denovo694 | Eukaryota; Opisthokonta; Fungi; Ascomycota; Dothideomycetes | dietary/environmental |
| denovo632 | Eukaryota; Opisthokonta; Fungi; Ascomycota; Dothideomycetes | dietary/environmental |
| denovo2984 | Eukaryota; Opisthokonta; Fungi; Ascomycota; Dothideomycetes | dietary/environmental |
| denovo284 | Eukaryota; Opisthokonta; Fungi; Ascomycota; Dothideomycetes | dietary/environmental |
| denovo1552 | Eukaryota; Opisthokonta; Fungi; Ascomycota; Dothideomycetes | dietary/environmental |
| denovo1867 | Eukaryota; Opisthokonta; Fungi; Ascomycota; Dothideomycetes | dietary/environmental |
| denovo649 | Eukaryota; Opisthokonta; Fungi; Ascomycota; Dothideomycetes; Acrospermales; Acrospermum | dietary/environmental |
| denovo3067 | Eukaryota; Opisthokonta; Fungi; Ascomycota; Dothideomycetes; Acrospermales; Acrospermum | dietary/environmental |
| denovo1030 | Eukaryota; Opisthokonta; Fungi; Ascomycota; Dothideomycetes; Acrospermales; Acrospermum | dietary/environmental |
| denovo1675 | Eukaryota; Opisthokonta; Fungi; Ascomycota; Dothideomycetes; Acrospermales; Acrospermum | dietary/environmental |
| denovo118 | Eukaryota; Opisthokonta; Fungi; Ascomycota; Dothideomycetes; Botryosphaeriales | dietary/environmental |
| denovo652 | Eukaryota; Opisthokonta; Fungi; Ascomycota; Dothideomycetes; Botryosphaeriales; Botryosphaeria | dietary/environmental |
| denovo1539 | Eukaryota; Opisthokonta; Fungi; Ascomycota; Dothideomycetes; Botryosphaeriales; Guignardia | dietary/environmental |
| denovo873 | Eukaryota; Opisthokonta; Fungi; Ascomycota; Dothideomycetes; Botryosphaeriales; Phyllosticta | dietary/environmental |
| denovo2864 | Eukaryota; Opisthokonta; Fungi; Ascomycota; Dothideomycetes; Botryosphaeriales; Phyllosticta; Phyllosticta_cryptomeriae | plant_pathogen |

| | | |
|---|---|---|
| denovo441 | Eukaryota; Opisthokonta; Fungi; Ascomycota; Dothideomycetes; Botryosphaeriales; Phyllosticta; Phyllosticta_kobus | plant_pathogen |
| denovo955 | Eukaryota; Opisthokonta; Fungi; Ascomycota; Dothideomycetes; Capnodiales | dietary/environmental |
| denovo887 | Eukaryota; Opisthokonta; Fungi; Ascomycota; Dothideomycetes; Capnodiales | dietary/environmental |
| denovo821 | Eukaryota; Opisthokonta; Fungi; Ascomycota; Dothideomycetes; Capnodiales | dietary/environmental |
| denovo3209 | Eukaryota; Opisthokonta; Fungi; Ascomycota; Dothideomycetes; Capnodiales | dietary/environmental |
| denovo3171 | Eukaryota; Opisthokonta; Fungi; Ascomycota; Dothideomycetes; Capnodiales | dietary/environmental |
| denovo2879 | Eukaryota; Opisthokonta; Fungi; Ascomycota; Dothideomycetes; Capnodiales | dietary/environmental |
| denovo1506 | Eukaryota; Opisthokonta; Fungi; Ascomycota; Dothideomycetes; Capnodiales | dietary/environmental |
| denovo2204 | Eukaryota; Opisthokonta; Fungi; Ascomycota; Dothideomycetes; Capnodiales | dietary/environmental |
| denovo2193 | Eukaryota; Opisthokonta; Fungi; Ascomycota; Dothideomycetes; Capnodiales | dietary/environmental |
| denovo1454 | Eukaryota; Opisthokonta; Fungi; Ascomycota; Dothideomycetes; Capnodiales; Capnobotryella | dietary/environmental |
| denovo2416 | Eukaryota; Opisthokonta; Fungi; Ascomycota; Dothideomycetes; Capnodiales; Capnodiales | dietary/environmental |
| denovo1605 | Eukaryota; Opisthokonta; Fungi; Ascomycota; Dothideomycetes; Capnodiales; Capnodiales | dietary/environmental |
| denovo485 | Eukaryota; Opisthokonta; Fungi; Ascomycota; Dothideomycetes; Capnodiales; Cladosporium | dietary/environmental |
| denovo2134 | Eukaryota; Opisthokonta; Fungi; Ascomycota; Dothideomycetes; Capnodiales; Cladosporium | dietary/environmental |
| denovo68 | Eukaryota; Opisthokonta; Fungi; Ascomycota; Dothideomycetes; Capnodiales; Cladosporium; Cladosporium_herbarum | dietary/environmental |
| denovo808 | Eukaryota; Opisthokonta; Fungi; Ascomycota; Dothideomycetes; Capnodiales; Mangifera; Mangifera_indica_(mango) | dietary/environmental |
| denovo225 | Eukaryota; Opisthokonta; Fungi; Ascomycota; Dothideomycetes; Capnodiales; Mycosphaerella; Ramularia_endophylla | plant_pathogen |
| denovo239 | Eukaryota; Opisthokonta; Fungi; Ascomycota; Dothideomycetes; Capnodiales; Neocatenulostroma; Neocatenulostroma_microsporum | dietary/environmental |
| denovo384 | Eukaryota; Opisthokonta; Fungi; Ascomycota; Dothideomycetes; Capnodiales; Pseudocercospora | dietary/environmental |
| denovo1749 | Eukaryota; Opisthokonta; Fungi; Ascomycota; Dothideomycetes; Capnodiales; Schizothyrium; Schizothyrium_pomi | plant_pathogen |
| denovo951 | Eukaryota; Opisthokonta; Fungi; Ascomycota; Dothideomycetes; Capnodiales; Scorias; Scorias_spongiosa | dietary/environmental |
| denovo943 | Eukaryota; Opisthokonta; Fungi; Ascomycota; Dothideomycetes; Capnodiales; Scorias; Scorias_spongiosa | dietary/environmental |
| denovo561 | Eukaryota; Opisthokonta; Fungi; Ascomycota; Dothideomycetes; Capnodiales; Scorias; Scorias_spongiosa | dietary/environmental |
| denovo447 | Eukaryota; Opisthokonta; Fungi; Ascomycota; Dothideomycetes; Capnodiales; Scorias; Scorias_spongiosa | dietary/environmental |
| denovo1849 | Eukaryota; Opisthokonta; Fungi; Ascomycota; Dothideomycetes; Capnodiales; uncultured | dietary/environmental |
| denovo2081 | Eukaryota; Opisthokonta; Fungi; Ascomycota; Dothideomycetes; Capnodiales; uncultured | dietary/environmental |
| denovo1897 | Eukaryota; Opisthokonta; Fungi; Ascomycota; Dothideomycetes; Dothideales; Aureobasidium; Aureobasidium_pullulans | dietary/environmental |
| denovo1602 | Eukaryota; Opisthokonta; Fungi; Ascomycota; Dothideomycetes; Incertae_Sedis | dietary/environmental |
| denovo2192 | Eukaryota; Opisthokonta; Fungi; Ascomycota; Dothideomycetes; Incertae_Sedis; Cenococcum; Cenococcum_geophilum | dietary/environmental |
| denovo2437 | Eukaryota; Opisthokonta; Fungi; Ascomycota; Dothideomycetes; Incertae_Sedis; Cryomyces; Cryomyces_antarcticus | dietary/environmental |
| denovo1309 | Eukaryota; Opisthokonta; Fungi; Ascomycota; Dothideomycetes; Incertae_Sedis; Cryomyces; Cryomyces_antarcticus | dietary/environmental |
| denovo386 | Eukaryota; Opisthokonta; Fungi; Ascomycota; Dothideomycetes; Incertae_Sedis; Pseudochaetosphaeronema; Pseudochaetosphaeronema_larense | dietary/environmental |
| denovo1313 | Eukaryota; Opisthokonta; Fungi; Ascomycota; Dothideomycetes; Incertae_Sedis; Pseudochaetosphaeronema; Pseudochaetosphaeronema_larense | dietary/environmental |
| denovo686 | Eukaryota; Opisthokonta; Fungi; Ascomycota; Dothideomycetes; Incertae_Sedis; Wiesneriomyces; Wiesneriomyces_conjunctosporus | dietary/environmental |
| denovo1208 | Eukaryota; Opisthokonta; Fungi; Ascomycota; Dothideomycetes; Incertae_Sedis; Wiesneriomyces; Wiesneriomyces_conjunctosporus | dietary/environmental |
| denovo1633 | Eukaryota; Opisthokonta; Fungi; Ascomycota; Dothideomycetes; Incertae_Sedis; Wiesneriomyces; Wiesneriomyces_conjunctosporus | dietary/environmental |
| denovo920 | Eukaryota; Opisthokonta; Fungi; Ascomycota; Dothideomycetes; Myriangiales; Elsinoe | dietary/environmental |
| denovo3016 | Eukaryota; Opisthokonta; Fungi; Ascomycota; Dothideomycetes; Myriangiales; Elsinoe | dietary/environmental |
| denovo2511 | Eukaryota; Opisthokonta; Fungi; Ascomycota; Dothideomycetes; Myriangiales; Elsinoe | dietary/environmental |

| | | |
|---|---|---|
| denovo1966 | Eukaryota; Opisthokonta; Fungi; Ascomycota; Dothideomycetes; Myriangiales; Elsinoe | dietary/environmental |
| denovo981 | Eukaryota; Opisthokonta; Fungi; Ascomycota; Dothideomycetes; Pleosporales | dietary/environmental |
| denovo620 | Eukaryota; Opisthokonta; Fungi; Ascomycota; Dothideomycetes; Pleosporales | dietary/environmental |
| denovo459 | Eukaryota; Opisthokonta; Fungi; Ascomycota; Dothideomycetes; Pleosporales | dietary/environmental |
| denovo434 | Eukaryota; Opisthokonta; Fungi; Ascomycota; Dothideomycetes; Pleosporales | dietary/environmental |
| denovo342 | Eukaryota; Opisthokonta; Fungi; Ascomycota; Dothideomycetes; Pleosporales | dietary/environmental |
| denovo325 | Eukaryota; Opisthokonta; Fungi; Ascomycota; Dothideomycetes; Pleosporales | dietary/environmental |
| denovo2852 | Eukaryota; Opisthokonta; Fungi; Ascomycota; Dothideomycetes; Pleosporales | dietary/environmental |
| denovo2844 | Eukaryota; Opisthokonta; Fungi; Ascomycota; Dothideomycetes; Pleosporales | dietary/environmental |
| denovo2657 | Eukaryota; Opisthokonta; Fungi; Ascomycota; Dothideomycetes; Pleosporales | dietary/environmental |
| denovo2655 | Eukaryota; Opisthokonta; Fungi; Ascomycota; Dothideomycetes; Pleosporales | dietary/environmental |
| denovo2482 | Eukaryota; Opisthokonta; Fungi; Ascomycota; Dothideomycetes; Pleosporales | dietary/environmental |
| denovo238 | Eukaryota; Opisthokonta; Fungi; Ascomycota; Dothideomycetes; Pleosporales | dietary/environmental |
| denovo2336 | Eukaryota; Opisthokonta; Fungi; Ascomycota; Dothideomycetes; Pleosporales | dietary/environmental |
| denovo164 | Eukaryota; Opisthokonta; Fungi; Ascomycota; Dothideomycetes; Pleosporales | dietary/environmental |
| denovo1285 | Eukaryota; Opisthokonta; Fungi; Ascomycota; Dothideomycetes; Pleosporales | dietary/environmental |
| denovo1323 | Eukaryota; Opisthokonta; Fungi; Ascomycota; Dothideomycetes; Pleosporales | dietary/environmental |
| denovo1515 | Eukaryota; Opisthokonta; Fungi; Ascomycota; Dothideomycetes; Pleosporales | dietary/environmental |
| denovo1697 | Eukaryota; Opisthokonta; Fungi; Ascomycota; Dothideomycetes; Pleosporales | dietary/environmental |
| denovo1789 | Eukaryota; Opisthokonta; Fungi; Ascomycota; Dothideomycetes; Pleosporales | dietary/environmental |
| denovo1774 | Eukaryota; Opisthokonta; Fungi; Ascomycota; Dothideomycetes; Pleosporales | dietary/environmental |
| denovo2085 | Eukaryota; Opisthokonta; Fungi; Ascomycota; Dothideomycetes; Pleosporales | dietary/environmental |
| denovo1968 | Eukaryota; Opisthokonta; Fungi; Ascomycota; Dothideomycetes; Pleosporales | dietary/environmental |
| denovo290 | Eukaryota; Opisthokonta; Fungi; Ascomycota; Dothideomycetes; Pleosporales; Biatriospora | dietary/environmental |
| denovo1251 | Eukaryota; Opisthokonta; Fungi; Ascomycota; Dothideomycetes; Pleosporales; Biatriospora | dietary/environmental |
| denovo92 | Eukaryota; Opisthokonta; Fungi; Ascomycota; Dothideomycetes; Pleosporales; Boeremia; Boeremia_exigua_var._exigua | dietary/environmental |
| denovo3029 | Eukaryota; Opisthokonta; Fungi; Ascomycota; Dothideomycetes; Pleosporales; Cochliobolus; Curvularia_hawaiiensis | plant_pathogen |
| denovo568 | Eukaryota; Opisthokonta; Fungi; Ascomycota; Dothideomycetes; Pleosporales; Corynespora | dietary/environmental |
| denovo404 | Eukaryota; Opisthokonta; Fungi; Ascomycota; Dothideomycetes; Pleosporales; Helicoma | dietary/environmental |
| denovo1480 | Eukaryota; Opisthokonta; Fungi; Ascomycota; Dothideomycetes; Pleosporales; Helicomyces; Helicomyces_roseus | dietary/environmental |
| denovo2828 | Eukaryota; Opisthokonta; Fungi; Ascomycota; Dothideomycetes; Pleosporales; Massarina | dietary/environmental |
| denovo1003 | Eukaryota; Opisthokonta; Fungi; Ascomycota; Dothideomycetes; Pleosporales; Massarina | dietary/environmental |
| denovo2948 | Eukaryota; Opisthokonta; Fungi; Ascomycota; Dothideomycetes; Pleosporales; Neotestudina; Neotestudina_rosatii | dietary/environmental |
| denovo271 | Eukaryota; Opisthokonta; Fungi; Ascomycota; Dothideomycetes; Pleosporales; Neotestudina; Neotestudina_rosatii | dietary/environmental |
| denovo2229 | Eukaryota; Opisthokonta; Fungi; Ascomycota; Dothideomycetes; Pleosporales; Neotestudina; Neotestudina_rosatii | dietary/environmental |
| denovo835 | Eukaryota; Opisthokonta; Fungi; Ascomycota; Dothideomycetes; Pleosporales; Paraconiothyrium | dietary/environmental |
| denovo139 | Eukaryota; Opisthokonta; Fungi; Ascomycota; Dothideomycetes; Pleosporales; Paraconiothyrium | dietary/environmental |
| denovo3058 | Eukaryota; Opisthokonta; Fungi; Ascomycota; Dothideomycetes; Pleosporales; Paraphaeosphaeria; Paraphaeosphaeria_minitans | dietary/environmental |
| denovo962 | Eukaryota; Opisthokonta; Fungi; Ascomycota; Dothideomycetes; Pleosporales; Preussia | dietary/environmental |
| denovo895 | Eukaryota; Opisthokonta; Fungi; Ascomycota; Dothideomycetes; Pleosporales; Preussia | dietary/environmental |

| | | |
|---|---|---|
| denovo165 | Eukaryota; Opisthokonta; Fungi; Ascomycota; Dothideomycetes; Pleosporales; Preussia | dietary/environmental |
| denovo78 | Eukaryota; Opisthokonta; Fungi; Ascomycota; Dothideomycetes; Pleosporales; Preussia; Preussia_lignicola | dietary/environmental |
| denovo541 | Eukaryota; Opisthokonta; Fungi; Ascomycota; Dothideomycetes; Pleosporales; Preussia; Preussia_lignicola | dietary/environmental |
| denovo2624 | Eukaryota; Opisthokonta; Fungi; Ascomycota; Dothideomycetes; Pleosporales; Saccharicola | dietary/environmental |
| denovo373 | Eukaryota; Opisthokonta; Fungi; Ascomycota; Dothideomycetes; Pleosporales; Tubeufia; Tubeufia_cerea | dietary/environmental |
| denovo322 | Eukaryota; Opisthokonta; Fungi; Ascomycota; Dothideomycetes; Pleosporales; Tubeufia; Tubeufia_cerea | dietary/environmental |
| denovo989 | Eukaryota; Opisthokonta; Fungi; Ascomycota; Dothideomycetes; Pleosporales; uncultured | dietary/environmental |
| denovo297 | Eukaryota; Opisthokonta; Fungi; Ascomycota; Dothideomycetes; Pleosporales; uncultured | dietary/environmental |
| denovo1624 | Eukaryota; Opisthokonta; Fungi; Ascomycota; Dothideomycetes; Pleosporales; uncultured | dietary/environmental |
| denovo1587 | Eukaryota; Opisthokonta; Fungi; Ascomycota; Dothideomycetes; Pleosporales; uncultured | dietary/environmental |
| denovo1847 | Eukaryota; Opisthokonta; Fungi; Ascomycota; Dothideomycetes; Pleosporales; uncultured | dietary/environmental |
| denovo3287 | Eukaryota; Opisthokonta; Fungi; Ascomycota; Dothideomycetes; Pleosporales; Westerdykella | dietary/environmental |
| denovo885 | Eukaryota; Opisthokonta; Fungi; Ascomycota; Eurotiomycetes; Chaetothyriales | dietary/environmental |
| denovo764 | Eukaryota; Opisthokonta; Fungi; Ascomycota; Eurotiomycetes; Chaetothyriales | dietary/environmental |
| denovo678 | Eukaryota; Opisthokonta; Fungi; Ascomycota; Eurotiomycetes; Chaetothyriales | dietary/environmental |
| denovo338 | Eukaryota; Opisthokonta; Fungi; Ascomycota; Eurotiomycetes; Chaetothyriales | dietary/environmental |
| denovo2851 | Eukaryota; Opisthokonta; Fungi; Ascomycota; Eurotiomycetes; Chaetothyriales | dietary/environmental |
| denovo2850 | Eukaryota; Opisthokonta; Fungi; Ascomycota; Eurotiomycetes; Chaetothyriales | dietary/environmental |
| denovo2397 | Eukaryota; Opisthokonta; Fungi; Ascomycota; Eurotiomycetes; Chaetothyriales | dietary/environmental |
| denovo2301 | Eukaryota; Opisthokonta; Fungi; Ascomycota; Eurotiomycetes; Chaetothyriales | dietary/environmental |
| denovo146 | Eukaryota; Opisthokonta; Fungi; Ascomycota; Eurotiomycetes; Chaetothyriales | dietary/environmental |
| denovo226 | Eukaryota; Opisthokonta; Fungi; Ascomycota; Eurotiomycetes; Chaetothyriales | dietary/environmental |
| denovo1060 | Eukaryota; Opisthokonta; Fungi; Ascomycota; Eurotiomycetes; Chaetothyriales | dietary/environmental |
| denovo1076 | Eukaryota; Opisthokonta; Fungi; Ascomycota; Eurotiomycetes; Chaetothyriales | dietary/environmental |
| denovo1174 | Eukaryota; Opisthokonta; Fungi; Ascomycota; Eurotiomycetes; Chaetothyriales | dietary/environmental |
| denovo1476 | Eukaryota; Opisthokonta; Fungi; Ascomycota; Eurotiomycetes; Chaetothyriales | dietary/environmental |
| denovo1511 | Eukaryota; Opisthokonta; Fungi; Ascomycota; Eurotiomycetes; Chaetothyriales | dietary/environmental |
| denovo1717 | Eukaryota; Opisthokonta; Fungi; Ascomycota; Eurotiomycetes; Chaetothyriales | dietary/environmental |
| denovo2199 | Eukaryota; Opisthokonta; Fungi; Ascomycota; Eurotiomycetes; Chaetothyriales | dietary/environmental |
| denovo475 | Eukaryota; Opisthokonta; Fungi; Ascomycota; Eurotiomycetes; Chaetothyriales; Ascomycota | dietary/environmental |
| denovo2215 | Eukaryota; Opisthokonta; Fungi; Ascomycota; Eurotiomycetes; Chaetothyriales; Ascomycota | dietary/environmental |
| denovo935 | Eukaryota; Opisthokonta; Fungi; Ascomycota; Eurotiomycetes; Chaetothyriales; Ceramothyrium | dietary/environmental |
| denovo3065 | Eukaryota; Opisthokonta; Fungi; Ascomycota; Eurotiomycetes; Chaetothyriales; Ceramothyrium | dietary/environmental |
| denovo246 | Eukaryota; Opisthokonta; Fungi; Ascomycota; Eurotiomycetes; Chaetothyriales; Ceramothyrium | dietary/environmental |
| denovo1434 | Eukaryota; Opisthokonta; Fungi; Ascomycota; Eurotiomycetes; Chaetothyriales; Ceramothyrium | dietary/environmental |
| denovo1224 | Eukaryota; Opisthokonta; Fungi; Ascomycota; Eurotiomycetes; Chaetothyriales; Cladophialophora; Cladophialophora_chaetospira | dietary/environmental |
| denovo455 | Eukaryota; Opisthokonta; Fungi; Ascomycota; Eurotiomycetes; Chaetothyriales; Cyphellophora | dietary/environmental |
| denovo3297 | Eukaryota; Opisthokonta; Fungi; Ascomycota; Eurotiomycetes; Chaetothyriales; Cyphellophora | dietary/environmental |
| denovo315 | Eukaryota; Opisthokonta; Fungi; Ascomycota; Eurotiomycetes; Chaetothyriales; Cyphellophora | dietary/environmental |
| denovo3005 | Eukaryota; Opisthokonta; Fungi; Ascomycota; Eurotiomycetes; Chaetothyriales; Cyphellophora | dietary/environmental |

| | | |
|---|---|---|
| denovo270 | Eukaryota; Opisthokonta; Fungi; Ascomycota; Eurotiomycetes; Chaetothyriales; Cyphellophora | dietary/environmental |
| denovo1507 | Eukaryota; Opisthokonta; Fungi; Ascomycota; Eurotiomycetes; Chaetothyriales; Cyphellophora | dietary/environmental |
| denovo1681 | Eukaryota; Opisthokonta; Fungi; Ascomycota; Eurotiomycetes; Chaetothyriales; Cyphellophora | dietary/environmental |
| denovo2012 | Eukaryota; Opisthokonta; Fungi; Ascomycota; Eurotiomycetes; Chaetothyriales; Cyphellophora | dietary/environmental |
| denovo859 | Eukaryota; Opisthokonta; Fungi; Ascomycota; Eurotiomycetes; Chaetothyriales; Cyphellophora; Cyphellophora_reptans | dietary/environmental |
| denovo1541 | Eukaryota; Opisthokonta; Fungi; Ascomycota; Eurotiomycetes; Chaetothyriales; Cyphellophora; Cyphellophora_reptans | dietary/environmental |
| denovo1754 | Eukaryota; Opisthokonta; Fungi; Ascomycota; Eurotiomycetes; Chaetothyriales; Cyphellophora; Cyphellophora_reptans | dietary/environmental |
| denovo626 | Eukaryota; Opisthokonta; Fungi; Ascomycota; Eurotiomycetes; Chaetothyriales; Exophiala | dietary/environmental |
| denovo2908 | Eukaryota; Opisthokonta; Fungi; Ascomycota; Eurotiomycetes; Chaetothyriales; Exophiala | dietary/environmental |
| denovo221 | Eukaryota; Opisthokonta; Fungi; Ascomycota; Eurotiomycetes; Chaetothyriales; Exophiala | dietary/environmental |
| denovo1109 | Eukaryota; Opisthokonta; Fungi; Ascomycota; Eurotiomycetes; Chaetothyriales; Exophiala; Exophiala_dermatitidis | dietary/environmental |
| denovo2257 | Eukaryota; Opisthokonta; Fungi; Ascomycota; Eurotiomycetes; Chaetothyriales; Phialophora; Phialophora_verrucosa | dietary/environmental |
| denovo1344 | Eukaryota; Opisthokonta; Fungi; Ascomycota; Eurotiomycetes; Chaetothyriales; Rhinocladiella; Rhinocladiella_phaeophora | dietary/environmental |
| denovo840 | Eukaryota; Opisthokonta; Fungi; Ascomycota; Eurotiomycetes; Chaetothyriales; Sarcinomyces; Sarcinomyces_sp._SL-2011 | dietary/environmental |
| denovo2080 | Eukaryota; Opisthokonta; Fungi; Ascomycota; Eurotiomycetes; Chaetothyriales; uncultured | dietary/environmental |
| denovo2713 | Eukaryota; Opisthokonta; Fungi; Ascomycota; Eurotiomycetes; Chaetothyriales; uncultured; uncultured_Ascomycota | dietary/environmental |
| denovo2871 | Eukaryota; Opisthokonta; Fungi; Ascomycota; Eurotiomycetes; Chaetothyriales; uncultured; uncultured_fungus | dietary/environmental |
| denovo1018 | Eukaryota; Opisthokonta; Fungi; Ascomycota; Eurotiomycetes; Coryneliales; Caliciopsis; Caliciopsis_pinea | plant_pathogen |
| denovo961 | Eukaryota; Opisthokonta; Fungi; Ascomycota; Eurotiomycetes; Eurotiales | dietary/environmental |
| denovo9 | Eukaryota; Opisthokonta; Fungi; Ascomycota; Eurotiomycetes; Eurotiales | dietary/environmental |
| denovo2455 | Eukaryota; Opisthokonta; Fungi; Ascomycota; Eurotiomycetes; Eurotiales | dietary/environmental |
| denovo1312 | Eukaryota; Opisthokonta; Fungi; Ascomycota; Eurotiomycetes; Eurotiales | dietary/environmental |
| denovo1764 | Eukaryota; Opisthokonta; Fungi; Ascomycota; Eurotiomycetes; Eurotiales | dietary/environmental |
| denovo842 | Eukaryota; Opisthokonta; Fungi; Ascomycota; Eurotiomycetes; Eurotiales; Aspergillus | dietary/environmental_possible_pathogen |
| denovo813 | Eukaryota; Opisthokonta; Fungi; Ascomycota; Eurotiomycetes; Eurotiales; Aspergillus | dietary/environmental_possible_pathogen |
| denovo8 | Eukaryota; Opisthokonta; Fungi; Ascomycota; Eurotiomycetes; Eurotiales; Aspergillus | dietary/environmental_possible_pathogen |
| denovo538 | Eukaryota; Opisthokonta; Fungi; Ascomycota; Eurotiomycetes; Eurotiales; Aspergillus | dietary/environmental_possible_pathogen |
| denovo366 | Eukaryota; Opisthokonta; Fungi; Ascomycota; Eurotiomycetes; Eurotiales; Aspergillus | dietary/environmental_possible_pathogen |
| denovo3159 | Eukaryota; Opisthokonta; Fungi; Ascomycota; Eurotiomycetes; Eurotiales; Aspergillus | dietary/environmental_possible_pathogen |
| denovo3155 | Eukaryota; Opisthokonta; Fungi; Ascomycota; Eurotiomycetes; Eurotiales; Aspergillus | dietary/environmental_possible_pathogen |
| denovo256 | Eukaryota; Opisthokonta; Fungi; Ascomycota; Eurotiomycetes; Eurotiales; Aspergillus | dietary/environmental_possible_pathogen |
| denovo2384 | Eukaryota; Opisthokonta; Fungi; Ascomycota; Eurotiomycetes; Eurotiales; Aspergillus | dietary/environmental_possible_pathogen |
| denovo1146 | Eukaryota; Opisthokonta; Fungi; Ascomycota; Eurotiomycetes; Eurotiales; Aspergillus | dietary/environmental_possible_pathogen |
| denovo1525 | Eukaryota; Opisthokonta; Fungi; Ascomycota; Eurotiomycetes; Eurotiales; Aspergillus | dietary/environmental_possible_pathogen |
| denovo705 | Eukaryota; Opisthokonta; Fungi; Ascomycota; Eurotiomycetes; Eurotiales; Aspergillus; Aspergillus_penicillioides | dietary/environmental_possible_pathogen |
| denovo350 | Eukaryota; Opisthokonta; Fungi; Ascomycota; Eurotiomycetes; Eurotiales; Aspergillus; Aspergillus_penicillioides | dietary/environmental_possible_pathogen |
| denovo2825 | Eukaryota; Opisthokonta; Fungi; Ascomycota; Eurotiomycetes; Eurotiales; Aspergillus; Aspergillus_penicillioides | dietary/environmental_possible_pathogen |
| denovo275 | Eukaryota; Opisthokonta; Fungi; Ascomycota; Eurotiomycetes; Eurotiales; Aspergillus; Aspergillus_penicillioides | dietary/environmental_possible_pathogen |
| denovo2580 | Eukaryota; Opisthokonta; Fungi; Ascomycota; Eurotiomycetes; Eurotiales; Aspergillus; Aspergillus_penicillioides | dietary/environmental_possible_pathogen |
| denovo1022 | Eukaryota; Opisthokonta; Fungi; Ascomycota; Eurotiomycetes; Eurotiales; Aspergillus; Aspergillus_penicillioides | dietary/environmental_possible_pathogen |

| | | |
|---|---|---|
| denovo1226 | Eukaryota; Opisthokonta; Fungi; Ascomycota; Eurotiomycetes; Eurotiales; Aspergillus; Aspergillus_penicillioides | dietary/environmental_possible_pathogen |
| denovo2088 | Eukaryota; Opisthokonta; Fungi; Ascomycota; Eurotiomycetes; Eurotiales; Aspergillus; Aspergillus_penicillioides | dietary/environmental_possible_pathogen |
| denovo1977 | Eukaryota; Opisthokonta; Fungi; Ascomycota; Eurotiomycetes; Eurotiales; Aspergillus; Aspergillus_penicillioides | dietary/environmental_possible_pathogen |
| denovo2155 | Eukaryota; Opisthokonta; Fungi; Ascomycota; Eurotiomycetes; Eurotiales; Aspergillus; Aspergillus_penicillioides | dietary/environmental_possible_pathogen |
| denovo3095 | Eukaryota; Opisthokonta; Fungi; Ascomycota; Eurotiomycetes; Eurotiales; Monascus | dietary/environmental |
| denovo704 | Eukaryota; Opisthokonta; Fungi; Ascomycota; Eurotiomycetes; Eurotiales; Penicillium | dietary/environmental |
| denovo47 | Eukaryota; Opisthokonta; Fungi; Ascomycota; Eurotiomycetes; Eurotiales; Penicillium | dietary/environmental |
| denovo452 | Eukaryota; Opisthokonta; Fungi; Ascomycota; Eurotiomycetes; Eurotiales; Penicillium | dietary/environmental |
| denovo1377 | Eukaryota; Opisthokonta; Fungi; Ascomycota; Eurotiomycetes; Eurotiales; Penicillium | dietary/environmental |
| denovo985 | Eukaryota; Opisthokonta; Fungi; Ascomycota; Eurotiomycetes; Incertae_Sedis; Knufia; Knufia_cryptophialidica | plant_pathogen |
| denovo497 | Eukaryota; Opisthokonta; Fungi; Ascomycota; Eurotiomycetes; Incertae_Sedis; Knufia; Knufia_cryptophialidica | plant_pathogen |
| denovo2353 | Eukaryota; Opisthokonta; Fungi; Ascomycota; Eurotiomycetes; Incertae_Sedis; Knufia; Knufia_cryptophialidica | plant_pathogen |
| denovo1967 | Eukaryota; Opisthokonta; Fungi; Ascomycota; Eurotiomycetes; Incertae_Sedis; Knufia; Knufia_cryptophialidica | plant_pathogen |
| denovo1813 | Eukaryota; Opisthokonta; Fungi; Ascomycota; Eurotiomycetes; Incertae_Sedis; Knufia; Knufia_petricola | dietary/environmental |
| denovo1932 | Eukaryota; Opisthokonta; Fungi; Ascomycota; Eurotiomycetes; Onygenales | dietary/environmental |
| denovo2656 | Eukaryota; Opisthokonta; Fungi; Ascomycota; Eurotiomycetes; Onygenales; Auxarthron | dietary/environmental |
| denovo1976 | Eukaryota; Opisthokonta; Fungi; Ascomycota; Eurotiomycetes; Onygenales; Auxarthron; Auxarthron_zuffianum | dietary/environmental |
| denovo3196 | Eukaryota; Opisthokonta; Fungi; Ascomycota; Incertae_Sedis; Incertae_Sedis; Exochalara; Exochalara_longissima | dietary/environmental |
| denovo2386 | Eukaryota; Opisthokonta; Fungi; Ascomycota; Incertae_Sedis; Incertae_Sedis; Exochalara; Exochalara_longissima | dietary/environmental |
| denovo639 | Eukaryota; Opisthokonta; Fungi; Ascomycota; Incertae_Sedis; Incertae_Sedis; Heliocephala | dietary/environmental |
| denovo3244 | Eukaryota; Opisthokonta; Fungi; Ascomycota; Incertae_Sedis; Incertae_Sedis; Heliocephala | dietary/environmental |
| denovo3154 | Eukaryota; Opisthokonta; Fungi; Ascomycota; Incertae_Sedis; Incertae_Sedis; Microcyclospora | dietary/environmental |
| denovo1062 | Eukaryota; Opisthokonta; Fungi; Ascomycota; Incertae_Sedis; Incertae_Sedis; Microcyclospora | dietary/environmental |
| denovo566 | Eukaryota; Opisthokonta; Fungi; Ascomycota; Incertae_Sedis; Incertae_Sedis; Ochroconis | dietary/environmental |
| denovo3242 | Eukaryota; Opisthokonta; Fungi; Ascomycota; Incertae_Sedis; Incertae_Sedis; Ochroconis | dietary/environmental |
| denovo2906 | Eukaryota; Opisthokonta; Fungi; Ascomycota; Incertae_Sedis; Incertae_Sedis; Ochroconis | dietary/environmental |
| denovo2730 | Eukaryota; Opisthokonta; Fungi; Ascomycota; Incertae_Sedis; Incertae_Sedis; Ochroconis | dietary/environmental |
| denovo1782 | Eukaryota; Opisthokonta; Fungi; Ascomycota; Incertae_Sedis; Incertae_Sedis; Ochroconis | dietary/environmental |
| denovo1925 | Eukaryota; Opisthokonta; Fungi; Ascomycota; Incertae_Sedis; Incertae_Sedis; Ochroconis | dietary/environmental |
| denovo2273 | Eukaryota; Opisthokonta; Fungi; Ascomycota; Incertae_Sedis; Incertae_Sedis; Ochroconis | dietary/environmental |
| denovo1120 | Eukaryota; Opisthokonta; Fungi; Ascomycota; Incertae_Sedis; Incertae_Sedis; Ochroconis; Ochroconis_cordanae | dietary/environmental |
| denovo425 | Eukaryota; Opisthokonta; Fungi; Ascomycota; Lecanoromycetes; Lecanorales | dietary/environmental |
| denovo3047 | Eukaryota; Opisthokonta; Fungi; Ascomycota; Lecanoromycetes; Lecanorales | dietary/environmental |
| denovo2401 | Eukaryota; Opisthokonta; Fungi; Ascomycota; Lecanoromycetes; Lecanorales | dietary/environmental |
| denovo2396 | Eukaryota; Opisthokonta; Fungi; Ascomycota; Lecanoromycetes; Lecanorales | dietary/environmental |
| denovo217 | Eukaryota; Opisthokonta; Fungi; Ascomycota; Lecanoromycetes; Lecanorales | dietary/environmental |
| denovo1844 | Eukaryota; Opisthokonta; Fungi; Ascomycota; Lecanoromycetes; Lecanorales | dietary/environmental |
| denovo1646 | Eukaryota; Opisthokonta; Fungi; Ascomycota; Lecanoromycetes; Lecanorales; Xanthoparmelia; Xanthoparmelia_conspersa | dietary/environmental |
| denovo967 | Eukaryota; Opisthokonta; Fungi; Ascomycota; Leotiomycetes; Helotiales | dietary/environmental |
| denovo355 | Eukaryota; Opisthokonta; Fungi; Ascomycota; Leotiomycetes; Helotiales | dietary/environmental |

| | | |
|---|---|---|
| denovo3319 | Eukaryota; Opisthokonta; Fungi; Ascomycota; Leotiomycetes; Helotiales | dietary/environmental |
| denovo3188 | Eukaryota; Opisthokonta; Fungi; Ascomycota; Leotiomycetes; Helotiales | dietary/environmental |
| denovo2376 | Eukaryota; Opisthokonta; Fungi; Ascomycota; Leotiomycetes; Helotiales | dietary/environmental |
| denovo1944 | Eukaryota; Opisthokonta; Fungi; Ascomycota; Leotiomycetes; Helotiales | dietary/environmental |
| denovo844 | Eukaryota; Opisthokonta; Fungi; Ascomycota; Leotiomycetes; Helotiales; Hyaloscypha; Hyaloscypha_aff._paludosa_A_TL-2012 | dietary/environmental |
| denovo1553 | Eukaryota; Opisthokonta; Fungi; Ascomycota; Leotiomycetes; Helotiales; Tricladium; Tricladium_splendens | dietary/environmental |
| denovo1317 | Eukaryota; Opisthokonta; Fungi; Ascomycota; Leotiomycetes; Helotiales; uncultured; unidentified | dietary/environmental |
| denovo1427 | Eukaryota; Opisthokonta; Fungi; Ascomycota; Leotiomycetes; Helotiales; uncultured; unidentified | dietary/environmental |
| denovo2478 | Eukaryota; Opisthokonta; Fungi; Ascomycota; Leotiomycetes; Incertae_Sedis | dietary/environmental |
| denovo2829 | Eukaryota; Opisthokonta; Fungi; Ascomycota; Leotiomycetes; Incertae_Sedis; Pseudogymnoascus | dietary/environmental |
| denovo3304 | Eukaryota; Opisthokonta; Fungi; Ascomycota; Leotiomycetes; Incertae_Sedis; Tetracladium | dietary/environmental |
| denovo3102 | Eukaryota; Opisthokonta; Fungi; Ascomycota; Leotiomycetes; Incertae_Sedis; Tetracladium | dietary/environmental |
| denovo112 | Eukaryota; Opisthokonta; Fungi; Ascomycota; Leotiomycetes; Incertae_Sedis; Tetracladium; Tetracladium_marchalianum | dietary/environmental |
| denovo380 | Eukaryota; Opisthokonta; Fungi; Ascomycota; Leotiomycetes; Thelebolales; Thelebolus; Thelebolus_stercoreus | dietary/environmental |
| denovo204 | Eukaryota; Opisthokonta; Fungi; Ascomycota; Leotiomycetes; Thelebolales; Thelebolus; Thelebolus_stercoreus | dietary/environmental |
| denovo1074 | Eukaryota; Opisthokonta; Fungi; Ascomycota; Lichinomycetes; Lichinales | dietary/environmental |
| denovo2175 | Eukaryota; Opisthokonta; Fungi; Ascomycota; Lichinomycetes; Lichinales; Peltula | dietary/environmental |
| denovo2216 | Eukaryota; Opisthokonta; Fungi; Ascomycota; Lichinomycetes; Lichinales; Phloeopeccania; Phloeopeccania_pulvinulina | dietary/environmental |
| denovo3001 | Eukaryota; Opisthokonta; Fungi; Ascomycota; Orbiliomycetes; Orbiliales | dietary/environmental |
| denovo1265 | Eukaryota; Opisthokonta; Fungi; Ascomycota; Orbiliomycetes; Orbiliales | dietary/environmental |
| denovo1839 | Eukaryota; Opisthokonta; Fungi; Ascomycota; Orbiliomycetes; Orbiliales | dietary/environmental |
| denovo2074 | Eukaryota; Opisthokonta; Fungi; Ascomycota; Orbiliomycetes; Orbiliales; Dactylella; Dactylella_oxyspora | dietary/environmental |
| denovo2076 | Eukaryota; Opisthokonta; Fungi; Ascomycota; Orbiliomycetes; Orbiliales; uncultured; uncultured_Orbiliaceae | dietary/environmental |
| denovo1050 | Eukaryota; Opisthokonta; Fungi; Ascomycota; Pezizomycetes; Incertae_Sedis; Lecophagus | dietary/environmental |
| denovo2186 | Eukaryota; Opisthokonta; Fungi; Ascomycota; Pezizomycetes; Incertae_Sedis; Lecophagus | dietary/environmental |
| denovo2285 | Eukaryota; Opisthokonta; Fungi; Ascomycota; Pezizomycetes; Incertae_Sedis; Lecophagus | dietary/environmental |
| denovo936 | Eukaryota; Opisthokonta; Fungi; Ascomycota; Pezizomycetes; Incertae_Sedis; Lecophagus; uncultured_fungal_contaminant | dietary/environmental |
| denovo2827 | Eukaryota; Opisthokonta; Fungi; Ascomycota; Pezizomycetes; Incertae_Sedis; Lecophagus; uncultured_fungal_contaminant | dietary/environmental |
| denovo2322 | Eukaryota; Opisthokonta; Fungi; Ascomycota; Pezizomycetes; Incertae_Sedis; Lecophagus; uncultured_fungal_contaminant | dietary/environmental |
| denovo2072 | Eukaryota; Opisthokonta; Fungi; Ascomycota; Pezizomycetes; Incertae_Sedis; Lecophagus; uncultured_fungal_contaminant | dietary/environmental |
| denovo479 | Eukaryota; Opisthokonta; Fungi; Ascomycota; Pezizomycetes; Pezizales | dietary/environmental |
| denovo396 | Eukaryota; Opisthokonta; Fungi; Ascomycota; Pezizomycetes; Pezizales | dietary/environmental |
| denovo312 | Eukaryota; Opisthokonta; Fungi; Ascomycota; Pezizomycetes; Pezizales | dietary/environmental |
| denovo1945 | Eukaryota; Opisthokonta; Fungi; Ascomycota; Pezizomycetes; Pezizales | dietary/environmental |
| denovo1090 | Eukaryota; Opisthokonta; Fungi; Ascomycota; Pezizomycetes; Pezizales; Aleuria; Aleuria_aurantia_(orange_peel_mushroom) | dietary/environmental |
| denovo611 | Eukaryota; Opisthokonta; Fungi; Ascomycota; Pezizomycetes; Pezizales; Ascobolus | dietary/environmental |
| denovo1556 | Eukaryota; Opisthokonta; Fungi; Ascomycota; Pezizomycetes; Pezizales; Peziza | dietary/environmental |
| denovo1348 | Eukaryota; Opisthokonta; Fungi; Ascomycota; Pezizomycetes; Pezizales; Pyronema | dietary/environmental |
| denovo451 | Eukaryota; Opisthokonta; Fungi; Ascomycota; Saccharomycetes | dietary/environmental |
| denovo1912 | Eukaryota; Opisthokonta; Fungi; Ascomycota; Saccharomycetes | dietary/environmental |

| | | |
|---|---|---|
| denovo291 | Eukaryota; Opisthokonta; Fungi; Ascomycota; Saccharomycetes; Incertae_Sedis | dietary/environmental |
| denovo1470 | Eukaryota; Opisthokonta; Fungi; Ascomycota; Saccharomycetes; Incertae_Sedis | dietary/environmental |
| denovo775 | Eukaryota; Opisthokonta; Fungi; Ascomycota; Saccharomycetes; Incertae_Sedis; Candida | dietary/environmental |
| denovo2904 | Eukaryota; Opisthokonta; Fungi; Ascomycota; Saccharomycetes; Incertae_Sedis; Candida | dietary/environmental |
| denovo2809 | Eukaryota; Opisthokonta; Fungi; Ascomycota; Saccharomycetes; Incertae_Sedis; Candida | dietary/environmental |
| denovo1130 | Eukaryota; Opisthokonta; Fungi; Ascomycota; Saccharomycetes; Incertae_Sedis; Candida | dietary/environmental |
| denovo1469 | Eukaryota; Opisthokonta; Fungi; Ascomycota; Saccharomycetes; Incertae_Sedis; Candida | dietary/environmental |
| denovo1499 | Eukaryota; Opisthokonta; Fungi; Ascomycota; Saccharomycetes; Incertae_Sedis; Candida | dietary/environmental |
| denovo1555 | Eukaryota; Opisthokonta; Fungi; Ascomycota; Saccharomycetes; Incertae_Sedis; Candida | dietary/environmental |
| denovo3303 | Eukaryota; Opisthokonta; Fungi; Ascomycota; Saccharomycetes; Incertae_Sedis; Candida; Candida_allociferrii | dietary/environmental |
| denovo153 | Eukaryota; Opisthokonta; Fungi; Ascomycota; Saccharomycetes; Incertae_Sedis; Candida; Candida_fructus | plant_pathogen |
| denovo305 | Eukaryota; Opisthokonta; Fungi; Ascomycota; Saccharomycetes; Incertae_Sedis; Candida; Candida_intermedia | dietary/environmental |
| denovo2093 | Eukaryota; Opisthokonta; Fungi; Ascomycota; Saccharomycetes; Incertae_Sedis; Candida; Candida_montana | dietary/environmental |
| denovo784 | Eukaryota; Opisthokonta; Fungi; Ascomycota; Saccharomycetes; Incertae_Sedis; Candida; Candida_nitratophila | dietary/environmental |
| denovo3267 | Eukaryota; Opisthokonta; Fungi; Ascomycota; Saccharomycetes; Incertae_Sedis; Candida; Candida_odintsovae | dietary/environmental |
| denovo2518 | Eukaryota; Opisthokonta; Fungi; Ascomycota; Saccharomycetes; Incertae_Sedis; Candida; Saccharomycopsis_amapae | dietary/environmental |
| denovo220 | Eukaryota; Opisthokonta; Fungi; Ascomycota; Saccharomycetes; Incertae_Sedis; Candida; Saturnispora_diversa | dietary/environmental |
| denovo71 | Eukaryota; Opisthokonta; Fungi; Ascomycota; Saccharomycetes; Incertae_Sedis; Cyberlindnera | dietary/environmental |
| denovo1182 | Eukaryota; Opisthokonta; Fungi; Ascomycota; Saccharomycetes; Incertae_Sedis; Cyberlindnera | dietary/environmental |
| denovo505 | Eukaryota; Opisthokonta; Fungi; Ascomycota; Saccharomycetes; Incertae_Sedis; Cyberlindnera; Cyberlindnera_saturnus | dietary/environmental |
| denovo285 | Eukaryota; Opisthokonta; Fungi; Ascomycota; Saccharomycetes; Incertae_Sedis; Cyberlindnera; Cyberlindnera_saturnus | dietary/environmental |
| denovo847 | Eukaryota; Opisthokonta; Fungi; Ascomycota; Saccharomycetes; Saccharomycetales | dietary/environmental |
| denovo43 | Eukaryota; Opisthokonta; Fungi; Ascomycota; Saccharomycetes; Saccharomycetales | dietary/environmental |
| denovo2452 | Eukaryota; Opisthokonta; Fungi; Ascomycota; Saccharomycetes; Saccharomycetales | dietary/environmental |
| denovo2366 | Eukaryota; Opisthokonta; Fungi; Ascomycota; Saccharomycetes; Saccharomycetales | dietary/environmental |
| denovo1007 | Eukaryota; Opisthokonta; Fungi; Ascomycota; Saccharomycetes; Saccharomycetales | dietary/environmental |
| denovo1730 | Eukaryota; Opisthokonta; Fungi; Ascomycota; Saccharomycetes; Saccharomycetales | dietary/environmental |
| denovo1878 | Eukaryota; Opisthokonta; Fungi; Ascomycota; Saccharomycetes; Saccharomycetales; Barnettozyma | dietary/environmental |
| denovo471 | Eukaryota; Opisthokonta; Fungi; Ascomycota; Saccharomycetes; Saccharomycetales; Candida | dietary/environmental |
| denovo3022 | Eukaryota; Opisthokonta; Fungi; Ascomycota; Saccharomycetes; Saccharomycetales; Candida | dietary/environmental |
| denovo1796 | Eukaryota; Opisthokonta; Fungi; Ascomycota; Saccharomycetes; Saccharomycetales; Candida | dietary/environmental |
| denovo276 | Eukaryota; Opisthokonta; Fungi; Ascomycota; Saccharomycetes; Saccharomycetales; Candida; Candida_sp._NRRL_YB-2243 | dietary/environmental |
| denovo460 | Eukaryota; Opisthokonta; Fungi; Ascomycota; Saccharomycetes; Saccharomycetales; Candida; Candida_sp._NRRL_YB-3031 | dietary/environmental |
| denovo105 | Eukaryota; Opisthokonta; Fungi; Ascomycota; Saccharomycetes; Saccharomycetales; Cyniclomyces; Cyniclomyces_guttulatus | dietary/environmental |
| denovo2354 | Eukaryota; Opisthokonta; Fungi; Ascomycota; Saccharomycetes; Saccharomycetales; Debaryomyces | dietary/environmental |
| denovo1607 | Eukaryota; Opisthokonta; Fungi; Ascomycota; Saccharomycetes; Saccharomycetales; Debaryomyces | dietary/environmental |
| denovo205 | Eukaryota; Opisthokonta; Fungi; Ascomycota; Saccharomycetes; Saccharomycetales; Debaryomyces; Debaryomyces_hansenii | dietary/environmental |
| denovo1820 | Eukaryota; Opisthokonta; Fungi; Ascomycota; Saccharomycetes; Saccharomycetales; Eremothecium; Eremothecium_sinecaudum | dietary/environmental |
| denovo1305 | Eukaryota; Opisthokonta; Fungi; Ascomycota; Saccharomycetes; Saccharomycetales; Galactomyces; Geotrichum_candidum | dietary/environmental |
| denovo114 | Eukaryota; Opisthokonta; Fungi; Ascomycota; Saccharomycetes; Saccharomycetales; Hanseniaspora | dietary/environmental |

| | | |
|---|---|---|
| denovo213 | Eukaryota; Opisthokonta; Fungi; Ascomycota; Saccharomycetes; Saccharomycetales; Hanseniaspora; Hanseniaspora_uvarum_DSM_2768 | dietary/environmental |
| denovo80 | Eukaryota; Opisthokonta; Fungi; Ascomycota; Saccharomycetes; Saccharomycetales; Hyphopichia; Hyphopichia_burtonii | dietary/environmental |
| denovo2141 | Eukaryota; Opisthokonta; Fungi; Ascomycota; Saccharomycetes; Saccharomycetales; Hyphopichia; Hyphopichia_burtonii | dietary/environmental |
| denovo1533 | Eukaryota; Opisthokonta; Fungi; Ascomycota; Saccharomycetes; Saccharomycetales; Issatchenkia; Issatchenkia_hanoiensis | dietary/environmental |
| denovo1135 | Eukaryota; Opisthokonta; Fungi; Ascomycota; Saccharomycetes; Saccharomycetales; Issatchenkia; Pichia_terricola | dietary/environmental |
| denovo393 | Eukaryota; Opisthokonta; Fungi; Ascomycota; Saccharomycetes; Saccharomycetales; Kazachstania; Kazachstania_telluris | dietary/environmental |
| denovo2780 | Eukaryota; Opisthokonta; Fungi; Ascomycota; Saccharomycetes; Saccharomycetales; Lachancea; Lachancea_thermotolerans_CBS_6340 | dietary/environmental |
| denovo2877 | Eukaryota; Opisthokonta; Fungi; Ascomycota; Saccharomycetes; Saccharomycetales; Metschnikowia | dietary/environmental |
| denovo1295 | Eukaryota; Opisthokonta; Fungi; Ascomycota; Saccharomycetes; Saccharomycetales; Metschnikowia; Metschnikowia_continentalis | dietary/environmental |
| denovo3131 | Eukaryota; Opisthokonta; Fungi; Ascomycota; Saccharomycetes; Saccharomycetales; Metschnikowia; Metschnikowia_sp._11-1129 | dietary/environmental |
| denovo2035 | Eukaryota; Opisthokonta; Fungi; Ascomycota; Saccharomycetes; Saccharomycetales; Meyerozyma; Meyerozyma_guilliermondii | dietary/environmental |
| denovo526 | Eukaryota; Opisthokonta; Fungi; Ascomycota; Saccharomycetes; Saccharomycetales; Pichia | dietary/environmental |
| denovo426 | Eukaryota; Opisthokonta; Fungi; Ascomycota; Saccharomycetes; Saccharomycetales; Pichia | dietary/environmental |
| denovo1070 | Eukaryota; Opisthokonta; Fungi; Ascomycota; Saccharomycetes; Saccharomycetales; Pichia | dietary/environmental |
| denovo1895 | Eukaryota; Opisthokonta; Fungi; Ascomycota; Saccharomycetes; Saccharomycetales; Pichia | dietary/environmental |
| denovo1269 | Eukaryota; Opisthokonta; Fungi; Ascomycota; Saccharomycetes; Saccharomycetales; Pichia; Pichia_kudriavzevii | dietary/environmental |
| denovo3160 | Eukaryota; Opisthokonta; Fungi; Ascomycota; Saccharomycetes; Saccharomycetales; Pichia; Pichia_norvegensis | dietary/environmental |
| denovo2444 | Eukaryota; Opisthokonta; Fungi; Ascomycota; Saccharomycetes; Saccharomycetales; Saccharomycopsis | dietary/environmental |
| denovo2610 | Eukaryota; Opisthokonta; Fungi; Ascomycota; Saccharomycetes; Saccharomycetales; uncultured; uncultured_Debaryomyces | dietary/environmental |
| denovo573 | Eukaryota; Opisthokonta; Fungi; Ascomycota; Saccharomycetes; Saccharomycetales; Wickerhamomyces | dietary/environmental |
| denovo313 | Eukaryota; Opisthokonta; Fungi; Ascomycota; Saccharomycetes; Saccharomycetales; Wickerhamomyces | dietary/environmental |
| denovo1234 | Eukaryota; Opisthokonta; Fungi; Ascomycota; Saccharomycetes; Saccharomycetales; Wickerhamomyces | dietary/environmental |
| denovo332 | Eukaryota; Opisthokonta; Fungi; Ascomycota; Saccharomycetes; Saccharomycetales; Yamadazyma; Yamadazyma_triangularis | dietary/environmental |
| denovo1358 | Eukaryota; Opisthokonta; Fungi; Ascomycota; Saccharomycetes; Saccharomycetales; Yarrowia | dietary/environmental |
| denovo968 | Eukaryota; Opisthokonta; Fungi; Ascomycota; Sordariomycetes | dietary/environmental |
| denovo862 | Eukaryota; Opisthokonta; Fungi; Ascomycota; Sordariomycetes | dietary/environmental |
| denovo836 | Eukaryota; Opisthokonta; Fungi; Ascomycota; Sordariomycetes | dietary/environmental |
| denovo761 | Eukaryota; Opisthokonta; Fungi; Ascomycota; Sordariomycetes | dietary/environmental |
| denovo585 | Eukaryota; Opisthokonta; Fungi; Ascomycota; Sordariomycetes | dietary/environmental |
| denovo552 | Eukaryota; Opisthokonta; Fungi; Ascomycota; Sordariomycetes | dietary/environmental |
| denovo2601 | Eukaryota; Opisthokonta; Fungi; Ascomycota; Sordariomycetes | dietary/environmental |
| denovo2504 | Eukaryota; Opisthokonta; Fungi; Ascomycota; Sordariomycetes | dietary/environmental |
| denovo2400 | Eukaryota; Opisthokonta; Fungi; Ascomycota; Sordariomycetes | dietary/environmental |
| denovo237 | Eukaryota; Opisthokonta; Fungi; Ascomycota; Sordariomycetes | dietary/environmental |
| denovo182 | Eukaryota; Opisthokonta; Fungi; Ascomycota; Sordariomycetes | dietary/environmental |
| denovo1423 | Eukaryota; Opisthokonta; Fungi; Ascomycota; Sordariomycetes | dietary/environmental |
| denovo1928 | Eukaryota; Opisthokonta; Fungi; Ascomycota; Sordariomycetes | dietary/environmental |
| denovo1985 | Eukaryota; Opisthokonta; Fungi; Ascomycota; Sordariomycetes | dietary/environmental |
| denovo1808 | Eukaryota; Opisthokonta; Fungi; Ascomycota; Sordariomycetes; Boliniales; uncultured | dietary/environmental |
| denovo2738 | Eukaryota; Opisthokonta; Fungi; Ascomycota; Sordariomycetes; Diaporthales | dietary/environmental |

| | | |
|---|---|---|
| denovo1720 | Eukaryota; Opisthokonta; Fungi; Ascomycota; Sordariomycetes; Diaporthales; Phaeoacremonium | dietary/environmental |
| denovo3151 | Eukaryota; Opisthokonta; Fungi; Ascomycota; Sordariomycetes; Diaporthales; uncultured; uncultured_Diaporthales | dietary/environmental |
| denovo262 | Eukaryota; Opisthokonta; Fungi; Ascomycota; Sordariomycetes; Diaporthales; uncultured; uncultured_Diaporthales | dietary/environmental |
| denovo910 | Eukaryota; Opisthokonta; Fungi; Ascomycota; Sordariomycetes; Hypocreales | plant_pathogen |
| denovo849 | Eukaryota; Opisthokonta; Fungi; Ascomycota; Sordariomycetes; Hypocreales | plant_pathogen |
| denovo79 | Eukaryota; Opisthokonta; Fungi; Ascomycota; Sordariomycetes; Hypocreales | plant_pathogen |
| denovo3261 | Eukaryota; Opisthokonta; Fungi; Ascomycota; Sordariomycetes; Hypocreales | plant_pathogen |
| denovo3249 | Eukaryota; Opisthokonta; Fungi; Ascomycota; Sordariomycetes; Hypocreales | plant_pathogen |
| denovo3243 | Eukaryota; Opisthokonta; Fungi; Ascomycota; Sordariomycetes; Hypocreales | plant_pathogen |
| denovo3166 | Eukaryota; Opisthokonta; Fungi; Ascomycota; Sordariomycetes; Hypocreales | plant_pathogen |
| denovo296 | Eukaryota; Opisthokonta; Fungi; Ascomycota; Sordariomycetes; Hypocreales | plant_pathogen |
| denovo2335 | Eukaryota; Opisthokonta; Fungi; Ascomycota; Sordariomycetes; Hypocreales | plant_pathogen |
| denovo2334 | Eukaryota; Opisthokonta; Fungi; Ascomycota; Sordariomycetes; Hypocreales | plant_pathogen |
| denovo174 | Eukaryota; Opisthokonta; Fungi; Ascomycota; Sordariomycetes; Hypocreales | plant_pathogen |
| denovo1187 | Eukaryota; Opisthokonta; Fungi; Ascomycota; Sordariomycetes; Hypocreales | plant_pathogen |
| denovo1211 | Eukaryota; Opisthokonta; Fungi; Ascomycota; Sordariomycetes; Hypocreales | plant_pathogen |
| denovo1343 | Eukaryota; Opisthokonta; Fungi; Ascomycota; Sordariomycetes; Hypocreales | plant_pathogen |
| denovo1333 | Eukaryota; Opisthokonta; Fungi; Ascomycota; Sordariomycetes; Hypocreales | plant_pathogen |
| denovo1623 | Eukaryota; Opisthokonta; Fungi; Ascomycota; Sordariomycetes; Hypocreales | plant_pathogen |
| denovo1745 | Eukaryota; Opisthokonta; Fungi; Ascomycota; Sordariomycetes; Hypocreales | plant_pathogen |
| denovo1984 | Eukaryota; Opisthokonta; Fungi; Ascomycota; Sordariomycetes; Hypocreales | plant_pathogen |
| denovo2972 | Eukaryota; Opisthokonta; Fungi; Ascomycota; Sordariomycetes; Hypocreales; Claviceps; Claviceps_panicoidearum | plant_pathogen |
| denovo1599 | Eukaryota; Opisthokonta; Fungi; Ascomycota; Sordariomycetes; Hypocreales; Epichloe | plant_pathogen |
| denovo925 | Eukaryota; Opisthokonta; Fungi; Ascomycota; Sordariomycetes; Hypocreales; Fusarium | plant_pathogen |
| denovo272 | Eukaryota; Opisthokonta; Fungi; Ascomycota; Sordariomycetes; Hypocreales; Fusarium | plant_pathogen |
| denovo3178 | Eukaryota; Opisthokonta; Fungi; Ascomycota; Sordariomycetes; Hypocreales; Fusarium; Fusarium_graminearum | plant_pathogen |
| denovo2676 | Eukaryota; Opisthokonta; Fungi; Ascomycota; Sordariomycetes; Hypocreales; Fusarium; Fusarium_graminearum | plant_pathogen |
| denovo2617 | Eukaryota; Opisthokonta; Fungi; Ascomycota; Sordariomycetes; Hypocreales; Fusarium; Fusarium_graminearum | plant_pathogen |
| denovo58 | Eukaryota; Opisthokonta; Fungi; Ascomycota; Sordariomycetes; Hypocreales; Fusarium; Fusarium_oxysporum_f._sp._lycopersici_4287 | plant_pathogen |
| denovo354 | Eukaryota; Opisthokonta; Fungi; Ascomycota; Sordariomycetes; Hypocreales; Geosmithia; Geosmithia_putterillii | plant_pathogen |
| denovo129 | Eukaryota; Opisthokonta; Fungi; Ascomycota; Sordariomycetes; Hypocreales; Geosmithia; Geosmithia_putterillii | plant_pathogen |
| denovo2188 | Eukaryota; Opisthokonta; Fungi; Ascomycota; Sordariomycetes; Hypocreales; Hypocrea; Trichoderma_lixii | plant_pathogen |
| denovo1108 | Eukaryota; Opisthokonta; Fungi; Ascomycota; Sordariomycetes; Hypocreales; Hypomyces; Hypomyces_chrysospermus | plant_pathogen |
| denovo1350 | Eukaryota; Opisthokonta; Fungi; Ascomycota; Sordariomycetes; Hypocreales; Isaria | plant_pathogen |
| denovo2395 | Eukaryota; Opisthokonta; Fungi; Ascomycota; Sordariomycetes; Hypocreales; Mariannaea; Mariannaea_elegans_var._punicea | plant_pathogen |
| denovo576 | Eukaryota; Opisthokonta; Fungi; Ascomycota; Sordariomycetes; Hypocreales; Metarhizium; Metarhizium_anisopliae | plant_pathogen |
| denovo320 | Eukaryota; Opisthokonta; Fungi; Ascomycota; Sordariomycetes; Hypocreales; Nectria; Nectria_cinnabarina | plant_pathogen |
| denovo1133 | Eukaryota; Opisthokonta; Fungi; Ascomycota; Sordariomycetes; Hypocreales; Nomuraea; Metarhizium_rileyi | plant_pathogen |
| denovo1564 | Eukaryota; Opisthokonta; Fungi; Ascomycota; Sordariomycetes; Hypocreales; Polycephalomyces; Polycephalomyces_nipponicus | plant_pathogen |
| denovo194 | Eukaryota; Opisthokonta; Fungi; Ascomycota; Sordariomycetes; Hypocreales; Trichoderma | plant_pathogen |

| | | |
|---|---|---|
| denovo1274 | Eukaryota; Opisthokonta; Fungi; Ascomycota; Sordariomycetes; Hypocreales; uncultured | plant_pathogen |
| denovo2654 | Eukaryota; Opisthokonta; Fungi; Ascomycota; Sordariomycetes; Hypocreales; uncultured; uncultured_fungus | plant_pathogen |
| denovo2325 | Eukaryota; Opisthokonta; Fungi; Ascomycota; Sordariomycetes; Hypocreales; uncultured; uncultured_fungus | plant_pathogen |
| denovo1177 | Eukaryota; Opisthokonta; Fungi; Ascomycota; Sordariomycetes; Hypocreales; uncultured; uncultured_fungus | plant_pathogen |
| denovo751 | Eukaryota; Opisthokonta; Fungi; Ascomycota; Sordariomycetes; Incertae_Sedis | dietary/environmental |
| denovo644 | Eukaryota; Opisthokonta; Fungi; Ascomycota; Sordariomycetes; Incertae_Sedis | dietary/environmental |
| denovo597 | Eukaryota; Opisthokonta; Fungi; Ascomycota; Sordariomycetes; Incertae_Sedis | dietary/environmental |
| denovo406 | Eukaryota; Opisthokonta; Fungi; Ascomycota; Sordariomycetes; Incertae_Sedis | dietary/environmental |
| denovo3322 | Eukaryota; Opisthokonta; Fungi; Ascomycota; Sordariomycetes; Incertae_Sedis | dietary/environmental |
| denovo3210 | Eukaryota; Opisthokonta; Fungi; Ascomycota; Sordariomycetes; Incertae_Sedis | dietary/environmental |
| denovo3184 | Eukaryota; Opisthokonta; Fungi; Ascomycota; Sordariomycetes; Incertae_Sedis | dietary/environmental |
| denovo306 | Eukaryota; Opisthokonta; Fungi; Ascomycota; Sordariomycetes; Incertae_Sedis | dietary/environmental |
| denovo2746 | Eukaryota; Opisthokonta; Fungi; Ascomycota; Sordariomycetes; Incertae_Sedis | dietary/environmental |
| denovo2704 | Eukaryota; Opisthokonta; Fungi; Ascomycota; Sordariomycetes; Incertae_Sedis | dietary/environmental |
| denovo2638 | Eukaryota; Opisthokonta; Fungi; Ascomycota; Sordariomycetes; Incertae_Sedis | dietary/environmental |
| denovo2569 | Eukaryota; Opisthokonta; Fungi; Ascomycota; Sordariomycetes; Incertae_Sedis | dietary/environmental |
| denovo254 | Eukaryota; Opisthokonta; Fungi; Ascomycota; Sordariomycetes; Incertae_Sedis | dietary/environmental |
| denovo2380 | Eukaryota; Opisthokonta; Fungi; Ascomycota; Sordariomycetes; Incertae_Sedis | dietary/environmental |
| denovo136 | Eukaryota; Opisthokonta; Fungi; Ascomycota; Sordariomycetes; Incertae_Sedis | dietary/environmental |
| denovo1112 | Eukaryota; Opisthokonta; Fungi; Ascomycota; Sordariomycetes; Incertae_Sedis | dietary/environmental |
| denovo1369 | Eukaryota; Opisthokonta; Fungi; Ascomycota; Sordariomycetes; Incertae_Sedis | dietary/environmental |
| denovo1576 | Eukaryota; Opisthokonta; Fungi; Ascomycota; Sordariomycetes; Incertae_Sedis | dietary/environmental |
| denovo1640 | Eukaryota; Opisthokonta; Fungi; Ascomycota; Sordariomycetes; Incertae_Sedis | dietary/environmental |
| denovo2265 | Eukaryota; Opisthokonta; Fungi; Ascomycota; Sordariomycetes; Incertae_Sedis | dietary/environmental |
| denovo2858 | Eukaryota; Opisthokonta; Fungi; Ascomycota; Sordariomycetes; Incertae_Sedis; Acremonium | dietary/environmental |
| denovo1934 | Eukaryota; Opisthokonta; Fungi; Ascomycota; Sordariomycetes; Incertae_Sedis; Acremonium | dietary/environmental |
| denovo1914 | Eukaryota; Opisthokonta; Fungi; Ascomycota; Sordariomycetes; Incertae_Sedis; Apiospora; Arthrinium_arundinis | dietary/environmental |
| denovo3321 | Eukaryota; Opisthokonta; Fungi; Ascomycota; Sordariomycetes; Incertae_Sedis; Arthrinium | dietary/environmental |
| denovo329 | Eukaryota; Opisthokonta; Fungi; Ascomycota; Sordariomycetes; Incertae_Sedis; Arthrinium | dietary/environmental |
| denovo3199 | Eukaryota; Opisthokonta; Fungi; Ascomycota; Sordariomycetes; Incertae_Sedis; Arthrinium | dietary/environmental |
| denovo2549 | Eukaryota; Opisthokonta; Fungi; Ascomycota; Sordariomycetes; Incertae_Sedis; Arthrinium | dietary/environmental |
| denovo1468 | Eukaryota; Opisthokonta; Fungi; Ascomycota; Sordariomycetes; Incertae_Sedis; Arthrinium | dietary/environmental |
| denovo1737 | Eukaryota; Opisthokonta; Fungi; Ascomycota; Sordariomycetes; Incertae_Sedis; Arthrinium | dietary/environmental |
| denovo2169 | Eukaryota; Opisthokonta; Fungi; Ascomycota; Sordariomycetes; Incertae_Sedis; Arthrinium | dietary/environmental |
| denovo472 | Eukaryota; Opisthokonta; Fungi; Ascomycota; Sordariomycetes; Incertae_Sedis; Arthrinium; Arthrinium_japonicum | dietary/environmental |
| denovo289 | Eukaryota; Opisthokonta; Fungi; Ascomycota; Sordariomycetes; Incertae_Sedis; Arthrinium; Arthrinium_phaeospermum | dietary/environmental |
| denovo2394 | Eukaryota; Opisthokonta; Fungi; Ascomycota; Sordariomycetes; Incertae_Sedis; Arthrinium; Arthrinium_phaeospermum | dietary/environmental |
| denovo278 | Eukaryota; Opisthokonta; Fungi; Ascomycota; Sordariomycetes; Incertae_Sedis; Arthrinium; Arthrinium_sacchari | dietary/environmental |
| denovo1725 | Eukaryota; Opisthokonta; Fungi; Ascomycota; Sordariomycetes; Incertae_Sedis; Canalisporium | dietary/environmental |
| denovo1057 | Eukaryota; Opisthokonta; Fungi; Ascomycota; Sordariomycetes; Incertae_Sedis; Ceratosphaeria; Ceratosphaeria_lampadophora | dietary/environmental |

| | | |
|---|---|---|
| denovo954 | Eukaryota; Opisthokonta; Fungi; Ascomycota; Sordariomycetes; Incertae_Sedis; Colletotrichum | dietary/environmental |
| denovo542 | Eukaryota; Opisthokonta; Fungi; Ascomycota; Sordariomycetes; Incertae_Sedis; Colletotrichum | dietary/environmental |
| denovo2253 | Eukaryota; Opisthokonta; Fungi; Ascomycota; Sordariomycetes; Incertae_Sedis; Conioscypha | dietary/environmental |
| denovo370 | Eukaryota; Opisthokonta; Fungi; Ascomycota; Sordariomycetes; Incertae_Sedis; Flammispora; Flammispora_bioteca | dietary/environmental |
| denovo3172 | Eukaryota; Opisthokonta; Fungi; Ascomycota; Sordariomycetes; Incertae_Sedis; Geosmithia; Geosmithia_putterillii | dietary/environmental |
| denovo1812 | Eukaryota; Opisthokonta; Fungi; Ascomycota; Sordariomycetes; Incertae_Sedis; Geosmithia; Geosmithia_putterillii | dietary/environmental |
| denovo1340 | Eukaryota; Opisthokonta; Fungi; Ascomycota; Sordariomycetes; Incertae_Sedis; Kionochaeta | dietary/environmental |
| denovo956 | Eukaryota; Opisthokonta; Fungi; Ascomycota; Sordariomycetes; Incertae_Sedis; Liberomyces | dietary/environmental |
| denovo3099 | Eukaryota; Opisthokonta; Fungi; Ascomycota; Sordariomycetes; Incertae_Sedis; Liberomyces | dietary/environmental |
| denovo1278 | Eukaryota; Opisthokonta; Fungi; Ascomycota; Sordariomycetes; Incertae_Sedis; Liberomyces | dietary/environmental |
| denovo3299 | Eukaryota; Opisthokonta; Fungi; Ascomycota; Sordariomycetes; Incertae_Sedis; Papulosa; Papulosa_amerospora | dietary/environmental |
| denovo1388 | Eukaryota; Opisthokonta; Fungi; Ascomycota; Sordariomycetes; Incertae_Sedis; Papulosa; Papulosa_amerospora | dietary/environmental |
| denovo1664 | Eukaryota; Opisthokonta; Fungi; Ascomycota; Sordariomycetes; Incertae_Sedis; Papulosa; Papulosa_amerospora | dietary/environmental |
| denovo2034 | Eukaryota; Opisthokonta; Fungi; Ascomycota; Sordariomycetes; Incertae_Sedis; Papulosa; Papulosa_amerospora | dietary/environmental |
| denovo1516 | Eukaryota; Opisthokonta; Fungi; Ascomycota; Sordariomycetes; Incertae_Sedis; Stachybotrys | dietary/environmental |
| denovo3060 | Eukaryota; Opisthokonta; Fungi; Ascomycota; Sordariomycetes; Incertae_Sedis; Surculiseries; Surculiseries_rugispora | dietary/environmental |
| denovo260 | Eukaryota; Opisthokonta; Fungi; Ascomycota; Sordariomycetes; Incertae_Sedis; Surculiseries; Surculiseries_rugispora | dietary/environmental |
| denovo1188 | Eukaryota; Opisthokonta; Fungi; Ascomycota; Sordariomycetes; Incertae_Sedis; uncultured | dietary/environmental |
| denovo2382 | Eukaryota; Opisthokonta; Fungi; Ascomycota; Sordariomycetes; Incertae_Sedis; Ustilaginoidea; Ustilaginoidea_virens | dietary/environmental |
| denovo815 | Eukaryota; Opisthokonta; Fungi; Ascomycota; Sordariomycetes; Magnaporthales; Gaeumannomyces | dietary/environmental |
| denovo3212 | Eukaryota; Opisthokonta; Fungi; Ascomycota; Sordariomycetes; Magnaporthales; Magnaporthe | dietary/environmental |
| denovo2705 | Eukaryota; Opisthokonta; Fungi; Ascomycota; Sordariomycetes; Magnaporthales; Magnaporthe; Magnaporthe_oryzae | dietary/environmental |
| denovo454 | Eukaryota; Opisthokonta; Fungi; Ascomycota; Sordariomycetes; Microascales | dietary/environmental |
| denovo2508 | Eukaryota; Opisthokonta; Fungi; Ascomycota; Sordariomycetes; Microascales; Graphium | dietary/environmental |
| denovo1342 | Eukaryota; Opisthokonta; Fungi; Ascomycota; Sordariomycetes; Microascales; Graphium | dietary/environmental |
| denovo1678 | Eukaryota; Opisthokonta; Fungi; Ascomycota; Sordariomycetes; Microascales; Graphium; Graphium_euwallaceae | dietary/environmental |
| denovo1744 | Eukaryota; Opisthokonta; Fungi; Ascomycota; Sordariomycetes; Microascales; Graphium; Graphium_euwallaceae | dietary/environmental |
| denovo104 | Eukaryota; Opisthokonta; Fungi; Ascomycota; Sordariomycetes; Microascales; Microascus | dietary/environmental |
| denovo1719 | Eukaryota; Opisthokonta; Fungi; Ascomycota; Sordariomycetes; Ophiostomatales | dietary/environmental |
| denovo67 | Eukaryota; Opisthokonta; Fungi; Ascomycota; Sordariomycetes; Ophiostomatales; Ophiostoma | dietary/environmental |
| denovo869 | Eukaryota; Opisthokonta; Fungi; Ascomycota; Sordariomycetes; Sordariales | dietary/environmental |
| denovo628 | Eukaryota; Opisthokonta; Fungi; Ascomycota; Sordariomycetes; Sordariales | dietary/environmental |
| denovo321 | Eukaryota; Opisthokonta; Fungi; Ascomycota; Sordariomycetes; Sordariales | dietary/environmental |
| denovo2436 | Eukaryota; Opisthokonta; Fungi; Ascomycota; Sordariomycetes; Sordariales | dietary/environmental |
| denovo1290 | Eukaryota; Opisthokonta; Fungi; Ascomycota; Sordariomycetes; Sordariales | dietary/environmental |
| denovo1627 | Eukaryota; Opisthokonta; Fungi; Ascomycota; Sordariomycetes; Sordariales | dietary/environmental |
| denovo1693 | Eukaryota; Opisthokonta; Fungi; Ascomycota; Sordariomycetes; Sordariales | dietary/environmental |
| denovo1865 | Eukaryota; Opisthokonta; Fungi; Ascomycota; Sordariomycetes; Sordariales | dietary/environmental |
| denovo1998 | Eukaryota; Opisthokonta; Fungi; Ascomycota; Sordariomycetes; Sordariales | dietary/environmental |
| denovo1987 | Eukaryota; Opisthokonta; Fungi; Ascomycota; Sordariomycetes; Sordariales | dietary/environmental |

| | | |
|---|---|---|
| denovo2091 | Eukaryota; Opisthokonta; Fungi; Ascomycota; Sordariomycetes; Sordariales | dietary/environmental |
| denovo484 | Eukaryota; Opisthokonta; Fungi; Ascomycota; Sordariomycetes; Sordariales; Chaetomium | dietary/environmental |
| denovo3285 | Eukaryota; Opisthokonta; Fungi; Ascomycota; Sordariomycetes; Sordariales; Chaetomium; Chaetomium_elatum | dietary/environmental |
| denovo530 | Eukaryota; Opisthokonta; Fungi; Ascomycota; Sordariomycetes; Sordariales; Coniochaeta | dietary/environmental |
| denovo2010 | Eukaryota; Opisthokonta; Fungi; Ascomycota; Sordariomycetes; Sordariales; Lecythophora | dietary/environmental |
| denovo474 | Eukaryota; Opisthokonta; Fungi; Ascomycota; Sordariomycetes; Sordariales; Phialemonium; Phialemonium_inflatum | dietary/environmental |
| denovo2706 | Eukaryota; Opisthokonta; Fungi; Ascomycota; Sordariomycetes; Sordariales; Sordaria | dietary/environmental |
| denovo615 | Eukaryota; Opisthokonta; Fungi; Ascomycota; Sordariomycetes; Sordariales; Sordaria; Sordaria_fimicola | dietary/environmental |
| denovo992 | Eukaryota; Opisthokonta; Fungi; Ascomycota; Sordariomycetes; Sordariales; uncultured | dietary/environmental |
| denovo1163 | Eukaryota; Opisthokonta; Fungi; Ascomycota; Sordariomycetes; Sordariales; uncultured | dietary/environmental |
| denovo1805 | Eukaryota; Opisthokonta; Fungi; Ascomycota; Sordariomycetes; Sordariales; uncultured | dietary/environmental |
| denovo3018 | Eukaryota; Opisthokonta; Fungi; Ascomycota; Sordariomycetes; Sordariales; uncultured; fungal_sp. | dietary/environmental |
| denovo2966 | Eukaryota; Opisthokonta; Fungi; Ascomycota; Sordariomycetes; Sordariales; uncultured; fungal_sp. | dietary/environmental |
| denovo503 | Eukaryota; Opisthokonta; Fungi; Ascomycota; Sordariomycetes; Sordariales; uncultured; uncultured_fungus | dietary/environmental |
| denovo2377 | Eukaryota; Opisthokonta; Fungi; Ascomycota; Sordariomycetes; Sordariales; uncultured; uncultured_fungus | dietary/environmental |
| denovo283 | Eukaryota; Opisthokonta; Fungi; Ascomycota; Sordariomycetes; uncultured; uncultured | dietary/environmental |
| denovo1496 | Eukaryota; Opisthokonta; Fungi; Ascomycota; Sordariomycetes; uncultured; uncultured | dietary/environmental |
| denovo1743 | Eukaryota; Opisthokonta; Fungi; Ascomycota; Sordariomycetes; uncultured; uncultured | dietary/environmental |
| denovo1801 | Eukaryota; Opisthokonta; Fungi; Ascomycota; Sordariomycetes; uncultured; uncultured | dietary/environmental |
| denovo2127 | Eukaryota; Opisthokonta; Fungi; Ascomycota; Sordariomycetes; uncultured; uncultured | dietary/environmental |
| denovo986 | Eukaryota; Opisthokonta; Fungi; Ascomycota; Sordariomycetes; Xylariales | dietary/environmental |
| denovo898 | Eukaryota; Opisthokonta; Fungi; Ascomycota; Sordariomycetes; Xylariales | dietary/environmental |
| denovo755 | Eukaryota; Opisthokonta; Fungi; Ascomycota; Sordariomycetes; Xylariales | dietary/environmental |
| denovo486 | Eukaryota; Opisthokonta; Fungi; Ascomycota; Sordariomycetes; Xylariales | dietary/environmental |
| denovo3263 | Eukaryota; Opisthokonta; Fungi; Ascomycota; Sordariomycetes; Xylariales | dietary/environmental |
| denovo3139 | Eukaryota; Opisthokonta; Fungi; Ascomycota; Sordariomycetes; Xylariales | dietary/environmental |
| denovo287 | Eukaryota; Opisthokonta; Fungi; Ascomycota; Sordariomycetes; Xylariales | dietary/environmental |
| denovo1755 | Eukaryota; Opisthokonta; Fungi; Ascomycota; Sordariomycetes; Xylariales | dietary/environmental |
| denovo2554 | Eukaryota; Opisthokonta; Fungi; Ascomycota; Sordariomycetes; Xylariales; Eutypa | dietary/environmental |
| denovo1969 | Eukaryota; Opisthokonta; Fungi; Ascomycota; Sordariomycetes; Xylariales; Eutypa | dietary/environmental |
| denovo753 | Eukaryota; Opisthokonta; Fungi; Ascomycota; Sordariomycetes; Xylariales; Liberomyces; Liberomyces_saliciphilus | dietary/environmental |
| denovo35 | Eukaryota; Opisthokonta; Fungi; Ascomycota; Sordariomycetes; Xylariales; Liberomyces; Liberomyces_saliciphilus | dietary/environmental |
| denovo2854 | Eukaryota; Opisthokonta; Fungi; Ascomycota; Sordariomycetes; Xylariales; Pestalotia | dietary/environmental |
| denovo2479 | Eukaryota; Opisthokonta; Fungi; Ascomycota; Sordariomycetes; Xylariales; Pestalotiopsis | dietary/environmental |
| denovo816 | Eukaryota; Opisthokonta; Fungi; Ascomycota; Sordariomycetes; Xylariales; Pestalotiopsis; Pestalotiopsis_maculans | dietary/environmental |
| denovo51 | Eukaryota; Opisthokonta; Fungi; Ascomycota; Sordariomycetes; Xylariales; Pestalotiopsis; Pestalotiopsis_maculans | dietary/environmental |
| denovo180 | Eukaryota; Opisthokonta; Fungi; Ascomycota; Sordariomycetes; Xylariales; Pestalotiopsis; Pestalotiopsis_maculans | dietary/environmental |
| denovo1167 | Eukaryota; Opisthokonta; Fungi; Ascomycota; Sordariomycetes; Xylariales; Seiridium | dietary/environmental |
| denovo2909 | Eukaryota; Opisthokonta; Fungi; Ascomycota; Sordariomycetes; Xylariales; uncultured | dietary/environmental |
| denovo850 | Eukaryota; Opisthokonta; Fungi; Ascomycota; Sordariomycetes; Xylariales; Xylaria | dietary/environmental |

| | | |
|---|---|---|
| denovo2783 | Eukaryota; Opisthokonta; Fungi; Ascomycota; Sordariomycetes; Xylariales; Xylaria | dietary/environmental |
| denovo1215 | Eukaryota; Opisthokonta; Fungi; Ascomycota; Sordariomycetes; Xylariales; Xylaria | dietary/environmental |
| denovo571 | Eukaryota; Opisthokonta; Fungi; Ascomycota; Sordariomycetes; Xylariales; Xylariales | dietary/environmental |
| denovo3133 | Eukaryota; Opisthokonta; Fungi; Ascomycota; uncultured; Dothideomycetes; Dothideomycetes; Dothideomycetes_sp._LS-2013g | dietary/environmental |
| denovo2180 | Eukaryota; Opisthokonta; Fungi; Ascomycota; uncultured; Dothideomycetes; Dothideomycetes; Dothideomycetes_sp._LS-2013g | dietary/environmental |
| denovo273 | Eukaryota; Opisthokonta; Fungi; Ascomycota; uncultured; Pezizales; uncultured; uncultured_eukaryote | dietary/environmental |
| denovo1467 | Eukaryota; Opisthokonta; Fungi; Ascomycota; uncultured; Sarcinomyces; Sarcinomyces | dietary/environmental |
| denovo1557 | Eukaryota; Opisthokonta; Fungi; Ascomycota; uncultured; Sarcinomyces; Sarcinomyces | dietary/environmental |
| denovo2281 | Eukaryota; Opisthokonta; Fungi; Ascomycota; uncultured; Sarcinomyces; Sarcinomyces | dietary/environmental |
| denovo2274 | Eukaryota; Opisthokonta; Fungi; Ascomycota; uncultured; Sarcinomyces; Sarcinomyces | dietary/environmental |
| denovo1920 | Eukaryota; Opisthokonta; Fungi; Basidiomycota; Agaricomycetes | dietary/environmental |
| denovo3302 | Eukaryota; Opisthokonta; Fungi; Basidiomycota; Agaricomycetes; Ganoderma; Ganoderma | dietary/environmental |
| denovo2843 | Eukaryota; Opisthokonta; Fungi; Basidiomycota; Agaricomycetes; Trechisporales; Trechispora | dietary/environmental |
| denovo2315 | Eukaryota; Opisthokonta; Fungi; Basidiomycota; Ustilaginomycetes; Ustilaginales; Ustilago | dietary/environmental |
| denovo2539 | Eukaryota; Opisthokonta; Fungi; Chytridiomycota; Chytridiomycetes | dietary/environmental |
| denovo1005 | Eukaryota; Opisthokonta; Fungi; Chytridiomycota; Chytridiomycetes | dietary/environmental |
| denovo2258 | Eukaryota; Opisthokonta; Fungi; Chytridiomycota; Chytridiomycetes | dietary/environmental |
| denovo2604 | Eukaryota; Opisthokonta; Fungi; Chytridiomycota; Chytridiomycetes; Chytridiales; Chytridiales; Chytridiales_sp._JEL187 | dietary/environmental |
| denovo3078 | Eukaryota; Opisthokonta; Fungi; Chytridiomycota; Chytridiomycetes; Chytridiales; uncultured | dietary/environmental |
| denovo2731 | Eukaryota; Opisthokonta; Fungi; Chytridiomycota; Chytridiomycetes; Chytridiales; uncultured | dietary/environmental |
| denovo2170 | Eukaryota; Opisthokonta; Fungi; Chytridiomycota; Chytridiomycetes; Chytridiales; uncultured | dietary/environmental |
| denovo3320 | Eukaryota; Opisthokonta; Fungi; Chytridiomycota; Chytridiomycetes; Chytridiales; uncultured; uncultured_rhizosphere_chytridiomycete | dietary/environmental |
| denovo1955 | Eukaryota; Opisthokonta; Fungi; Chytridiomycota; Chytridiomycetes; Chytridiales; uncultured; uncultured_rhizosphere_chytridiomycete | dietary/environmental |
| denovo618 | Eukaryota; Opisthokonta; Fungi; Chytridiomycota; Chytridiomycetes; Cladochytriales; Nowakowskiella | dietary/environmental |
| denovo1228 | Eukaryota; Opisthokonta; Fungi; Chytridiomycota; Chytridiomycetes; Cladochytriales; Nowakowskiella | dietary/environmental |
| denovo1127 | Eukaryota; Opisthokonta; Fungi; Chytridiomycota; Chytridiomycetes; Incertae_Sedis; uncultured | dietary/environmental |
| denovo1875 | Eukaryota; Opisthokonta; Fungi; Chytridiomycota; Chytridiomycetes; Incertae_Sedis; uncultured | dietary/environmental |
| denovo1540 | Eukaryota; Opisthokonta; Fungi; Chytridiomycota; Chytridiomycetes; Incertae_Sedis; uncultured; uncultured_soil_fungus | dietary/environmental |
| denovo1173 | Eukaryota; Opisthokonta; Fungi; Chytridiomycota; Chytridiomycetes; Rhizophlyctidales | dietary/environmental |
| denovo1563 | Eukaryota; Opisthokonta; Fungi; Chytridiomycota; Chytridiomycetes; Spizellomycetales | dietary/environmental |
| denovo1384 | Eukaryota; Opisthokonta; Fungi; Chytridiomycota; uncultured; Incertae_Sedis; uncultured | dietary/environmental |
| denovo1866 | Eukaryota; Opisthokonta; Fungi; Chytridiomycota; uncultured; Incertae_Sedis; uncultured | dietary/environmental |
| denovo1803 | Eukaryota; Opisthokonta; Fungi; Chytridiomycota; uncultured; Incertae_Sedis; uncultured; uncultured_Chytridiomycota | dietary/environmental |
| denovo2022 | Eukaryota; Opisthokonta; Fungi; Chytridiomycota; uncultured; Incertae_Sedis; uncultured; uncultured_Chytridiomycota | dietary/environmental |
| denovo1756 | Eukaryota; Opisthokonta; Fungi; Chytridiomycota; uncultured; Rhizophydiales | dietary/environmental |
| denovo2337 | Eukaryota; Opisthokonta; Fungi; Chytridiomycota; uncultured; Rhizophydiales; uncultured | dietary/environmental |
| denovo711 | Eukaryota; Opisthokonta; Fungi; Cryptomycota; uncultured; uncultured; uncultured | dietary/environmental |
| denovo604 | Eukaryota; Opisthokonta; Fungi; Cryptomycota; uncultured; uncultured; uncultured | dietary/environmental |
| denovo2767 | Eukaryota; Opisthokonta; Fungi; Cryptomycota; uncultured; uncultured; uncultured | dietary/environmental |
| denovo2439 | Eukaryota; Opisthokonta; Fungi; Cryptomycota; uncultured; uncultured; uncultured | dietary/environmental |

| | | |
|---|---|---|
| denovo1078 | Eukaryota; Opisthokonta; Fungi; Cryptomycota; uncultured; uncultured; uncultured | dietary/environmental |
| denovo1502 | Eukaryota; Opisthokonta; Fungi; Cryptomycota; uncultured; uncultured; uncultured | dietary/environmental |
| denovo1742 | Eukaryota; Opisthokonta; Fungi; Cryptomycota; uncultured; uncultured; uncultured | dietary/environmental |
| denovo2110 | Eukaryota; Opisthokonta; Fungi; Cryptomycota; uncultured; uncultured; uncultured | dietary/environmental |
| denovo802 | Eukaryota; Opisthokonta; Fungi; Cryptomycota; uncultured; uncultured; uncultured; uncultured_Eimeriidae | dietary/environmental |
| denovo1798 | Eukaryota; Opisthokonta; Fungi; Cryptomycota; uncultured; uncultured; uncultured; uncultured_Eimeriidae | dietary/environmental |
| denovo1517 | Eukaryota; Opisthokonta; Fungi; Dikarya | dietary/environmental |
| denovo2014 | Eukaryota; Opisthokonta; Fungi; Dikarya; Ascomycota; saccharomyceta; Pezizomycotina; leotiomyceta | dietary/environmental |
| denovo757 | Eukaryota; Opisthokonta; Fungi; Dikarya; Ascomycota; saccharomyceta; Saccharomycotina; Saccharomycetes; Saccharomycetales | dietary/environmental |
| denovo691 | Eukaryota; Opisthokonta; Fungi; Dikarya; Ascomycota; saccharomyceta; Saccharomycotina; Saccharomycetes; Saccharomycetales | dietary/environmental |
| denovo605 | Eukaryota; Opisthokonta; Fungi; Dikarya; Ascomycota; saccharomyceta; Saccharomycotina; Saccharomycetes; Saccharomycetales | dietary/environmental |
| denovo534 | Eukaryota; Opisthokonta; Fungi; Dikarya; Ascomycota; saccharomyceta; Saccharomycotina; Saccharomycetes; Saccharomycetales | dietary/environmental |
| denovo481 | Eukaryota; Opisthokonta; Fungi; Dikarya; Ascomycota; saccharomyceta; Saccharomycotina; Saccharomycetes; Saccharomycetales | dietary/environmental |
| denovo469 | Eukaryota; Opisthokonta; Fungi; Dikarya; Ascomycota; saccharomyceta; Saccharomycotina; Saccharomycetes; Saccharomycetales | dietary/environmental |
| denovo417 | Eukaryota; Opisthokonta; Fungi; Dikarya; Ascomycota; saccharomyceta; Saccharomycotina; Saccharomycetes; Saccharomycetales | dietary/environmental |
| denovo2882 | Eukaryota; Opisthokonta; Fungi; Dikarya; Ascomycota; saccharomyceta; Saccharomycotina; Saccharomycetes; Saccharomycetales | dietary/environmental |
| denovo2801 | Eukaryota; Opisthokonta; Fungi; Dikarya; Ascomycota; saccharomyceta; Saccharomycotina; Saccharomycetes; Saccharomycetales | dietary/environmental |
| denovo2770 | Eukaryota; Opisthokonta; Fungi; Dikarya; Ascomycota; saccharomyceta; Saccharomycotina; Saccharomycetes; Saccharomycetales | dietary/environmental |
| denovo1156 | Eukaryota; Opisthokonta; Fungi; Dikarya; Ascomycota; saccharomyceta; Saccharomycotina; Saccharomycetes; Saccharomycetales | dietary/environmental |
| denovo1253 | Eukaryota; Opisthokonta; Fungi; Dikarya; Ascomycota; saccharomyceta; Saccharomycotina; Saccharomycetes; Saccharomycetales | dietary/environmental |
| denovo1629 | Eukaryota; Opisthokonta; Fungi; Dikarya; Ascomycota; saccharomyceta; Saccharomycotina; Saccharomycetes; Saccharomycetales | dietary/environmental |
| denovo1870 | Eukaryota; Opisthokonta; Fungi; Dikarya; Ascomycota; saccharomyceta; Saccharomycotina; Saccharomycetes; Saccharomycetales | dietary/environmental |
| denovo1946 | Eukaryota; Opisthokonta; Fungi; Dikarya; Ascomycota; saccharomyceta; Saccharomycotina; Saccharomycetes; Saccharomycetales | dietary/environmental |
| denovo2251 | Eukaryota; Opisthokonta; Fungi; Entomophthoromycota; Entomophthoromycotina; Entomophthorales | dietary/environmental |
| denovo664 | Eukaryota; Opisthokonta; Fungi; Entomophthoromycota; Entomophthoromycotina; Entomophthorales; Conidiobolus | dietary/environmental |
| denovo508 | Eukaryota; Opisthokonta; Fungi; Entomophthoromycota; Entomophthoromycotina; Entomophthorales; Conidiobolus | dietary/environmental |
| denovo435 | Eukaryota; Opisthokonta; Fungi; Entomophthoromycota; Entomophthoromycotina; Entomophthorales; Conidiobolus | dietary/environmental |
| denovo2868 | Eukaryota; Opisthokonta; Fungi; Entomophthoromycota; Entomophthoromycotina; Entomophthorales; Conidiobolus | dietary/environmental |
| denovo1079 | Eukaryota; Opisthokonta; Fungi; Entomophthoromycota; Entomophthoromycotina; Entomophthorales; Conidiobolus | dietary/environmental |
| denovo592 | Eukaryota; Opisthokonta; Fungi; environmental_samples_<Fungi>; uncultured_fungus | dietary/environmental |
| denovo175 | Eukaryota; Opisthokonta; Fungi; Fungi_incertae_sedis; Microsporidia | dietary/environmental |
| denovo2000 | Eukaryota; Opisthokonta; Fungi; Glomeromycota; Glomeromycetes; Glomerales | dietary/environmental |
| denovo1954 | Eukaryota; Opisthokonta; Fungi; Glomeromycota; Glomeromycetes; Glomerales; uncultured; uncultured_mycorrhizal_fungus | dietary/environmental |
| denovo1908 | Eukaryota; Opisthokonta; Fungi; Glomeromycota; Glomeromycetes; Glomerales; uncultured; uncultured_mycorrhizal_fungus | dietary/environmental |
| denovo1781 | Eukaryota; Opisthokonta; Fungi; Kickxellomycotina; Kickxellomycotina; Kickxellales | dietary/environmental |
| denovo3002 | Eukaryota; Opisthokonta; Fungi; LKM15; uncultured; uncultured; uncultured; uncultured_eukaryote | dietary/environmental |
| denovo2973 | Eukaryota; Opisthokonta; Fungi; LKM15; uncultured; uncultured; uncultured; uncultured_fungus | dietary/environmental |
| denovo2573 | Eukaryota; Opisthokonta; Fungi; Mucoromycotina; Mucoromycotina; Incertae_Sedis; Basidiobolus | dietary/environmental |
| denovo911 | Eukaryota; Opisthokonta; Fungi; Mucoromycotina; Mucoromycotina; Mortierellales | dietary/environmental |
| denovo871 | Eukaryota; Opisthokonta; Fungi; Mucoromycotina; Mucoromycotina; Mortierellales; Mortierella | dietary/environmental |

| | | |
|---|---|---|
| denovo832 | Eukaryota; Opisthokonta; Fungi; Mucoromycotina; Mucoromycotina; Mortierellales; uncultured | dietary/environmental |
| denovo428 | Eukaryota; Opisthokonta; Fungi; Mucoromycotina; Mucoromycotina; Mortierellales; uncultured | dietary/environmental |
| denovo614 | Eukaryota; Opisthokonta; Fungi; Mucoromycotina; Mucoromycotina; Mortierellales; uncultured; uncultured_Eimeriidae | dietary/environmental |
| denovo598 | Eukaryota; Opisthokonta; Fungi; Mucoromycotina; Mucoromycotina; Mortierellales; uncultured; uncultured_Eimeriidae | dietary/environmental |
| denovo546 | Eukaryota; Opisthokonta; Fungi; Mucoromycotina; Mucoromycotina; Mortierellales; uncultured; uncultured_Eimeriidae | dietary/environmental |
| denovo2527 | Eukaryota; Opisthokonta; Fungi; Mucoromycotina; Mucoromycotina; Mortierellales; uncultured; uncultured_Eimeriidae | dietary/environmental |
| denovo1727 | Eukaryota; Opisthokonta; Fungi; Mucoromycotina; Mucoromycotina; Mortierellales; uncultured; uncultured_Eimeriidae | dietary/environmental |
| denovo916 | Eukaryota; Opisthokonta; Fungi; Mucoromycotina; Mucoromycotina; Mucorales | dietary/environmental |
| denovo2979 | Eukaryota; Opisthokonta; Fungi; Mucoromycotina; Mucoromycotina; Mucorales | dietary/environmental |
| denovo1116 | Eukaryota; Opisthokonta; Fungi; Mucoromycotina; Mucoromycotina; Mucorales; Blakeslea; Blakeslea_trispora | dietary/environmental |
| denovo2211 | Eukaryota; Opisthokonta; Fungi; Mucoromycotina; Mucoromycotina; Mucorales; Mucor; Mucor_amphibiorum | dietary/environmental |
| denovo1704 | Eukaryota; Opisthokonta; Fungi; Mucoromycotina; Mucoromycotina; Mucorales; Pilaira; Pilaira_anomala | dietary/environmental |
| denovo198 | Eukaryota; Opisthokonta; Fungi; Mucoromycotina; Mucoromycotina; Mucorales; Rhizopus | dietary/environmental |
| denovo2894 | Eukaryota; Opisthokonta; Fungi; Mucoromycotina; Mucoromycotina; Mucorales; Rhizopus; Rhizopus_oryzae | dietary/environmental |
| denovo299 | Eukaryota; Opisthokonta; Fungi; unclassified_Fungi; fungal_sp. | dietary/environmental |
| denovo857 | Eukaryota; Opisthokonta; Fungi; Zoopagales; Zoopagales; Zoopagales | dietary/environmental |
| denovo2817 | Eukaryota; Opisthokonta; Fungi; Zoopagales; Zoopagales; Zoopagales | dietary/environmental |
| denovo3253 | Eukaryota; Opisthokonta; Fungi; Zoopagales; Zoopagales; Zoopagales; Piptocephalis | dietary/environmental |
| denovo3150 | Eukaryota; Opisthokonta; Fungi; Zoopagales; Zoopagales; Zoopagales; uncultured; uncultured_Eimeriidae | dietary/environmental |
| denovo2550 | Eukaryota; Opisthokonta; Fungi; Zoopagales; Zoopagales; Zoopagales; uncultured; uncultured_Eimeriidae | dietary/environmental |
| denovo468 | Eukaryota; Opisthokonta; Metazoa; Nematoda; Chromadorea; Araeolaimida | environmental |
| denovo207 | Eukaryota; Opisthokonta; Metazoa; Nematoda; Chromadorea; Araeolaimida | environmental |
| denovo3149 | Eukaryota; Opisthokonta; Metazoa; Nematoda; Chromadorea; Desmodorida; Prodesmodora | environmental |
| denovo3012 | Eukaryota; Opisthokonta; Metazoa; Nematoda; Chromadorea; Desmodorida; Prodesmodora | environmental |
| denovo1988 | Eukaryota; Opisthokonta; Metazoa; Nematoda; Chromadorea; Desmodorida; Prodesmodora | environmental |
| denovo2597 | Eukaryota; Opisthokonta; Metazoa; Nematoda; Chromadorea; Desmodorida; Prodesmodora; Prodesmodora_sp._1338 | environmental |
| denovo1104 | Eukaryota; Opisthokonta; Metazoa; Nematoda; Chromadorea; Monhysterida; Eumonhystera | environmental |
| denovo2793 | Eukaryota; Opisthokonta; Metazoa; Nematoda; Chromadorea; Monhysterida; Paralamyctes; Paralamyctes_environmental_sample | environmental |
| denovo1951 | Eukaryota; Opisthokonta; Metazoa; Nematoda; Chromadorea; Monhysterida; Paralamyctes; Paralamyctes_environmental_sample | environmental |
| denovo2090 | Eukaryota; Opisthokonta; Metazoa; Nematoda; Chromadorea; Monhysterida; Paralamyctes; Paralamyctes_environmental_sample | environmental |
| denovo1465 | Eukaryota; Opisthokonta; Metazoa; Nematoda; Chromadorea; Monhysterida; uncultured; uncultured_eukaryote | environmental |
| denovo98 | Eukaryota; Opisthokonta; Metazoa; Nematoda; Chromadorea; Oxyurida | gut_resident |
| denovo953 | Eukaryota; Opisthokonta; Metazoa; Nematoda; Chromadorea; Oxyurida; Enterobius; Enterobius_vermicularis_(human_pinworm) | gut_resident |
| denovo814 | Eukaryota; Opisthokonta; Metazoa; Nematoda; Chromadorea; Oxyurida; Enterobius; Enterobius_vermicularis_(human_pinworm) | gut_resident |
| denovo4 | Eukaryota; Opisthokonta; Metazoa; Nematoda; Chromadorea; Oxyurida; Enterobius; Enterobius_vermicularis_(human_pinworm) | gut_resident |
| denovo3309 | Eukaryota; Opisthokonta; Metazoa; Nematoda; Chromadorea; Oxyurida; Enterobius; Enterobius_vermicularis_(human_pinworm) | gut_resident |
| denovo2714 | Eukaryota; Opisthokonta; Metazoa; Nematoda; Chromadorea; Oxyurida; Enterobius; Enterobius_vermicularis_(human_pinworm) | gut_resident |
| denovo2663 | Eukaryota; Opisthokonta; Metazoa; Nematoda; Chromadorea; Oxyurida; Enterobius; Enterobius_vermicularis_(human_pinworm) | gut_resident |
| denovo2616 | Eukaryota; Opisthokonta; Metazoa; Nematoda; Chromadorea; Oxyurida; Enterobius; Enterobius_vermicularis_(human_pinworm) | gut_resident |
| denovo253 | Eukaryota; Opisthokonta; Metazoa; Nematoda; Chromadorea; Oxyurida; Enterobius; Enterobius_vermicularis_(human_pinworm) | gut_resident |

| | | |
|---|---|---|
| denovo251 | Eukaryota; Opisthokonta; Metazoa; Nematoda; Chromadorea; Oxyurida; Enterobius; Enterobius_vermicularis_(human_pinworm) | gut_resident |
| denovo1020 | Eukaryota; Opisthokonta; Metazoa; Nematoda; Chromadorea; Oxyurida; Enterobius; Enterobius_vermicularis_(human_pinworm) | gut_resident |
| denovo1059 | Eukaryota; Opisthokonta; Metazoa; Nematoda; Chromadorea; Oxyurida; Enterobius; Enterobius_vermicularis_(human_pinworm) | gut_resident |
| denovo1281 | Eukaryota; Opisthokonta; Metazoa; Nematoda; Chromadorea; Oxyurida; Enterobius; Enterobius_vermicularis_(human_pinworm) | gut_resident |
| denovo1504 | Eukaryota; Opisthokonta; Metazoa; Nematoda; Chromadorea; Oxyurida; Enterobius; Enterobius_vermicularis_(human_pinworm) | gut_resident |
| denovo1684 | Eukaryota; Opisthokonta; Metazoa; Nematoda; Chromadorea; Oxyurida; Enterobius; Enterobius_vermicularis_(human_pinworm) | gut_resident |
| denovo1971 | Eukaryota; Opisthokonta; Metazoa; Nematoda; Chromadorea; Oxyurida; Enterobius; Enterobius_vermicularis_(human_pinworm) | gut_resident |
| denovo2082 | Eukaryota; Opisthokonta; Metazoa; Nematoda; Chromadorea; Oxyurida; Enterobius; Enterobius_vermicularis_(human_pinworm) | gut_resident |
| denovo957 | Eukaryota; Opisthokonta; Metazoa; Nematoda; Chromadorea; Rhabditida | gut_resident |
| denovo901 | Eukaryota; Opisthokonta; Metazoa; Nematoda; Chromadorea; Rhabditida | gut_resident |
| denovo3030 | Eukaryota; Opisthokonta; Metazoa; Nematoda; Chromadorea; Rhabditida | gut_resident |
| denovo2648 | Eukaryota; Opisthokonta; Metazoa; Nematoda; Chromadorea; Rhabditida | gut_resident |
| denovo2371 | Eukaryota; Opisthokonta; Metazoa; Nematoda; Chromadorea; Rhabditida | gut_resident |
| denovo23 | Eukaryota; Opisthokonta; Metazoa; Nematoda; Chromadorea; Rhabditida | gut_resident |
| denovo138 | Eukaryota; Opisthokonta; Metazoa; Nematoda; Chromadorea; Rhabditida | gut_resident |
| denovo134 | Eukaryota; Opisthokonta; Metazoa; Nematoda; Chromadorea; Rhabditida | gut_resident |
| denovo188 | Eukaryota; Opisthokonta; Metazoa; Nematoda; Chromadorea; Rhabditida | gut_resident |
| denovo1164 | Eukaryota; Opisthokonta; Metazoa; Nematoda; Chromadorea; Rhabditida | gut_resident |
| denovo1852 | Eukaryota; Opisthokonta; Metazoa; Nematoda; Chromadorea; Rhabditida | gut_resident |
| denovo412 | Eukaryota; Opisthokonta; Metazoa; Nematoda; Chromadorea; Rhabditida; Caenorhabditis | gut_resident |
| denovo772 | Eukaryota; Opisthokonta; Metazoa; Nematoda; Chromadorea; Rhabditida; Choriorhabditis | gut_resident |
| denovo767 | Eukaryota; Opisthokonta; Metazoa; Nematoda; Chromadorea; Rhabditida; Cryptistama | gut_resident |
| denovo563 | Eukaryota; Opisthokonta; Metazoa; Nematoda; Chromadorea; Rhabditida; Cryptistama | gut_resident |
| denovo122 | Eukaryota; Opisthokonta; Metazoa; Nematoda; Chromadorea; Rhabditida; Ostertagia; Ostertagia_ostertagi | gut_resident |
| denovo3298 | Eukaryota; Opisthokonta; Metazoa; Nematoda; Chromadorea; Rhabditida; Panagrolaimus | gut_resident |
| denovo3197 | Eukaryota; Opisthokonta; Metazoa; Nematoda; Chromadorea; Rhabditida; Strongyloides | gut_resident |
| denovo2651 | Eukaryota; Opisthokonta; Metazoa; Nematoda; Chromadorea; Rhabditida; Strongyloides | gut_resident |
| denovo1122 | Eukaryota; Opisthokonta; Metazoa; Nematoda; Chromadorea; Rhabditida; Strongyloides | gut_resident |
| denovo1520 | Eukaryota; Opisthokonta; Metazoa; Nematoda; Chromadorea; Rhabditida; Strongyloides | gut_resident |
| denovo2419 | Eukaryota; Opisthokonta; Metazoa; Nematoda; Chromadorea; Rhabditida; Teratocephalus | gut_resident |
| denovo197 | Eukaryota; Opisthokonta; Metazoa; Nematoda; Chromadorea; Rhabditida; Teratocephalus | gut_resident |
| denovo1004 | Eukaryota; Opisthokonta; Metazoa; Nematoda; Chromadorea; Rhabditida; Teratocephalus | gut_resident |
| denovo140 | Eukaryota; Opisthokonta; Metazoa; Nematoda; Chromadorea; Rhabditida; Viannaia | gut_resident |
| denovo830 | Eukaryota; Opisthokonta; Metazoa; Nematoda; Chromadorea; Spirurida | gut_resident |
| denovo715 | Eukaryota; Opisthokonta; Metazoa; Nematoda; Chromadorea; Spirurida | gut_resident |
| denovo682 | Eukaryota; Opisthokonta; Metazoa; Nematoda; Chromadorea; Spirurida | gut_resident |
| denovo638 | Eukaryota; Opisthokonta; Metazoa; Nematoda; Chromadorea; Spirurida | gut_resident |
| denovo529 | Eukaryota; Opisthokonta; Metazoa; Nematoda; Chromadorea; Spirurida | gut_resident |
| denovo495 | Eukaryota; Opisthokonta; Metazoa; Nematoda; Chromadorea; Spirurida | gut_resident |
| denovo3294 | Eukaryota; Opisthokonta; Metazoa; Nematoda; Chromadorea; Spirurida | gut_resident |

| | | |
|---|---|---|
| denovo3236 | Eukaryota; Opisthokonta; Metazoa; Nematoda; Chromadorea; Spirurida | gut_resident |
| denovo3234 | Eukaryota; Opisthokonta; Metazoa; Nematoda; Chromadorea; Spirurida | gut_resident |
| denovo3232 | Eukaryota; Opisthokonta; Metazoa; Nematoda; Chromadorea; Spirurida | gut_resident |
| denovo3045 | Eukaryota; Opisthokonta; Metazoa; Nematoda; Chromadorea; Spirurida | gut_resident |
| denovo2723 | Eukaryota; Opisthokonta; Metazoa; Nematoda; Chromadorea; Spirurida | gut_resident |
| denovo2628 | Eukaryota; Opisthokonta; Metazoa; Nematoda; Chromadorea; Spirurida | gut_resident |
| denovo2619 | Eukaryota; Opisthokonta; Metazoa; Nematoda; Chromadorea; Spirurida | gut_resident |
| denovo2568 | Eukaryota; Opisthokonta; Metazoa; Nematoda; Chromadorea; Spirurida | gut_resident |
| denovo2565 | Eukaryota; Opisthokonta; Metazoa; Nematoda; Chromadorea; Spirurida | gut_resident |
| denovo2432 | Eukaryota; Opisthokonta; Metazoa; Nematoda; Chromadorea; Spirurida | gut_resident |
| denovo21 | Eukaryota; Opisthokonta; Metazoa; Nematoda; Chromadorea; Spirurida | gut_resident |
| denovo119 | Eukaryota; Opisthokonta; Metazoa; Nematoda; Chromadorea; Spirurida | gut_resident |
| denovo1093 | Eukaryota; Opisthokonta; Metazoa; Nematoda; Chromadorea; Spirurida | gut_resident |
| denovo1065 | Eukaryota; Opisthokonta; Metazoa; Nematoda; Chromadorea; Spirurida | gut_resident |
| denovo1387 | Eukaryota; Opisthokonta; Metazoa; Nematoda; Chromadorea; Spirurida | gut_resident |
| denovo1674 | Eukaryota; Opisthokonta; Metazoa; Nematoda; Chromadorea; Spirurida | gut_resident |
| denovo1786 | Eukaryota; Opisthokonta; Metazoa; Nematoda; Chromadorea; Spirurida | gut_resident |
| denovo1758 | Eukaryota; Opisthokonta; Metazoa; Nematoda; Chromadorea; Spirurida | gut_resident |
| denovo2248 | Eukaryota; Opisthokonta; Metazoa; Nematoda; Chromadorea; Spirurida | gut_resident |
| denovo2247 | Eukaryota; Opisthokonta; Metazoa; Nematoda; Chromadorea; Spirurida | gut_resident |
| denovo141 | Eukaryota; Opisthokonta; Metazoa; Nematoda; Chromadorea; Spirurida; Physaloptera | gut_resident |
| denovo1807 | Eukaryota; Opisthokonta; Metazoa; Nematoda; Chromadorea; Spirurida; Physaloptera | gut_resident |
| denovo2001 | Eukaryota; Opisthokonta; Metazoa; Nematoda; Chromadorea; Spirurida; Physaloptera | gut_resident |
| denovo3083 | Eukaryota; Opisthokonta; Metazoa; Nematoda; Chromadorea; Tylenchida | plant_parasite |
| denovo1670 | Eukaryota; Opisthokonta; Metazoa; Nematoda; Chromadorea; Tylenchida | plant_parasite |
| denovo1790 | Eukaryota; Opisthokonta; Metazoa; Nematoda; Chromadorea; Tylenchida; Aphelenchoides | plant_parasite |
| denovo972 | Eukaryota; Opisthokonta; Metazoa; Nematoda; Chromadorea; Tylenchida; Schistonchus | plant_parasite |
| denovo838 | Eukaryota; Opisthokonta; Metazoa; Nematoda; Chromadorea; Tylenchida; Schistonchus | plant_parasite |
| denovo2600 | Eukaryota; Opisthokonta; Metazoa; Nematoda; Chromadorea; Tylenchida; Schistonchus | plant_parasite |
| denovo2555 | Eukaryota; Opisthokonta; Metazoa; Nematoda; Chromadorea; Tylenchida; Schistonchus | plant_parasite |
| denovo1374 | Eukaryota; Opisthokonta; Metazoa; Nematoda; Chromadorea; Tylenchida; Schistonchus | plant_parasite |
| denovo1424 | Eukaryota; Opisthokonta; Metazoa; Nematoda; Chromadorea; Tylenchida; Schistonchus | plant_parasite |
| denovo1869 | Eukaryota; Opisthokonta; Metazoa; Nematoda; Chromadorea; Tylenchida; Schistonchus | plant_parasite |
| denovo1938 | Eukaryota; Opisthokonta; Metazoa; Nematoda; Chromadorea; Tylenchida; Schistonchus | plant_parasite |
| denovo562 | Eukaryota; Opisthokonta; Metazoa; Nematoda; Chromadorea; Tylenchida; Sphaerularia | insect_parasite |
| denovo1407 | Eukaryota; Opisthokonta; Metazoa; Nematoda; Enoplea; Capillaria; Capillaria | gut_resident |
| denovo2778 | Eukaryota; Opisthokonta; Metazoa; Nematoda; Enoplea; Enoplida | environmental |
| denovo976 | Eukaryota; Opisthokonta; Metazoa; Nematoda; Enoplea; Nematoda; Nematoda; Nematoda_environmental_sample | environmental |
| denovo707 | Eukaryota; Opisthokonta; Metazoa; Nematoda; Enoplea; Trichuris; Trichuris; Trichuris_trichiura_(human_whipworm) | gut_resident |
| denovo326 | Eukaryota; Opisthokonta; Metazoa; Platyhelminthes; Rhabditophora; Lecithoepitheliata; Geocentrophora | environmental |

| | | |
|---|---|---|
| denovo115 | Eukaryota; Opisthokonta; Metazoa; Platyhelminthes; Rhabditophora; Lecithoepitheliata; Geocentrophora | environmental |
| denovo3153 | Eukaryota; Rhizaria | environmental |
| denovo3262 | Eukaryota; Rhizaria; Cercozoa | environmental |
| denovo3015 | Eukaryota; Rhizaria; Cercozoa | environmental |
| denovo2020 | Eukaryota; Rhizaria; Cercozoa | environmental |
| denovo2287 | Eukaryota; Rhizaria; Cercozoa | environmental |
| denovo987 | Eukaryota; Rhizaria; Cercozoa; Cercomonadida; Sainouridae; Cholamonas; Cholamonas_cyrtodiopsidis | environmental |
| denovo872 | Eukaryota; Rhizaria; Cercozoa; Cercomonadida; Sainouridae; Cholamonas; Cholamonas_cyrtodiopsidis | environmental |
| denovo825 | Eukaryota; Rhizaria; Cercozoa; Cercomonadida; Sainouridae; Cholamonas; Cholamonas_cyrtodiopsidis | environmental |
| denovo763 | Eukaryota; Rhizaria; Cercozoa; Cercomonadida; Sainouridae; Cholamonas; Cholamonas_cyrtodiopsidis | environmental |
| denovo735 | Eukaryota; Rhizaria; Cercozoa; Cercomonadida; Sainouridae; Cholamonas; Cholamonas_cyrtodiopsidis | environmental |
| denovo718 | Eukaryota; Rhizaria; Cercozoa; Cercomonadida; Sainouridae; Cholamonas; Cholamonas_cyrtodiopsidis | environmental |
| denovo608 | Eukaryota; Rhizaria; Cercozoa; Cercomonadida; Sainouridae; Cholamonas; Cholamonas_cyrtodiopsidis | environmental |
| denovo3114 | Eukaryota; Rhizaria; Cercozoa; Cercomonadida; Sainouridae; Cholamonas; Cholamonas_cyrtodiopsidis | environmental |
| denovo2785 | Eukaryota; Rhizaria; Cercozoa; Cercomonadida; Sainouridae; Cholamonas; Cholamonas_cyrtodiopsidis | environmental |
| denovo2736 | Eukaryota; Rhizaria; Cercozoa; Cercomonadida; Sainouridae; Cholamonas; Cholamonas_cyrtodiopsidis | environmental |
| denovo2535 | Eukaryota; Rhizaria; Cercozoa; Cercomonadida; Sainouridae; Cholamonas; Cholamonas_cyrtodiopsidis | environmental |
| denovo1017 | Eukaryota; Rhizaria; Cercozoa; Cercomonadida; Sainouridae; Cholamonas; Cholamonas_cyrtodiopsidis | environmental |
| denovo1223 | Eukaryota; Rhizaria; Cercozoa; Cercomonadida; Sainouridae; Cholamonas; Cholamonas_cyrtodiopsidis | environmental |
| denovo1729 | Eukaryota; Rhizaria; Cercozoa; Cercomonadida; Sainouridae; Cholamonas; Cholamonas_cyrtodiopsidis | environmental |
| denovo1799 | Eukaryota; Rhizaria; Cercozoa; Cercomonadida; Sainouridae; Cholamonas; Cholamonas_cyrtodiopsidis | environmental |
| denovo2173 | Eukaryota; Rhizaria; Cercozoa; Cercomonadida; Sainouridae; Cholamonas; Cholamonas_cyrtodiopsidis | environmental |
| denovo930 | Eukaryota; Rhizaria; Cercozoa; Cercomonadidae; Cercomonas; Cercomonas; Cercomonas | environmental |
| denovo897 | Eukaryota; Rhizaria; Cercozoa; Cercomonadidae; Cercomonas; Cercomonas; Cercomonas | environmental |
| denovo765 | Eukaryota; Rhizaria; Cercozoa; Cercomonadidae; Cercomonas; Cercomonas; Cercomonas | environmental |
| denovo729 | Eukaryota; Rhizaria; Cercozoa; Cercomonadidae; Cercomonas; Cercomonas; Cercomonas | environmental |
| denovo554 | Eukaryota; Rhizaria; Cercozoa; Cercomonadidae; Cercomonas; Cercomonas; Cercomonas | environmental |
| denovo3255 | Eukaryota; Rhizaria; Cercozoa; Cercomonadidae; Cercomonas; Cercomonas; Cercomonas | environmental |
| denovo3205 | Eukaryota; Rhizaria; Cercozoa; Cercomonadidae; Cercomonas; Cercomonas; Cercomonas | environmental |
| denovo3004 | Eukaryota; Rhizaria; Cercozoa; Cercomonadidae; Cercomonas; Cercomonas; Cercomonas | environmental |
| denovo2795 | Eukaryota; Rhizaria; Cercozoa; Cercomonadidae; Cercomonas; Cercomonas; Cercomonas | environmental |
| denovo2575 | Eukaryota; Rhizaria; Cercozoa; Cercomonadidae; Cercomonas; Cercomonas; Cercomonas | environmental |
| denovo2510 | Eukaryota; Rhizaria; Cercozoa; Cercomonadidae; Cercomonas; Cercomonas; Cercomonas | environmental |
| denovo211 | Eukaryota; Rhizaria; Cercozoa; Cercomonadidae; Cercomonas; Cercomonas; Cercomonas | environmental |
| denovo1250 | Eukaryota; Rhizaria; Cercozoa; Cercomonadidae; Cercomonas; Cercomonas; Cercomonas | environmental |
| denovo1247 | Eukaryota; Rhizaria; Cercozoa; Cercomonadidae; Cercomonas; Cercomonas; Cercomonas | environmental |
| denovo1451 | Eukaryota; Rhizaria; Cercozoa; Cercomonadidae; Cercomonas; Cercomonas; Cercomonas | environmental |
| denovo1780 | Eukaryota; Rhizaria; Cercozoa; Cercomonadidae; Cercomonas; Cercomonas; Cercomonas | environmental |
| denovo1935 | Eukaryota; Rhizaria; Cercozoa; Cercomonadidae; Cercomonas; Cercomonas; Cercomonas | environmental |
| denovo1975 | Eukaryota; Rhizaria; Cercozoa; Cercomonadidae; Cercomonas; Cercomonas; Cercomonas | environmental |

| | | |
|---|---|---|
| denovo2077 | Eukaryota; Rhizaria; Cercozoa; Cercomonadidae; Cercomonas; Cercomonas; Cercomonas | environmental |
| denovo2212 | Eukaryota; Rhizaria; Cercozoa; Cercomonadidae; Cercomonas; Cercomonas; Cercomonas | environmental |
| denovo2171 | Eukaryota; Rhizaria; Cercozoa; Cercomonadidae; Cercomonas; Cercomonas; Cercomonas | environmental |
| denovo2264 | Eukaryota; Rhizaria; Cercozoa; Cercomonadidae; Cercomonas; Cercomonas; Cercomonas | environmental |
| denovo2260 | Eukaryota; Rhizaria; Cercozoa; Cercomonadidae; Cercomonas; Cercomonas; Cercomonas | environmental |
| denovo1261 | Eukaryota; Rhizaria; Cercozoa; Cercomonadidae; Cercomonas; Cercomonas; Cercomonas; Cercomonas_plasmodialis | environmental |
| denovo2324 | Eukaryota; Rhizaria; Cercozoa; Cercomonadidae; Cercomonas; Cercomonas; Cercomonas; uncultured_eukaryote | environmental |
| denovo1474 | Eukaryota; Rhizaria; Cercozoa; Cercomonadidae; Cercomonas; Cercomonas; Cercomonas; uncultured_eukaryote | environmental |
| denovo1494 | Eukaryota; Rhizaria; Cercozoa; Cercomonadidae; Eocercomonas; Eocercomonas; Eocercomonas | environmental |
| denovo1505 | Eukaryota; Rhizaria; Cercozoa; Cercomonadidae; Eocercomonas; Eocercomonas; Eocercomonas | environmental |
| denovo2381 | Eukaryota; Rhizaria; Cercozoa; Cercomonadidae; uncultured; uncultured; uncultured | environmental |
| denovo1676 | Eukaryota; Rhizaria; Cercozoa; Glissomonadida | environmental |
| denovo1718 | Eukaryota; Rhizaria; Cercozoa; Glissomonadida | environmental |
| denovo2262 | Eukaryota; Rhizaria; Cercozoa; Glissomonadida; Amb-18S-1124; Amb-18S-1124; Amb-18S-1124 | environmental |
| denovo2596 | Eukaryota; Rhizaria; Cercozoa; Glissomonadida; Bodomorpha; Bodomorpha; Bodomorpha; uncultured_cercozoan | environmental |
| denovo2551 | Eukaryota; Rhizaria; Cercozoa; Glissomonadida; Bodomorpha; Bodomorpha; Bodomorpha; uncultured_cercozoan | environmental |
| denovo1293 | Eukaryota; Rhizaria; Cercozoa; Glissomonadida; Bodomorpha; Bodomorpha; Bodomorpha; uncultured_cercozoan | environmental |
| denovo1027 | Eukaryota; Rhizaria; Cercozoa; Glissomonadida; Heteromita; Heteromita; Heteromita | environmental |
| denovo1095 | Eukaryota; Rhizaria; Cercozoa; Glissomonadida; Heteromita; Heteromita; Heteromita | environmental |
| denovo1243 | Eukaryota; Rhizaria; Cercozoa; Glissomonadida; Heteromita; Heteromita; Heteromita | environmental |
| denovo1355 | Eukaryota; Rhizaria; Cercozoa; Glissomonadida; Heteromita; Heteromita; Heteromita | environmental |
| denovo1734 | Eukaryota; Rhizaria; Cercozoa; Glissomonadida; Heteromita; Heteromita; Heteromita | environmental |
| denovo1723 | Eukaryota; Rhizaria; Cercozoa; Glissomonadida; Heteromita; Heteromita; Heteromita | environmental |
| denovo1794 | Eukaryota; Rhizaria; Cercozoa; Glissomonadida; Heteromita; Heteromita; Heteromita | environmental |
| denovo1996 | Eukaryota; Rhizaria; Cercozoa; Glissomonadida; Heteromita; Heteromita; Heteromita | environmental |
| denovo991 | Eukaryota; Rhizaria; Cercozoa; Glissomonadida; Heteromita; Heteromita; Heteromita; uncultured_cercozoan | environmental |
| denovo710 | Eukaryota; Rhizaria; Cercozoa; Glissomonadida; Heteromita; Heteromita; Heteromita; uncultured_cercozoan | environmental |
| denovo3179 | Eukaryota; Rhizaria; Cercozoa; Glissomonadida; Heteromita; Heteromita; Heteromita; uncultured_cercozoan | environmental |
| denovo2955 | Eukaryota; Rhizaria; Cercozoa; Glissomonadida; Heteromita; Heteromita; Heteromita; uncultured_cercozoan | environmental |
| denovo1747 | Eukaryota; Rhizaria; Cercozoa; Glissomonadida; Heteromita; Heteromita; Heteromita; uncultured_cercozoan | environmental |
| denovo1876 | Eukaryota; Rhizaria; Cercozoa; Glissomonadida; Heteromita; Heteromita; Heteromita; uncultured_cercozoan | environmental |
| denovo1500 | Eukaryota; Rhizaria; Cercozoa; Glissomonadida; Proleptomonas; Proleptomonas; Proleptomonas; Proleptomonas_faecicola | environmental |
| denovo1592 | Eukaryota; Rhizaria; Cercozoa; Glissomonadida; Proleptomonas; Proleptomonas; Proleptomonas; Proleptomonas_faecicola | environmental |
| denovo2970 | Eukaryota; Rhizaria; Cercozoa; Glissomonadida; uncultured; uncultured; uncultured; uncultured_eukaryote | environmental |
| denovo1590 | Eukaryota; Rhizaria; Cercozoa; Glissomonadida; uncultured; uncultured; uncultured; uncultured_eukaryote | environmental |
| denovo1845 | Eukaryota; Rhizaria; Cercozoa; Imbricatea; Silicofilosea; Euglyphida | environmental |
| denovo2899 | Eukaryota; Rhizaria; Cercozoa; Imbricatea; Silicofilosea; Euglyphida; 13-1.8; uncultured_cercozoan | environmental |
| denovo2794 | Eukaryota; Rhizaria; Cercozoa; Imbricatea; Silicofilosea; Euglyphida; 13-1.8; uncultured_cercozoan | environmental |
| denovo1171 | Eukaryota; Rhizaria; Cercozoa; Imbricatea; Silicofilosea; Euglyphida; 13-1.8; uncultured_cercozoan | environmental |
| denovo3000 | Eukaryota; Rhizaria; Cercozoa; Imbricatea; Silicofilosea; Euglyphida; Amb-18S-1480 | environmental |

| | | |
|---|---|---|
| denovo766 | Eukaryota; Rhizaria; Cercozoa; Imbricatea; Silicofilosea; Euglyphida; environmental_samples_<Euglyphida>; uncultured_Euglyphida | environmental |
| denovo2826 | Eukaryota; Rhizaria; Cercozoa; Imbricatea; Silicofilosea; Euglyphida; environmental_samples_<Euglyphida>; uncultured_Euglyphida | environmental |
| denovo2218 | Eukaryota; Rhizaria; Cercozoa; Imbricatea; Silicofilosea; Euglyphida; environmental_samples_<Euglyphida>; uncultured_Euglyphida | environmental |
| denovo622 | Eukaryota; Rhizaria; Cercozoa; Imbricatea; Silicofilosea; Euglyphida; Euglypha; Euglypha_rotunda | environmental |
| denovo3268 | Eukaryota; Rhizaria; Cercozoa; Imbricatea; Silicofilosea; Euglyphida; Euglypha; Euglypha_rotunda | environmental |
| denovo3241 | Eukaryota; Rhizaria; Cercozoa; Imbricatea; Silicofilosea; Euglyphida; Euglypha; Euglypha_rotunda | environmental |
| denovo2946 | Eukaryota; Rhizaria; Cercozoa; Imbricatea; Silicofilosea; Euglyphida; Euglypha; Euglypha_rotunda | environmental |
| denovo2729 | Eukaryota; Rhizaria; Cercozoa; Imbricatea; Silicofilosea; Euglyphida; Euglypha; Euglypha_rotunda | environmental |
| denovo2629 | Eukaryota; Rhizaria; Cercozoa; Imbricatea; Silicofilosea; Euglyphida; Euglypha; Euglypha_rotunda | environmental |
| denovo2572 | Eukaryota; Rhizaria; Cercozoa; Imbricatea; Silicofilosea; Euglyphida; Euglypha; Euglypha_rotunda | environmental |
| denovo1268 | Eukaryota; Rhizaria; Cercozoa; Imbricatea; Silicofilosea; Euglyphida; Euglypha; Euglypha_rotunda | environmental |
| denovo1860 | Eukaryota; Rhizaria; Cercozoa; Imbricatea; Silicofilosea; Euglyphida; Euglypha; Euglypha_rotunda | environmental |
| denovo2185 | Eukaryota; Rhizaria; Cercozoa; Imbricatea; Silicofilosea; Euglyphida; Euglypha; Euglypha_rotunda | environmental |
| denovo1560 | Eukaryota; Rhizaria; Cercozoa; Imbricatea; Silicofilosea; Euglyphida; Paulinella | environmental |
| denovo3011 | Eukaryota; Rhizaria; Cercozoa; Imbricatea; Silicofilosea; Euglyphida; Paulinella; uncultured_eukaryote | environmental |
| denovo1829 | Eukaryota; Rhizaria; Cercozoa; Imbricatea; Silicofilosea; Incertae_Sedis; Tracheleuglypha; Tracheleuglypha_dentata | environmental |
| denovo2310 | Eukaryota; Rhizaria; Cercozoa; Imbricatea; Silicofilosea; Thaumatomonadida | environmental |
| denovo3049 | Eukaryota; Rhizaria; Cercozoa; Imbricatea; Silicofilosea; Thaumatomonadida; Gyromitus | environmental |
| denovo2968 | Eukaryota; Rhizaria; Cercozoa; Imbricatea; Silicofilosea; Thaumatomonadida; Gyromitus | environmental |
| denovo1267 | Eukaryota; Rhizaria; Cercozoa; Imbricatea; Silicofilosea; Thaumatomonadida; Gyromitus | environmental |
| denovo582 | Eukaryota; Rhizaria; Cercozoa; Imbricatea; Silicofilosea; Thaumatomonadida; Thaumatomonas | environmental |
| denovo1227 | Eukaryota; Rhizaria; Cercozoa; Imbricatea; Spongomonadida; Spongomonas; Spongomonas | environmental |
| denovo467 | Eukaryota; Rhizaria; Cercozoa; Incertae_Sedis; Incertae_Sedis; Incertae_Sedis; Cholamonas; Cholamonas_cyrtodiopsidis | environmental |
| denovo333 | Eukaryota; Rhizaria; Cercozoa; Incertae_Sedis; Incertae_Sedis; Incertae_Sedis; Cholamonas; Cholamonas_cyrtodiopsidis | environmental |
| denovo1509 | Eukaryota; Rhizaria; Cercozoa; Incertae_Sedis; Incertae_Sedis; Incertae_Sedis; Gymnophrys | environmental |
| denovo1636 | Eukaryota; Rhizaria; Cercozoa; Incertae_Sedis; Incertae_Sedis; Incertae_Sedis; Gymnophrys; Athalamea_environmental_sample | environmental |
| denovo975 | Eukaryota; Rhizaria; Cercozoa; Metromonadea; Metopion; Metopion; Metopion | environmental |
| denovo3064 | Eukaryota; Rhizaria; Cercozoa; Metromonadea; Metopion; Metopion; Metopion | environmental |
| denovo2818 | Eukaryota; Rhizaria; Cercozoa; Metromonadea; Metopion; Metopion; Metopion | environmental |
| denovo2681 | Eukaryota; Rhizaria; Cercozoa; Metromonadea; Metopion; Metopion; Metopion | environmental |
| denovo1094 | Eukaryota; Rhizaria; Cercozoa; Metromonadea; Metopion; Metopion; Metopion | environmental |
| denovo1291 | Eukaryota; Rhizaria; Cercozoa; Metromonadea; Metopion; Metopion; Metopion | environmental |
| denovo1471 | Eukaryota; Rhizaria; Cercozoa; Metromonadea; Metopion; Metopion; Metopion | environmental |
| denovo1513 | Eukaryota; Rhizaria; Cercozoa; Metromonadea; Metopion; Metopion; Metopion | environmental |
| denovo415 | Eukaryota; Rhizaria; Cercozoa; Phytomyxea | environmental |
| denovo720 | Eukaryota; Rhizaria; Cercozoa; Phytomyxea; Polymyxa; Polymyxa; Polymyxa; uncultured_plasmodiophorid | environmental |
| denovo1626 | Eukaryota; Rhizaria; Cercozoa; RM2-SGM58 | environmental |
| denovo1421 | Eukaryota; Rhizaria; Cercozoa; RT5iin19 | environmental |
| denovo1545 | Eukaryota; Rhizaria; Cercozoa; RT5iin19 | environmental |
| denovo2267 | Eukaryota; Rhizaria; Cercozoa; RT5iin19 | environmental |

| | | |
|---|---|---|
| denovo741 | Eukaryota; Rhizaria; Cercozoa; Thecofilosea | environmental |
| denovo3237 | Eukaryota; Rhizaria; Cercozoa; Thecofilosea | environmental |
| denovo1198 | Eukaryota; Rhizaria; Cercozoa; Thecofilosea | environmental |
| denovo882 | Eukaryota; Rhizaria; Cercozoa; Thecofilosea; Amb-18S-431; uncultured; uncultured; uncultured_eukaryote | environmental |
| denovo3204 | Eukaryota; Rhizaria; Cercozoa; Thecofilosea; Amb-18S-431; uncultured; uncultured; uncultured_eukaryote | environmental |
| denovo934 | Eukaryota; Rhizaria; Cercozoa; Thecofilosea; Cryomonadida; Protaspidae; Protaspis | environmental |
| denovo3055 | Eukaryota; Rhizaria; Cercozoa; Thecofilosea; Cryomonadida; Rhizaspididae; Rhogostoma; uncultured_eukaryote | environmental |
| denovo2701 | Eukaryota; Rhizaria; Cercozoa; Thecofilosea; Cryomonadida; Rhizaspididae; Rhogostoma; uncultured_eukaryote | environmental |
| denovo1000 | Eukaryota; Rhizaria; Cercozoa; Thecofilosea; Cryomonadida; Rhizaspididae; Rhogostoma; uncultured_eukaryote | environmental |
| denovo2203 | Eukaryota; Rhizaria; Cercozoa; Thecofilosea; Incertae_Sedis; Incertae_Sedis; Lecythium; Lecythium_terrestris | environmental |
| denovo811 | Eukaryota; Rhizaria; Cercozoa; Thecofilosea; uncultured; uncultured; uncultured; uncultured_eukaryote | environmental |
| denovo2980 | Eukaryota; Rhizaria; Cercozoa; Thecofilosea; uncultured; uncultured; uncultured; uncultured_eukaryote | environmental |
| denovo2781 | Eukaryota; Rhizaria; Cercozoa; Thecofilosea; uncultured; uncultured; uncultured; uncultured_eukaryote | environmental |
| denovo2727 | Eukaryota; Rhizaria; Cercozoa; Thecofilosea; uncultured; uncultured; uncultured; uncultured_eukaryote | environmental |
| denovo2690 | Eukaryota; Rhizaria; Cercozoa; Thecofilosea; uncultured; uncultured; uncultured; uncultured_eukaryote | environmental |
| denovo2418 | Eukaryota; Rhizaria; Cercozoa; Thecofilosea; uncultured; uncultured; uncultured; uncultured_eukaryote | environmental |
| denovo2360 | Eukaryota; Rhizaria; Cercozoa; Thecofilosea; uncultured; uncultured; uncultured; uncultured_eukaryote | environmental |
| denovo1157 | Eukaryota; Rhizaria; Cercozoa; Thecofilosea; uncultured; uncultured; uncultured; uncultured_eukaryote | environmental |
| denovo1150 | Eukaryota; Rhizaria; Cercozoa; Thecofilosea; uncultured; uncultured; uncultured; uncultured_eukaryote | environmental |
| denovo1264 | Eukaryota; Rhizaria; Cercozoa; Thecofilosea; uncultured; uncultured; uncultured; uncultured_eukaryote | environmental |
| denovo1368 | Eukaryota; Rhizaria; Cercozoa; Thecofilosea; uncultured; uncultured; uncultured; uncultured_eukaryote | environmental |
| denovo1464 | Eukaryota; Rhizaria; Cercozoa; Thecofilosea; uncultured; uncultured; uncultured; uncultured_eukaryote | environmental |
| denovo2108 | Eukaryota; Rhizaria; Cercozoa; Thecofilosea; uncultured; uncultured; uncultured; uncultured_eukaryote | environmental |
| denovo2195 | Eukaryota; Rhizaria; Cercozoa; Thecofilosea; uncultured; uncultured; uncultured; uncultured_eukaryote | environmental |
| denovo1259 | Eukaryota; Rhizaria; Cercozoa; uncultured; uncultured; Cercozoa; Cercozoa; Cercozoa_sp._ATCC_50378 | environmental |
| denovo1033 | Eukaryota; Rhizaria; Cercozoa; uncultured; uncultured; uncultured; uncultured | environmental |
| denovo923 | Eukaryota; Rhizaria; Cercozoa; Vampyrellidae | environmental |
| denovo823 | Eukaryota; Rhizaria; Cercozoa; Vampyrellidae | environmental |
| denovo3052 | Eukaryota; Rhizaria; Cercozoa; Vampyrellidae | environmental |
| denovo2540 | Eukaryota; Rhizaria; Cercozoa; Vampyrellidae | environmental |
| denovo1668 | Eukaryota; Rhizaria; Cercozoa; Vampyrellidae | environmental |
| denovo2571 | Eukaryota; Rhizaria; Cercozoa; Vampyrellidae; uncultured; uncultured; uncultured | environmental |
| denovo1855 | Eukaryota; Rhizaria; Cercozoa; Vampyrellidae; uncultured; uncultured; uncultured; uncultured_eukaryote | environmental |
| denovo2692 | Eukaryota; Stramenopiles; Bacillariophyta | environmental |
| denovo2630 | Eukaryota; Stramenopiles; Bacillariophyta | environmental |
| denovo1378 | Eukaryota; Stramenopiles; Bacillariophyta | environmental |
| denovo841 | Eukaryota; Stramenopiles; Incertae_Sedis; Incertae_Sedis; Incertae_Sedis; Incertae_Sedis; Blastocystis; Blastocystis_ST1 | gut_resident |
| denovo512 | Eukaryota; Stramenopiles; Incertae_Sedis; Incertae_Sedis; Incertae_Sedis; Incertae_Sedis; Blastocystis; Blastocystis_ST1 | gut_resident |
| denovo361 | Eukaryota; Stramenopiles; Incertae_Sedis; Incertae_Sedis; Incertae_Sedis; Incertae_Sedis; Blastocystis; Blastocystis_ST1 | gut_resident |
| denovo2834 | Eukaryota; Stramenopiles; Incertae_Sedis; Incertae_Sedis; Incertae_Sedis; Incertae_Sedis; Blastocystis; Blastocystis_ST1 | gut_resident |

| | | |
|---|---|---|
| denovo229 | Eukaryota; Stramenopiles; Incertae_Sedis; Incertae_Sedis; Incertae_Sedis; Incertae_Sedis; Blastocystis; Blastocystis_ST1 | gut_resident |
| denovo3163 | Eukaryota; Stramenopiles; Incertae_Sedis; Incertae_Sedis; Incertae_Sedis; Incertae_Sedis; Blastocystis; Blastocystis_ST2 | gut_resident |
| denovo2430 | Eukaryota; Stramenopiles; Incertae_Sedis; Incertae_Sedis; Incertae_Sedis; Incertae_Sedis; Blastocystis; Blastocystis_ST2 | gut_resident |
| denovo1455 | Eukaryota; Stramenopiles; Incertae_Sedis; Incertae_Sedis; Incertae_Sedis; Incertae_Sedis; Blastocystis; Blastocystis_ST2 | gut_resident |
| denovo550 | Eukaryota; Stramenopiles; Incertae_Sedis; Incertae_Sedis; Incertae_Sedis; Incertae_Sedis; Blastocystis; Blastocystis_ST3 | gut_resident |
| denovo511 | Eukaryota; Stramenopiles; Incertae_Sedis; Incertae_Sedis; Incertae_Sedis; Incertae_Sedis; Blastocystis; Blastocystis_ST3 | gut_resident |
| denovo491 | Eukaryota; Stramenopiles; Incertae_Sedis; Incertae_Sedis; Incertae_Sedis; Incertae_Sedis; Blastocystis; Blastocystis_ST3 | gut_resident |
| denovo377 | Eukaryota; Stramenopiles; Incertae_Sedis; Incertae_Sedis; Incertae_Sedis; Incertae_Sedis; Blastocystis; Blastocystis_ST3 | gut_resident |
| denovo319 | Eukaryota; Stramenopiles; Incertae_Sedis; Incertae_Sedis; Incertae_Sedis; Incertae_Sedis; Blastocystis; Blastocystis_ST3 | gut_resident |
| denovo288 | Eukaryota; Stramenopiles; Incertae_Sedis; Incertae_Sedis; Incertae_Sedis; Incertae_Sedis; Blastocystis; Blastocystis_ST3 | gut_resident |
| denovo2227 | Eukaryota; Stramenopiles; Incertae_Sedis; Incertae_Sedis; Incertae_Sedis; Incertae_Sedis; Blastocystis; Blastocystis_ST8 | gut_resident |
| denovo2842 | Eukaryota; Stramenopiles; Ochrophyta; Chrysophyceae | environmental |
| denovo684 | Eukaryota; Stramenopiles; Ochrophyta; Chrysophyceae; Chromulinales; Spumella; Spumella | environmental |
| denovo2558 | Eukaryota; Stramenopiles; Ochrophyta; Chrysophyceae; Chromulinales; Spumella; Spumella | environmental |
| denovo1081 | Eukaryota; Stramenopiles; Ochrophyta; Chrysophyceae; uncultured; uncultured; uncultured; marine_metagenome | environmental |
| denovo892 | Eukaryota; Stramenopiles; Peronosporomycetes | environmental |
| denovo2653 | Eukaryota; Stramenopiles; Peronosporomycetes | environmental |
| denovo2838 | Eukaryota; Stramenopiles; Peronosporomycetes; Haliphthoros; Haliphthoros; Haliphthoros; Haliphthoros | environmental |
| denovo685 | Eukaryota; Stramenopiles; Peronosporomycetes; Phytophthora; Phytophthora; Phytophthora; Phytophthora; Phytophthora_agathidicida | environmental |
| denovo1319 | Eukaryota; Stramenopiles; Peronosporomycetes; Phytopythium; Phytopythium; Phytopythium; Phytopythium | environmental |
| denovo1802 | Eukaryota; Stramenopiles; Peronosporomycetes; Pythium; Pythium; Pythium; Pythium | environmental |
| denovo1986 | Eukaryota; Stramenopiles; Peronosporomycetes; Pythium; Pythium; Pythium; Pythium | environmental |
| denovo2292 | Eukaryota; Stramenopiles; Peronosporomycetes; Pythium; Pythium; Pythium; Pythium; Pythium_ultimum_DAOM_BR144 | environmental |
| denovo344 | Eukaryota; Stramenopiles; Peronosporomycetes; Pythium; Pythium; Pythium; Pythium; uncultured_Eimeriidae | environmental |
| denovo2682 | Eukaryota; Stramenopiles; Peronosporomycetes; uncultured; uncultured; uncultured; uncultured | environmental |
| denovo2489 | Eukaryota; Stramenopiles; Peronosporomycetes; uncultured; uncultured; uncultured; uncultured | environmental |
| denovo1542 | Eukaryota; uncultured; uncultured; uncultured; uncultured; uncultured; uncultured; uncultured_Eimeriidae | environmental |

Supplementary Table 4: LEfSe results

LEfSe results for blastocystis positive and negative individuals at the Bacterial genus level

| Genus | log(highest_class_average) | class | LDA_effect_size | p_value | FDR_corrected |
|---|---|---|---|---|---|
| Bacteria_Firmicutes_Erysipelotrichi_Erysipelotrichales_Erysipelotrichaceae_CCMM_g | 3.87698718443 | positive | 3.30702044389 | 0.00502220089365 | 6.434695e-03 |
| Bacteria_Bacteroidetes_Bacteroidia_Bacteroidales_AC160630_f_PAC001134_g | 3.33176461264 | positive | 3.03287552155 | 7.08865789147e-06 | 4.632274e-05 |
| Bacteria_Firmicutes_Clostridia_Clostridiales_Christensenellaceae_PAC001141_g | 3.65641765365 | positive | 3.16030606318 | 0.00275952689757 | 3.771353e-03 |
| Bacteria_Firmicutes_Clostridia_Clostridiales_Clostridiaceae_Clostridium | 3.6327946083 | positive | 3.17323954491 | 0.00365284664683 | 4.831184e-03 |
| Bacteria_Verrucomicrobia_Kiritimatiellae_EF436358_o_EF436358_f_EF436358_g | 4.55404474494 | positive | 4.14398871721 | 8.72978617654e-05 | 2.237008e-04 |
| Bacteria_Firmicutes_Clostridia_Clostridiales_Ruminococcaceae_Oscillibacter | 4.62517521143 | positive | 4.23599892319 | 9.57254007514e-06 | 4.905927e-05 |
| Bacteria_Firmicutes_Clostridia_Clostridiales_Ruminococcaceae_NHOC_g | 4.14983469672 | positive | 3.53109933388 | 0.0117335733452 | 1.336324e-02 |
| Bacteria_Proteobacteria_Alphaproteobacteria_Rhodospirillales_Rhodospirillaceae_PAC001322_g | 3.55307353064 | positive | 3.31729508437 | 1.25027320253e-06 | 1.281530e-05 |
| Bacteria_Lentisphaerae_Lentisphaeria_Victivallales_PAC001406_f_PAC001699_g | 3.73346477019 | positive | 3.4356125192 | 0.0001569807508 | 3.387479e-04 |
| Bacteria_Firmicutes_Clostridia_Clostridiales_Christensenellaceae_PAC001435_g | 3.83758843824 | positive | 3.45697870432 | 3.09557282508e-07 | 4.230612e-06 |
| Bacteria_Bacteroidetes_Bacteroidia_Bacteroidales_Anaerocella_f_Anaerocella | 3.66275783168 | positive | 3.40862468364 | 0.00869154345315 | 1.048098e-02 |
| Bacteria_Firmicutes_Clostridia_Clostridiales_Christensenellaceae_PAC001207_g | 3.97924477841 | positive | 3.09737674485 | 0.0133885572976 | 1.483597e-02 |
| Bacteria_Tenericutes_Mollicutes_PAC001057_o_PAC000197_f_PAC001323_g | 3.72482180868 | positive | 3.32806598568 | 0.000403579194411 | 7.194238e-04 |
| Bacteria_Firmicutes_Erysipelotrichi_Erysipelotrichales_Erysipelotrichaceae_EU844830_g | 3.48287358361 | positive | 3.14736822926 | 2.25086703411e-05 | 9.228555e-05 |
| Bacteria_Elusimicrobia_Elusimicrobia_c_Elusimicrobiales_Elusimicrobiaceae_Elusimicrobium | 3.72043495843 | positive | 3.47760091448 | 4.58314796636e-05 | 1.445454e-04 |
| Bacteria_Bacteroidetes_Bacteroidia_Bacteroidales_Muribaculaceae_PAC001286_g | 3.95488543255 | positive | 3.50052212172 | 0.000204123974702 | 4.184541e-04 |
| Bacteria_Bacteroidetes_Bacteroidia_Bacteroidales_Prevotellaceae_PAC001421_g | 3.82823011473 | positive | 3.33030123796 | 0.00242980017251 | 3.435235e-03 |
| Bacteria_Firmicutes_Clostridia_Clostridiales_Mogibacterium_f_PAC001609_g | 3.77427359532 | positive | 3.06697643822 | 4.00010579732e-05 | 1.366703e-04 |
| Bacteria_Cyanobacteria_PAC002560_c_PAC000393_o_GU174155_f_AB506418_g | 3.61066016309 | positive | 3.32504528816 | 0.000105538041563 | 2.545329e-04 |
| Bacteria_Spirochaetes_Spirochaetia_Spirochaetales_Spirochaetaceae_Treponema | 4.25204353497 | positive | 3.80644396136 | 0.000135917069957 | 3.095889e-04 |
| Bacteria_Firmicutes_Clostridia_Clostridiales_Lachnospiraceae_Dorea | 3.96189547367 | positive | 3.63807429245 | 1.19810758957e-05 | 5.458046e-05 |
| Bacteria_Firmicutes_Clostridia_Clostridiales_Christensenellaceae_FJ848448_g | 3.34765520776 | positive | 3.00785483258 | 0.000236136967602 | 4.610293e-04 |
| Bacteria_Firmicutes_Clostridia_Clostridiales_Ruminococcaceae_AY854276_g | 3.39445168083 | positive | 3.07168984009 | 0.00165979899196 | 2.430420e-03 |
| Bacteria_Firmicutes_Clostridia_Clostridiales_Christensenellaceae_PAC001115_g | 3.56427143044 | positive | 3.13617417619 | 0.00114684395165 | 1.808485e-03 |
| Bacteria_Firmicutes_Clostridia_Clostridiales_Peptostreptococcaceae_Other | 3.38738982634 | positive | 3.13191108297 | 2.18424053291e-07 | 4.230612e-06 |
| Archaea_Euryarchaeota_Thermoplasmata_Methanomassiliicoccales_Methanomassiliicoccaceae_Methanogranum | 4.52131246414 | positive | 4.17194411303 | 7.90876105171e-06 | 4.632274e-05 |
| Bacteria_Bacteroidetes_Bacteroidia_Bacteroidales_AC160630_f_AB494828_g | 4.3384564936 | positive | 3.90346192899 | 3.86883626296e-05 | 1.366703e-04 |
| Bacteria_Firmicutes_Clostridia_Clostridiales_Ruminococcaceae_PAC000748_g | 4.47031246717 | positive | 3.93546150276 | 0.000568770113602 | 9.716489e-04 |
| Bacteria_Bacteroidetes_Bacteroidia_Bacteroidales_Prevotellaceae_GU304053_g | 3.58433122437 | positive | 3.14644382872 | 5.06191297982e-05 | 1.482417e-04 |
| Bacteria_Actinobacteria_Actinobacteria_c_Bifidobacteriales_Bifidobacteriaceae_Bifidobacterium | 3.80978409825 | positive | 3.59694002858 | 0.000311632343474 | 5.807694e-04 |
| Bacteria_Bacteroidetes_Bacteroidia_Bacteroidales_RF16_f_GU303877_g | 3.97374343766 | positive | 3.59362822913 | 2.06131349442e-07 | 4.230612e-06 |
| Bacteria_Firmicutes_Negativicutes_Selenomonadales_Selenomonadaceae_Anaerovibrio | 3.45127459754 | positive | 3.00780454954 | 0.00122809057049 | 1.864878e-03 |
| Bacteria_Firmicutes_Clostridia_Clostridiales_Ruminococcaceae_Eubacterium_g23 | 4.50623435961 | positive | 3.71495167048 | 0.0194698614284 | 2.100696e-02 |
| Bacteria_Bacteroidetes_Bacteroidia_Bacteroidales_AC160630_f_EU464332_g | 3.56110138365 | positive | 3.27783363985 | 2.62355834912e-06 | 2.151318e-05 |
| Bacteria_Firmicutes_Erysipelotrichi_Erysipelotrichales_Erysipelotrichaceae_Catenibacterium | 4.23451728351 | positive | 3.98456392853 | 6.61090811864e-05 | 1.806982e-04 |
| Bacteria_Verrucomicrobia_Opitutae_Puniceicoccales_GU305779_f_EU469726_g | 4.04813956549 | negative | 3.78524675304 | 0.0457639644244 | 4.576396e-02 |
| Bacteria_Firmicutes_Clostridia_Clostridiales_Ruminococcaceae_PAC000661_g | 4.92093455038 | negative | 4.51387508876 | 0.00094026081056 | 1.542028e-03 |

| | | | | | |
|---|---|---|---|---|---|
| Bacteria_Firmicutes_Clostridia_Clostridiales_Ruminococcaceae_Pseudoflavonifractor | 4.08695272426 | negative | 3.61231811975 | 0.00576317454247 | 7.160308e-03 |
| Bacteria_Bacteroidetes_Bacteroidia_Bacteroidales_Bacteroidaceae_Bacteroides | 3.95718145594 | negative | 3.57694498821 | 0.00983564937433 | 1.152176e-02 |
| Bacteria_Firmicutes_Clostridia_Clostridiales_Lachnospiraceae_KE159571_g | 3.84198480459 | negative | 3.54860514434 | 0.0404407340891 | 4.145175e-02 |
| Bacteria_Firmicutes_Clostridia_Clostridiales_Ruminococcaceae_PAC000683_g | 3.64673038625 | negative | 3.1849476707 | 0.0279628274465 | 2.939682e-02 |

LEfSe results for nematode positive and negative individuals at the Bacterial genus level

| Genus | log(highest_class_average) | class | LDA_effect_size | p_value | FDR_corrected |
|---|---|---|---|---|---|
| Bacteria_Verrucomicrobia_Opitutae_Puniceicoccales_GU305779_f_EU469726_g | 4.3214666178 | negative | 3.93053097656 | 0.0105624111928 | 0,03 |
| Bacteria_Firmicutes_Clostridia_Clostridiales_Ruminococcaceae_Pseudoflavonifractor | 4.26395868429 | negative | 3.68890539278 | 0.00597128984761 | 0,03 |
| Bacteria_Firmicutes_Clostridia_Clostridiales_Lachnospiraceae_Acetatifactor | 3.68124123738 | negative | 3.40573628537 | 0.0210130157161 | 0,03 |
| Bacteria_Firmicutes_Clostridia_Clostridiales_Lachnospiraceae_GU302778_g | 3.67793860183 | negative | 3.3444230546 | 0.0104305136762 | 0,03 |
| Bacteria_Firmicutes_Clostridia_Clostridiales_Lachnospiraceae_AB185516_g | 3.59005108385 | negative | 3.34286929952 | 0.014247525083 | 0,03 |
| Bacteria_Firmicutes_Clostridia_Clostridiales_Lachnospiraceae_AB185775_g | 3.54067067775 | negative | 3.19516752249 | 0.0264873438632 | 0,03 |
| Bacteria_Firmicutes_Clostridia_Clostridiales_Ruminococcaceae_Oscillibacter | 4.37748838338 | positive | 3.98466058699 | 0.00733793421287 | 0,03 |
| Bacteria_Verrucomicrobia_Kiritimatiellae_EF436358_o_EF436358_f_EF436358_g | 4.30556631352 | positive | 3.81506946428 | 0.03160658387 | 0,03 |
| Bacteria_Firmicutes_Clostridia_Clostridiales_Ruminococcaceae_PAC000748_g | 4.29622628726 | positive | 3.7468178587 | 0.019174587927 | 0,03 |
| Bacteria_Bacteroidetes_Bacteroidia_Bacteroidales_AC160630_f_AB494828_g | 4.09760432887 | positive | 3.67848529399 | 0.0187392946281 | 0,03 |
| Bacteria_Spirochaetes_Spirochaetia_Spirochaetales_Spirochaetaceae_Treponema | 4.03802374005 | positive | 3.43219654794 | 0.0256035270427 | 0,03 |
| Bacteria_Firmicutes_Clostridia_Clostridiales_Christensenellaceae_PAC001207_g | 4.03039730086 | positive | 3.61896108759 | 0.0225293803007 | 0,03 |
| Bacteria_Firmicutes_Clostridia_Clostridiales_Ruminococcaceae_Ruminococcus_g2 | 3.82575058135 | positive | 3.48440313381 | 0.0101453236414 | 0,03 |
| Bacteria_Firmicutes_Clostridia_Clostridiales_Lachnospiraceae_Roseburia | 3.81888541459 | positive | 3.3980902194 | 0.0120524412218 | 0,03 |
| Bacteria_Firmicutes_Clostridia_Clostridiales_Ruminococcaceae_PAC000672_g | 3.71558555189 | positive | 3.26020316406 | 0.00463411567697 | 0,03 |
| Bacteria_Firmicutes_Clostridia_Clostridiales_Ruminococcaceae_Subdoligranulum | 3.70671778234 | positive | 3.35057954835 | 0.0252870879239 | 0,03 |
| Bacteria_Firmicutes_Clostridia_Clostridiales_Lachnospiraceae_Dorea | 3.6565772914 | positive | 3.33211582907 | 0.0123503868826 | 0,03 |
| Bacteria_Firmicutes_Clostridia_Clostridiales_Lachnospiraceae_Coprococcus_g2 | 3.652246341 | positive | 3.05013964503 | 0.0359614694889 | 0,04 |
| Bacteria_Firmicutes_Clostridia_Clostridiales_Mogibacterium_f_PAC001609_g | 3.64295887941 | positive | 3.1648732704 | 0.0060155294173 | 0,03 |
| Bacteria_Firmicutes_Clostridia_Clostridiales_Mogibacterium_f_PAC001236_g | 3.62736585659 | positive | 3.26753641535 | 0.00260007567721 | 0,03 |
| Bacteria_Bacteroidetes_Bacteroidia_Bacteroidales_RF16_f_GU303877_g | 3.61700034112 | positive | 3.1668716869 | 0.0172321437214 | 0,03 |
| Bacteria_Firmicutes_Clostridia_Clostridiales_Christensenellaceae_PAC001141_g | 3.52244423351 | positive | 3.16676811187 | 0.0152619762344 | 0,03 |
| Bacteria_Firmicutes_Clostridia_Clostridiales_Christensenellaceae_PAC001435_g | 3.51982799378 | positive | 3.16543506526 | 0.0104569932312 | 0,03 |
| Bacteria_Lentisphaerae_Lentisphaeria_Victivallales_PAC001406_f_PAC001699_g | 3.32735893439 | positive | 3.05747562673 | 0.0324748636806 | 0,03 |

LEfSe results for entamoeba positive and negative individuals at the Bacterial genus level

| Genus | log(highest_class_average) | class | LDA_effect_size | p_value | FDR_corrected |
|---|---|---|---|---|---|
| Bacteria_Cyanobacteria_Vampirovibrio_c_FR888536_o_FR888536_f_FR888536_g | 4.73639650228 | negative | 4.26742410244 | 0.0295740806852 | 3.473549e-02 |
| Bacteria_Bacteroidetes_Bacteroidia_Bacteroidales_Bacteroidaceae_Bacteroides | 4.37106786227 | negative | 3.92025501506 | 0.0232486307063 | 3.355713e-02 |
| Bacteria_Proteobacteria_Alphaproteobacteria_Rhodospirillales_Rhodospirillaceae_LARJ_g | 4.14379520385 | negative | 3.70488087352 | 0.0265517881232 | 3.355713e-02 |
| Bacteria_Firmicutes_Erysipelotrichi_Erysipelotrichales_Erysipelotrichaceae_EU771685_g | 4.00324505481 | negative | 3.60092962483 | 0.0455117655462 | 4.697989e-02 |
| Bacteria_Bacteroidetes_Bacteroidia_Bacteroidales_Prevotellaceae_Paraprevotella | 3.71600334363 | negative | 3.37388044045 | 0.0204273409994 | 3.355713e-02 |
| Bacteria_Firmicutes_Clostridia_Clostridiales_Lachnospiraceae_AF349416_g | 3.6359861118 | negative | 3.3359124895 | 0.0262533021249 | 3.355713e-02 |
| Bacteria_Firmicutes_Clostridia_Clostridiales_Christensenellaceae_PAC001440_g | 3.58546072951 | negative | 3.27340506025 | 0.00593705031643 | 3.355713e-02 |
| Bacteria_Firmicutes_Clostridia_Clostridiales_Lachnospiraceae_PAC001138_g | 3.54406804435 | negative | 3.0479632524 | 0.0252449679209 | 3.355713e-02 |

| | | | | | |
|---|---|---|---|---|---|
| Bacteria_Firmicutes_Erysipelotrichi_Erysipelotrichales_Erysipelotrichaceae_Clostridium_g6 | 3.51851393988 | negative | 3.21884887414 | 0.0204273409994 | 3.355713e-02 |
| Bacteria_Firmicutes_Clostridia_Clostridiales_Ruminococcaceae_Paludicola | 3.49136169383 | negative | 3.17948306312 | 0.0222710509188 | 3.355713e-02 |
| Bacteria_Tenericutes_Mollicutes_Mycoplasmatales_Mycoplasmataceae_f1_Mycoplasma_g12 | 3.43136376416 | negative | 3.19978817473 | 0.0204273409994 | 3.355713e-02 |
| Bacteria_Firmicutes_Clostridia_Clostridiales_Lachnospiraceae_PAC001166_g | 3.34733001532 | negative | 3.16845351661 | 0.0204273409994 | 3.355713e-02 |
| Bacteria_Bacteroidetes_Bacteroidia_Bacteroidales_Muribaculaceae_FJ880190_g | 3.33745926129 | negative | 3.22826498674 | 0.0204273409994 | 3.355713e-02 |
| Bacteria_Firmicutes_Clostridia_Clostridiales_Ruminococcaceae_EU381487_g | 2.96614173274 | negative | 3.06851916657 | 1.77467444776e-06 | 5.678958e-05 |
| Bacteria_Actinobacteria_Coriobacteriia_Coriobacteriales_Coriobacteriaceae_PAC002509_g | 2.39794000867 | negative | 3.33615710582 | 0.0100673834117 | 3.355713e-02 |
| Bacteria_Firmicutes_Clostridia_Clostridiales_Lachnospiraceae_Other | 4.58752824157 | positive | 4.00505307966 | 0.00855990307933 | 3.355713e-02 |
| Bacteria_Firmicutes_Clostridia_Clostridiales_Ruminococcaceae_Eubacterium_g23 | 4.43907553006 | positive | 3.92873325574 | 0.0128859001381 | 3.355713e-02 |
| Bacteria_Firmicutes_Clostridia_Clostridiales_Ruminococcaceae_PAC000748_g | 4.29472249246 | positive | 3.93137517672 | 0.0252033345382 | 3.355713e-02 |
| Bacteria_Bacteroidetes_Bacteroidia_Bacteroidales_Muribaculaceae_Other | 4.0699948863 | positive | 3.75794488221 | 0.00126995290015 | 2.031925e-02 |
| Bacteria_Spirochaetes_Spirochaetia_Spirochaetales_Spirochaetaceae_Treponema | 4.0209006354 | positive | 3.54283342765 | 0.0439857176923 | 4.691810e-02 |
| Bacteria_Firmicutes_Clostridia_Clostridiales_Christensenellaceae_PAC001207_g | 4.01234666272 | positive | 3.66023615994 | 0.0272651684095 | 3.355713e-02 |
| Bacteria_Firmicutes_Bacilli_Bacillales_Staphylococcaceae_Staphylococcus | 3.7696520656 | positive | 3.47283892152 | 0.0303935495249 | 3.473549e-02 |
| Bacteria_Firmicutes_Clostridia_Clostridiales_Christensenellaceae_PAC001437_g | 3.75746665152 | positive | 3.32387280607 | 0.0119697237502 | 3.355713e-02 |
| Bacteria_Firmicutes_Clostridia_Clostridiales_Lachnospiraceae_Coprococcus_g2 | 3.70578417845 | positive | 3.41069757427 | 0.0470457735647 | 4.704577e-02 |
| Bacteria_Firmicutes_Clostridia_Clostridiales_Ruminococcaceae_Other | 3.68039876479 | positive | 3.25059456493 | 0.00760296810544 | 3.355713e-02 |
| Bacteria_Firmicutes_Clostridia_Clostridiales_Mogibacterium_f_PAC001609_g | 3.61303012522 | positive | 3.20957232947 | 0.024088582748 | 3.355713e-02 |
| Bacteria_Firmicutes_Erysipelotrichi_Erysipelotrichales_Erysipelotrichaceae_Coprobacillus | 3.44061623345 | positive | 3.08563813937 | 0.033669712209 | 3.715279e-02 |
| Bacteria_Firmicutes_Clostridia_Clostridiales_Lachnospiraceae_Eubacterium_g5 | 3.36739926595 | positive | 3.05878932891 | 0.0242709421247 | 3.355713e-02 |
| Bacteria_Firmicutes_Clostridia_Clostridiales_Ruminococcaceae_Eubacterium_g8 | 3.36566208571 | positive | 3.04604137592 | 0.0176595645784 | 3.355713e-02 |
| Bacteria_Firmicutes_Clostridia_Clostridiales_Clostridiaceae_Clostridium | 3.3478970535 | positive | 3.06119019824 | 0.0232891734999 | 3.355713e-02 |
| Bacteria_Actinobacteria_Coriobacteriia_Coriobacteriales_Coriobacteriaceae_Collinsella | 3.31592155107 | positive | 3.0397359074 | 0.0238066905223 | 3.355713e-02 |
| Bacteria_Firmicutes_Clostridia_Clostridiales_Ruminococcaceae_Monoglobus | 3.23927037189 | positive | 3.01345004259 | 0.00311641304091 | 3.324174e-02 |

## Supplementary Table 5: *Blastocystis* placement tree

| Method | Accession | Subtype original | Subtype current | Host | Reference |
|---|---|---|---|---|---|
| Initial tree | AB070987 | ST2 | | Homo sapiens | Arisue et al. (2003) |
| Initial tree | AB070991 | ST7 | | Homo sapiens | Arisue et al. (2003) |
| Initial tree | AB070992 | ST3 | | Homo sapiens | Arisue et al. (2003) |
| Initial tree | AB070994 | ST6 | | Gallus gallus domesticus | Arisue et al. (2003) |
| Initial tree | AB071000 | ST4 | | Rattus norvegicus | Yoshikawa et al. (1998) |
| Initial tree | AB091237 | ST6 | | Homo sapiens | Arisue et al. (2003) |
| Initial tree | AB107961 | ST1 | | Sus scrofa domesticus | Abe (2003) |
| Initial tree | AB107963 | ST3 | | Sus scrofa domesticus | Abe (2003) |
| Initial tree | AB107964 | ST5 | | Sus scrofa domesticus | Abe (2003) |
| Initial tree | AB107965 | ST3 | | Bos taurus | Abe (2003) |
| Initial tree | AB107966 | ST5 | | Bos taurus | Abe (2003) |
| Initial tree | AB107968 | ST1 | | Chlorocebus pygerythrus;Cercopithecus aethiops;Mandrillus leucophaeus | Abe (2003) |
| Initial tree | AB107969 | ST2 | | Macaca nemestrina;Macaca silenus | Abe (2003) |
| Initial tree | AB107970 | ST8 | | Varecia variegata | Abe (2003) |
| Initial tree | AB107971 | ST8 | | Argusianus argus | Abe (2003) |
| Initial tree | AB107973 | ST7 | | Anser cygnoides | Abe (2003) |
| Initial tree | AF408425 | ST9 | | Homo sapiens | Yoshikawa et al. (2004) |
| Initial tree | AF408427 | ST7 | | Homo sapiens | Arisue et al. (2003) |
| Initial tree | AF439782 | ST1 | | Homo sapiens | Thathaisong et al. (2003) |
| Initial tree | AF538348 | Blastocystis hominis | | Sus scrofa domesticus | Thathaisong et al. (2003) |
| Initial tree | AY135404 | ST1 | | Sus scrofa domesticus | Noel et al. (2003) |
| Initial tree | AY135409 | ST7 | | Chicken | Noel et al. (2003) |
| Initial tree | AY135410 | ST7 | | Chicken | Noel et al. (2003) |
| Initial tree | AY135411 | ST6 | | Turkey | Noel et al. (2003) |
| Initial tree | AY135412 | ST7 | | Duck | Noel et al. (2003) |
| Initial tree | AY244620 | ST4 | | Homo sapiens | Yoshikawa et al. (2004) |
| Initial tree | AY266467 | AFJ96-T8 | | Leopard frog | Yoshikawa et al. (2004) |

| | | | | |
|---|---|---|---|---|
| Initial tree | AY266468 | AFJ96-U12 | Bulfrog | Yoshikawa et al. (2004) |
| Initial tree | AY266469 | ST5 | Toad | Yoshikawa et al. (2004) |
| Initial tree | AY266470 | AFJ96-H12 | Toad | Yoshikawa et al. (2004) |
| Initial tree | AY266471 | Blastocystis lapemi | Sea snake | Yoshikawa et al. (2004) |
| Initial tree | AY266472 | Blastocystis pythoni | Reticulated python | Yoshikawa et al. (2004) |
| Initial tree | AY266473 | ST | Red-footed tortoise | Yoshikawa et al. (2004) |
| Initial tree | AY266474 | Blastocystis cycluri | Rhino iguana | Yoshikawa et al. (2004) |
| Initial tree | AY266475 | Blastocystis sp. R44 | Rhino Iguana | Yoshikawa et al. (2004) |
| Initial tree | AY590107 | ST7 | Homo sapiens | Noel et al. (2005) |
| Initial tree | AY590108 | ST7 | Homo sapiens | Noel et al. (2005) |
| Initial tree | AY590115 | Blastocystis lapemi | Sea snake | Noel et al. (2005) |
| Initial tree | AY618265 | ST2 | Homo sapiens | Thathaisong et al. (2004) |
| Initial tree | AY956324 | ST2 | na | Thathaisong et al. (2005) |
| Initial tree | CABX01000063 | ST7 | na | Wincker et al. (2010) |
| Initial tree | CABX01000085 | ST7 | na | Wincker et al. (2010) |
| Initial tree | CABX01000137 | ST7 | na | Wincker et al. (2010) |
| Initial tree | CABX01000153 | ST7 | na | Wincker et al. (2010) |
| Initial tree | DQ186645 | ST17 | Cockroach | Yoshikawa et al. (2007) |
| Initial tree | DQ186646 | ST17 | Cockroach | Yoshikawa et al. (2007) |
| Initial tree | DQ366343 | ST7 | Homo sapiens | Yan et al. (2006) |
| Initial tree | EF079872 | ST7 | na | Yan et al. (2006) |
| Initial tree | EF209016 | GERA3b | Geochelone radiata | Kostka et al. (2008) |
| Initial tree | EF209017 | GERA3b | Geochelone radiata | Kostka et al. (2008) |
| Initial tree | EF209018 | GECA2 | Geochelone carbonaria | Kostka et al. (2008) |
| Initial tree | EF209019 | KINIX2 | Kinixys belliana | Kostka et al. (2008) |
| Initial tree | EF209020 | GEPA2 | Geochelone carbonaria | Kostka et al. (2008) |
| Initial tree | EF468654 | ST5 | Homo sapiens | Yan et al. (2007) |
| Initial tree | EU082109 | ST7 | Homo sapiens | Li et al. (2007) |
| Initial tree | EU427511 | ST16 | Macropus rufus | Yoshikawa et al. (2008) |

| | | | | |
|---|---|---|---|---|
| Initial tree | EU427512 | ST16 | Macropus rufus | Yoshikawa et al. (2008) |
| Initial tree | EU427514 | ST16 | Macropus rufus | Yoshikawa et al. (2008) |
| Initial tree | EU427515 | ST12 | Wallabia bicolor masterii | Yoshikawa et al. (2008) |
| Initial tree | EU445485 | ST6 | Gallus gallus domesticus | Rivera et al. (2008) |
| Initial tree | EU445486 | ST1 | Sus scrofa domesticus | Rivera et al. (2008) |
| Initial tree | EU445487 | ST2 | Sus scrofa domesticus | Rivera et al. (2008) |
| Initial tree | EU445489 | ST3 | unidentified primate | Rivera et al. (2008) |
| Initial tree | EU445491 | ST2 | unidentified primate | Rivera et al. (2008) |
| Initial tree | GQ223285 | ST17 | environment | Yubuki et al. (2010) |
| Initial tree | GU256902 | ST12 | Giraffe | Parkar et al. (2010) |
| Initial tree | GU256922 | ST11 | Elephant | Parkar et al. (2010) |
| Initial tree | GU256934 | ST13 | Quokka | Parkar et al. (2010) |
| Initial tree | GU992412 | ST2 | wastewater | Banaticla et al. (2011) |
| Initial tree | GU992419 | ST1 | wastewater | Banaticla et al. (2011) |
| Initial tree | HQ909889 | ST3 | Homo sapiens | Stensvold and Clark (2011) |
| Initial tree | HQ909890 | ST3 | Baboon | Stensvold and Clark (2011) |
| Initial tree | HQ909891 | ST3 | Colobus abyssinicus | Stensvold and Clark (2011) |
| Initial tree | JN682513 | ST4 | Homo sapiens | Stensvold et al. (2012) |
| Initial tree | KC138681 | ST9 | Homo sapiens | Engsbro and Stensvold (2012) |
| Initial tree | KC148205 | ST14 | Bos taurus | Alfellani et al. (2013) |
| Initial tree | KC148206 | ST14 | Moufflon | Alfellani et al. (2013) |
| Initial tree | KC148207 | ST10 | Camel | Alfellani et al. (2013) |
| Initial tree | KC148208 | ST17 | Gundi | Alfellani et al. (2013) |
| Initial tree | KC148209 | ST13 | Mouse deer | Alfellani et al. (2013) |
| Initial tree | KC148210 | ST15 | Camel | Alfellani et al. (2013) |
| Initial tree | KC148211 | ST15 | Gibbon | Alfellani et al. (2013) |
| Initial tree | KF002512 | ST4 | na | Ramirez et al. (2014) |
| Initial tree | KF002522 | ST2 | na | Ramirez et al. (2014) |
| Initial tree | KF002529 | ST1 | na | Ramirez et al. (2014) |

| | | | | |
|---|---|---|---|---|
| Initial tree | KF002558 | ST3 | na | Ramirez et al. (2014) |
| Initial tree | KF447161 | ST7 | na | Poirier et al. (2014) |
| Initial tree | KF447167 | ST7 | na | Poirier et al. (2014) |
| Initial tree | KF447168 | ST7 | na | Poirier et al. (2014) |
| Initial tree | KT438703 | ST9 | Homo sapiens | Yoshikawa et al. (2016) |
| Initial tree | KT438707 | Blastocystis sp isolate RTJ12-R4 | Astrochelys radiata | Yoshikawa et al. (2016) |
| Initial tree | KT438710 | Blastocystis sp isolate RTJ12-R45 | Geochelone elegans | Yoshikawa et al. (2016) |
| Initial tree | KT438715 | Blastocystis pythoni | Platysternon megacephalum megacephalum | Yoshikawa et al. (2016) |
| Initial tree | KT438717 | Blastocystis sp isolate RSJ13-R73 | Morelia viridis | Yoshikawa et al. (2016) |
| Initial tree | U26177 | ST4 | Cavis porcellus | Leipe et al. (1994) |
| Initial tree | U51151 | ST1 | Homo sapiens | Silberman et al. (1996) |
| Initial tree | U51152 | ST4 | Guinea pig | Silberman et al. (1996) |
| Initial tree | AY618267 | ST1 | na | Thathaisong et al. (2004) |
| Outgroup | AB032606 | Wobblia lunata | na | Moriya et al. (2002) |
| Outgroup | L27633 | Cafeteria roenbergensis | na | Leipe et al. (1994) |
| Outgroup | AB175931 | Saprolegnia sp. THMK0306 | na | Khomvilai et al. (2004) |
| Outgroup | ADCG02001924 | Saprolegnia parasitica CBS 223.65 | na | Jiang et al. (2013) |
| Outgroup | AF174366 | Cafeteria sp. EPM1 | na | Atkins et al. (2000) |
| Outgroup | AJ238655 | Saprolegnia ferax | na | Dick et al. (1999) |
| Outgroup | AY129062 | uncultured marine eukaryote | na | Worden et al. (2006) |
| Outgroup | AY520454 | marine gliding biciliate TCS-2004 | na | Cavalier-Smith et al. (2006) |
| Outgroup | AY827850 | Cafeteria roenbergensis | na | Scheckenbach et al. (2005) |
| Outgroup | AY827851 | Cafeteria roenbergensis | na | Scheckenbach et al. (2005) |
| Outgroup | AY916583 | uncultured eukaryote | na | Luo et al. (2005) |
| Outgroup | EF620522 | uncultured bicosoecid | na | Massana et al. (2007) |
| Outgroup | EU446333 | uncultured marine eukaryote | na | Alexander et al. (2009) |
| Outgroup | FJ794911 | Saprolegnia sp. SAP4 | na | Wolinska et al. (2009) |
| Outgroup | FJ794913 | Saprolegnia sp. SAP1 | na | Wolinska et al. (2009) |
| Outgroup | GU170211 | Stramenopile sp. MESS12 | na | Park et al. (2010) |

| | | | | | |
|---|---|---|---|---|---|
| Outgroup | GU479947 | uncultured Saprolegniales | | na | Lara et al. (2010) |
| Outgroup | GU479948 | uncultured Saprolegniales | | na | Lara et al. (2010) |
| Outgroup | JQ692034 | uncultured Cafeteria | | na | Menrique et al. (2012) |
| Placement tree | AB070997 | ST2 | | Macaca fuscata | Yoshikawa et al. (1998) |
| Placement tree | AB107967 | ST1 | | Pongo pygmaeus | Abe (2003) |
| Placement tree | denovo1455 JB13 | | ST2 | Papio hamadryas | Present study |
| Placement tree | denovo2227 APF39 | | ST8 | Alouatta pigra | Present study |
| Placement tree | denovo229 JB13 | | ST1 | Papio anubis hamadryas Theropithecus gelada | Present study |
| Placement tree | denovo2430 JB13 | | ST2 | Papio hamadryas | Present study |
| Placement tree | denovo2834 JB15 | | ST1 | Papio hamadryas | Present study |
| Placement tree | denovo288 JB10 | | ST3 | Papio anubis hamadryas | Present study |
| Placement tree | denovo3163 JB25 | | ST2 | Papio anubis hamadryas | Present study |
| Placement tree | denovo319 RT2048 | | ST3 | Cercopithecus ascianus | Present study |
| Placement tree | denovo361 JB15 | | ST1 | Papio anubis hamadryas | Present study |
| Placement tree | denovo377 JB10 | | ST3 | Papio anubis hamadryas Theropithecus gelada | Present study |
| Placement tree | denovo491 RT2048 | | ST3 | Cercopithecus ascianus | Present study |
| Placement tree | denovo511 JB10 | | ST3 | Papio anubis hamadryas | Present study |
| Placement tree | denovo512 JB10 | | ST1 | Papio anubis hamadryas | Present study |
| Placement tree | denovo550 RT2021 | | ST3 | Cercopithecus ascianus | Present study |
| Placement tree | denovo841 APF37 | | ST11 | Pan troglodytes | Present study |
| Placement tree | DQ232788 | ST3 | | unidentified primate | Scicluna et al. (2006) |
| Placement tree | DQ232789 | ST3 | | unidentified primate | Scicluna et al. (2006) |
| Placement tree | DQ232790 | ST3 | | unidentified primate | Scicluna et al. (2006) |
| Placement tree | DQ232791 | ST3 | | unidentified primate | Scicluna et al. (2006) |
| Placement tree | DQ232792 | ST3 | | unidentified primate | Scicluna et al. (2006) |
| Placement tree | DQ232797 | ST3 | | Macaca arctoides | Scicluna et al. (2006) |
| Placement tree | DQ462716 | ST3 | | Lagothrix lagotricha | Victory et al. (2006) |
| Placement tree | DQ462722 | ST3 | | Lagothrix lagotricha | Victory et al. (2006) |
| Placement tree | DQ462724 | ST3 | | Lagothrix lagotricha | Victory et al. (2006) |

| | | | | |
|---|---|---|---|---|
| Placement tree | HQ286904 | ST1 | Pan troglodytes | Petrasova et al. (2011) |
| Placement tree | HQ286905 | ST1 | Pan troglodytes | Petrasova et al. (2011) |
| Placement tree | HQ286906 | ST1 | Pan troglodytes | Petrasova et al. (2011) |
| Placement tree | HQ286907 | ST1 | Pan troglodytes | Petrasova et al. (2011) |
| Placement tree | HQ286908 | ST3 | Chlorocebus aethiops pygerythrus | Petrasova et al. (2011) |
| Placement tree | HQ286909 | ST2 | Chlorocebus aethiops pygerythrus | Petrasova et al. (2011) |
| Placement tree | HQ286910 | ST2 | Chlorocebus aethiops pygerythrus | Petrasova et al. (2011) |
| Placement tree | HQ286911 | ST1 | Chlorocebus aethiops pygerythrus | Petrasova et al. (2011) |
| Placement tree | HQ286912 | ST1 | Chlorocebus aethiops pygerythrus | Petrasova et al. (2011) |
| Placement tree | HQ286913 | ST1 | Chlorocebus aethiops pygerythrus | Petrasova et al. (2011) |
| Placement tree | HQ286914 | ST2 | Colobus guereza | Petrasova et al. (2011) |
| Placement tree | HQ286915 | ST5 | Colobus guereza | Petrasova et al. (2011) |
| Placement tree | HQ286916 | ST3 | Colobus guereza | Petrasova et al. (2011) |
| Placement tree | HQ641637 | ST1 | Hapalemur aureus | Santin et al. (2011) |
| Placement tree | HQ641638 | ST1 | Hapalemur aureus | Santin et al. (2011) |
| Placement tree | HQ641639 | ST1 | Hapalemur aureus | Santin et al. (2011) |
| Placement tree | HQ641640 | ST1 | Hapalemur aureus | Santin et al. (2011) |
| Placement tree | HQ641641 | ST1 | Hapalemur aureus | Santin et al. (2011) |
| Placement tree | HQ641642 | ST1 | Cercopithecus hamlyni | Santin et al. (2011) |
| Placement tree | HQ641643 | ST2 | Cercopithecus hamlyni | Santin et al. (2011) |
| Placement tree | HQ641644 | ST2 | Cercopithecus hamlyni | Santin et al. (2011) |
| Placement tree | HQ641645 | ST3 | Cercopithecus hamlyni | Santin et al. (2011) |
| Placement tree | HQ641646 | ST3 | Cercopithecus hamlyni | Santin et al. (2011) |
| Placement tree | HQ641647 | ST3 | Cercopithecus hamlyni | Santin et al. (2011) |
| Placement tree | HQ641648 | ST3 | Cercopithecus hamlyni | Santin et al. (2011) |
| Placement tree | HQ641649 | ST3 | Cercopithecus hamlyni | Santin et al. (2011) |
| Placement tree | HQ641650 | ST3 | Cercopithecus hamlyni | Santin et al. (2011) |
| Placement tree | HQ641651 | ST3 | Cercopithecus hamlyni | Santin et al. (2011) |
| Placement tree | HQ641652 | ST4 | Lemur catta | Santin et al. (2011) |

| | | | | | |
|---|---|---|---|---|---|
| Placement tree | HQ641653 | ST3 | | Mandrillus leucophaeus | Santin et al. (2011) |
| Placement tree | HQ641654 | ST2 | | Gorilla gorilla | Santin et al. (2011) |
| Placement tree | HQ641655 | ST2 | | Gorilla gorilla | Santin et al. (2011) |
| Placement tree | HQ641656 | ST3 | | Cercocebus atys | Santin et al. (2011) |
| Placement tree | HQ641657 | ST3 | | Cercocebus neglectus | Santin et al. (2011) |
| Placement tree | JX158496 | na | ST4 | Gorilla gorilla gorilla | Hamad et al. (2014) |
| Placement tree | JX158683 | na | ST4 | Gorilla gorilla gorilla | Hamad et al. (2014) |
| Placement tree | JX158946 | na | ST4 | Gorilla gorilla gorilla | Hamad et al. (2014) |
| Placement tree | JX159238 | na | ST4 | Gorilla gorilla gorilla | Hamad et al. (2014) |
| Placement tree | KF002511 | ST4 | | na | Ramirez et al. (2014) |
| Placement tree | KM374608 | ST8 | | Alouatta palliata aequatorialis | Helenbrook et al. (2015) |
| Placement tree | KM374610 | ST8 | | Alouatta palliata aequatorialis | Helenbrook et al. (2015) |
| Placement tree | KT591768 | ST2 | | Alouatta palliata | Villanueva et al. (2017) |
| Placement tree | KT591770 | ST2 | | Alouatta pigra | Villanueva et al. (2017) |
| Placement tree | KT591777 | ST2 | | Alouatta pigra | Villanueva et al. (2017) |
| Placement tree | KT591788 | ST2 | | Alouatta pigra | Villanueva et al. (2017) |
| Placement tree | KT591789 | ST2 | | Alouatta pigra | Villanueva et al. (2017) |
| Placement tree | KT591795 | ST2 | | Alouatta pigra | Villanueva et al. (2017) |
| Placement tree | KT591799 | ST2 | | Alouatta pigra | Villanueva et al. (2017) |
| Placement tree | KT591806 | ST2 | | Alouatta pigra | Villanueva et al. (2017) |
| Placement tree | KT591811 | ST2 | | Alouatta pigra | Villanueva et al. (2017) |
| Placement tree | KT591814 | ST2 | | Alouatta pigra | Villanueva et al. (2017) |
| Placement tree | KT591818 | ST2 | | Alouatta pigra | Villanueva et al. (2017) |
| Placement tree | KT591820 | ST2 | | Alouatta pigra | Villanueva et al. (2017) |
| Placement tree | KT591824 | ST2 | | Alouatta palliata | Villanueva et al. (2017) |
| Placement tree | KT591831 | ST2 | | Alouatta pigra | Villanueva et al. (2017) |
| Placement tree | KT591832 | ST2 | | Alouatta pigra | Villanueva et al. (2017) |
| Placement tree | KT591833 | ST2 | | Alouatta pigra | Villanueva et al. (2017) |
| Placement tree | KT591834 | ST2 | | Alouatta pigra | Villanueva et al. (2017) |

| | | | | |
|---|---|---|---|---|
| Placement tree | KT591835 | ST2 | Alouatta palliata | Villanueva et al. (2017) |
| Placement tree | KT591836 | ST2 | Alouatta pigra | Villanueva et al. (2017) |
| Placement tree | KT591837 | ST2 | Alouatta palliata | Villanueva et al. (2017) |
| Placement tree | KT591838 | ST2 | Alouatta pigra | Villanueva et al. (2017) |
| Placement tree | KT591839 | ST2 | Alouatta pigra | Villanueva et al. (2017) |
| Placement tree | KT591840 | ST2 | Alouatta pigra | Villanueva et al. (2017) |
| Placement tree | KT591848 | ST1 | Alouatta pigra | Villanueva et al. (2017) |
| Placement tree | KT591849 | ST1 | Alouatta palliata | Villanueva et al. (2017) |
| Placement tree | KT591850 | ST1 | Alouatta pigra | Villanueva et al. (2017) |
| Placement tree | KT591851 | ST1 | Alouatta pigra | Villanueva et al. (2017) |
| Placement tree | KT591854 | ST8 | Alouatta palliata | Villanueva et al. (2017) |
